# *Haemophilus ducreyi* Cutaneous Ulcer Strains Are Nearly Identical to Class I Genital Ulcer Strains

**DOI:** 10.1371/journal.pntd.0003918

**Published:** 2015-07-06

**Authors:** Dharanesh Gangaiah, Kristen M. Webb, Tricia L. Humphreys, Kate R. Fortney, Evelyn Toh, Albert Tai, Samantha S. Katz, Allan Pillay, Cheng-Yen Chen, Sally A. Roberts, Robert S. Munson, Stanley M. Spinola

**Affiliations:** 1 Department of Microbiology and Immunology, Indiana University School of Medicine, Indianapolis, Indiana, United States of America; 2 Department of Biology, Allegheny College, Meadville, Pennsylvania, United States of America; 3 Genomics Core, Tufts University School of Medicine, Boston, Massachusetts, United States of America; 4 Laboratory Reference and Research Branch, Division of STD Prevention, National Center for HIV/AIDS, Viral Hepatitis, STD and Tuberculosis Prevention, Centers for Disease Control and Prevention, Atlanta, Georgia, United States of America; 5 Department of Microbiology, Auckland District Health Board, Auckland, New Zealand; 6 The Center for Microbial Pathogenesis in the Research Institute at Nationwide Children’s Hospital, The Ohio State University College of Medicine, Columbus, Ohio, United States of America; 7 Department of Pediatrics, The Ohio State University College of Medicine, Columbus, Ohio, United States of America; 8 Department of Medicine, Indiana University School of Medicine, Indianapolis, Indiana, United States of America; 9 Department of Pathology and Laboratory Medicine, Indiana University School of Medicine, Indianapolis, Indiana, United States of America; 10 The Center for Immunobiology, Indiana University School of Medicine, Indianapolis, Indiana, United States of America; University of Tennessee, UNITED STATES

## Abstract

**Background:**

Although cutaneous ulcers (CU) in the tropics is frequently attributed to *Treponema pallidum* subspecies *pertenue*, the causative agent of yaws, *Haemophilus ducreyi* has emerged as a major cause of CU in yaws-endemic regions of the South Pacific islands and Africa. *H*. *ducreyi* is generally susceptible to macrolides, but CU strains persist after mass drug administration of azithromycin for yaws or trachoma. *H*. *ducreyi* also causes genital ulcers (GU) and was thought to be exclusively transmitted by microabrasions that occur during sex. In human volunteers, the GU strain 35000HP does not infect intact skin; wounds are required to initiate infection. These data led to several questions: Are CU strains a new variant of *H*. *ducreyi* or did they evolve from GU strains? Do CU strains contain additional genes that could allow them to infect intact skin? Are CU strains susceptible to azithromycin?

**Methodology/Principal Findings:**

To address these questions, we performed whole-genome sequencing and antibiotic susceptibility testing of 5 CU strains obtained from Samoa and Vanuatu and 9 archived class I and class II GU strains. Except for single nucleotide polymorphisms, the CU strains were genetically almost identical to the class I strain 35000HP and had no additional genetic content. Phylogenetic analysis showed that class I and class II strains formed two separate clusters and CU strains evolved from class I strains. Class I strains diverged from class II strains ~1.95 million years ago (mya) and CU strains diverged from the class I strain 35000HP ~0.18 mya. CU and GU strains evolved under similar selection pressures. Like 35000HP, the CU strains were highly susceptible to antibiotics, including azithromycin.

**Conclusions/Significance:**

These data suggest that CU strains are derivatives of class I strains that were not recognized until recently. These findings require confirmation by analysis of CU strains from other regions.

## Author Summary

Cutaneous ulcers (CU) in children living in equatorial Africa and the South Pacific islands have long been attributed to yaws, which is caused by *Treponema pallidum* subsp. *pertenue*. However, PCR-based cross sectional surveys done in yaws-endemic regions show that *Haemophilus ducreyi* is the leading cause of CU in these regions. *H*. *ducreyi* classically causes the genital ulcer (GU) disease chancroid and was once thought to be exclusively sexually transmitted. We show that CU strains obtained from Samoa and Vanuatu are genetically nearly identical to class 1 GU strains and contain no additional genetic content. The CU strains are highly susceptible to antibiotics, including azithromycin. The data suggest an urgent need to obtain and analyze CU isolates from Africa and other countries in the South Pacific and to search for environmental sources of the organism.

## Introduction

*Haemophilus ducreyi* classically causes chancroid, a sexually transmitted disease that presents as painful genital ulcers (GU), which are often accompanied by infected regional lymph nodes. Although the current global prevalence of chancroid is undefined due to syndromic management of genital ulcer disease and lack of surveillance programs, the worldwide prevalence of chancroid has declined over the last decade [1]. In addition to causing its own morbidity, chancroid facilitates the acquisition and transmission of the human immunodeficiency virus type 1 [1].

In addition to causing chancroid, *H*. *ducreyi* has been isolated from or its DNA has been detected in chronic cutaneous ulcers (CU) in yaws-endemic regions in the South Pacific islands and equatorial Africa [2–7]. Yaws is a chronic infection of skin, bone, and cartilage that occurs mainly in poor communities in tropical areas of Africa, Asia, and Latin America; yaws is caused by *Treponema pallidum* subspecies *pertenue*, which is closely related to *T*. *pallidum* subsp. *pallidum*, the cause of venereal syphilis. A prospective cohort study by Mitjà and colleagues in yaws-endemic villages of Papua New Guinea showed that *H*. *ducreyi* is a major cause of chronic CU in children younger than 15 years old [6]. In that study, nearly 60% of patients with ulcers had detectable lesional *H*. *ducreyi* DNA, while only 34% were positive for lesional *T*. *pallidum* subsp. *pertenue* DNA. Approximately 2% of the total population and more than 7% of the children aged 5–15 years had ulcers positive for *H*. *ducreyi* as detected by PCR. Similar findings were reported from yaws-endemic communities in the Solomon Islands [8].

Mass drug administration (MDA) of oral azithromycin (AZT) for yaws in Papua New Guinea with a population coverage rate of 84% reduced the prevalence of CU by 90% [9]. Although MDA significantly reduced the proportion of ulcers with *T*. *pallidum* subsp. *pertenue* DNA, the proportion of ulcers containing *H*. *ducreyi* DNA was not affected [9]. The presence of *H*. *ducreyi*-positive CU was also reported from districts of Ghana that had received several rounds of MDA of AZT for trachoma [7]. These data raise the possibility that CU strains may be resistant to AZT, exist in an environmental reservoir, or are so infectious that MDA at the above coverage rate fails to eradicate *H*. *ducreyi*.

Multilocus sequence analysis is frequently used to determine the genetic relatedness of bacterial strains. Based on analysis of 11 *H*. *ducreyi* genes, GU strains form two genetically distinct classes, designated class I and class II, which diverged from each other approximately five million years ago (mya) and may represent distinct species [10]. A similar analysis including four CU strains suggests that they are a subset of class I GU strains [11]. However, this analysis was limited by the fact that it was based on only three informative loci.

To obtain additional insights into the evolutionary relationship of CU and GU strains, here we performed whole-genome sequencing of CU strains isolated from patients infected in Samoa and Vanuatu and archived class I and class II GU strains. Due to the persistence of CU strains after MDA of AZT, we also determined the *in vitro* susceptibilities of CU and GU strains to antimicrobials used for the treatment of chancroid.

## Materials and Methods

### Bacterial strains and culture conditions

The 5 CU strains used in this study were the only strains available at the time the study was initiated (Table 1); their associated clinical features are listed in S1 Table. The class I and class II strains used in this study were chosen because these strains had been previously analyzed by multilocus sequencing (Table 1) [10]. 35000HP, whose genome has been sequenced (GenBank accession no. NC_002940.2), was used as the reference strain in this study; 35000HP was isolated from a volunteer who was experimentally infected on the arm with strain 35000 and has been extensively characterized in human inoculation experiments [12, 13]. The *H*. *ducreyi* strains were grown on Columbia agar plates or in Columbia broth supplemented with 1% bovine hemoglobin (Sigma-Aldrich), 1% IsoVitaleX, and 5% fetal bovine serum (Hyclone) at 33°C with 5% CO_2_.

**Table 1:** *H*. *ducreyi* strains selected for the study and their genome sequencing statistics.

**Strain**	Class	Origin	Year of isolation	Total no. of reads	Genome coverage (fold)	Total no. of contigs	Genome size (Mb)	GC%	% coverage/ % identity to 35000HP
****35000****	Class I	Winnipeg, Canada	1975–8	-	-	-	1.70	38.1	-
****NZS1**** ^a^	CU	Samoa	2006	876968	155	68	1.60	38.1	97/99
****NZS2****	CU	Samoa	2006	1637818	290	40	1.62	38	98/99
****NZS3****	CU	Samoa	2006	1070271	189	50	1.62	38.1	98/99
****NZS4****	CU	Samoa	2007	900034	159	88	1.60	38.1	97/99
****NZV1****	CU	Vanuatu	2014	2707811	400	95	1.56	38.2	95/99
****82–029362****	Class I	California, USA	1982	1048968	186	63	1.64	38.2	98/99
****6644****	Class I	Massachusetts, USA	1989	3585289	529	118	1.74	38.6	98/99
****HD183****	Class I	Singapore	1982	981095	174	129	1.67	38.6	98/99
****HMC46****	Class I	Kenya	1995	520008	92	123	1.63	38.3	97/99
****HMC56****	Class I	Dominican Republic	1995	1151430	204	59	1.59	38.1	95/99
****33921****	Class II	Nairobi, Kenya	Unk	927423	164	67	1.59	37.9	89/98
****CIP542****	Class II	Hanoi, Vietnam	1954	4036804	596	54	1.52	37.8	89/98
****DMC64****	Class II	Bangladesh	Unk	1064765	188	46	1.57	37.9	90/98
****DMC111****	Class II	Bangladesh	Unk	1049051	186	50	1.54	37.9	90/98

^a^The description NZS is defined as a strain isolated in New Zealand from a patient who had acquired disease in Samoa; NZV, from a patient who had acquired disease in Vanuatu.

Unk, unknown

### DNA extraction

Genomic DNA was extracted from *H*. *ducreyi* strains using the DNeasy Blood & Tissue kit (Qiagen) and quantified using the Quant-It High Sensitivity dsDNA Assay kit (Life Technologies).

### Library preparation, sequencing, assembly, and annotation

The sequencing libraries were prepared using the NexteraXT DNA Library Preparation kit (Illumina, Inc.) following the manufacturer’s instructions. Samples were multiplexed using the NexteraXT Dual Index Primer kit. Equimolar concentrations of indexed libraries were combined into a single pool and were sequenced at the Tufts University Genomics Core Facility. Paired-end 250-bp sequencing was performed on the Illumina MiSeq platform using the MiSeq V2 500 cycles chemistry. The *de novo* assembly was performed using Edena, with a customized bash script that optimizes the assembly process by optimizing three key Edena parameters [14]. The assembled contigs were annotated using the RAST online annotation tool [15].

### Contig ordering and estimation of genome conservation distance

A flow chart of comparative genome analysis of CU and GU strains is depicted in S1 Fig. For all comparative genome analyses in this study, the genome sequence of 35000HP was used as the reference. The *de novo* assembled contigs were ordered into Locally Collinear Blocks (LCBs) by Mauve Contig Mover (MCM) [16]. The breakpoints between LCBs were resolved by using BLAST analysis of the unaligned contigs produced by MCM, the breakpoint regions in the *de novo* assembled contigs, and by alignment of raw reads against 35000HP. After resolving the breakpoints, the ordered contigs were concatenated into draft genomes using Emboss 6.3.1. Pairwise genome conservation distances, which represent both gene content and sequence similarity, were estimated from draft genomes using ProgressiveMauve and plotted as heat map using CIMminer [17, 18].

### Nucleotide sequence accession numbers

The draft genome sequences for the 14 *H*. *ducreyi* strains NZS1, NZS2, NZS3, NZS4, 82–029362, 6644, HD183, HMC46, HMC56, NZV1, 33921, CIP542, DMC64, and DMC111 were deposited in GenBank under the accession numbers CP011218, CP011219, CP011220, CP011221, CP011222, CP011223, CP011224, CP011225, CP011226, CP011227, CP011228, CP011229, CP011230, and CP011231, respectively.

### Identification of genome rearrangements

Genome rearrangements were identified from multiple alignments of the draft genomes generated by ProgressiveMauve, BLAST Ring Image Generator, and nucleotide BLAST and from the assembly of raw reads against 35000HP by SeqMan NGen [17, 19, 20]. Because reference-based alignment can miss additional genes that might be absent in the 35000HP genome, the *de novo* assembled contigs that did not align to the 35000HP genome by ProgressiveMauve were aligned against other microbial genomes using translated nucleotide BLAST.

### Detection of single nucleotide polymorphisms (SNPs) and small insertions and deletions

SNPs and small insertions and deletions (indels, <10 bp) were detected using DNASTAR Lasergene (DNASTAR, Inc., Madison, WI). Briefly, the sequenced reads were assembled by SeqMan NGen against 35000HP. SNPs and indels were discovered by Seqman Pro using default parameters except that a minimum frequency of 90% reads and a minimum coverage of 50 reads were used for the analysis. SNPs were grouped as non-coding, synonymous, or nonsynonymous. Nonsynonymous SNPs were further categorized as substitutions, no-start, no-stop, nonsense, or frameshifts. All SNPs in the genomes of the CU strains were manually verified for accuracy.

### Diversity analyses

Diversity analyses of whole-genome nucleotide sequences and translated concatenated coding sequences were performed using Mega 6.0 [21]. The reliability of the diversity analyses was tested using 1000 bootstrap replicates.

### Detection of recombination

Recombination analysis was performed using the Phi test implemented in PhiPack and the likelihood ratio test implemented in TOPALi v2 [22, 23]. For both tests, a threshold *P* < 0.05 was used to define a recombination event.

### Phylogenetic analyses

Phylogenetic analyses were performed using Mega 6.0 and Realphy [21, 24]. Briefly, whole-genome alignments were imported into Mega 6.0 and subjected to model testing to identify the best-fit models of nucleotide substitution. Model testing identified Hasegawa-Kishino-Yano plus invariant sites plus gamma-distributed model as the best-fit nucleotide substitution model for our data. Using the best-fit model, phylogenetic analyses were performed with both whole-genome alignments and alignments of translated amino acid sequences from concatenated protein-coding regions using different methods of phylogeny reconstruction, including Maximum Likelihood, Maximum Parsimony, Minimum Evolution, and Neighbor Joining with different gap treatment approaches. We also inferred phylogenies using Realphy, which generates phylogenetic trees by merging alignments obtained by mapping to multiple reference genomes. A rooted Maximum Likelihood tree was reconstructed by including other *Pasteurellaceae* members (*Actinobacillus pleuropneumoniae*, *Mannheimia haemolytica*, *Pasteurella multocida*, *Aggregatibacter actinomycetemcomitans*, and *Haemophilus influenzae*) as outgroups. The reliability of all the trees generated was verified by 1000 bootstrap replicates.

### Estimation of divergence time

The times to the most recent common ancestor (MRCA) were estimated by Bayesian molecular clock method using Beast v1.8.1 [25]. Hasegawa-Kishino-Yano plus invariant sites plus gamma-distributed model and a relaxed clock model were used to account for variation in substitution rates. The results from the Beast analysis were visualized using Tracer v1.6. A best-fit tree was identified from the tree data generated by Beast using TreeAnnotator and visualized using FigTree v1.4.2. As described previously, we used a substitution rate of 4.5 × 10^−9^ per site per year to calibrate the tree [26].

### Evolutionary selection analyses

Selection analyses were performed using Mega 6.0 and Hyphy 2.1 [21, 27]. Briefly, protein-coding regions were extracted from the annotated genomes, ordered against 35000HP using MCM, concatenated using Emboss 6.3.1, and aligned using ProgressiveMauve [16, 17, 28]. The alignments were manually edited for accuracy to obtain a codon-delimited alignment, which was used for all the selection analyses. Rates of nonsynonymous (*d*_N_) and synonymous (*d*_S_) substitutions are widely used as a sensitive measure of selection occurring in a protein with *d*_N_ = *d*_S_, *d*_N_ > *d*_S_, and *d*_N_ < *d*_S_ indicating neutral, positive, and negative selection, respectively. Alignment-wide evidence for selection was tested using the codon-based Z test. For the codon-based Z test, we first calculated *d*_N_ and *d*_S_ and their variances using 1000 bootstrap replicates. We then used this information to test the null hypothesis of neutrality (*d*_N_ = *d*_S_) versus alternative hypothesis of positive (*d*_N_ > *d*_S_) or negative (*d*_N_ < *d*_S_) selection using a Z-test. A branch-site random effects likelihood test was used to test whether any of the branches in the tree are evolving under positive selection. A branchTestDNDS test was performed to test whether a prespecified branch of the tree is evolving under different selection strength than the rest of the tree [27]. Individual sites under positive or negative selection were identified using the single likelihood ancestral counting and fixed effects likelihood methods [27].

### Identification of genes encoding known *H*. *ducreyi* virulence determinants

The draft genomes were interrogated for the presence of genes that are required for the virulence of strain 35000HP in the human inoculation experiments, using nucleotide BLAST [13]. For identifying sequence variation, the nucleotide sequences of virulence genes were translated into amino acids and the translated sequences were aligned using Clustal Omega [29].

### Identification of genes encoding antimicrobial resistance determinants and antimicrobial susceptibility testing (AST)

The draft genomes were searched for the presence of known antimicrobial resistance genes using ResFinder with default parameters [30]. AST was performed using the agar dilution method as described previously with some modifications [31–33]. Briefly, *H*. *ducreyi* strains were grown on Columbia agar (Difco) containing 1% hemoglobin (BBL), 0.2% activated charcoal (Sigma-Aldrich), 5% fetal bovine serum (Atlanta Biologicals), and 1% IsoVitaleX (BBL) for 48 h at 33°C under microaerophilic conditions. The colonies were suspended into Mueller-Hinton (BBL) broth containing 1% IsoVitaleX and 0.002% Tween-80 (Sigma-Aldrich), passed through a 22-gauge needle and left at room temperature for 15 min. The optical density of the culture was adjusted to that of a 0.5 McFarland standard using a Spectronic 20 Plus spectrophotometer (Milton Roy). AST was performed on Mueller-Hinton II medium (BBL) containing 33% lysed horse blood (Remel), 5% fetal bovine serum, and 1% IsoVitaleX. The following antibiotics were tested: amoxicillin (AMX), amoxicillin/clavulanic acid (AMC; 2:1), azithromycin (AZT), ciprofloxacin (CIP), ceftriaxone (CRO), doxycycline (DOX), erythromycin (ERY), and penicillin (PEN; all from Sigma-Aldrich). The *H*. *ducreyi* strains CIP542, 35000HP, and the *H*. *influenzae* strain 49247 were used as controls. A 10^4^/ml suspension of each strain was delivered onto each plate with a Steer’s Replicator (CMI-Promex, Inc.), and the plates were dried for 15 min at room temperature. The minimal inhibitory concentrations were recorded after incubating the plates for 48 h at 33°C under microaerophilic conditions. The presence of three or fewer colonies was recorded as no growth.

## Results

### Whole-genome sequencing

Whole-genome sequencing generated between 0.5 and 4 million reads for each of the 14 strains (Table 1). The estimated genome sizes ranged from 1.52 Mb to 1.74 Mb, with an average GC content of 37.8% to 38.6% (Table 1). The total number of contigs for each strain ranged from 40 to 129 (Table 1). The estimated average genome coverage ranged from 92 to 596 fold (Table 1). Contig ordering generated 2 to 6 LCBs for the CU strains, 5–15 LCBs for the class I strains, and 12–16 LCBs for the class II strains. Inspection of the LCBs revealed that the majority of the putative breakpoints between LCBs occurred in genes that share high homology with other genes in the *H*. *ducreyi* genome such as *lspA1* and *lspA2*, genes encoding rRNAs, and bacteriophage-related genes and that the majority of the breakpoints did not contain any rearrangements.

### Genome conservation distance

**Fig 1:**
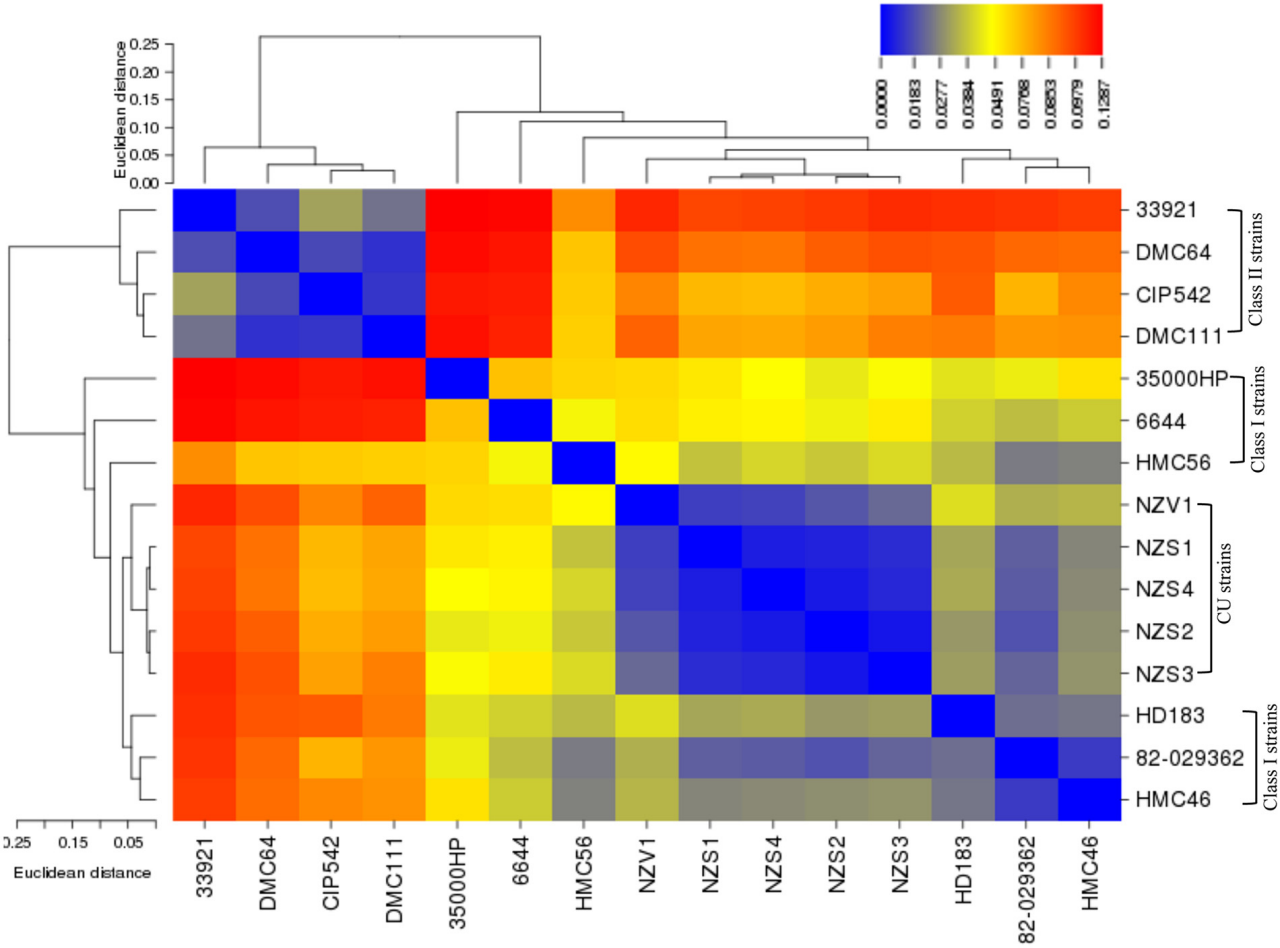
Whole-genome heat map showing the pairwise genome conservation distances of the CU and GU strains. The genome conservation distances were calculated using ProgressiveMauve; the distance matrix was plotted as an heat map using CIMminer with heat map clustering methods. Dendrograms across the top and left of the heat map show the relationship of genomes based on genome conservation. The strain names are indicated to the right and bottom of the heat map. Distance values range from 0.0000 to 0.1207, which are depicted by the gradient of colors ranging from dark blue (lowest distance value indicating high similarity between genomes) to red (highest distance value indicating low similarity between genomes).

Analysis of pairwise genome conservation distance of the draft genomes showed that CU strains form a subcluster within class I strains and that class II strains form a separate cluster from CU and class I strains (Fig 1).

### Genome rearrangements

**Fig 2:**
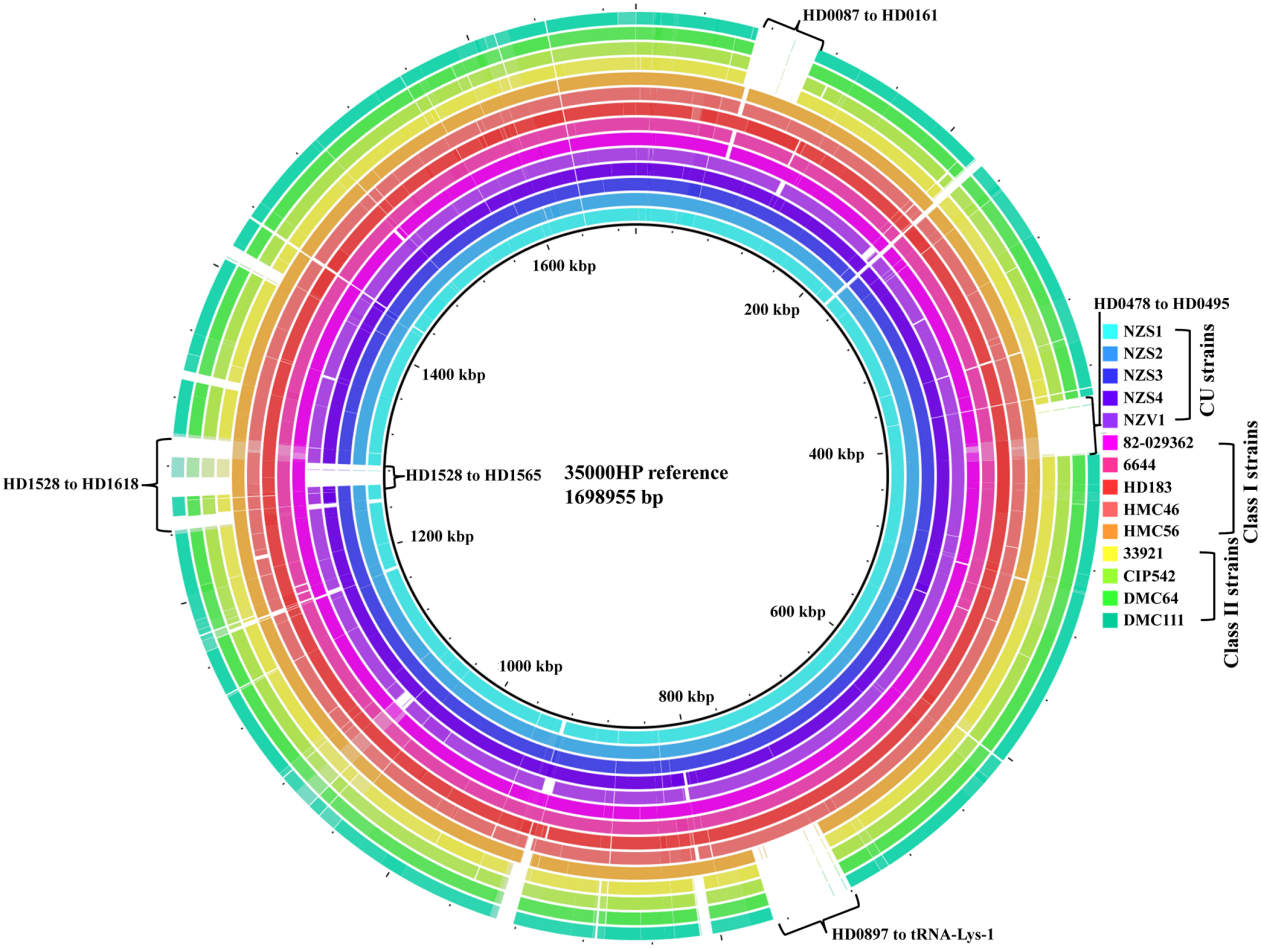
Circular visualization of multiple alignment of the CU and GU strains using Blast Ring Image Generator. The draft genomes of the CU and GU strains were mapped to 35000HP using nucleotide BLAST. The innermost ring showing the genomic positions represents the reference genome 35000HP. For clarity, each ring representing each strain is indicated by a different color. Positions covered by nucleotide BLAST are indicated as solid color, while positions not covered by nucleotide BLAST are indicated as white spaces. The gene coordinates of potential large-scale deletions are indicated.

Compared to 35000HP, all the CU strains consistently contained ~20-kb deletion (HD1528 to HD1565) in a bacteriophage locus that is homologous to *Pseudomonas aeruginosa* bacteriophage B3 and five small deletions that ranged in size from 30–767 bp (Fig 2 and S2 Table). The class I strain HMC56 contained a 50-kb deletion (HD0897 to tRNA-Lys-1) in a region homologous to the *H*. *influenzae* ICEHin1056 integrative conjugative element (Fig 2 and S3 Table). All the class II strains contained 3 major deletions of 37 kb (HD0087 to HD0161), 35 kb (HD0478 to HD0495) and 50 kb (HD0897 to tRNA-Lys-1), which are homologous to *Escherichia coli* bacteriophage D108, *Haemophilus* bacteriophage SuMu, and *H*. *influenzae* ICEHin1056, respectively (Fig 2 and S4 Table). The class II strains also contained several deletions (between HD1528 and HD1618) in a region that is homologous to *P*. *aeruginosa* bacteriophage B3 (Fig 2 and S4 Table). All the GU strains also contained several other small deletions as listed in S3 and S4 Tables.

Compared to 35000HP, we did not find any inversions in CU strains with the exception of NZV1, which contained an inversion of ~428 kb that spanned from HD0054 (*tuf*) to HD0659 (S2 Fig). Among the class I strains, HMC56 contained an inversion of ~300 kb that spanned from *glpA* (HD1157) to *lspA1* (HD1505) (S2 Fig). HD183 contained a ~161 kb inversion that spanned from *hhdA* (HD1327) to *lspA1* (HD1505) (S2 Fig). All the class II strains contained an inversion of ~17 kb that spanned from HD1532 to HD1565 (S2 Fig). However, BLAST analysis of the inversion breakpoints showed no major changes in their genetic content.

Compared to 35000HP, the CU strains, the class I strain 82–029362, and the class II strain CIP542 contained no additional genes in their genomes. All the remaining class I and class II strains contained several additional genes as listed in S5 Table.

### SNPs and genetic diversity

To get a deeper understanding of the relationship of CU strains to GU strains, we next performed whole-genome SNP analysis using 35000HP as a reference. CU strains differed from 35000HP by ~400 SNPs (Table 2). The class I strain HD183 differed from 35000HP by ~160 SNPs, while all other class I strains differed by ~2,000 SNPs (Table 2). The class II strains differed from 35000HP by ~30,000 SNPs (Table 2).

**Table 2:** Distribution of putative SNPs in the CU, class I and class II GU strains of *H*. *ducreyi* relative to 35000HP.

Class	Strain	Total no. of SNPs	Total no. of nonsynonymous SNPs
****CU strains****	NZS1	421	168
	NZS2	426	169
	NZS3	417	165
	NZS4	416	166
	NZV1	512	194
****Class I strains****	82–029362	2,121	587
	6644	2,091	580
	HD183	161	59
	HMC46	2,124	593
	HMC56	2,114	586
****Class II strains****	33921	31,351	8,357
	CIP542	30,581	8,078
	DMC64	30,616	8,125
	DMC111	30,572	8,112

SNPs were identified using DNASTAR Lasergene program and 35000HP as a reference. The SNPs in CU genomes were manually verified for accuracy.

Analysis of within lineage genetic diversity showed that CU strains had the least nucleotide and amino acid divergence followed by class I and class II strains (Table 3). Analysis of interlineage diversity showed that there was little divergence between CU and class I strains; however, a greater amount of divergence was observed between CU and class II strains (Table 3). Interlineage diversity analysis showed that there was high divergence between class I and class II strains (Table 3).

**Table 3:** Intra- and inter-lineage genetic diversity in CU and GU strains.

**Strains**	**Nucleotide diversity**	**Amino acid diversity**
****Within CU strains****	0.000013	0.000027
****Within class I strains****	0.00044	0.00063
****Within class II strains****	0.0016	0.002
****CU*****versus*****class I****	0.00012	0.00019
****CU*****versus*****class II****	0.0098	0.014
****Class I*****versus*****class II****	0.01	0.015

Diversity analysis was performed using Mega 6.0; the reliability of diversity analysis was tested using 1000 bootstrap replicates.

### Evidence of recombination

While the Phi test showed no evidence of recombination (*P* = 0.44), the likelihood ratio test identified five putative recombination events in CU and GU strains (S6 Table). Removal of recombination regions had no major effect on the overall topology of the phylogenetic tree described in the following section, except for minor differences in bootstrap values and positioning of individual species within class clades (S3 Fig).

### CU strains form a phylogenetic subcluster under class I GU strains

**Fig 3:**
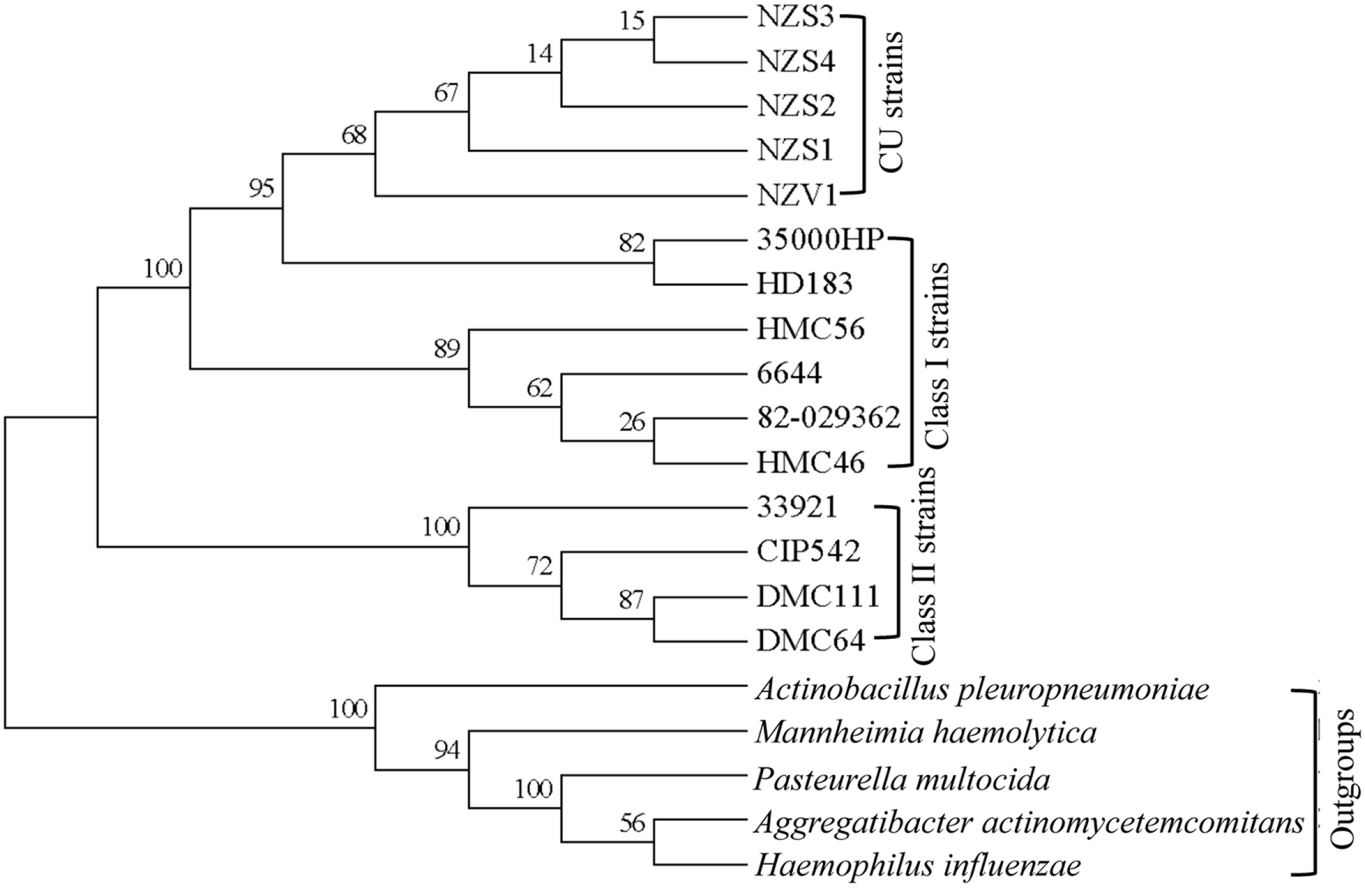
The evolutionary relationships of the CU and GU strains. A rooted phylogenetic tree was inferred by using the Maximum Likelihood method based on the Hasegawa-Kishino-Yano model using *Pasteurellaceae* members as outgroups. All positions containing gaps and missing data were eliminated. The reliability of the tree was tested using 1000 bootstrap replicates and the bootstrap support values are indicated next to the branches in percentage.

In general, all methods showed that class I and class II strains formed two separate phylogenetic clusters and that CU strains formed a subcluster within the class I clade with minor differences in bootstrap values and positioning of individual species within class clades. A rooted tree generated by the Maximum Likelihood method and *Pasteurellaceae* members as outgroups was used as the final tree (Fig 3).

**Fig 4:**
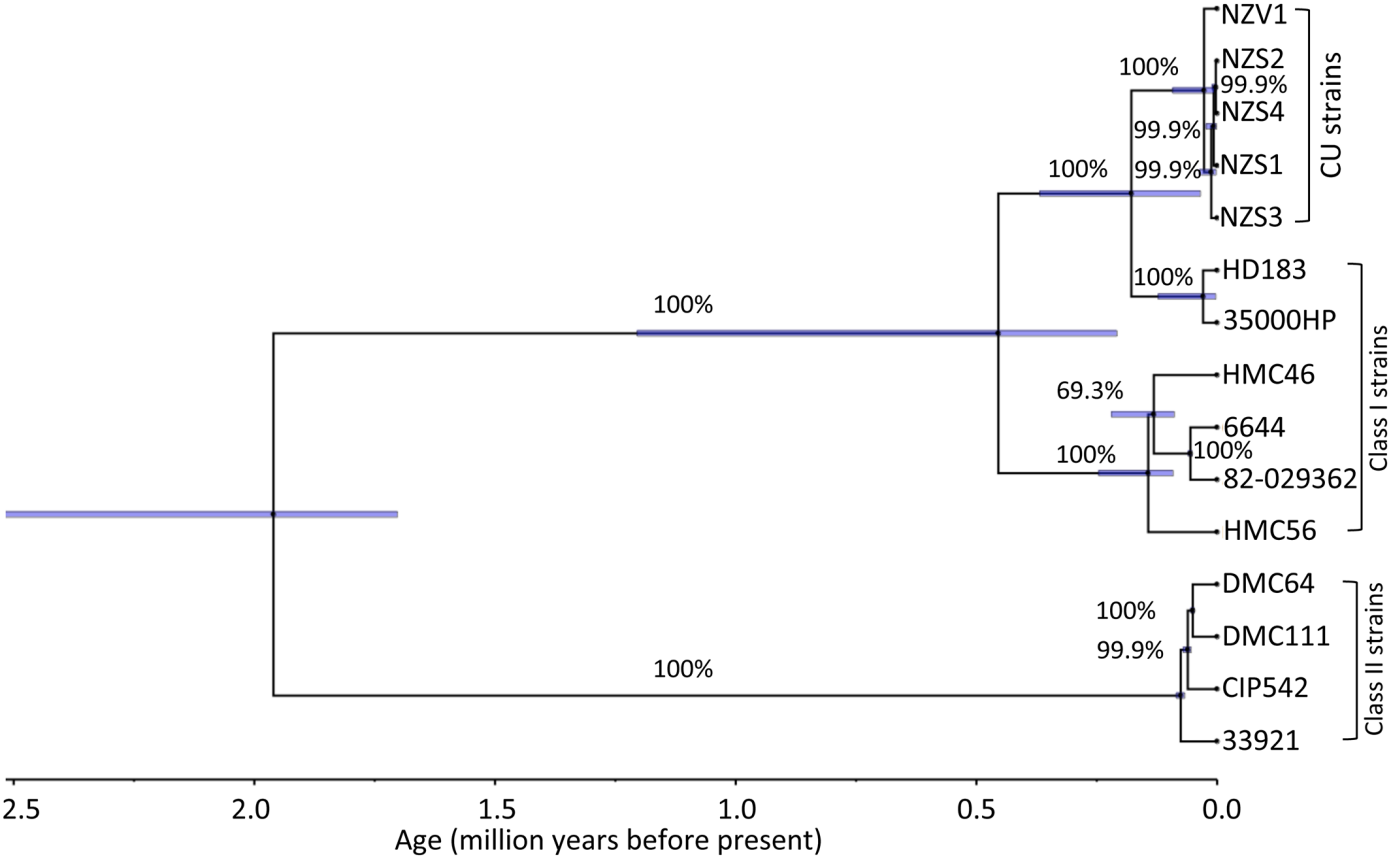
Maximum clade credibility tree from Bayesian molecular clock analysis of the CU and GU strains. The maximum clade credibility tree was generated using TreeAnnotator and visualized using FigTree v1.4.2. Values above the branches indicate posterior probability values in percentage. The blue bars indicate the 95% highest probability density of the inferred node ages. The posterior probability and 95% highest probability density were obtained from four independent runs of 10,000,000 iterations. The values on the time line indicate age in million years before present calculated using a mutation rate of 4.5 × 10^−9^ per site per year.

To determine the approximate time to the MRCA of the CU strains, we performed a molecular clock analysis using the Bayesian method and the mutation rates proposed by Ochman *et al*. for calibration [25, 26]. The divergence time of the CU strains from the MRCA of the class I strains 35000HP and HD183 was estimated as 180,000 years ago (Fig 4). The divergence time of the CU strains, 35000HP, and HD183 from the MRCA of other class I strains was estimated as 450,000 years ago (Fig 4). The divergence time of class I strains from the MRCA of class II strains was estimated as 1.95 mya (Fig 4). Molecular clock analysis also showed that the CU strains began to diversify from each other around 27,000 years ago (Fig 4). Thus, CU strains appear to have recently diverged from class I GU strains.

### The CU and GU strains evolve under negative selection

Pairwise analysis of rates of nonsynonymous (*d*_N_) and synonymous (*d*_S_) substitutions and their variances showed that the Z-test rejected the null hypothesis of neutrality (*d*_N_ = *d*_S_) in favor of the alternative hypothesis of negative selection (*d*_N_ < *d*_S_) (Table 4). The *d*_N_-*d*_S_ value averaging over all sequence pairs was -75.55 (*P* = 0.0000000001). Utilizing the rates of nonsynonymous (*d*_N_) and synonymous (*d*_S_) substitutions, we also calculated the overall mean and pairwise mean *d*_N_/*d*_S_ ratios; the overall mean *d*_N_/*d*_S_ ratio for all genomes was 0.31 and the pairwise mean *d*_N_/*d*_S_ ratios for most comparisons were less than 1 (Table 4). The pairwise mean *d*_N_/*d*_S_ ratio between the CU and GU lineages was 0.35, between CU and class I lineages was 0.38, and between CU and class II lineages was 0.33. Consistent with these analyses, the single likelihood ancestral counting and the fixed effects likelihood analyses identified 141 and 132 negatively selected sites, respectively.

**Table 4:** Evidence of negative selection (*d*_N_ < *d*_S_) in the genomes of CU and GU strains as determined by the codon-based Z test.

	35000HP	NZS1	NZS2	NZS3	NZS4	NZV1	HD183	HMC56	6644	HMC46	82–029362	CIP542	33921	DMC64	DMC111
35000HP		**-3.8** ^**0.47**^ ^**a**^	**-3.6^0.49^**	**-3.6^0.49^**	**-3.8^0.47^**	**-3.9^0.47^**	-2.5^0.41^	**-7.7^0.36^**	**-7.6^0.37^**	**-7.6^0.37^**	**-7.6^0.37^**	**-59.4^0.32^**	**-58.0^0.35^**	**-59.4^0.32^**	**-58.8^0.33^**
NZS1	0.00020		-0.5^0.59^	-0.2^0.88^	-1.2^0.29^	-0.5^0.76^	-3.0^0.55^	**-8.1^0.34^**	**-8.1^0.34^**	**-8.1^0.34^**	**-8.1^0.34^**	**-59.5^0.32^**	**-58.1^0.35^**	**-59.5^0.32^**	**-58.9^0.33^**
NZS2	0.00039	0.58865		2.4^2.6^	-0.7^0.29^	-0.5^0.76^	-2.8^0.57^	**-8.1^0.34^**	**-8.0^0.34^**	**-8.0^0.35^**	**-8.0^0.34^**	**-59.5^0.32^**	**-58.1^0.35^**	**-59.5^0.32^**	**-58.8^0.33^**
NZS3	0.00039	0.87753	0.01575		0.4^1.46^	-0.5^0.76^	-2.8^0.57^	**-8.1^0.34^**	**-8.0^0.34^**	**-8.0^0.35^**	**-8.0^0.34^**	**-59.5^0.32^**	**-58.1^0.35^**	**-59.5^0.32^**	**-58.8^0.33^**
NZS4	0.00024	0.24188	0.49843	0.69932		-0.2^0.88^	-2.9^0.56^	**-8.2^0.33^**	**-8.1^0.34^**	**-8.1^0.34^**	**-8.1^0.34^**	**-59.5^0.32^**	**-58.1^0.35^**	**-59.5^0.32^**	**-58.8^0.33^**
NZV1	0.00016	0.62912	0.62912	0.62912	0.82749		-3.1^0.55^	**-8.1^0.34^**	**-8.1^0.34^**	**-8.1^0.34^**	**-8.1^0.34^**	**-59.5^0.32^**	**-58.1^0.35^**	**-59.5^0.32^**	**-58.9^0.33^**
HD183	0.01571	0.00326	0.00610	0.00610	0.00396	0.00268		**-7.3^0.39^**	**-7.2^0.39^**	**-7.2^0.40^**	**-7.2^0.39^**	**-59.4^0.32^**	**-58.0^0.35^**	**-59.4^0.32^**	**-58.7^0.33^**
HMC56	0.00000	0.00000	0.00000	0.00000	0.00000	0.00000	0.00000		-1.5^0.22^	-1.2^0.50^	-1.4^0.35^	**-59.3^0.32^**	**-57.9^0.35^**	**-59.4^0.32^**	**-58.7^0.33^**
6644	0.00000	0.00000	0.00000	0.00000	0.00000	0.00000	0.00000	0.13280		-0.7^0.65^	-0.8^0.49^	**-59.3^0.32^**	**-57.9^0.35^**	**-59.4^0.32^**	**-58.7^0.33^**
HMC46	0.00000	0.00000	0.00000	0.00000	0.00000	0.00000	0.00000	0.22759	0.48668		-0.4^0.77^	**-59.3^0.32^**	**-57.9^0.35^**	**-59.4^0.32^**	**-58.7^0.33^**
82–029362	0.00000	0.00000	0.00000	0.00000	0.00000	0.00000	0.00000	0.16956	0.40798	0.65881		**-59.3^0.32^**	**-57.9^0.35^**	**-59.4^0.32^**	**-58.7^0.33^**
CIP542	0.00000	0.00000	0.00000	0.00000	0.00000	0.00000	0.00000	0.00000	0.00000	0.00000	0.00000		-0.4^0.98^	-2.2^0.55^	0.4^1.04^
33921	0.00000	0.00000	0.00000	0.00000	0.00000	0.00000	0.00000	0.00000	0.00000	0.00000	0.00000	0.69792		-1.1^0.94^	-0.3^0.99^
DMC64	0.00000	0.00000	0.00000	0.00000	0.00000	0.00000	0.00000	0.00000	0.00000	0.00000	0.00000	0.02797	0.26324		1.3^1.14^
DMC111	0.00000	0.00000	0.00000	0.00000	0.00000	0.00000	0.00000	0.00000	0.00000	0.00000	0.00000	0.70578	0.76022	0.19940	

The test statistic (*d*_N_-*d*_S_) is shown above the diagonal. *d*_N_ and *d*_N_ are the synonymous and nonsynonymous substitutions per site, respectively. The Nei-Gobori method was used to calculate synonymous and non-synonymous substitutions. The probability of rejecting the null hypothesis of strict neutrality (*d*_N_ = *d*_S_) in favor of the alternative hypothesis (*d*_N_<*d*_S_) is shown below the diagonal. *P* values < 0.00047 are considered significant after correcting for multiple comparisons and the significant test statistic (*d*_N_-*d*_S_) values are indicated in bold. The variance was calculated using 1000 bootstrap replicates.

^a^Corresponding pairwise mean *d*_N_/*d*_S_ ratio.

### CU and GU strains evolve under similar selection strength

To determine whether CU strains evolved under different selection strength than GU strains, we performed a TestBranchDNDS analysis. This analysis showed that the strength of selection in CU strains was not significantly different than in GU strains (likelihood ratio difference = 3.8; *P* = 0.58).

### Genes encoding known *H*. *ducreyi* virulence determinants

We determined whether the genomes of CU strains contained the genes that are required for the virulence of strain 35000HP in the human challenge model of infection and whether there were variations in these virulence determinants compared to GU strains [13]. BLAST analysis showed that all the CU and GU strains contained all of the genes known to be required for virulence in the human challenge model (S7 Table). Alignment of amino acid sequences of the virulence determinants showed that the DsrA, LspA1, and LspA2 proteins of the CU strains differed by at least 1 amino acid from class I strains (S7 Table).

### Antimicrobial susceptibility patterns of CU and GU strains

To determine whether CU strains were resistant to clinically relevant antimicrobials, we performed AST using the agar dilution method. The CU strains from Samoa and Vanuatu were AZT susceptible, and had similar susceptibility patterns as the type strains 35000HP and CIP542 (Table 5). With the exception of 82–029362 and 35000HP, all the class I strains were resistant to penicillin (MIC, >256 μg/ml), amoxicillin (MIC, 64–256 μg/ml), and doxycycline (MIC, 8–16 μg/ml) (Table 5). With the exception of CIP542, all class II strains were resistant to amoxicillin and penicillin (MIC, 128–256 μg/ml) (Table 5). The class II strains 33921 and DMC64 were also resistant to doxycycline (MIC, 8–16 μg/ml) (Table 5). All the strains were susceptible to ciprofloxacin, azithromycin, erythromycin and ceftriaxone (Table 5).

**Table 5:** Antimicrobial susceptibility of the CU and GU strains (minimal inhibitory concentrations, μg/ml).

Strain	Class	AMX	PEN	AMC	DOX	CIP	AZT	ERY	CRO
****NZS1****	CU	0.5	0.25	0.5	<0.125	0.015	0.015	0.125	0.008
****NZS2****	CU	1	0.25	1	0.5	0.015	0.03	0.125	0.008
****NZS3****	CU	1	0.25	1	0.5	0.015	0.03	0.125	0.015
****NZS4****	CU	1	0.25	1	0.5	0.015	0.03	0.125	0.015
****NZV1****	CU	1	0.25	1	0.5	0.015	0.03	0.06	0.008
****35000HP****	Class I	1	1	2	0.25	0.008	0.03	0.125	0.03
****82–029362****	Class I	2	1	4	8**^T32^**	0.015	0.015	0.015	0.008
****6644****	Class I	128**^B^**	>256**^B^**	4	16**^TB,T32^**	0.015	0.015	0.015	0.004
****HD183****	Class I	64**^B^**	>256**^B^**	4	8**^T32^**	0.002	0.03	0.125	0.008
****HMC46****	Class I	128**^B^**	>256**^B^**	4	4**^T32^**	0.015	0.03	0.015	0.008
****HMC56****	Class I	>256**^B^**	>256**^B^**	8	4**^T32^**	0.015	0.03	0.015	0.008
****DMC64****	Class II	>256**^B^**	>256**^B^**	4	8**^TM^**	0.008	0.03	0.06	0.004
****DMC111****	Class II	>256**^B^**	>256**^B^**	4	0.25	0.008	0.06	0.06	0.004
****CIP542****	Class II	0.5	0.25	1	0.25	0.004	0.03	0.06	0.004
****33921****	Class II	256**^B^**	>256**^B^**	4	16**^TM^**	0.002	0.06	0.125	<0.002

B- *blaTEM-1B*; TB- *tet(B)*; T32- *tet(32)*; TM- *tet(M)*

AMX- amoxicillin; PEN- penicillin; AMC- amoxicillin and clavulanate; DOX- doxycycline; CIP- ciprofloxacin; AZT- azithromycin; ERY- erythromycin; CRO- ceftriaxone

Consistent with their susceptibility to clinically relevant antimicrobials, the CU strains contained no horizontally acquired genes encoding antimicrobial resistance determinants in their genomes. Consistent with their resistance to penicillin/amoxicillin and doxycycline, the genomes of GU strains contained genes that confer resistance to penicillin/amoxicillin (*blaTEM-1B*) and doxycycline [*tet(B)*, *tet(32)* or *tet(M)*] (Table 5).

## Discussion

*H*. *ducreyi* was previously thought to exclusively cause the sexually transmitted disease chancroid but has emerged as a major cause of the nonsexually transmitted CU in children in yaws-endemic regions of South Pacific islands and equatorial Africa. Here, we performed whole-genome sequencing of a limited number of CU strains and compared them to class I and class II GU strains. Comparative genome analyses showed that the CU strains are remarkably similar to class I strains. Phylogenetic analyses showed that the CU strains evolved from class I GU strains.

Analysis of genome conservation of CU and GU strains showed that CU strains had 98–99% similarity to each other, 94–98% similarity to class I strains, and 81–92% similarity to class II strains. Kunin *et al*., estimated genome conservation within different bacterial taxonomic ranks and found that strains within most bacterial species have a genome conservation of approximately 87% (range, 73–101%) [34]. Thus, the *H*. *ducreyi* genome conservation values are well within the range of those of other bacterial species. Genome conservation analysis also showed that CU strains form a subcluster within class I GU strains and that class II strains form a distinct cluster from class I and CU strains. These findings are in good agreement with the results of the whole-genome phylogenetic analysis as well as with previous multilocus sequence-based phylogenetic analysis [11].

Consistent with the genome conservation data, analysis of whole-genome genetic diversity also showed that there was smallest amount of genetic diversity within the CU strains (*d*_nucleotide_ = 0.000013), little genetic diversity between CU and class I strains (*d*_nucleotide_ = 0.00012), and a greater amount of genetic diversity between CU and class II strains (*d*_nucleotide_ = 0.0098) and class I and class II strains (*d*_nucleotide_ = 0.01). Cejcova *et al*., reported a whole-genome nucleotide diversity of 0.00033 for strains within *T*. *pallidum* subsp. *pallidum* and of 0.00032 for strains within *T*. *pallidum* subsp. *pertenue* [35]. Thus, the *H*. *ducreyi* genetic diversity values for CU and Class I strains are similar to those of the two *Treponema* species that inhabit similar ecological niches as *H*. *ducreyi*, while the diversity values between CU and class II strains and class I and class II strains are higher than those of the two *Treponema* species.

Our study estimated that class I strains diverged from class II strains 1.95 mya and that CU strains diverged from class I strains 0.18 mya. Previous studies estimated that class I strains diverged from class II strains 5 mya and that CU strains diverged from class I strains 0.355 mya [10, 11]. In our study, divergence times were estimated using entire genomes. The previous studies used only 11 *H*. *ducreyi* loci, all of which were selected to contain variant alleles to allow for epidemiological typing. Since a large proportion of genes in the genome do not contain variant alleles, averaging the variance over the entire genome would result in relatively lower divergence times than those estimated in previous studies.

Although CU strains lacked additional genetic material compared to 35000HP, CU strains differed from 35000HP by ~400 SNPs. Nearly 40% of these SNPs were nonsynonymous and 25% were located in noncoding regions of the genome. Previous studies have shown that SNPs can have a profound impact on global gene expression in bacteria [36]. Whether SNPs in the CU strains would result in a different global gene expression pattern than 35000HP requires additional investigation.

A large number of SNPs in the CU strains were located in 21 genes that individually or in combination are required for *H*. *ducreyi* infection in human volunteers. CU strains differed from class I strains by at least one amino acid in 3 virulence determinants, specifically DsrA, LspA1, and LspA2. DsrA is a surface protein and LspA1 and LspA2 are secreted proteins; all three are required for evasion of immune defenses [37, 38]. Thus, the variations of these proteins in CU strains are likely an effect of host immune pressure. Consistent with the fact that class I strains differ from class II strains in several of the known virulence determinants and our finding that CU strains formed a subcluster under class I strains, CU strains differed from class II strains by at least one amino acid in 19 of the 21 virulence determinants [10]. In agreement with a previous study, compared to class I strains, the nucleotide sequences of DsrA and NcaA of class II strains also contained several short rearrangements including deletions and insertions [10].

Analysis of rates of nonsynonymous substitutions (*d*_N_) and synonymous substitutions (*d*_S_) in the CU and GU genomes showed that synonymous substitutions were found at a higher rate than nonsynonymous substitutions with an overall mean *d*_N_/*d*_S_ ratio for all strains of 0.31. Similarly, the pairwise mean *d*_N_/*d*_S_ ratio between CU and GU lineages was 0.35. These data suggest that CU and GU strains evolve under negative selection. Other sexually transmitted bacterial pathogens such as *Neisseria gonorrhoeae* and *Chlamydia trachomatis* also evolve under negative selection, with overall mean *d*_N_/*d*_S_ ratios of 0.3184 and 0.4021, respectively [39, 40]. These findings are in agreement with the neutral theory of molecular evolution, which postulates that selective fixation of neutral mutations by genetic drift is the major determinant behind species divergence [41]. Our data also showed that both CU and GU strains evolve under similar selection strength, which may be due to the similar immunological pressures that these strains encounter in their respective ecological niches of human skin *versus* mucosal surfaces and human skin.

Using SNPs, molecular dating analysis indicates that the CU strains began to diversify from each other ~27,000 years ago. The CU clade is characterized by several shared, derived deletions of defined lengths (synapomorphies), which were most likely inherited from the common ancestor of modern CU strains. Given that these deletions were absent in all the class I GU strains including 35000HP and HD183, we speculate that the Samoan/Vanuatu CU lineage may have existed for at least 27,000 years.

Mitjà and colleagues hypothesized that syndromic management of genital ulcers in the South Pacific may have forced *H*. *ducreyi* into a new niche of cutaneous ulcers in children [6]. Syndromic management of GU in the South Pacific was introduced in 2002, while CU due to *H*. *ducreyi* was first reported in 1989 [6]. The fact that the CU strains diverged from GU strains ~180,000 years ago and from each other ~27,000 years ago supports the idea that cutaneous infection with *H*. *ducreyi* preceded syndromic management of GU. A possible explanation why *H*. *ducreyi* was not recognized as a cause of CU previously is that CU in the South Pacific has traditionally been empirically treated with penicillin [42]. As CU strains are susceptible to penicillin, CU due to *H*. *ducreyi* would have responded to empirical treatment. The current World Health Organization case definition of yaws includes a patient with a chronic atraumatic skin ulcer and seropositivity for *T*. *pallidum* subsp. *pertenue*. In the cross sectional survey in Papua New Guinea, a reasonable proportion of children with detectable *H*. *ducreyi* DNA in ulcers were also seropositive for *T*. *pallidum subsp*. *pertenue* [6] and therefore would be classified as having yaws. This could account for the lack of earlier recognition of *H*. *ducreyi* as a source of CU.

Although penicillin had been the cornerstone of yaws eradication efforts for the last several decades, MDA of AZT is the mainstay of the World Health Organization’s new program for the eradication of yaws [42]. MDA was given to 84% of the villagers who were studied in Papua New Guinea [9]. At 12-months follow-up, MDA reduced the prevalence of CU by 90% [9]. In those who had ulcers at follow-up, there was a significant reduction in the proportion of ulcers with *T*. *pallidum subsp*. *pertenue* DNA [9]. However, the proportion of ulcers containing *H*. *ducreyi* DNA was unchanged relative to the baseline level of 60% [9]. The CU strains from Samoa and Vanuatu were as susceptible to AZT as 35000HP. Whether CU strains from Papua New Guinea are susceptible to AZT is not known. If they are susceptible, their persistence after MDA suggests that CU strains may have a higher level of infectivity than *T*. *pallidum subsp*. *pertenue* or may be present in an environmental reservoir.

Inoculation of the upper arm of human volunteers with the GU strain 35000HP produces an infection that is clinically and histopathologically nearly identical to natural chancroid [13, 43, 44]. Evolutionary analyses showed that CU strains are closely related to 35000HP. Similar to 35000HP, CU strains are capable of infecting nongenital skin. Our data showed that CU strains evolve under selection strength similar to that of GU strains. Due to lack of biopsy specimens, we do not know whether the histopathology of a CU lesion is similar to that of an experimental lesion caused by 35000HP or natural chancroid. Nevertheless, these data suggest that *H*. *ducreyi* likely encounters similar host pressures in the genital and nongenital skin.

Placement of 10^6^ CFU of 35000HP on intact skin does not cause disease in human volunteers; but as few as one bacterium delivered by a puncture wound causes infection [13]. These data raises the possibility that either wounds are required for CU strains to initiate infection or that CU strains possess additional genes that allow them to penetrate intact skin. Our data showed that the CU strains did not contain additional genetic elements, suggesting that CU strains likely use wounds to initiate infection. In Papua New Guinea, up to 7% of children have CU with detectable *H*. *ducreyi* DNA [6]; it is difficult to imagine that wound to wound transmission is responsible for this astoundingly high prevalence. In the Papua New Guinea study, many children infected with *H*. *ducreyi* were seropositive for *T*. *pallidum* subsp. *pertenue* and some ulcers contained both *H*. *ducreyi* and *T*. *pallidum* subsp. *pertenue* DNA [6]. Thus, *T*. *pallidum* subsp. *pertenue* may serve as an instigating pathogen while *H*. *ducreyi* superinfects yaws lesions. Photographs of typical CU lesions show that flies frequently land on ulcers [6]. Thus, it is possible that CU strains are transmitted from person to person by direct contact of wounds with infected lesions, or by vectors such as flies.

In a randomized controlled clinical trial, treatment with 1 gram of AZT prevented experimental infection of adult volunteers with 35000HP for nearly two months [45]. Given that a 2-gram dose of AZT is being used to eradicate yaws, MDA may provide treatment and prophylaxis against CU strains for a similar period of time. These data also suggest that repetition of MDA on a bimonthly basis and/or higher coverage rates may contribute to successful eradication of CU strains from yaws-endemic areas.

By PCR-based testing, 2% of commercial sex workers in a chancroid endemic region are asymptomatically colonized in the cervico-vaginal tract with *H*. *ducreyi* [46]. Whether CU strains asymptomatically colonize the skin of humans living in the tropics is unknown, but colonization would provide a source of bacteria that could enter wounds. As AZT is concentrated intracellularly especially in fibroblasts [45], colonization of the skin surface could allow CU strains to escape AZT treatment.

Our study has several limitations. We only reported draft genomes, and the genetic variation among the strains was not confirmed by PCR and sequencing. Our study involved a small number of GU strains, with limited clinical and epidemiological data. Our analysis only included CU strains that were acquired in Samoa and Vanuatu; our findings should not be extrapolated to CU strains from other regions. All strains used in this study were obtained following culture and storage; their sequences could have been affected by these factors over time. Finally, the CU strains were not compared to contemporaneous GU strains from the same or other regions; to our knowledge and due to syndromic management, few such GU strains exist.

This was the first study using comparative genomics to examine a small number of cultured *H*. *ducreyi* strains isolated from CU and GU. Our findings show that CU strains are derivatives of class I GU strains whose lineage may be 27,000 years old. Further studies are needed to determine the phylogeny of CU strains from other endemic areas, such as Papua New Guinea, Ghana, and the Solomon Islands, and to examine strains that persist after MDA of azithromycin. Flies and nonhuman primates are thought to serve as reservoirs for *T*. *pallidum subsp*. *pertenue* [6, 47]; it would be interesting to determine whether they serve as reservoirs for CU strains or whether humans who reside in endemic areas are colonized with *H*. *ducreyi*.

## Supporting Information

S1 Fig Flow chart of comparative genome analysis of CU and GU strains. (PDF)

S2 Fig Multiple genome alignment of the CU and GU strains generated by ProgressiveMauve. (PDF)

S3 Fig The phylogenetic relationship of CU and GU strains with (A) and without (B) putative recombination regions. (PDF)

S1 Table Clinical features of the CU strains used in the present study. (PDF)

S2 Table Putative deletions in the genomes of CU strains relative to 35000HP. (PDF)

S3 Table Putative deletions in the genomes of class I strains relative to 35000HP. (PDF)

S4 Table Putative deletions in the genomes of class II strains relative to 35000HP. (PDF)

S5 Table Additional genes/DNA sequences present in the GU strains relative to 35000HP. (PDF)

S6 Table Putative recombination points in the genomes of CU and GU strains as determined by the likelihood ratio test (LRT). (PDF)

S7 Table Alignment of CU and GU strains’ virulence determinants required for infection in the human challenge model of *H*. *ducreyi* infection. (PDF)
