# Supplementary figures and images for: Haemophilus ducreyi Cutaneous Ulcer Strains Are Nearly Identical to Class I Genital Ulcer Strains
Source: PLoS Negl Trop Dis. 2015 Jul 6;9(7):e0003918. doi: 10.1371/journal.pntd.0003918 (PMC4492979; doi:10.1371/journal.pntd.0003918)

## S1 Figure

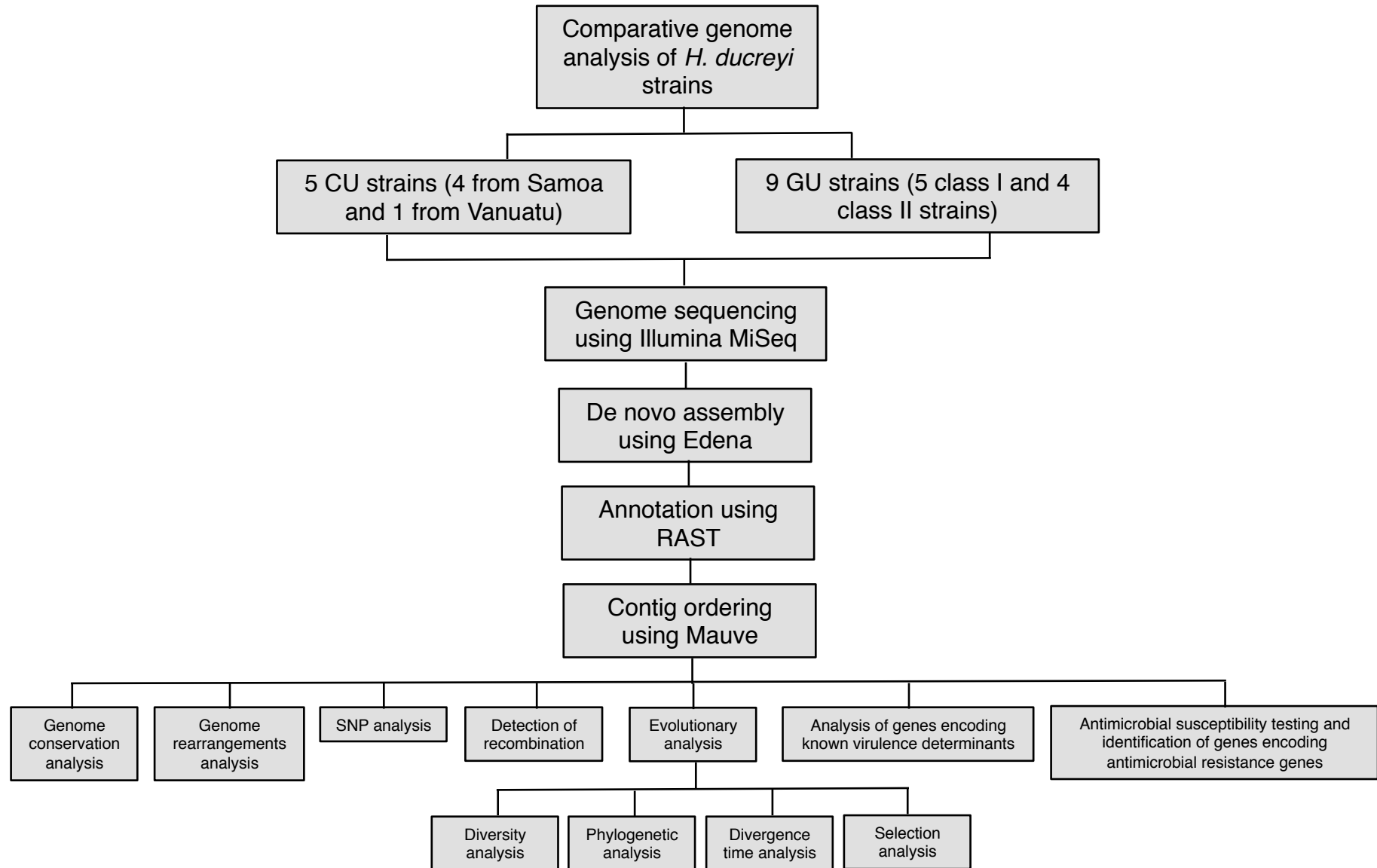

Supplement: S1 Fig — (PDF) [file pntd.0003918.s001.pdf]

## S2 Figure

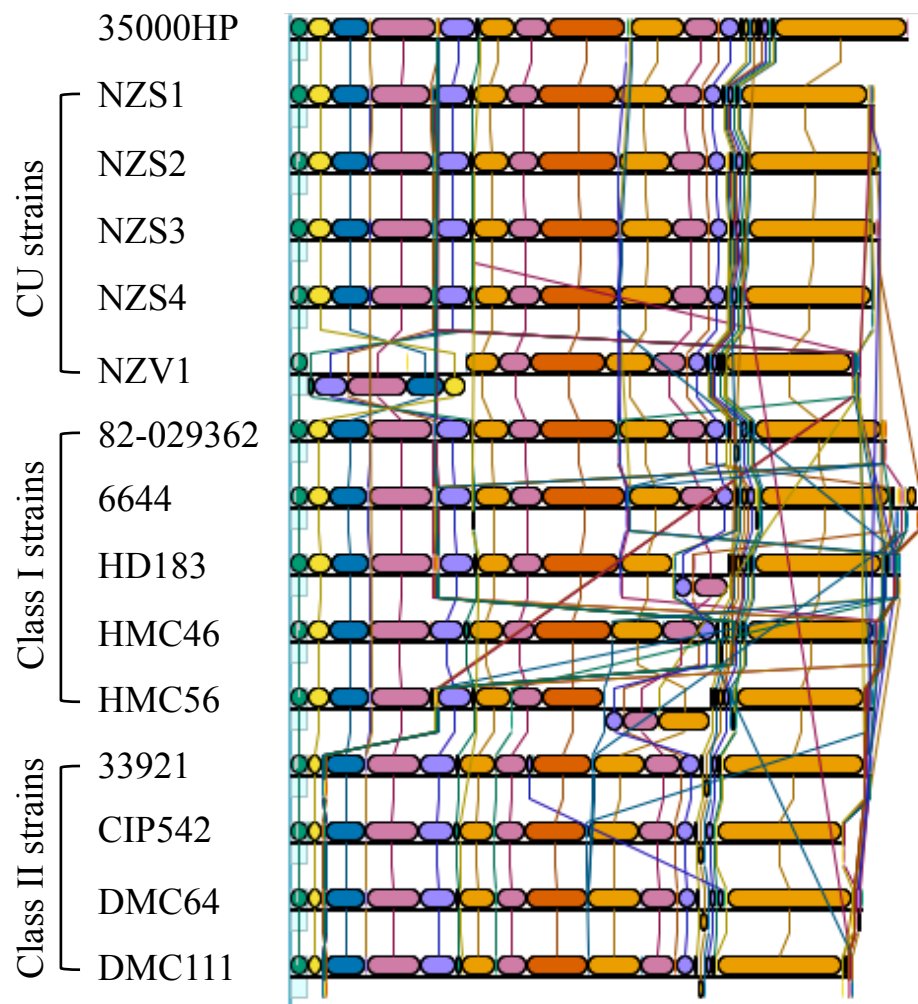

Supplement: S2 Fig — (PDF) [file pntd.0003918.s002.pdf]

S3 Figure

A

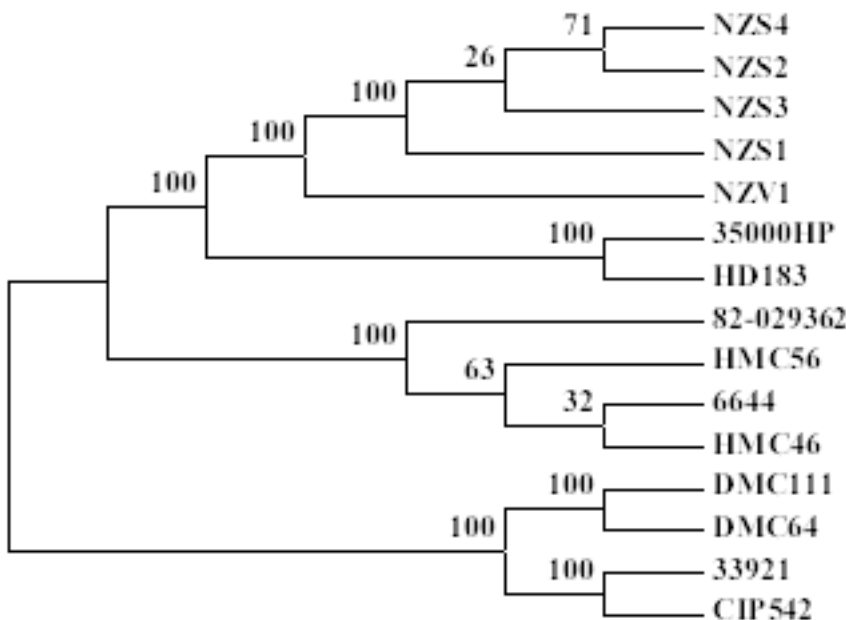

B

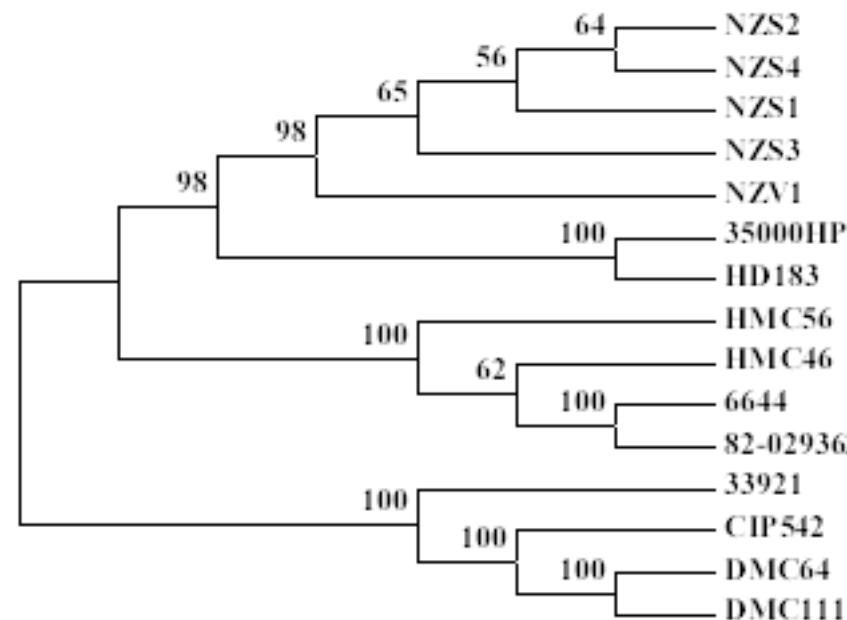

Supplement: S3 Fig — (PDF) [file pntd.0003918.s003.pdf]
