# Supplementary material for: Haemophilus ducreyi Cutaneous Ulcer Strains Are Nearly Identical to Class I Genital Ulcer Strains
Source: PLoS Negl Trop Dis. 2015 Jul 6;9(7):e0003918. doi: 10.1371/journal.pntd.0003918 (PMC4492979; doi:10.1371/journal.pntd.0003918)
Supplement: S1 Table — (PDF) [file pntd.0003918.s004.pdf]

TABLE S1. Clinical features of the CU strains used in the present study.

Strain no.	Year	Age (years)	Gender	Origin	Site	Microbiology	Treatment	Outcome	Reference
NZS1	February 2005	9	Female	Samoa	Both knees	*H. ducreyi*, cultured, and confirmed by 16S rDNA sequencing	Flucloxacillin with minimal improvement followed by benzyl penicillin	Healed	(1)
NZS2	February 2006	6	Female	Samoa	Right lower calf	*H. ducreyi*, cultured, and confirmed by 16S rDNA sequencing	Flucloxacillin with minimal improvement followed by azithromycin	Healed	(1)
NZS3	June 2006	5	Female	Samoa	Right ankle	*H. ducreyi*, cultured, and confirmed by 16S rDNA sequencing	Flucloxacillin and erythromycin with minimal improvement followed by benzyl penicillin	Healed	(1)
NZS4	February 2007	15	Female	Samoa	Left lower thigh	*H. ducreyi*, cultured, and confirmed by 16S rDNA sequencing	Samoan oil followed by flucloxacillin	Healed	(2)
NZV1	May 2014	2	Male	Vanuatu	Not known	*H. ducreyi*, cultured, and confirmed by 16S rDNA sequencing	No response to cephalexin; final treatment unknown	Healed	This study

1. **Ussher JE, Wilson E, Campanella S, Taylor SL, Roberts SA.** 2007. *Haemophilus ducreyi* causing chronic skin ulceration in children visiting samoa. Clinical Infectious Diseases 44: 85.
2. **Gaston J, Roberts, SA, Humphreys, TL.** 2015. Molecular phylogenetic analysis of non-sexually transmitted strains of *Haemophilus ducreyi*. PLoS One. 10(3): e0118613.
