# Supplementary material for: Haemophilus ducreyi Cutaneous Ulcer Strains Are Nearly Identical to Class I Genital Ulcer Strains
Source: PLoS Negl Trop Dis. 2015 Jul 6;9(7):e0003918. doi: 10.1371/journal.pntd.0003918 (PMC4492979; doi:10.1371/journal.pntd.0003918)
Supplement: S2 Table — (PDF) [file pntd.0003918.s005.pdf]

TABLE S2. Putative deletions in the genomes of CU strains relative to 35000HP.

**Feature**	**Reference position**	**Length (bp)**[a]
Intergenic	213,567	30
HD1034	825,958	388
HD1278	1,040,857	69
HD1528-HD1565	1,265,042	20,193
HD1715	1,429,444	196
insB & HD1973	1,645,354	767

[a]These deletions were present in all the 5 cutaneous ulcer strains sequenced in this study.
