# Supplementary material for: Haemophilus ducreyi Cutaneous Ulcer Strains Are Nearly Identical to Class I Genital Ulcer Strains
Source: PLoS Negl Trop Dis. 2015 Jul 6;9(7):e0003918. doi: 10.1371/journal.pntd.0003918 (PMC4492979; doi:10.1371/journal.pntd.0003918)
Supplement: S3 Table — (PDF) [file pntd.0003918.s006.pdf]

TABLE S3. Putative deletions in the genomes of class I strains relative to 35000HP.

Feature	Reference position	Length (bp)	82-029362	6644	HD183	HMC46	HMC56
HD0094 [pseudo]-HD0097	73898	3156	Δ	Δ	WT	Δ	Δ
HD0113 & HD0114	82733	129	WT	WT	Δ	WT	WT
HD0192	136845	111	Δ	Δ	WT	Δ	Δ
nagZ	412854	79	Δ	Δ	WT	Δ	Δ
Intergenic & HD0778	610591	85	Δ	Δ	WT	Δ	Δ
HD0897-Lys-1	719122	49457	WT	WT	WT	WT	Δ
glpF & *glpK*	914594	1424	WT	WT	Δ	WT	WT
glpK	915836	322	WT	WT	WT	WT	Δ
glpQ [pseudo]	921511	322	Δ	Δ	WT	Δ	Δ
ompP2A &/or *ompP2B*	1175494	1545	WT	WT	WT	WT	Δ
InsB & HD1973	1645353	768	Δ	Δ	Δ	Δ	Δ

[Δ]Deletion.
[WT]No deletion.
