# Supplementary material for: Haemophilus ducreyi Cutaneous Ulcer Strains Are Nearly Identical to Class I Genital Ulcer Strains
Source: PLoS Negl Trop Dis. 2015 Jul 6;9(7):e0003918. doi: 10.1371/journal.pntd.0003918 (PMC4492979; doi:10.1371/journal.pntd.0003918)
Supplement: S4 Table — (PDF) [file pntd.0003918.s007.pdf]

TABLE S4. Putative deletions in the genomes of class II strains relative to 35000HP.

**Feature**	**Reference position**	**Length**[a]
HD0087-HD0161	72,267	37,342
HD0192	136,855	111
Intergenic	162,237	62
HD0286-HD0294	221,125	5,864
HD0343	267,589	40
HD0356	276,758	106
HD0478-HD0495	377,197	34,936
HD0868	693,734	288
sprT	699,284	233
HD0897-Lys-1	719,122	49,457
HD1013-macB	804,551	7,532
HD1094	872,236	1,342
HD1096	874,772	99
Intergenic	911,217	48
Intergenic	988,434	45
HD1288-HD1289	1,052,027	529
hhdA	1,086,032	169
torY-torZ	1,140,852	814
HD1522-*gam*	1,263,813	615
HD1526	1,264,727	169
HD1528-HD1529	1,266,081	240
HD1532-HD1533	1,268,824	956
HD1540-HD1546	1,270,497	2,692
HD1554-HD1555	1,276,455	98
HD1567-HD1569	1,286,403	680
HD1609-HD1618	1,330,812	5,638
Intergenic	1,358,706	85
HD1687-HD1698	1,405,645	9,350
nanE	1,554,824	451
HD1920	1,614,695	585
insB-HD1973	1,645,369	752

[a]These deletions were present in all the four class II strains sequenced in this study.
