# Supplementary material for: Haemophilus ducreyi Cutaneous Ulcer Strains Are Nearly Identical to Class I Genital Ulcer Strains
Source: PLoS Negl Trop Dis. 2015 Jul 6;9(7):e0003918. doi: 10.1371/journal.pntd.0003918 (PMC4492979; doi:10.1371/journal.pntd.0003918)
Supplement: S5 Table — (PDF) [file pntd.0003918.s008.pdf]

TABLE S5. Additional genes/DNA sequences present in the GU strains relative to 35000HP.

**Size (kb)**	**Homology**	**Identity (%)**	**Strains**						
			**6644**	**HD183**	**HMC46**	**HMC56**	**33921**	**DMC64**	**DMC111**
5	*Escherichia fergusoni* ATCC 35469 plasmid pEFER	99	+	+	+	+	+	+	+
26	GI-like type IV secretion system of *Haemophilus parainfluenzae* T3T1	97	+	-	-	-	-	-	-
2	Mercuric oxidase and mercuric resistance operon regulatory protein of *Streptococcus agalactiae*	99	+	-	-	-	-	-	-
2.6	Two hypothetical proteins in *E. coli* O104:H4 str. C227-11	99-100	-	+	-	-	+	-	+
1.3	*Yersinia ruckeri* YR71 plasmid pYR1	100	-	+	-	-	+	+	+
1.3	*Corynebacterium aurimucosum* transposase	94-96	-	+	-	-	-	-	-
7	*Acidaminococcus intestini* RyC-MR95	100	-	-	+	-	-	-	-

+Present
-Absent
