# Supplementary material for: Haemophilus ducreyi Cutaneous Ulcer Strains Are Nearly Identical to Class I Genital Ulcer Strains
Source: PLoS Negl Trop Dis. 2015 Jul 6;9(7):e0003918. doi: 10.1371/journal.pntd.0003918 (PMC4492979; doi:10.1371/journal.pntd.0003918)
Supplement: S6 Table — (PDF) [file pntd.0003918.s009.pdf]

TABLE S6. Putative recombination points in the genomes of CU and GU strains as determined by the likelihood ratio test (LRT).

Gene	Description	Nucleotide position	LRT score
argH	Arginosuccinate lyase	664720	54.7
		664730	60.0
		664740	49.8
		664750	49.8
		664760	49.8
		664770	49.8
		664780	49.8
		664790	49.8
		664800	49.8
		664810	49.8
lon	ATP-dependent protease LA	741540	79.2
		741550	79.2
		741560	79.2
		741570	88.4
		741580	88.4
		741590	88.4
		741600	88.4
HD0637	Hypothetical protein	1400630	27.7
flp3	*flp* operon protein Flp3	1400800	19.4
		1400810	21.4
		1400820	22.5
		1400830	25.4
		1400840	27.5
		1400850	30.7
		1400860	32.3
		1400870	32.3
HD1070	Hypothetical protein	1424410	36.3
		1424420	36.7
		1424430	47.1
		1424440	47.5
		1424450	47.9
		1424460	47.9
		1424470	48.0
		1424480	48.4
		1424490	48.8
		1424500	49.1
		1424510	49.2
		1424520	49.2
