# Supplementary material for: Haemophilus ducreyi Cutaneous Ulcer Strains Are Nearly Identical to Class I Genital Ulcer Strains
Source: PLoS Negl Trop Dis. 2015 Jul 6;9(7):e0003918. doi: 10.1371/journal.pntd.0003918 (PMC4492979; doi:10.1371/journal.pntd.0003918)
Supplement: S7 Table — (PDF) [file pntd.0003918.s010.pdf]

TABLE S7. Alignment of CU and GU strains' virulence determinants required for virulence in the human challenge model of *H. ducreyi* infection.

# 1) CpxA (HD1470)

```
35000HP      MPKFLKKIKSVRNYLAYQLFAYFGLTFAIMLAITLAIPNFDARSFSRLEKGEHEFFIQES
33921        MPKFLKKIKSVRNYLAYQLFAYFGLTFAIMLAITLAIPNFDARSFSRLEKGEHEFFIQES
CIP542       MPKFLKKIKSVRNYLAYQLFAYFGLTFAIMLAITLAIPNFDARSFSRLEKGEHEFFIQES
DMC64        MPKFLKKIKSVRNYLAYQLFAYFGLTFAIMLAITLAIPNFDARSFSRLEKGEHEFFIQES
DMC111       MPKFLKKIKSVRNYLAYQLFAYFGLTFAIMLAITLAIPNFDARSFSRLEKGEHEFFIQES
             ************************************************************

35000HP      RQTELQYNLDEIFERRLSVATQNGFNIILFDPKTRIFVGAGADSNRIQSFQVFLYRAQTP
33921        RQTELQYNLDEIFERRLSVATQSGFNIILFDPKTRIFVGAGADSNRIQSFQVFLYRAQTP
CIP542       RQTELQYNLDEIFERRLSVATQSGFNIILFDPKTRIFVGAGADSNRIQSFQVFLYRAQTP
DMC64        RQTELQYNLDEIFERRLSVATQSGFNIILFDPKTRIFVGAGADSNRIQSFQVFLYRAQTP
DMC111       RQTELQYNLDEIFERRLSVATQSGFNIILFDPKTRIFVGAGADSNRIQSFQVFLYRAQTP
             **********************.*************************************

35000HP      LEPLQRRFGSLEISGPFLVKSNKREYMQYFAQIVDPQEEFFNRIFDSPWLMLMIVLIVSV
33921        LEPLQRRFGSLEISGPFLVKSNKREYMQYFAQIVDPQEEFFNRIFDSPWLMLMIVLIVSV
CIP542       LEPLQRRFGSLEISGPFLVKSNKREYMQYFAQIVDPQEEFFNRIFDSPWLMLMIVLIVSV
DMC64        LEPLQRRFGSLEISGPFLVKSNKREYMQYFAQIVDPQEEFFNRIFDSPWLMLMIVLIVSV
DMC111       LEPLQRRFGSLEISGPFLVKSNKREYMQYFAQIVDPQEEFFNRIFDSPWLMLMIVLIVSV
             ************************************************************

35000HP      PILLWLSWKIARPVKELRICANAVATGNLAINPKLETEGIHEFREVGRSFNQMITSLQEL
33921        PILLWLSWKIARPVKELRICANAVATGNLAINPKLETEGIHEFREVGRSFNQMITSLQEL
CIP542       PILLWLSWKIARPVKELRICANAVATGNLAINPKLETEGIHEFREVGRSFNQMITSLQEL
DMC64        PILLWLSWKIARPVKELRICANAVATGNLAINPKLETEGIHEFREVGRSFNQMITSLQEL
DMC111       PILLWLSWKIARPVKELRICANAVATGNLAINPKLETEGIHEFREVGRSFNQMITSLQEL
             ************************************************************

35000HP      TEYQQRLLSDISHELKTPLARLQLATALIRRRNGDSAELTRIDNQIMKLDTMVHDLLSLS
33921        TEYQQRLLSDISHELKTPLARLQLATALIRRRNGDSAELTRIDNQIMKLDTMVHDLLSLS
CIP542       TEYQQRLLSDISHELKTPLARLQLATALIRRRNGDSAELTRIDNQIMKLDTMVHDLLSLS
DMC64        TEYQQRLLSDISHELKTPLARLQLATALIRRRNGDSAELTRIDNQIMKLDTMVHDLLSLS
DMC111       TEYQQRLLSDISHELKTPLARLQLATALIRRRNGDSAELTRIDNQIMKLDTMVHDLLSLS
             ************************************************************

35000HP      RQQINQHLMREVFSINKIWDDILEDAKFEAEQNQIDLFIEQRIDNVEGYFINGNEIILAS
33921        RQQINQHLMREVFSINKIWDDILEDAKFEAEQNQIDLFIEQRIDNVEGYFINGNEIILAS
CIP542       RQQINQHLMREVFSINKIWDDILEDAKFEAEQNQIDLFIEQRIDNVEGYFINGNEIILAS
DMC64        RQQINQHLMREVFSINKIWDDILEDAKFEAEQNQIDLFIEQRIDNVEGYFINGNEIILAS
DMC111       RQQINQHLMREVFSINKIWDDILEDAKFEAEQNQIDLFIEQRIDNVEGYFINGNEIILAS
             ************************************************************

35000HP      ALENLIRNAQKYAKQSITVLIYIDDKELVMSVDDDGEGVPESEYKQIFRPFYRVGEARDR
33921        ALENLIRNAQKYAKQSITVLIYIDDKELVMSVDDDGEGVPESEYKQIFRPFYRVGEARDR
CIP542       ALENLIRNAQKYAKQSITVLIYIDDKELVMSVDDDGEGVPESEYKQIFRPFYRVGEARDR
DMC64        ALENLIRNAQKYAKQSITVLIYIDDKELVMSVDDDGEGVPESEYKQIFRPFYRVGEARDR
DMC111       ALENLIRNAQKYAKQSITVLIYIDDKELVMSVDDDGEGVPESEYKQIFRPFYRVGEARDR
             ************************************************************

35000HP      QSGGTGLGLAIVANAAQQHKGRVEAMKSILGGLRVETRLPLWLE*
33921        QSGGTGLGLAIVANAAQQHKGRVEAMKSILGGLRVEIRLPLWLE*
CIP542       QSGGTGLGLAIVANAAQQHKGRVEAMKSILGGLRVEIRLPLWLE*
DMC64        QSGGTGLGLAIVANAAQQHKGRVEAMKSILGGLRVEIRLPLWLE*
DMC111       QSGGTGLGLAIVANAAQQHKGRVEAMKSILGGLRVEIRLPLWLE*
             ************************************ ********
```

**Note:** The CpxA sequence of NZS1, NZS2, NZS3, NZS4, NZV1, 82-029362, 6644, HD183, HMC46, and HMC56 were identical to that of 35000HP.

# 2) CsrA (HD1430)

The CsrA sequence of all the class I and class II strains were identical to 35000HP.

# 3) DksA (HD0603)

```
35000HP      MVQVATTTSLGLLALAGVTPYQPKKDEEYMNDAQKEHFRKILRAWHVQIMEEAERTKNQM
33921        MVQVATTTSLGLLALAGVTPYQPKKDEEYMNDAQKEHFRKILRAWHVQIMEEAERTKNQM
CIP542       MVQVATTTSLGLLALAGVTPYQPKKDEEYMNDAQKEHFRKILRAWHVQIMEEAERTKNQM
DMC64        MVQVATTTSLGLLALAGVTPYQPKKDEEYMNDAQKEHFRKILRAWHVQIMEEAERTKNQM
DMC111       MVQVATTTSLGLLALAGVTPYQPKKDEEYMNDAQKEHFRKILRAWHVQIMEEAERTKNQM
             ************************************************************

35000HP      QEEVANFADPADRATQEEEFNLELRNRDRERKLLKKIEQTLNSISEDDYGYCQTCGVEIG
33921        QEEVANFADPADRATQEEEFNLELRNRDRERKLLKKIEQTLNSISEDDYGYCQTCGVEIG
CIP542       QEEVANFADPADRATQEEEFNLELRNRDRERKLLKKIEQTLNSISEDDYGYCQTCGVEIG
DMC64        QEEVANFADPADRATQEEEFNLELRNRDRERKLLKKIEQTLNSISEDDYGYCQTCGVEIG
DMC111       QEEVANFADPADRATQEEEFNLELRNRDRERKLLKKIEQTLNSISEDDYGYCQTCGVEIG
             ************************************************************

35000HP      LRRLEARPTADMCIDCKTLAEIREKQMGL*
33921        LRRLEARPTADMCIDCKTLAEIREKQMGL*
CIP542       LRRLEARPTADMCIDCKTLAEIREKQMGL*
DMC64        LRRLEARPTADMCIDCKTLAEIREKQMGL*
DMC111       LRRLEARPTADMCIDCKTLAEIREKQMGL*
             ******************************
```

**Note:** The DksA sequence of NZS1, NZS2, NZS3, NZS4, NZV1, 82-029362, 6644, HD183, HMC46, and HMC56 were identical to that of 35000HP.

# 4) DltA (HD0746)

```
35000HP      MKTLGLFSIIGLLSGCVYAPPAVDHPIEDTVVIQSEHHHTIYHESDKYDRDKYDRNDRYP
33921        MKTLGLFSIIGLLSGCVYAPPAVDNPIEDTVVIQSEHHHMIYHESDKYDRDKYDRNDRYP
CIP542       MKTLGLFSIIGLLSGCVYAPPAVDNPIEDTVVIQSEHHHMIYHESDKYDRDKYDRNDRYP
DMC64        MKTLGLFSIIGLLSGCVYAPPAVDNPIEDTVVIQSEHHHMIYHESDKYDRDKYDRNDRYP
DMC111       MKTLGLFSIIGLLSGCVYAPPAVDNPIEDTVVIQSEHHHMIYHESDKYDRDKYDRNDRYP
             ************************.************** ********************

35000HP      HYRYTGEIRTYWGECLDQSRSNYKGIISYRCHGGDNQRFTFYRDSIRVNGQCLDVGSENK
33921        HYHYTGEIRTYWGKCLDQSRSNYKGIISYRCHGGDNQRFTFYRDSIRVNGQCLDVGSENK
CIP542       HYHYTGEIRTYWGKCLDQSRSNYKGIISYRCHGGDNQRFTFYRDSIRVNGQCLDVGSENK
DMC64        HYHYTGEIRTYWGKCLDQSRSNYKGIISYRCHGGDNQRFTFYRDSIRVNGQCLDVGSENK
DMC111       HYHYTGEIRTYWGKCLDQSRSNYKGIISYRCHGGDNQRFTFYRDSIRVNGQCLDVGSENK
             **:**********:**********************************************

35000HP      FDGARIIAYRCHGGKNQRWFRQGHQIRSEMNGKCLEVGRDRNKLTLQQCDGSRSQQFFY*
33921        FDGARIIAYRCHGGKNQRWFRQGHQIRSEMNGKCLEVGRDRNKLTLQQCDGSRSQQFFY*
CIP542       FDGARIIAYRCHGGKNQRWFRQGHQIRSEMNGKCLEVGRDRNKLTLQQCDGSRSQQFFY*
DMC64        FDGARIIAYRCHGGKNQRWFRQGHQIRSEMNGKCLEVGRDRNKLTLQQCDGSRSQQFFY*
DMC111       FDGARIIAYRCHGGKNQRWFRQGHQIRSEMNGKCLEVGRDRNKLTLQQCDGSRSQQFFY*
             ************************************************************
```

**Note:** The DltA sequence of NZS1, NZS2, NZS3, NZS4, NZV1, 82-029362, 6644, HD183, HMC46, and HMC56 were identical to that of 35000HP.

# 5) DsrA (HD0769)

```
35000HP        ------MKIKCLVAVVGLACSTITTMAQQPPKFAGVSSLYSYEYDYGKGKWTWSNEGGFD
NZS1           ------MKIKCLVAVVGLACSTITTMAQQPPKFAGVSSLYSYEYDYGKGKWTWSNEDGFD
NZS2           ------MKIKCLVAVVGLACSTITTMAQQPPKFAGVSSLYSYEYDYGKGKWTWSNEDGFD
NZS3           ------MKIKCLVAVVGLACSTITTMAQQPPKFAGVSSLYSYEYDYGKGKWTWSNEDGFD
NZS4           ------MKIKCLVAVVGLACSTITTMAQQPPKFAGVSSLYSYEYDYGKGKWTWSNEDGFD
NZV1           ------MKIKCLVAVVGLACSTITTMAQQPPKFAGVSSLYSYEYDYGKGKWTWSNEDGFD
82-029362      ------MKIKCLVAVVGLACSTITTMAQQPPKFAGVSSLDSYEYDYGKGKWTWSEKDGFD
6644           ------MKIKCLVAVVGLACSTITTMAQQPPKFAGVSSLDSYEYDYGKGKWTWSEKDGFD
HD183          ------MKIKCLVAVVGLACSTITTMAQQPPKFAGVSSLYSYEYDYGKGKWTWSNEGGFD
HMC46          ------MKIKCLVAVVGLACSTITTMAQQPPKFAGVSSLDSYEYDYGKGKWTWSEKDGFD
HMC56          ------MKIKCLVAVVGLACSTITTMAQQPPKFAGVSSLDSYEYDYGKGKWTWSEKDGFD
33921          MKMKMKMKMKLTALMVGLVASNGSLSAQMQPQNFDLGRIKIGDVSFDVDAFIKRV-DELD
CIP542         MKMKMKMKMKLTALMVGLVASNGSLSAQMQPQNFDLGRIKIGDVSFDVDAFIKRV-DELD
DMC64          MKMKMKMKMKLTALMVGLVASNGSLSAQMQPQNFDLGRIKIGDVSFDVDAFIKRV-DELD
DMC111         MKMKMKMKMKLTALMVGLVASNGSLSAQMQPQNFDLGRIKIGDVSFDVDAFIKRV-DELD
                     **:*  . :***..*. :  **  *:   :. :   : .:    :       :*
```

```
35000HP        -IKVPGIKMKPKEWISKQATYLELQHYMPYTPVLVTYA-PGVSPSPILLYPMSDPDQLGI
NZS1           -IKAPRIKMKPKEWISKQVTYLKLQHYMPYTPVLVTSDVPP--PSSILLYPMSDPDQLGI
NZS2           -IKAPRIKMKPKEWISKQVTYLKLQHYMPYTPVLVTSDVPPPPPSSILLYPMSDPDQLGI
NZS3           -IKAPRIKMKPKEWISKQVTYLKLQHYMPYTPVLVTSDVPPPPPSSILLYPMSDPDQLGI
NZS4           -IKAPRIKMKPKEWISKQVTYLKLQHYMPYTPVLVTSDVPPP-PSSILLYPMSDPDQLGI
NZV1           -IKAPRIKMKPKEWISKQVTYLKLQHYMPYTPVLVTSDVPPP-PSSILLYPMSDPDQLGI
82-029362      -IKAPGIKMKPKKWISRQATYLGLQHYMPYTPVLVTYAS--AEPNTVLLYPMPDPDQLGI
6644           -IKAPGIKMKPKKWISRQATYLGLQHYMPYTPVLVTYAS--AEPNTVLLYPMPDPDQLGI
HD183          -IKVPGIKMKPKEWISKQATYLELQHYMPYTPVLVTSA-PDVPPSSILLYPMSDPDQLGI
HMC46          -IKAPGIKMKPKKWISRQATYLGLQHYMPYTPVLVTYAS--AEPNTVLLYPMPDPDQLGI
HMC56          -IKAPGIKMKPKKWISRQATYLGLQHYMPYTPVLVTYAS--AEPNTVLLYPMPDPDQLGI
33921          KNIRDGIGLGDEDFKRKYYSYVDHE---------LGS-----MIGSPFIYKEKNPLGNGI
CIP542         KNIRDGIGLGDEDFKRKYYSYVDHE---------LGS-----MIGSPFIYKEKNPLGNGI
DMC64          KNIRDGIGLGDEDFKRKYYSYVDHE---------LGS-----MIGSPFIYKEKNPLGNGI
DMC111         KNIRDGIGLGDEDFKRKYYSYVDHE---------LGS-----MIGSPFIYKEKNPLGNGI
                     * :  :.:  :  :*:  :         :            ::*   :*   **

35000HP        NRQQL---KLNLYSYFN------------------------DLRHDFKLKVLDARI----
NZS1           NRQQL---KLNLYSYFN------------------------DLRHDFKLNVLDARI----
NZS2           NRQQL---KLNLYSYFN------------------------DLRHDFKLNVLDARI----
NZS3           NRQQL---KLNLYSYFN------------------------DLRHDFKLNVLDARI----
NZS4           NRQQL---KLNLYSYFN------------------------DLRHDFKLNVLDARI----
NZV1           NRQQL---KLNLYSYFN------------------------DLRHDFKLNVLDARI----
82-029362      NRQQL---KLNLYSYFN------------------------DLRHGFKLNVLDARI----
6644           NRQQL---KLNLYSYFN------------------------DLRHGFKLNVLDARI----
HD183          NRQQL---KLNLYSYFN------------------------DLRHDFKLKVLDARI----
HMC46          NRQQL---KLNLYSYFN------------------------DLRHGFKLNVLDARI----
HMC56          NRQQL---KLNLYSYFN------------------------DLRHGFKLNVLDARI----
33921          ARGLGYSGKLTMPRYIRGPLLRRSVIRGYLLGRVIGRQAHVQAKNRLNMELLQSRLMSAS
CIP542         ARGLGYSGKLTMPRYIRGPLLRRSVIRGYLLGRVIGRQAHVQAKNRLNMELLQSRLMSAS
DMC64          ARGLGYSGKLTMPRYIRGPLLRLSVIRGYLLGRVIGRQAHVQAKNRLNMELLQSRLMSAS
DMC111         ARGLGYSGKLTMPRYIRGPLLRLSVIRGYLLGRVIGRQAHVQAKNRLNMELLQSRLMSAS
                *      **.:  *:.                        : :. :::::*::*:

35000HP        SKNKQNIDTISKYLLELGTYLDDSYRMMEQNTH--------------NINKLSKELQTGL
NZS1           SKNKQNIDTISKHLLELGTYLDGSYRMMEQNTHNINKN-------THNINKLSKELQTGL
NZS2           SKNKQNIDTISKHLLELGTYLDGSYRMMEQNTHNINKN-------THNINKLSKELQTGL
NZS3           SKNKQNIDTISKHLLELGTYLDGSYRMMEQNTHNINKN-------THNINKLSKELQTGL
NZS4           SKNKQNIDTISKHLLELGTYLDGSYRMMEQNTHNINKN-------THNINKLSKELQTGL
NZV1           SKNKQNIDTISKHLLELGTYLDGSYRMMEQNTHNINKN-------THNINKLSKELQTGL
82-029362      SQNKQNIDTISEYLLKLGTYLDSSYRMMEQNTHNINKN-------THNINKLSKELQTGL
6644           SQNKQNIDTISEYLLKLGTYLDSSYRMMEQNTHNINKN-------THNINKLSKELQTGL
HD183          SKNKQNIDTISKYLLELGTYLDGSYRMMEQNTHNINKNTHNINKNTHNINKLSKELQTGL
HMC46          SQNKQNIDTISEYLLKLGTYLDSSYRMMEQNTHNINKN-------THNINKLSKELQTGL
HMC56          SQNKQNIDTISEYLLKLGTYLDSSYRMMEQNTHNINKN-------THNINKLSKELQTGL
33921          LLNRAEILNVGNHLNSLLKYTEGAFAFLN---GGVTKNSADIFKNTQRIDALSKNLETGL
CIP542         LLNRAEILNVGNHLNSLLKYTEGAFAFLN---GGVTKNSADIFKNTQRIDALSKNLETGL
DMC64          LLNRAEILNVGNHLNSLLKYTEGAFAFLN---GGVTKNSADIFKNTQRIDALSKNLETGL
DMC111         LLNRAEILNVGNHLNSLLKYTEGAFAFLN---GGVTKNSADIFKNTQRIDALSKNLETGL
                 *: :* .:.::* .* .* : :: :::                  .*: ***:*:***

35000HP        ANQSALSMLVQPNGVGKTSVSAAVGGYRDKTALAIGVGSRITDRFTAKAGVAFNTYNGGM
NZS1           ANQSALSMLVQPNGVGKTSVSAAVGGYRDKTALAIGVGSRITDRFTAKAGVAFNTYNGGM
NZS2           ANQSALSMLVQPNGVGKTSVSAAVGGYRDKTALAIGVGSRITDRFTAKAGVAFNTYNGGM
NZS3           ANQSALSMLVQPNGVGKTSVSAAVGGYRDKTALAIGVGSRITDRFTAKAGVAFNTYNGGM
NZS4           ANQSALSMLVQPNGVGKTSVSAAVGGYRDKTALAIGVGSRITDRFTAKAGVAFNTYNGGM
NZV1           ANQSALSMLVQPNGVGKTSVSAAVGGYRDKTALAIGVGSRITDRFTAKAGVAFNTYNGGM
82-029362      ANQSALSMLVQPNGVGKTSVSAAVGGYRDKTALAIGVGSRITDRFTAKAGVAFNTYNGGM
6644           ANQSALSMLVQPNGVGKTSVSAAVGGYRDKTALAIGVGSRITDRFTAKAGVAFNTYNGGM
HD183          ANQSALSMLVQPNGVGKTSVSAAVGGYRDKTALAIGVGSRITDRFTAKAGVAFNTYNGGM
HMC46          ANQSALSMLVQPNGVGKTSVSAAVGGYRDKTALAIGVGSRITDRFTAKAGVAFNTYNGGM
HMC56          ANQSALSMLVQPNGVGKTSVSAAVGGYRDKTALAIGVGSRITDRFTAKAGVAFNTYNGGM
33921          ANQSALSMLVQPNGVGKTSVSAAVGGYRGKSALAIGVGSRITDAFTAKAGVAFNTYNGGM
CIP542         ANQSALSMLVQPNGVGKTSVSAAVGGYRGKSALAIGVGSRITDAFTAKAGVAFNTYNGGM
DMC64          ANQSALSMLVQPNGVGKTSVSAAVGGYRGKSALAIGVGSRITDAFTAKAGVAFNTYNGGM
DMC111         ANQSALSMLVQPNGVGKTSVSAAVGGYRGKSALAIGVGSRITDAFTAKAGVAFNTYNGGM
               **************************** *:************ ****************

35000HP        SYGASVGYEF
NZS1           SYGASVGYEF
NZS2           SYGASVGYEF
NZS3           SYGASVGYEF
NZS4           SYGASVGYEF
```

```
NZV1           SYGASVGYEF
82-029362      SYGASVGYEF
6644           SYGASVGYEF
HD183          SYGASVGYEF
HMC46          SYGASVGYEF
HMC56          SYGASVGYEF
33921          SYGASVGYEF
CIP542         SYGASVGYEF
DMC64          SYGASVGYEF
DMC111         SYGASVGYEF
               **********
```

# 6) FgbA (HD0192)

```
35000HP        MKKSILALVLGTTFTLAACDKPQVEEMKQTVTDTAANTKAIVVEKAGDMKESASEMKDAA
82-029362      MKKSILALVLGTTFTLAACDKPQVEEMKQTVTDTAANTKAIVVEKAGDMKESASEMKDAA
6644           MKKSILALVLGTTFTLAACDKPQVEEMKQTVTDTAANTKAIVVEKAGDMKESASEMKDAA
HMC46          MKKSILALVLGTTFTLAACDKPQVEEMKQTVTDTAANTKAIVVEKAGDMKESASEMKDAA
HMC56          MKKSILALVLGTTFTLAACDKPQVEEMKQTVTDTAANTKAIVVEKAGDMKESASEMKDAA
33921          MKKSILALVLGTTFTLAACDKPQVEEMKQTVTDTAANTKAIVVEKAGDMKESANEMKDAA
CIP542         MKKSILALVLGTTFTLAACDKPQVEEMKQTVTDTAANTKAIVVEKAGDMKESANEMKDAA
DMC64          MKKSILALVLGTTFTLAACDKPQVEEMKQTVTDTAANTKAIVVEKAGDMKESANEMKDAA
DMC111         MKKSILALVLGTTFTLAACDKPQVEEMKQTVTDTAANTKAIVVEKAGDMKESANEMKDAA
               *****************************************************.******

35000HP        KAKLEDMKESAAEAKESLAEKANEMKDAAKAKLEGMKESAAEAKESLAEKANEMKDAAKA
82-029362      KAKLEDMKEAAAE-------------------------------------ASEMKDAAKA
6644           KAKLEDMKEAAAE-------------------------------------ASEMKDAAKA
HMC46          KAKLEDMKEAAAE-------------------------------------ASEMKDAAKA
HMC56          KAKLEDMKEAAAE-------------------------------------ASEMKDAAKA
33921          KAKLEDMKEAAAE-------------------------------------ANEMKDAAKA
CIP542         KAKLEDMKEAAAE-------------------------------------ANEMKDAAKA
DMC64          KAKLEDMKEAAAE-------------------------------------ANEMKDAAKA
DMC111         KAKLEDMKEAAAE-------------------------------------ANEMKDAAKA
               *********:***                                     *.********

35000HP        KLEDMKEAAADKKAEMAEKMDSAADAMKEKVDEMKK*--
82-029362      KLEDMKEAAADKKAEMAEKMDSAADAMKEKVDEMKK--*
6644           KLEDMKEAAADKKAEMAEKMDSAADAMKEKVDEMKK--*
HMC46          KLEDMKEAAADKKAEMAEKMDSAADAMKEKVDEMKK--*
HMC56          KLEDMKEAAADKKAEMAEKMDSAADAMKEKVDEMKK--*
33921          KLEDMKEAAADKKAEMAEKKKWTPQLMQ*KKKWTK*KNX
CIP542         KLEDMKEAAADKKAEMAEKKKWTPQLMQ*KKKWTK*KNX
DMC64          KLEDMKEAAADKKAEMAEKKKWTPQLMQ*KKKWTK*KNX
DMC111         KLEDMKEAAADKKAEMAEKKKWTPQLMQ*KKKWTK*KNX
               ******************* . : : *: * .  *
```

**Note:** The FgbA sequence of NZS1, NZS2, NZS3, NZS4, and NZV1 were identical to that of 35000HP.

# 7) Flp1 (HD1312)

```
35000HP        MLSVLMTQAYISATESLRTSIQRFRKNQQGVTAIEYGLIAVAVAILIIAVFYNNQGFLMK
NZS1           MLSVLMTQAYISATESLRTSIQRFRKNQQGVTAIEYGLIAVAVAILIIAVFYNNQGFLMK
NZS2           MLSVLMTQAYISATESLRTSIQRFRKNQQGVTAIEYGLIAVAVAILIIAVFYNNQGFLMK
NZS3           MLSVLMTQAYISATESLRTSIQRFRKNQQGVTAIEYGLIAVAVAILIIAVFYNNQGFLMK
NZS4           MLSVLMTQAYISATESLRTSIQRFRKNQQGVTAIEYGLIAVAVAILIIAVFYNNQGFLMK
NZV1           MLSVLMTQAYISATESLRTSIQRFRKNQQGVTAIEYGLIAVAVAILIIAVFYNNQGFLMK
82-029362      MLSVLMTQAYISATESLRTSIQRFRKNQQGVTAIEYGLIAVAVAILIIAVFYNNQGFLMK
6644           MLSVLMTQAYISATESLRTSIQRFRKNQQGVTAIEYGLIAVAVAILIIAVFYNNQGFLMK
HD183          MLSVLMTQAYISATESLRTSIQRFRKNQQGVTAIEYGLIAVAVAILIIAVFYNNQGFLMK
HMC46          MLSVLMTQAYISATESLRTSIQRFRKNQQGVTAIEYGLIAVAVAILIIAVFYNNQGFLMK
HMC56          MLSVLMTQAYISATESLRTSIQRFRKNQQGVTAIEYGLIAVAVAILIIAVFYNNQGFLMK
33921          MLSTLTTQAYISATESVRAGIRRFKENQQGVTAIEYGLIAVAVAVLIIAAFYSENSFIKK
CIP542         MLSTLTTQAYISATESVRAGIRRFKENQQGVTAIEYGLIAVAVAVLIIAAFYSENSFIKK
DMC64          MLSTLTTQAYISATESVRAGIRRFKENQQGVTAIEYGLIAVAVAVLIIAAFYSENSFIKK
DMC111         MLSTLTTQAYISATESVRAGIRRFKENQQGVTAIEYGLIAVAVAVLIIAAFYSENSFIKK
               ***.* **********:*:.*:**::******************:****.**.::.*: *

35000HP        LKTKFSDLATGISSANGTTSLNSFK*
NZS1           LKTKFSDLATGISSANGTTSLNSFK*
NZS2           LKTKFSDLATGISSANGTTSLNSFK*
```

```
NZS3           LKTKFSDLATGISSANGTTSLNSFK*
NZS4           LKTKFSDLATGISSANGTTSLNSFK*
NZV1           LKTKFSDLATGISSANGTTSLNSFK*
82-029362      LKTKFSDLATGISSANGTTSLNSFK*
6644           LKTKFSDLATGISSANGTTSLNSFK*
HD183          LKTKFSDLATGISSANGTTSLNSFK*
HMC46          LKTKFSDLATGISSANGTTSLNSFK*
HMC56          LKTKFSDLATGISSANGTTSLNSFK*
33921          LQSKFSSLASGISNQNINFT*-----
CIP542         LQSKFSSLASGISNQNINFT*-----
DMC64          LQSKFSSLASGISNQNINFT*-----
DMC111         LQSKFSSLASGISNQNINFT*-----
               *::***.**:***. * . :
```

# 8) Flp2 (HD1311)

```
35000HP        MLSVLMTQAYISATESLRTSIQRFRKNQQGVTAIEYGLIAVAVAILIIAVFYNNQGFLMK
NZS1           MLSVLMTQAYISATESLRTSIQRFRKNQQGVTAIEYGLIAVAVAILIIAVFYNNQGFLMK
NZS2           MLSVLMTQAYISATESLRTSIQRFRKNQQGVTAIEYGLIAVAVAILIIAVFYNNQGFLMK
NZS3           MLSVLMTQAYISATESLRTSIQRFRKNQQGVTAIEYGLIAVAVAILIIAVFYNNQGFLMK
NZS4           MLSVLMTQAYISATESLRTSIQRFRKNQQGVTAIEYGLIAVAVAILIIAVFYNNQGFLMK
NZV1           MLSVLMTQAYISATESLRTSIQRFRKNQQGVTAIEYGLIAVAVAILIIAVFYNNQGFLMK
82-029362      MLSVLMTQAYISATESLRTSIQRFRKNQQGVTAIEYGLIAVAVAILIIAVFYNNQGFLMK
6644           MLSVLMTQAYISATESLRTSIQRFRKNQQGVTAIEYGLIAVAVAILIIAVFYNNQGFLMK
HD183          MLSVLMTQAYISATESLRTSIQRFRKNQQGVTAIEYGLIAVAVAILIIAVFYNNQGFLMK
HMC46          MLSVLMTQAYISATESLRTSIQRFRKNQQGVTAIEYGLIAVAVAILIIAVFYNNQGFLMK
HMC56          MLSVLMTQAYISATESLRTSIQRFRKNQQGVTAIEYGLIAVAVAILIIAVFYNNQGFLMK
33921          --------VYISTTEILCSSIQRFKKNQRGVTAIEYGLIAVAVAVLIIAAFYSENSFIKK
CIP542         --------VYISTTEILCSSIQRFKKNQRGVTAIEYGLIAVAVAVLIIAAFYSENSFIKK
DMC64          --------VYISTTEILCSSIQRFKKNQRGVTAIEYGLIAVAVAVLIIAAFYSENSFIKK
DMC111         --------VYISTTEILCSSIQRFKKNQRGVTAIEYGLIAVAVAVLIIAAFYSENSFIKK
                       .***:** * :*****:***:***************:****.**.::.*: *

35000HP        LKTKFSDLATGISAQSVSFNN*----
NZS1           LKTKFSDLATGISAQSVSFNN*----
NZS2           LKTKFSDLATGISAQSVSFNN*----
NZS3           LKTKFSDLATGISAQSVSFNN*----
NZS4           LKTKFSDLATGISAQSVSFNN*----
NZV1           LKTKFSDLATGISAQSVSFNN*----
82-029362      LKTKFSDLATGISAQSVSFNN*----
6644           LKTKFSDLATGISAQSVSFNN*----
HD183          LKTKFSDLATGISAQSVSFNN*----
HMC46          LKTKFSDLATGISAQSVSFNN*----
HMC56          LKTKFSDLATGISAQSVSFNN*----
33921          LQSKFSNLASGINGTANELNNFHKNS
CIP542         LQSKFSNLASGINGTANELNNFHKNS
DMC64          LQSKFSNLASGINGTANELNNFHKNS
DMC111         LQSKFSNLASGINGTANELNNFHKNS
               *::***:**:**.. : .:**
```

# 9) Flp3 (HD1310)

```
35000HP        MLITIIITKPYLSMKETLISWFNCFKINQKGVTAIEYGLIAVAVAILIIAVFYSESGFLFA
NZS1           MLITIIITKPYLSMKETLISWFNCFKINQKGVTAIEYGLIAVAVAILIIAVFYSESGFLFA
NZS2           MLITIIITKPYLSMKETLISWFNCFKINQKGVTAIEYGLIAVAVAILIIAVFYSESGFLFA
NZS3           MLITIIITKPYLSMKETLISWFNCFKINQKGVTAIEYGLIAVAVAILIIAVFYSESGFLFA
NZS4           MLITIIITKPYLSMKETLISWFNCFKINQKGVTAIEYGLIAVAVAILIIAVFYSESGFLFA
NZV1           MLITIIITKPYLSMKETLISWFNCFKINQKGVTAIEYGLIAVAVAILIIAVFYSESGFLFA
82-029362      MLITIIITKPYLSMKETLISWFNCFKINQKGVTAIEYGLIAVAVAILIIAVFYSESGFLFA
6644           MLITIIITKPYLSMKETLISWFNCFKINQKGVTAIEYGLIAVAVAILIIAVFYSESGFLFA
HD183          MLITIIITKPYLSMKETLISWFNCFKINQKGVTAIEYGLIAVAVAILIIAVFYSESGFLFA
HMC46          MLITIIITKPYLSMKETLISWFNCFKINQKGVTAIEYGLIAVAVAILIIAVFYSESGFLFA
HMC56          MLITIIITKPYLSMKETLISWFNCFKINQKGVTAIEYGLIAVAVAILIIAVFYSESGFLFA
33921          MLSTLTTKAYISTSETLRNKMKTFRQDQQGVTAIEYGLIAVAVAVLIIAVFYSEDGFLFK
CIP542         MLSTLTTKAYISTSETLRNKMKTFRQDQQGVTAIEYGLIAVAVAVLIIAVFYSEDGFLFK
DMC64          MLSTLTTKAYISTSETLRNKMKTFRQDQQGVTAIEYGLIAVAVAVLIIAVFYSEDGFLFK
DMC111         MLSTLTTKAYISTSETLRNKMKTFRQDQQGVTAIEYGLIAVAVAVLIIAVFYSEDGFLFK
               ** *: ** *:* .*** . :: *: :*:***************:*********.****

35000HP        LKEKFFQLEGGVGKAAPDSYLLNFNKGRL*
```

```
NZS1          LKEKFFQLEGGVGKAAPDSYLLNFNKGRL*
NZS2          LKEKFFQLEGGVGKAAPDSYLLNFNKGRL*
NZS3          LKEKFFQLEGGVGKAAPDSYLLNFNKGRL*
NZS4          LKEKFFQLEGGVGKAAPDSYLLNFNKGRL*
NZV1          LKEKFFQLEGGVGKAAPDSYLLNFNKGRL*
82-029362     LKEKFFQLEGGVGKAAPDSYLLNFNKGRL*
6644          LKEKFFQLEGGVGKAAPDSYLLNFNKGRL*
HD183         LKEKFFQLEGGVGKAAPDSYLLNFNKGRL*
HMC46         LKEKFFQLEGGVGKAAPDSYLLNFNKGRL*
HMC56         LKEKFFQLEGGVGKAAPDSYLLNFNKGRL*
33921         LREKFFHLEVGLGNAAPDGDLI--------
CIP542        LREKFFHLEVGLGNAAPDGDLI--------
DMC64         LREKFFHLEVGLGNAAPDGDLI--------
DMC111        LREKFFHLEVGLGNAAPDGDLI--------
              *:****:** *:*:****. *:
```

# 10) Hfq (HD0741)

```
35000HP     MAKGQSLQDPYLNALRRERIPVSIYLVNGIKLQGQIESFDQFIILLKNTVSQMVYKHAIS
33921       MAKGQSLQDPYLNALRRERIPVSIYLVNGIKLQGQIESFDQFVILLKNTVSQMVYKHAIS
CIP542      MAKGQSLQDPYLNALRRERIPVSIYLVNGIKLQGQIESFDQFVILLKNTVSQMVYKHAIS
DMC64       MAKGQSLQDPYLNALRRERIPVSIYLVNGIKLQGQIESFDQFVILLKNTVSQMVYKHAIS
DMC111      MAKGQSLQDPYLNALRRERIPVSIYLVNGIKLQGQIESFDQFVILLKNTVSQMVYKHAIS
            ******************************************:*****************

35000HP     TVVPARSISHNNNGSSQAQAPQQAVQTTQPVEAIVATDKME*
33921       TVVPARSISHNNNGSSQAQAPQQAVQTTQPVEATVATDKME*
CIP542      TVVPARSISHNNNGSSQAQAPQQAVQTTQPVEATVATDKME*
DMC64       TVVPARSISHNNNGSSQAQAPQQAVQTTQPVEATVATDKME*
DMC111      TVVPARSISHNNNGSSQAQAPQQAVQTTQPVEATVATDKME*
            ********************************* ********
```

**Note:** The Hfq sequence of NZS1, NZS2, NZS3, NZS4, NZV1, 82-029362, 6644, HD183, HMC46, and HMC56 were identical to that of 35000HP.

# 11) HgbA (HD2025)

```
35000HP       MKANKLSAITLCILGYAHTVYAESNMQTEKLETIVVSSEDDSVHNKNVGEIKKNAKALSK
82-029362     MKANKLSAITLCILGYAHTVYAESNMQTEKLETIVVSSEDDSVHNKNVGEIKKNAKALSK
6644          MKANKLSAITLCILGYAHTVYAESNMQTEKLETIVVSSEDDSVHNKNVGEIKKNAKALSK
HMC46         MKANKLSAITLCILGYAHTVYAESNMQTEKLETIVVSSEDDSVHNKNVGEIKKNAKALSK
HMC56         MKANKLSAITLCILGYAHTVYAESNMQTEKLETIVVSSEDDSVHNKNVGEIKKNAKALSK
33921         MKTNKLSAITLCILGYAHTVYAESNMQTEKLETIVVSSEDDSVHNKNVGEIKKNAKALSK
CIP542        MKTNKLSAITLCILGYAHTVYAESNMQTEKLETIVVSSEDDSVHNKNVGEIKKNAKALSK
DMC64         MKTNKLSAITLCILGYAHTVYAESNMQTEKLETIVVSSEDDSVHNKNVGEIKKNAKALSK
DMC111        MKANKLSAITLCILGYAHTVYAESNMQTEKLETIVVSSEDDSVHNKNVGEIKKNAKALSK
              **:*********************************************************

35000HP       QQVQDSRDLVRYETGVTVVEKGRFGSSGYAIRGVDENRVAVVVDGLHQAETISSQGFKEL
82-029362     QQVQDSRDLVRYETGVTVVEKGRFGSSGYAIRGVDENRVAVVVDGLHQAETISSQGFKEL
6644          QQVQDSRDLVRYETGVTVVEKGRFGSSGYAIRGVDENRVAVVVDGLHQAETISSQGFKEL
HMC46         QQVQDSRDLVRYETGVTVVEKGRFGSSGYAIRGVDENRVAVVVDGLHQAETISSQGFKEL
HMC56         QQVQDSRDLVRYETGVTVVEKGRFGSSGYAIRGVDENRVAVVVDGLHQAETISSQGFKEL
33921         QQVQDSRDLVRYETGITVVEKGRFGSSGYAIRGVDENRVAVVVDGLHQAETISSQGFKEL
CIP542        QQVQDSRDLVRYETGITVVEKGRFGSSGYAIRGVDENRVAVVVDGLHQAETISSQGFKEL
DMC64         QQVQDSRDLVRYETGITVVEKGRFGSSGYAIRGVDENRVAVVVDGLHQAETISSQGFKEL
DMC111        QQVQDSRDLVRYETGVTVVEKGRFGSSGYAIRGVDENRVAVVVDGLHQAETISSQGFKEL
              ***************:********************************************

35000HP       FEGYGNFNNTRNGVEVENLKQAVIQKGADAIRTGSGSLGGTVSFESKDARDYLIDKNYHF
82-029362     FEGYGNFNNTRNGVEVENLKQAVIQKGADAIRTGSGSLGGTVSFESKDARDYLIDKNYHF
6644          FEGYGNFNNTRNGVEVENLKQAVIQKGADAIRTGSGSLGGTVSFESKDARDYLIDKNYHF
HMC46         FEGYGNFNNTRNGVEVENLKQAVIQKGADAIRTGSGSLGGTVSFESKDARDYLIDKNYHF
HMC56         FEGYGNFNNTRNGVEVENLKQAVIQKGADAIRTGSGSLGGTVSFESKDARDYLIDKNYHF
33921         FEGYGNFNNTRNGVEVENLKQVVIQKGADAIRTGSGSLGGTVSFESKDVRDYLIDKNYHF
CIP542        FEGYGNFNNTRNGVEVENLKQVVIQKGADAIRTGSGSLGGTVSFESKDVRDYLIDKNYHF
DMC64         FEGYGNFNNTRNGVEVENLKQVVIQKGADAIRTGSGSLGGTVSFESKDVRDYLIDKNYHF
DMC111        FEGYGNFNNTRNGVEVENLKQAVIQKGADAIRTGSGSLGGTVSFESKDARDYLIDKNYHF
              *********************.**************************.***********

35000HP       GYKTGYSSADNQKLHSVTAAGRYSDFDLLAVHTQRHGNELRNYGYRHYDGSVVRKEREKA
```

```
82-029362      GYKTGYSSADNQKLHSVTAAGRYSDFDLLAVHTQRHGNELRNYGYRHYDGSVVRKEREKA
6644           GYKTGYSSADNQKLHSVTAAGRYSDFDLLAVHTQRHGNELRNYGYRHYDGSVVRKEREKA
HMC46          GYKTGYSSADNQKLHSVTAAGRYSDFDLLAVHTQRHGNELRNYGYRHYDGSVVRKEREKA
HMC56          GYKTGYSSADNQKLHSVTAAGRYSDFDLLAVHTQRHGNELRNYGYRHYDGSVVRKEREKA
33921          GYKTGYSSADNQKLHSVTAAGRYSDFDLLAVHTQRHGNELRNYGHRHYDGSVVRKEREKA
CIP542         GYKTGYSSADNQKLHSVTAAGRYSDFDLLAVHTQRHGNELRNYGHRHYDGSVVRKEREKA
DMC64          GYKTGYSSADNQKLHSVTAAGRYSDFDLLAVHTQRHGNELRNYGHRHYDGSVVRKEREKA
DMC111         GYKTGYSSADNQKLHSVTAAGRYSDFDLLAVHTQRHGNELRNYGYRHYDGSVVRKEREKA
               ********************************************:***************

35000HP        DPYKITKQSSLIKIGYQLNDTNRFTLGYDDSRNTSRGTDWSNAFTSYNGGPFLKDVRHTN
82-029362      DPYKITKQSSLIKIGYQLNDTNRFTLGYDDSRNTSRGTDWSNAFTSYNGGPFLKDVRHTN
6644           DPYKITKQSSLIKIGYQLNDTNRFTLGYDDSRNTSRGTDWSNAFTSYNGGPFLKDVRHTN
HMC46          DPYKITKQSSLIKIGYQLNDTNRFTLGYDDSRNTSRGTDWSNAFTSYNGGPFLKDVRHTN
HMC56          DPYKITKQSSLIKIGYQLNDTNRFTLGYDDSRNTSRGTDWSNAFTSYNGGPFLKDVRHTN
33921          DPYKITKQSSLIKIGYQLNDTNRFTLGYDDSRNTSRGTDWSNAFTSYNGGPFLKDVRHTH
CIP542         DPYKITKQSSLIKIGYQLNDTNRFTLGYDDSRNTSRGTDWSNAFTSYNGGPFLKDVRHTH
DMC64          DPYKITKQSSLIKIGYQLNDTNRFTLGYDDSRNTSRGTDWSNAFTSYNGGPFLKDVRHTH
DMC111         DPYKITKQSSLIKIGYQLNDTNRFTLGYDDSRNTSRGTDWSNAFTSYNGGPFLKDVRHTN
               ***********************************************************.

35000HP        DQSNRKNISFVYENFDTNDFWDTLKITHNHQKIKLKARLDEYCDVNGEIDCPAIANPSGL
82-029362      DQSNRKNISFVYENFDTNDFWDTLKITHNHQKIKLKARLDEYCDVNGEIDCPAIANPSGL
6644           DQSNRKNISFVYENFDTNDFWDTLKITHNHQKIKLKARLDEYCDVNGEIDCPAIANPSGL
HMC46          DQSNRKNISFVYENFDTNDFWDTLKITHNHQKIKLKARLDEYCDVNGEIDCPAIANPSGL
HMC56          DQSNRKNISFVYENFDTNDFWDTLKITHNHQKIKLKARLDEYCDVNGEIDCPAIANPSGL
33921          DKSNRKNISVVYENFDTNDFWDTLKITHNHQKIKLKARLDEYCDVNGEIDCPTIANPSGL
CIP542         DKSNRKNISVVYENFDTNDFWDTLKITHNHQKIKLKARLDEYCDVNGEIDCPTIANPSGL
DMC64          DKSNRKNISVVYENFDTNDFWDTLKITHNHQKIKLKARLDEYCDVNGEIDCPTIANPSGL
DMC111         DQSNRKNISFVYENFDTNDFWDTLKITHNHQKIKLKARLDEYCDVNGEIDCPAIANPSGL
               *:*******.******************************************:*******

35000HP        YINDKGIFLDKHDGEITHKKEGEFNNYFDSKGKEVRVKGFNVDSILINCDQYDCSKPMQL
82-029362      YINDKGIFLDKHDGEITHKKEGEFNNYFDSKGKEVRVTGFNVDSILINCDQYDCSKPMQL
6644           YINDKGIFLDKHDGEITHKKEGEFNNYFDSKGKEVRVTGFNVDSILINCDQYDCSKPMQL
HMC46          YINDKGIFLDKHDGEITHKKEGEFNNYFDSKGKEVRVTGFNVDSILINCDQYDCSKPMQL
HMC56          YINDKGIFLDKHDGEITHKKEGEFNNYFDSKGKEVRVTGFNVDSILINCDQYDCSKPMQL
33921          YINDKGIFLDKHDGKITHKKEGEFNNYFDSKGKEVRVKEFNVDSILINCDQYDCSKPMQL
CIP542         YINDKGIFLDKHDGKITHKKEGEFNNYFDSKGKEVRVKEFNVDSILINCDQYDCSKPMQL
DMC64          YINDKGIFLDKHDGKITHKKEGEFNNYFDSKGKEVRVKEFNVDSILINCDQYDCSKPMQL
DMC111         YINDKGIFLDKHDGEITHKKEGEFNNYFDSKGKEVRVKGFNVDSILINCDQYDCSKPMQL
               **************:**********************. *********************

35000HP        LSSTNNGYGGSPNKYIYKTYELFEKTMNNGNGKYAVLEIRSSGHEKFSRVYLPSEKGYVE
82-029362      LSSTNNGYGGSPNKYIYKTYELFEKTMNNGNGKYAVLEIRSSGHEKFSRVYLPSEKGYVE
6644           LSSTNNGYGGSPNKYIYKTYELFEKTMNNGNGKYAVLEIRSSGHEKFSRVYLPSEKGYVE
HMC46          LSSTNNGYGGSPNKYIYKTYELFEKTMNNGNGKYAVLEIRSSGHEKFSRVYLPSEKGYVE
HMC56          LSSTNNGYGGSPNKYIYKTYELFEKTMNNGNGKYAVLEIRSSGHEKFSRVYLPSEKGYVE
33921          LSSTNNGYGNSPNEYKYITYELSEKTMNNGNGKYAVLESRGE-NKKFSRVYLPSEKGYVE
CIP542         LSSTNNGYGNSPNEYKYITYELSEKTMNNGNGKYAVLESRGE-NKKFSRVYLPSEKGYVE
DMC64          LSSTNNGYGNSPNEYKYITYELSEKPMNNGNGKYAVLESRGE-NKKFSRVYLPSEKGYVE
DMC111         LSSTNNGYGGSPNKYIYKTYELFEKTMNNGNGKYAVLEIRSSGHEKFSRVYLPSEKGYVE
               ********* ***:* * **** ** ************ *.. .:***************

35000HP        NQWKDRDLNTDTQQYNIDLTKSFKLKSVEHNATYGGLYSEVKKSMTNRAGYEAYNRQWWA
82-029362      NQWKDRDLNTDTQQYNIDLTKSFKLKSVEHNATYGGLYSEVKKSMTNRAGYEAYNRQWWA
6644           NQWKDRDLNTDTQQYNIDLTKSFKLKSVEHNATYGGLYSEVKKSMTNRAGYEAYNRQWWA
HMC46          NQWKDRDLNTDTQQYNIDLTKSFKLKSVEHNATYGGLYSEVKKSMTNRAGYEAYNRQWWA
HMC56          NQWKDRDLNTDTQQYNIDLTKSFKLKSVEHNATYGGLYSEVKKSMTNRAGYEAYNRQWWA
33921          NQWRDRDLNTDTQQYNIDLTKSFKLKSVEHNATYGGLYSEVKKSMINRAGYEAYNRQWWA
CIP542         NQWRDRDLNTDTQQYNIDLTKSFKLKSVEHNATYGGLYSEVKKSMINRAGYEAYNRQWWA
DMC64          NQWRDRDLNTDTQQYNIDLTKSFKLKSVEHNATYGGLYSEVKKSMINRAGYEAYNRQWWA
DMC111         NQWKDRDLNTDTQQYNIDLTKSFKLKSVEHNATYGGLYSEVKKSMTNRAGYEAYNRQWWA
               ***:****************************************** *************

35000HP        NIFFGKENNKPNKCQPYNGNSFTTLCSHEDRLFSFLIPVKTKTGALYVTDKIKLNDKVNL
82-029362      NIFFGKENNKPNKCQPYNGNSFTTLCSHEDRLFSFLIPVKTKTGALYVTDKIKLNDKVNL
6644           NIFFGKENNKPNKCQPYNGNSFTTLCSHEDRLFSFLIPVKTKTGALYVTDKIKLNDKVNL
HMC46          NIFFGKENNKPNKCQPYNGNSFTTLCSHEDRLFSFLIPVKTKTGALYVTDKIKLNDKVNL
HMC56          NIFFGKENNKPNKCQPYNGNSFTTLCSHEDRLFSFLIPVKTKTGALYVTDKIKLNDKVNL
33921          NIFFGEENGKPNKCQPYNGNSFTTLCSHEDRLFSFLIPVKTKTGALYVTDKIKLNDKVNL
CIP542         NIFFGEENGKPNKCQPYNGNSFTTLCSHEDRLFSFLIPVKTKTGALYVTDKIKLNDKVNL
DMC64          NIFFGEENGKPNKCQPYNGNSFTTLCSHEDRLFSFLIPVKTKTGALYVTDKIKLNDKVNL
```

```
DMC111          NIFFGKENNKPNKCQPYNGNSFTTLCSHEDRLFSFLIPVKTKTGALYVTDKIKLNDKVNL
                *****:** ***************************************************

35000HP         DVAYRYDRIKHDPKYIPGTTPKLPTDLILGRFIEFKPKNTYATQDEKNENAEKNAVYLAS
82-029362       DVAYRYDRIKHDPKYIPGTTPKLPTDLILGRFIEFKPKNTYATQDEKNENAEKNAVYLAS
6644            DVAYRYDRIKHDPKYIPGTTPKLPTDLILGRFIEFKPKNTYATQDEKNENAEKNAVYLAS
HMC46           DVAYRYDRIKHDPKYIPGTTPKLPTDLILGRFIEFKPKNTYATQDEKNENAEKNAVYLAS
HMC56           DVAYRYDRIKHDPKYIPGTTPKLPTDLILGRFIEFKPKNTYATQDEKNENAEKNAVYLAS
33921           DLAYRYDRIKHDPKYIPGTTPKLPTDLILGRFIELKPKNTYATQDEKNENAEKNAVYLAS
CIP542          DLAYRYDRIKHDPKYIPGTTPKLPTDLILGRFIELKPKNTYATQDEKNENAEKNAVYLAS
DMC64           DLAYRYDRIKHDPKYIPGTTPKLPTDLILGRFIELKPKNTYATQDEKNENAEKNAVYLAS
DMC111          DVAYRYDRIKHDPKYIPGTTPKLPTDLILGRFIEFKPKNTYATQDEKNENAEKNAVYLAS
                *:********************************:*************************

35000HP         KKTKFSANSYSATFSFDPMDFLKIQAKYATGFRAPTSDEIYFVFQHPSFSIYPNLYLKAE
82-029362       KKTKFSANSYSATFSFDPMDFLKIQAKYATGFRAPTSDEIYFVFQHPSFSIYPNLYLKAE
6644            KKTKFSANSYSATFSFDPMDFLKIQAKYATGFRAPTSDEIYFVFQHPSFSIYPNLYLKAE
HMC46           KKTKFSANSYSATFSFDPMDFLKIQAKYATGFRAPTSDEIYFVFQHPSFSIYPNLYLKAE
HMC56           KKTKFSANSYSATFSFDPMDFLKIQAKYATGFRAPTSDEIYFVFQHPSFSIYPNLYLKAE
33921           KKTKFSANSYSATFSFDPMDFLKIQAKYATGFRAPTSDEIYFVFQHPSFSIYPNLDLKAE
CIP542          KKTKFSANSYSATFSFDPMDFLKIQAKYATGFRAPTSDEIYFVFQHPSFSIYPNLDLKAE
DMC64           KKTKFSANSYSATFSFDPMDFLKIQAKYATGFRAPTSDEIYFVFQHPSFSIYPNLDLKAE
DMC111          KKTKFSANSYSATFSFDPMDFLKIQAKYATGFRAPTSDEIYFVFQHPSFSIYPNLYLKAE
                ******************************************************* ****

35000HP         RSKNKEVAITLHKQKSFLTVNLFQTDYKDFLDLAYLKKGSLPYGNGGSQLETLLYQNVNR
82-029362       RSKNKEVAITLHKQKSFLTVNLFQTDYKDFLDLAYLKKGSLPYGNGGSQLETLLYQNVNR
6644            RSKNKEVAITLHKQKSFLTVNLFQTDYKDFLDLAYLKKGSLPYGNGGSQLETLLYQNVNR
HMC46           RSKNKEVAITLHKQKSFLTVNLFQTDYKDFLDLAYLKKGSLPYGNGGSQLETLLYQNVNR
HMC56           RSKNKEVAITLHKQKSFLTVNLFQTDYKDFLDLAYLKKGSLPYGNGGSQLETLLYQNVNR
33921           RSKNKEVAITLHKQQSFLTVNLFQTDYKDFLDLAYLKKGSLPYGNGGSQLETLLYQNVNR
CIP542          RSKNKEVAITLHKQQSFLTVNLFQTDYKDFLDLAYLKKGSLPYGNGGSQLETLLYQNVNR
DMC64           RSKNKEVAITLHKQQSFLTVNLFQTDYKDFLDLAYLKKGSLPYGNGGSQLETLLYQNVNR
DMC111          RSKNKEVAITLHKQKSFLTVNLFQTDYKDFLDLAYLKKGSLPYGNGGSQLETLLYQNVNR
                **************:*********************************************

35000HP         DKARVKGLEVNSKLHLGDVWRTLDGFNLSYKLSLQKGRMSSKVGEEGKQRDTNKLDTPMN
82-029362       DKARVKGLEVNSKLHLGDVWRTLDGFNLSYKLSLQKGRMSSKVGEEGKQRDTNKLDTPMN
6644            DKARVKGLEVNSKLHLGDVWRTLDGFNLSYKLSLQKGRMSSKVGEEGKQRDTNKLDTPMN
HMC46           DKARVKGLEVNSKLHLGDVWRTLDGFNLSYKLSLQKGRMSSKVGEEGKQRDTNKLDTPMN
HMC56           DKARVKGLEVNSKLHLGDVWRTLDGFNLSYKLSLQKGRMSSKVGEEGKQRDTNKLDTPMN
33921           DKARVKGLEVNSRLHLGDVWGVLDGFNLSYKLSLQKGRMSSKVGEEGKQRDTNKLDTPMN
CIP542          DKARVKGLEVNSRLHLGDVWGVLDGFNLSYKLSLQKGRMSSKVGEEGKQRDTNKLDTPMN
DMC64           DKARVKGLEVNSRLHLGDVWGVLDGFNLSYKLSLQKGRMSSKVGEEGKQRDTNKLDTPMN
DMC111          DKARVKGLEVNSKLHLGDVWRTLDGFNLSYKLSLQKGRMSSKVGEEGKQRDTNKLDTPMN
                ************:******* .**************************************

35000HP         AIQPQTHVVGVGYEHPQEKFGVDMYLTHASAKKEKDTFNMFYDGKDQKDQHIKWRSDRYT
82-029362       AIQPQTHVVGVGYEHPQEKFGVDMYLTHASAKKEKDTFNMFYDGKDQKDQHIKWRSDRYT
6644            AIQPQTHVVGVGYEHPQEKFGVDMYLTHASAKKEKDTFNMFYDGKDQKDQHIKWRSDRYT
HMC46           AIQPQTHVVGVGYEHPQEKFGVDMYLTHASAKKEKDTFNMFYDGKDQKDQHIKWRSDRYT
HMC56           AIQPQTHVVGVGYEHPQEKFGVDMYLTHASAKKEKDTFNMFYDGKDQKDQHIKWRSDRYT
33921           VIQPQTHVVGLGYEHTQEKFGVDMYLTHASAKKEKDTFNMFYDGKDQKDQHIKWRSDSYT
CIP542          VIQPQTHVVGLGYEHTQEKFGVDMYLTHASAKKEKDTFNMFYDGKDQKDQHIKWRSDSYT
DMC64           VIQPQTHVVGLGYEHTQEKFGVDMYLTHASAKKEKDTFNMFYDGKDQKDQHIKWRSDSYT
DMC111          AIQPQTHVVGVGYEHPQEKFGVDMYLTHASAKKEKDTFNMFYDGKDQKDQHIKWRSDRYT
                .*********:**** ***************************************** **

35000HP         LVDLIAYVKPVKNVTLRAGVYNLTNREYGTWDSIRSIRPFGTTNLINQETGKGIKRFNAP
82-029362       LVDLIAYVKPVKNVTLRAGVYNLTNREYGTWDSIRSIRPFGTTNLINQETGKGIKRFNAP
6644            LVDLIAYVKPVKNVTLRAGVYNLTNREYGTWDSIRSIRPFGTTNLINQETGKGIKRFNAP
HMC46           LVDLIAYVKPVKNVTLRAGVYNLTNREYGTWDSIRSIRPFGTTNLINQETGKGIKRFNAP
HMC56           LVDLIAYVKPVKNVTLRAGVYNLTNREYGTWDSIRSIRPFGTTNLINQETGKGIKRFNAP
33921           LVDLIAYIKPVKNVTLRAGVYNLTNREYGTWDSIRSIRPFGTTNLINQKTGKGIKRFNAP
CIP542          LVDLIAYIKPVKNVTLRAGVYNLTNREYGTWDSIRSIRPFGTTNLINQKTGKGIKRFNAP
DMC64           LVDLIAYIKPVKNVTLRAGVYNLTNREYGTWDSIRSIRPFGTTNLINQKTGKGIKRFNAP
DMC111          LVDLIAYVKPVKNVTLRAGVYNLTNREYGTWDSIRSIRPFGTTNLINQETGKGIKRFNAP
                *******:****************************************:***********

35000HP         GRNFRVNAEITF*
82-029362       GRNFRVNAEITF*
6644            GRNFRVNAEITF*
HMC46           GRNFRVNAEITF*
```

```
HMC56          GRNFRVNAEITF*
33921          GRNFRVNAEITF*
CIP542         GRNFRVNAEITF*
DMC64          GRNFRVNAEITF*
DMC111         GRNFRVNAEITF*
               *************
```

**Note:** The HgbA sequence of NZS1, NZS2, NZS3, NZS4, and NZV1 were identical to that of 35000HP.

# 12) LspA1 (HD1505)

```
35000HP        MNNKRYKLIFSKVKNCLVPVAENIKSASGNSGSSSNSKIAEDQEEEPDSLACSLSPLSSS
NZS1           MNNKRYKLIFSKVKNCLVPVAENIKSASGNSGSSSNSKIAEDQEEEPDSLACSLSPLSSS
NZS2           MNNKRYKLIFSKVKNCLVPVAENIKSASGNSGSSSNSKIAEDQEEEPDSLACSLSPLSSS
NZS3           MNNKRYKLIFSKVKNCLVPVAENIKSASGNSGSSSNSKIAEDQEEEPDSLACSLSPLSSS
NZS4           MNNKRYKLIFSKVKNCLVPVAENIKSASGNSGSSSNSKIAEDQEEEPDSLACSLSPLSSS
NZV1           MNNKRYKLIFSKVKNCLVPVAENIKSASGNSGSSSNSKIAEDQEEEPDSLACSLSPLSSS
82-029362      MNNKRYKLIFSKVKNCLVPVAENIKSASGNSGSSSNSKIAEDQEEEPDSLACSLSPLSSS
6644           MNNKRYKLIFSKVKNCLVPVAENIKSASGNSGSSSNSKIAEDQEEEPDSLACSLSPLSSS
HD183          MNNKRYKLIFSKVKNCLVPVAENIKSASGNSGSSSNSKIAEDQEEEPDSLACSLSPLSSS
HMC46          MNNKRYKLIFSKVKNCLVPVAENIKSASGNSGSSSNSKIAEDQEEEPDSLACSLSPLSSS
HMC56          MNNKRYKLIFSKVKNCLVPVAENIKSASGNSGSSSNSKIAEDQEEEPDSLACSLSPLSSS
               ************************************************************

35000HP        IHLGLHNHSPLKVFKGKSLSVVLLSLMPATPLLAQQNYAEALNGKVYVDSQHSSTRIYEQ
NZS1           IHLGLHNHSPLKVFKGKSLSVVLLSLMPATPLLAQQNYAEALNGKVYVDSQHSSTRIYEQ
NZS2           IHLGLHNHSPLKVFKGKSLSVVLLSLMPATPLLAQQNYAEALNGKVYVDSQHSSTRIYEQ
NZS3           IHLGLHNHSPLKVFKGKSLSVVLLSLMPATPLLAQQNYAEALNGKVYVDSQHSSTRIYEQ
NZS4           IHLGLHNHSPLKVFKGKSLSVVLLSLMPATPLLAQQNYAEALNGKVYVDSQHSSTRIYEQ
NZV1           IHLGLHNHSPLKVFKGKSLSVVLLSLMPATPLLAQQNYAEALNGKVYVDSQHSSTRIYEQ
82-029362      IHLGLHNHSPLKVFKGKSLSVVLLSLMPATPLLAQQNYAEALNGKVYVDSQHSSTRIYEQ
6644           IHLGLHNHSPLKVFKGKSLSVVLLSLMPATPLLAQQNYAEALNGKVYVDSQHSSTRIYEQ
HD183          IHLGLHNHSPLKVFKGKSLSVVLLSLMPATPLLAQQNYAEALNGKVYVDSQHSSTRIYEQ
HMC46          IHLGLHNHSPLKVFKGKSLSVVLLSLMPATPLLAQQNYAEALNGKVYVDSQHSSTRIYEQ
HMC56          IHLGLHNHSPLKVFKGKSLSVVLLSLMPATPLLAQQNYAEALNGKVYVDSQHSSTRIYEQ
               ************************************************************

35000HP        KTNDNSKDGIVVVEIANPEVDGVSDNRFKEFNIPNSAVFNNSRTESTSQLVGKLHANIQL
NZS1           KTNDNSKDGIVVVEIANPEVDGVSDNRFKEFNIPNSAVFNNSRTESTSQLVGKLHANIQL
NZS2           KTNDNSKDGIVVVEIANPEVDGVSDNRFKEFNIPNSAVFNNSRTESTSQLVGKLHANIQL
NZS3           KTNDNSKDGIVVVEIANPEVDGVSDNRFKEFNIPNSAVFNNSRTESTSQLVGKLHANIQL
NZS4           KTNDNSKDGIVVVEIANPEVDGVSDNRFKEFNIPNSAVFNNSRTESTSQLVGKLHANIQL
NZV1           KTNDNSKDGIVVVEIANPEVDGVSDNRFKEFNIPNSAVFNNSRTESTSQLVGKLHANIQL
82-029362      KTNDNSKDGIVVVEIANPEVDGVSDNRFKEFNIPNSAVFNNSRTESTSQLVGKLHANIQL
6644           KTNDNSKDGIVVVEIANPEVDGVSDNRFKEFNIPNSAVFNNSRTESTSQLVGKLHANIQL
HD183          KTNDNSKDGIVVVEIANPEVDGVSDNRFKEFNIPNSAVFNNSRTESTSQLVGKLHANIQL
HMC46          KTNDNSKDGIVVVEIANPEVDGVSDNRFKEFNIPNSAVFNNSRTESTSQLVGKLHANIQL
HMC56          KTNDNSKDGIVVVEIANPEVDGVSDNRFKEFNIPNSAVFNNSRTESTSQLVGKLHANIQL
               ************************************************************

35000HP        QKEAKLILNQVTGDHESNIQGALEVAGKKADLIIVNPNGITLNGVKTINTDRFVVSTSDI
NZS1           QKEAKLILNQVTGDHESNIQGALEVAGKKADLIIVNPNGITLNGVKTINTDRFVVSTSDI
NZS2           QKEAKLILNQVTGDHESNIQGALEVAGKKADLIIVNPNGITLNGVKTINTDRFVVSTSDI
NZS3           QKEAKLILNQVTGDHESNIQGALEVAGKKADLIIVNPNGITLNGVKTINTDRFVVSTSDI
NZS4           QKEAKLILNQVTGDHESNIQGALEVAGKKADLIIVNPNGITLNGVKTINTDRFVVSTSDI
NZV1           QKEAKLILNQVTGDHESNIQGALEVAGKKADLIIVNPNGITLNGVKTINTDRFVVSTSDI
82-029362      QKEAKLILNQVTGDHESNIQGALEVAGKKADLIIVNPNGITLNGVKTINTDRFVVSTSDI
6644           QKEAKLILNQVTGDHESNIQGALEVAGKKADLIIVNPNGITLNGVKTINTDRFVVSTSDI
HD183          QKEAKLILNQVTGDHESNIQGALEVAGKKADLIIVNPNGITLNGVKTINTDRFVVSTSDI
HMC46          QKEAKLILNQVTGDHESNIQGALEVAGKKADLIIVNPNGITLNGVKTINTDRFVVSTSDI
HMC56          QKEAKLILNQVTGDHESNIQGALEVAGKKADLIIVNPNGITLNGVKTINTDRFVVSTSDI
               ************************************************************

35000HP        IPHRENGLLSVRNGKVTIDKGGVATNGLSHFEVVARNIDQKGKITVAKTENQKSVNPANI
NZS1           IPHRENGLLSVRNGKVTIDKGGVATNGLSHFEVVARNIDQKGKITVAKTENQKSVNPANI
NZS2           IPHRENGLLSVRNGKVTIDKGGVATNGLSHFEVVARNIDQKGKITVAKTENQKSVNPANI
NZS3           IPHRENGLLSVRNGKVTIDKGGVATNGLSHFEVVARNIDQKGKITVAKTENQKSVNPANI
NZS4           IPHRENGLLSVRNGKVTIDKGGVATNGLSHFEVVARNIDQKGKITVAKTENQKSVNPANI
NZV1           IPHRENGLLSVRNGKVTIDKGGVATNGLSHFEVVARNIDQKGKITVAKTENQKSVNPANI
82-029362      IPHRENGLLSVRNGKVTIDKGGVATNGLSHFEVVARNIDQKGKITVAKTENQKSVNPANI
6644           IPHRENGLLSVRNGKVTIDKGGVATNGLSHFEVVARNIDQKGKITVAKTENQKSVNPANI
```

```
HD183         IPHRENGLLSVRNGKVTIDKGGVATNGLSHFEVVARNIDQKGKITVAKTENQKSVNPANI
HMC46         IPHRENGLLSVRNGKVTIDKGGVATNGLSHFEVVARNIDQKGKITVAKTENQKSVNPANI
HMC56         IPHRENGLLSVRNGKVTIDKGGVATNGLSHFEVVARNIDQKGKITVAKTENQKSVNPANI
              ************************************************************

35000HP       TFAAGSLNYNLKTREATPISSGTSRTSDTPAISADSAGSMYGSNIKFVVTDKGAGVKHKG
NZS1          TFAAGSLNYNLKTREATPISSGTSRTSDTPAISADSAGSMYGSNIKFVVTDKGAGVKHKG
NZS2          TFAAGSLNYNLKTREATPISSGTSRTSDTPAISADSAGSMYGSNIKFVVTDKGAGVKHKG
NZS3          TFAAGSLNYNLKTREATPISSGTSRTSDTPAISADSAGSMYGSNIKFVVTDKGAGVKHKG
NZS4          TFAAGSLNYNLKTREATPISSGTSRTSDTPAISADSAGSMYGSNIKFVVTDKGAGVKHKG
NZV1          TFAAGSLNYNLKTREATPISSGTSRTSDTPAISADSAGSMYGSNIKFVVTDKGAGVKHKG
82-029362     TFAAGSLNYNLKTREATPISSGTSRTSDTPAISADSAGSMYGSNIKFVVTDKGAGVKHKG
6644          TFAAGSLNYNLKTREATPISSGTSRTSDTPAISADSAGSMYGSNIKFVVTDKGAGVKHKG
HD183         TFAAGSLNYNLKTREATPISSGTSRTSDTPAISADSAGSMYGSNIKFVVTDKGAGVKHKG
HMC46         TFAAGSLNYNLKTREATPISSGTSRTSDTPAISADSAGSMYGSNIKFVVTDKGAGVKHKG
HMC56         TFAAGSLNYNLKTREATPISSGTSRTSDTPAISADSAGSMYGSNIKFVVTDKGAGVKHKG
              ************************************************************

35000HP       IIFSENDINIKMDGGNASLKELYAKKDIDILAKDIELTEKGQLQANNKIILNSTGKINLR
NZS1          IIFSENDINIKMDGGNASLKELYAKKDIDILAKDIELTEKGQLQANNKIILNSTGKINLR
NZS2          IIFSENDINIKMDGGNASLKELYAKKDIDILAKDIELTEKGQLQANNKIILNSTGKINLR
NZS3          IIFSENDINIKMDGGNASLKELYAKKDIDILAKDIELTEKGQLQANNKIILNSTGKINLR
NZS4          IIFSENDINIKMDGGNASLKELYAKKDIDILAKDIELTEKGQLQANNKIILNSTGKINLR
NZV1          IIFSENDINIKMDGGNASLKELYAKKDIDILAKDIELTEKGQLQANNKIILNSTGKINLR
82-029362     IIFSENDINIKMDGGNASLKELYAKKDIDILAKDIELTEKGQLQANNKIILNSTGKINLR
6644          IIFSENDINIKMDGGNASLKELYAKKDIDILAKDIELTEKGQLQANNKIILNSTGKINLR
HD183         IIFSENDINIKMDGGNASLKELYAKKDIDILAKDIELTEKGQLQANNKIILNSTGKINLR
HMC46         IIFSENDINIKMDGGNASLKELYAKKDIDILAKDIELTEKGQLQANNKIILNSTGKINLR
HMC56         IIFSENDINIKMDGGNASLKELYAKKDIDILAKDIELTEKGQLQANNKIILNSTGKINLR
              ************************************************************

35000HP       NASEVSADNVNVKSENLALENASMSANSLDVIVTKIEVNRSSKVSAGTANIKASNITLDG
NZS1          NASEVSADNVNVKSENLALENASMSANSLDVIVTKIEVNRSSKVSAGTANIKASNITLDG
NZS2          NASEVSADNVNVKSENLALENASMSANSLDVIVTKIEVNRSSKVSAGTANIKASNITLDG
NZS3          NASEVSADNVNVKSENLALENASMSANSLDVIVTKIEVNRSSKVSAGTANIKASNITLDG
NZS4          NASEVSADNVNVKSENLALENASMSANSLDVIVTKIEVNRSSKVSAGTANIKASNITLDG
NZV1          NASEVSADNVNVKSENLALENASMSANSLDVIVTKIEVNRSSKVSAGTANIKASNITLDG
82-029362     NASEVSADNVNVKSENLALENASMSANSLDVIVTKIEVNRSSKVSAGTANIKASNITLDG
6644          NASEVSADNVNVKSENLALENASMSANSLDVIVTKIEVNRSSKVSAGTANIKASNITLDG
HD183         NASEVSADNVNVKSENLALENASMSANSLDVIVTKIEVNRSSKVSAGTANIKASNITLDG
HMC46         NASEVSADNVNVKSENLALENASMSANSLDVIVTKIEVNRSSKVSAGTANIKASNITLDG
HMC56         NASEVSADNVNVKSENLALENASMSANSLDVIVTKIEVNRSSKVSAGTANIKASNITLDG
              ************************************************************

35000HP       SSVVANKITLNVTNNATLNNQSKLSAKDMELNVTHNITLNNTSKLSAQKANIKTENLTLN
NZS1          SSVVANKITLNVTNNATLNNQSKLSAKDMELNVTHNITLNNTSKLSAQKANIKTENLTLN
NZS2          SSVVANKITLNVTNNATLNNQSKLSAKDMELNVTHNITLNNTSKLSAQKANIKTENLTLN
NZS3          SSVVANKITLNVTNNATLNNQSKLSAKDMELNVTHNITLNNTSKLSAQKANIKTENLTLN
NZS4          SSVVANKITLNVTNNATLNNQSKLSAKDMELNVTHNITLNNTSKLSAQKANIKTENLTLN
NZV1          SSVVANKITLNVTNNATLNNQSKLSAKDMELNVTHNITLNNTSKLSAQKANIKTENLTLN
82-029362     SSVVANKITLNVTNNATLNNQSKLSAKDMELNVTHNITLNNTSKLSAQKANIKTENLTLN
6644          SSVVANKITLNVTNNATLNNQSKLSAKDMELNVTHNITLNNTSKLSAQKANIKTENLTLN
HD183         SSVVANKITLNVTNNATLNNQSKLSAKDMELNVTHNITLNNTSKLSAQKANIKTENLTLN
HMC46         SSVVANKITLNVTNNATLNNQSKLSAKDMELNVTHNITLNNTSKLSAQKANIKTENLTLN
HMC56         SSVVANKITLNVTNNATLNNQSKLSAKDMELNVTHNITLNNTSKLSAQKANIKTENLTLN
              ************************************************************

35000HP       GEASLVAEKLDINAIDKITNNGTIAGLTANITTKALENRDNALILAHQNLNFTVNGSHYV
NZS1          GEASLVAEKLDINAIDKITNNGTIAGLTANITTKALENRDNALILAHQNLNFTVNGSHYV
NZS2          GEASLVAEKLDINAIDKITNNGTIAGLTANITTKALENRDNALILAHQNLNFTVNGSHYV
NZS3          GEASLVAEKLDINAIDKITNNGTIAGLTANITTKALENRDNALILAHQNLNFTVNGSHYV
NZS4          GEASLVAEKLDINAIDKITNNGTIAGLTANITTKALENRDNALILAHQNLNFTVNGSHYV
NZV1          GEASLVAEKLDINAIDKITNNGTIAGLTANITTKALENRDNALILAHQNLNFTVNGSHYV
82-029362     GEASLVAEKLDINAIDKITNNGTIAGLTANITTKALENRDNALILAHQNLNFTVNGSHYV
6644          GEASLVAEKLDINAIDKITNNGTIAGLTANITTKALENRDNALILAHQNLNFTVNGSHYV
HD183         GEASLVAEKLDINAIDKITNNGTIAGLTANITTKALENRDNALILAHQNLNFTVNGSHYV
HMC46         GEASLVAEKLDINAIDKITNNGTIAGLTANITTKALENRDNALILAHQNLNFTVNGSHYV
HMC56         GEASLVAEKLDINAIDKITNNGTIAGLTANITTKALENRDNALILAHQNLNFTVNGSHYV
              ************************************************************

35000HP       NKGDIVSKDKAIVTFSNNSDFTSNGSKLVDAQNNLTVNVNNFNITQGSEIILHGNVTLNA
NZS1          NKGDIVSKDKAIVTFSNNSDFTSNGSKLVDAQNNLTVNVNNFNITQGSEIILHGNVTLNA
NZS2          NKGDIVSKDKAIVTFSNNSDFTSNGSKLVDAQNNLTVNVNNFNITQGSEIILHGNVTLNA
```

```
NZS3         NKGDIVSKDKAIVTFSNNSDFTSNGSKLVDAQNNLTVNVNNFNITQGSEIILHGNVTLNA
NZS4         NKGDIVSKDKAIVTFSNNSDFTSNGSKLVDAQNNLTVNVNNFNITQGSEIILHGNVTLNA
NZV1         NKGDIVSKDKAIVTFSNNSDFTSNGSKLVDAQNNLTVNVNNFNITQGSEIILHGNVTLNA
82-029362    NKGDIVSKDKAIVTFSNNSDFTSNGSKLVDAQNNLTVNVNNFNITQGSEIILHGNVTLNA
6644         NKGDIVSKDKAIVTFSNNSDFTSNGSKLVDAQNNLTVNVNNFNITQGSEIILHGNVTLNA
HD183        NKGDIVSKDKAIVTFSNNSDFTSNGSKLVDAQNNLTVNVNNFNITQGSEIILHGNVTLNA
HMC46        NKGDIVSKDKAIVTFSNNSDFTSNGSKLVDAQNNLTVNVNNFNITQGSEIILHGNVTLNA
HMC56        NKGDIVSKDKAIVTFSNNSDFTSNGSKLVDAQNNLTVNVNNFNITQGSEIILHGNVTLNA
             ************************************************************

35000HP      KGNFTNSGNLTTMKELNISNIESFINAGNLTTGKNLEVHSNTTVKNDGKLVSIENLNISS
NZS1         KGNFTNSGNLTTMKELNISNIESFINAGNLTTGKNLEVHSNTTVKNDGKLVSIENLNISS
NZS2         KGNFTNSGNLTTMKELNISNIESFINAGNLTTGKNLEVHSNTTVKNDGKLVSIENLNISS
NZS3         KGNFTNSGNLTTMKELNISNIESFINAGNLTTGKNLEVHSNTTVKNDGKLVSIENLNISS
NZS4         KGNFTNSGNLTTMKELNISNIESFINAGNLTTGKNLEVHSNTTVKNDGKLVSIENLNISS
NZV1         KGNFTNSGNLTTMKELNISNIESFINAGNLTTGKNLEVHSNTTVKNDGKLVSIENLNISS
82-029362    KGNFTNSGNLTTMKELNISNIESFINAGNLTTGKNLEVHSNTTVKNDGKLVSIENLNISS
6644         KGNFTNSGNLTTMKELNISNIESFINAGNLTTGKNLEVHSNTTVKNDGKLVSIENLNISS
HD183        KGNFTNSGNLTTMKELNISNIESFINAGNLTTGKNLEVHSNTTVKNDGKLVSIENLNISS
HMC46        KGNFTNSGNLTTMKELNISNIESFINAGNLTTGKNLEVHSNTTVKNDGKLVSIENLNISS
HMC56        KGNFTNSGNLTTMKELNISNIESFINAGNLTTGKNLEVHSNTTVKNDGKLVSIENLNISS
             ************************************************************

35000HP      KTDFTNNGTLLGLEALKIASGGNFTNASNGSLASNKSLDIYGNNFTNNGTIESVKSLNIT
NZS1         KTDFTNNGTLLGLEALKIASGGNFTNASNGSLASNKSLDIYGNNFTNNGTIESVKSLNIT
NZS2         KTDFTNNGTLLGLEALKIASGGNFTNASNGSLASNKSLDIYGNNFTNNGTIESVKSLNIT
NZS3         KTDFTNNGTLLGLEALKIASGGNFTNASNGSLASNKSLDIYGNNFTNNGTIESVKSLNIT
NZS4         KTDFTNNGTLLGLEALKIASGGNFTNASNGSLASNKSLDIYGNNFTNNGTIESVKSLNIT
NZV1         KTDFTNNGTLLGLEALKIASGGNFTNASNGSLASNKSLDIYGNNFTNNGTIESVKSLNIT
82-029362    KTDFTNNGTLLGLEALKIASGGNFTNASNGSLASNKSLDIYGNNFTNNGTIESVKSLNIT
6644         KTDFTNNGTLLGLEALKIASGGNFTNASNGSLASNKSLDIYGNNFTNNGTIESVKSLNIT
HD183        KTDFTNNGTLLGLEALKIASGGNFTNASNGSLASNKSLDIYGNNFTNNGTIESVKSLNIT
HMC46        KTDFTNNGTLLGLEALKIASGGNFTNASNGSLASNKSLDIYGNNFTNNGTIESVKSLNIT
HMC56        KTDFTNNGTLLGLEALKIASGGNFTNASNGSLASNKSLDIYGNNFTNNGTIESVKSLNIT
             ************************************************************

35000HP      NNYTFINNATIKSYGVLNITSQGNFTNDSNGTVMSHDLLNITSQANIINKNLLAGGQGLN
NZS1         NNYTFINNATIKSYGVLNITSQGNFTNDSNGTVMSHDLLNITSQANIINKNLLAGGQGLN
NZS2         NNYTFINNATIKSYGVLNITSQGNFTNDSNGTVMSHDLLNITSQANIINKNLLAGGQGLN
NZS3         NNYTFINNATIKSYGVLNITSQGNFTNDSNGTVMSHDLLNITSQANIINKNLLAGGQGLN
NZS4         NNYTFINNATIKSYGVLNITSQGNFTNDSNGTVMSHDLLNITSQANIINKNLLAGGQGLN
NZV1         NNYTFINNATIKSYGVLNITSQGNFTNDSNGTVMSHDLLNITSQANIINKNLLAGGQGLN
82-029362    NNYTFINNATIKSYGVLNITSQGNFTNDSNGTVMSHDLLNITSQANIINKNLLAGGQGLN
6644         NNYTFINNATIKSYGVLNITSQGNFTNDSNGTVMSHDLLNITSQANIINKNLLAGGQGLN
HD183        NNYTFINNATIKSYGVLNITSQGNFTNDSNGTVMSHDLLNITSQANIINKNLLAGGQGLN
HMC46        NNYTFINNATIKSYGVLNITSQGNFTNDSNGTVMSHDLLNITSQANIINKNLLAGGQGLN
HMC56        NNYTFINNATIKSYGVLNITSQGNFTNDSNGTVMSHDLLNITSQANIINKNLLAGGQGLN
             ************************************************************

35000HP      LTAKGNITNDSNSTAIAVLHSNNDINLNANNKVYNIGEIYSQAGNISVEAKLLHNDVKLS
NZS1         LTAKGNITNDSNSTAIAVLHSNNDINLNANNKVYNIGEIYSQAGNISVEAKLLHNDVKLS
NZS2         LTAKGNITNDSNSTAIAVLHSNNDINLNANNKVYNIGEIYSQAGNISVEAKLLHNDVKLS
NZS3         LTAKGNITNDSNSTAIAVLHSNNDINLNANNKVYNIGEIYSQAGNISVEAKLLHNDVKLS
NZS4         LTAKGNITNDSNSTAIAVLHSNNDINLNANNKVYNIGEIYSQAGNISVEAKLLHNDVKLS
NZV1         LTAKGNITNDSNSTAIAVLHSNNDINLNANNKVYNIGEIYSQAGNISVEAKLLHNDVKLS
82-029362    LTAKGNITNDSNSTAIAVLHSNNDINLNANNKVYNIGEIYSQAGNISVEAKLLHNDVKLS
6644         LTAKGNITNDSNSTAIAVLHSNNDINLNANNKVYNIGEIYSQAGNISVEAKLLHNDVKLS
HD183        LTAKGNITNDSNSTAIAVLHSNNDINLNANNKVYNIGEIYSQAGNISVEAKLLHNDVKLS
HMC46        LTAKGNITNDSNSTAIAVLHSNNDINLNANNKVYNIGEIYSQAGNISVEAKLLHNDVKLS
HMC56        LTAKGNITNDSNSTAIAVLHSNNDINLNANNKVYNIGEIYSQAGNISVEAKLLHNDVKLS
             ************************************************************

35000HP      GNITTTTKSGNATVKTNSIGGGLHDANSIRVGELTLNGKFADLDNQLKVALRGKIYAGSN
NZS1         GNITTTTKSGNATVKTNSIGGGLHDANSIRVGELTLNGKFADLDNQLKVALRGKIYAGSN
NZS2         GNITTTTKSGNATVKTNSIGGGLHDANSIRVGELTLNGKFADLDNQLKVALRGKIYAGSN
NZS3         GNITTTTKSGNATVKTNSIGGGLHDANSIRVGELTLNGKFADLDNQLKVALRGKIYAGSN
NZS4         GNITTTTKSGNATVKTNSIGGGLHDANSIRVGELTLNGKFADLDNQLKVALRGKIYAGSN
NZV1         GNITTTTKSGNATVKTNSIGGGLHDANSIRVGELTLNGKFADLDNQLKVALRGKIYAGSN
82-029362    GNITTTTKSGNATVKTNSIGGGLHDANSIRVGELTLNGKFADLDNQLKVALRGKIYAGSN
6644         GNITTTTKSGNATVKTNSIGGGLHDANSIRVGELTLNGKFADLDNQLKVALRGKIYAGSN
HD183        GNITTTTKSGNATVKTNSIGGGLHDANSIRVGELTLNGKFADLDNQLKVALRGKIYAGSN
HMC46        GNITTTTKSGNATVKTNSIGGGLHDANSIRVGELTLNGKFADLDNQLKVALRGKIYAGSN
HMC56        GNITTTTKSGNATVKTNSIGGGLHDANSIRVGELTLNGKFADLDNQLKVALRGKIYAGSN
```

```
                ************************************************************

35000HP         LTFKAKEGEKEQKSTAQAKIINRGTINVKNKLEYGSNVDVENNMRSMQVNLYEKIFNGDN
NZS1            LTFKAKEGEKEQKSTAQAKIINRGTINVKNKLEYGSNVDVENNMHSMQVNLYEKIFNGDN
NZS2            LTFKAKEGEKEQKSTAQAKIINRGTINVKNKLEYGSNVDVENNMHSMQVNLYEKIFNGDN
NZS3            LTFKAKEGEKEQKSTAQAKIINRGTINVKNKLEYGSNVDVENNMHSMQVNLYEKIFNGDN
NZS4            LTFKAKEGEKEQKSTAQAKIINRGTINVKNKLEYGSNVDVENNMHSMQVNLYEKIFNGDN
NZV1            LTFKAKEGEKEQKSTAQAKIINRGTINVKNKLEYGSNVDVENNMHSMQVNLYEKIFNGDN
82-029362       LTFKAKEGEKEQKSTAQAKIINRGTINVKNKLEYGSNVDVENNMHSMQVNLYEKIFNGDN
6644            LTFKAKEGEKEQKSTAQAKIINRGTINVKNKLEYGSNVDVENNMHSMQVNLYEKIFNGDN
HD183           LTFKAKEGEKEQKSTAQAKIINRGTINVKNKLEYGSNVDVENNMRSMQVNLYEKIFNGDN
HMC46           LTFKAKEGEKEQKSTAQAKIINRGTINVKNKLEYGSNVDVENNMHSMQVNLYEKIFNGDN
HMC56           LTFKAKEGEKEQKSTAQAKIINRGTINVKNKLEYGSNVDVENNMHSMQVNLYEKIFNGDN
                ********************************************:***************

35000HP         PITLTLKNGVTFAKDFSNRRRRASNDGEGTNKKTFDNVAHLIEEAFSGYSNGNDHRASDD
NZS1            PITLTLKNGVTFAKDFSGRR-RASNDGGGTNKKTFDNVAHLIEEAFSGYSNGNDHGASGD
NZS2            PITLTLKNGVTFAKDFSGRR-RASNDGGGTNKKTFDNVAHLIEEAFSGYSNGNDHGASGD
NZS3            PITLTLKNGVTFAKDFSGRR-RASNDGGGTNKKTFDNVAHLIEEAFSGYSNGNDHGASGD
NZS4            PITLTLKNGVTFAKDFSGRR-RASNDGGGTNKKTFDNVAHLIEEAFSGYSNGNDHGASGD
NZV1            PITLTLKNGVTFAKDFSGRR-RASNDGGGTNKKTFDNVAHLIEEAFSGYSNGNDHGASGD
82-029362       PITLTLKNGVTFAKDFSKRRRRASGDSSGTNEKTFDNVAHLIEEAFSGYSDGNDHGASDG
6644            PITLTLKNGVTFAKDFSKRRRRASGDSSGTNEKTFDNVAHLIEEAFSGYSDGNDHGASDG
HD183           PITLTLKNGVTFAKDFSNRRRRASNDGEGTNKKTFDNVAHLIEEAFSGYSNGNDHRASDD
HMC46           PITLTLKNGVTFAKDFSKRRRRASGDSSGTNEKTFDNVAHLIEEAFSGYSDGNDHGASDG
HMC56           PITLTLKNGVTFAKDFSKRRRRASGDSSGTNEKTFDNVAHLIEEAFSGYSDGNDHGASDG
                ***************** ** *** *. ***:******************:**** **

35000HP         GHVKSPYYLLVLAQAVNNTEGENYLKTALQHIFGPNWNDLTTTNNDTTINDKWNQLKLKW
NZS1            GHVKSPYYLLVLAQAVNNTEGENYLKTALQHIFGPNWNDLTTTNNDTTINDKWNQLKLKW
NZS2            GHVKSPYYLLVLAQAVNNTEGENYLKTALQHIFGPNWNDLTTTNNDTTINDKWNQLKLKW
NZS3            GHVKSPYYLLVLAQAVNNTEGENYLKTALQHIFGPNWNDLTTTNNDTTINDKWNQLKLKW
NZS4            GHVKSPYYLLVLAQAVNNTEGENYLKTALQHIFGPNWNDLTTTNNDTTINDKWNQLKLKW
NZV1            GHVKSPYYLLVLAQAVNNTEGENYLKTALQHIFGPNWNDLTTTNNDTTINDKWNQLKLKW
82-029362       GHVKSPYYLLVLAQAVNNTEGENYLKTALQHIFGPNWNDLTTTNNGTTINDKWNQLKLKW
6644            GHVKSPYYLLVLAQAVNNTEGENYLKTALQHIFGPNWNDLTTTNNGTTINDKWNQLKLKW
HD183           GHVKSPYYLLVLAQAVNNTEGENYLKTALQHIFGPNWNDLTTTNNDTTINDKWNQLKLKW
HMC46           GHVKSPYYLLVLAQAVNNTEGENYLKTALQHIFGPNWNDLTTTNNGTTINDKWNQLKLKW
HMC56           GHVKSPYYLLVLAQAVNNTEGENYLKTALQHIFGPNWNDLTTTNNGTTINDKWNQLKLKW
                ********************************************* **************

35000HP         EKFKNNGENNHSINLNIYPADEGVEKAKIFAGVLRNGTNGVEDKVYQELNDKAKKEYEDK
NZS1            EKFKNNGENNHSINLNIYPADEGVEKAKIFAGVLRNGTNGVEDKVYQELNDKAKKEYEDK
NZS2            EKFKNNGENNHSINLNIYPADEGVEKAKIFAGVLRNGTNGVEDKVYQELNDKAKKEYEDK
NZS3            EKFKNNGENNHSINLNIYPADEGVEKAKIFAGVLRNGTNGVEDKVYQELNDKAKKEYEDK
NZS4            EKFKNNGENNHSINLNIYPADEGVEKAKIFAGVLRNGTNGVEDKVYQELNDKAKKEYEDK
NZV1            EKFKNNGENNHSINLNIYPADEGVEKAKIFAGVLRNGTNGVEDKVYQELNDKAKKEYEDK
82-029362       EKFKNNGENNHSINLNIYPADEGVEKAKIFAGVLRNGTNGVEDKVYQELNDKAKKEYEDK
6644            EKFKNNGENNHSINLNIYPADEGVEKAKIFAGVLRNGTNGVEDKVYQELNDKAKKEYEDK
HD183           EKFKNNGENNHSINLNIYPADEGVEKAKIFAGVLRNGTNGVEDKVYQELNDKAKKEYEDK
HMC46           EKFKNNGENNHSINLNIYPADEGVEKAKIFAGVLRNGTNGVEDKVYQELNDKAKKEYEDK
HMC56           EKFKNNGENNHSINLNIYPADEGVEKAKIFAGVLRNGTNGVEDKVYQELNDKAKKEYEDK
                ************************************************************

35000HP         FAKKFQGRFKSRFQNGEFDWAGDWAKEGNESYGSKETEEKYNGIKKEHTVNIGKHEIKVP
NZS1            FAKKFQGRFKSRFQNGEFDWAGDWAKEGNESYGSKETEEKYNGIKKEHTVNIGKHEIKVP
NZS2            FAKKFQGRFKSRFQNGEFDWAGDWAKEGNESYGSKETEEKYNGIKKEHTVNIGKHEIKVP
NZS3            FAKKFQGRFKSRFQNGEFDWAGDWAKEGNESYGSKETEEKYNGIKKEHTVNIGKHEIKVP
NZS4            FAKKFQGRFKSRFQNGEFDWAGDWAKEGNESYGSKETEEKYNGIKKEHTVNIGKHEIKVP
NZV1            FAKKFQGRFKSRFQNGEFDWAGDWAKEGNESYGSKETEEKYNGIKKEHTVNIGKHEIKVP
82-029362       FAKKFQGRFKSRFQNGEFDWAGDWAKEGNESYGSKETEEKYNGIKKEHTVNIGKHEIKVP
6644            FAKKFQGRFKSRFQNGEFDWAGDWAKEGNESYGSKETEEKYNGIKKEHTVNIGKHEIKVP
HD183           FAKKFQGRFKSRFQNGEFDWAGDWAKEGNESYGSKETEEKYNGIKKEHTVNIGKHEIKVP
HMC46           FAKKFQGRFKSRFQNGEFDWAGDWAKEGNESYGSKETEEKYNGIKKEHTVNIGKHEIKVP
HMC56           FAKKFQGRFKSRFQNGEFDWAGDWAKEGNESYGSKETEEKYNGIKKEHTVNIGKHEIKVP
                ************************************************************

35000HP         TVSFENLNNINHQQDKSDGIDKSIISELLAQPIYVAKADVPDVDPRVAQNDKAVDEDGLY
NZS1            TVSFENLNNINHQQDKSDGIDKSIISELLAQPIYVAKADVPDVDPRVAQNDKAVDEDGLY
NZS2            TVSFENLNNINHQQDKSDGIDKSIISELLAQPIYVAKADVPDVDPRVAQNDKAVDEDGLY
NZS3            TVSFENLNNINHQQDKSDGIDKSIISELLAQPIYVAKADVPDVDPRVAQNDKAVDEDGLY
NZS4            TVSFENLNNINHQQDKSDGIDKSIISELLAQPIYVAKADVPDVDPRVAQNDKAVDEDGLY
NZV1            TVSFENLNNINHQQDKSDGIDKSIISELLAQPIYVAKADVPDVDPRVAQNDKAVDEDGLY
```

```
82-029362      TVSFENLNNINHQQDKSDGIDKSIISELLAQPIYVAKADVPDVDPRVAQNDKAVDEDGLY
6644           TVSFENLNNINHQQDKSDGIDKSIISELLAQPIYVAKADVPDVDPRVAQNDKAVDEDGLY
HD183          TVSFENLNNINHQQDKSDGIDKSIISELLAQPIYVAKADVPDVDPRVAQNDKAVDEDGLY
HMC46          TVSFENLNNINHQQDKSDGIDKSIISELLAQPIYVAKADVPDVDPRVAQNDKAVDEDGLY
HMC56          TVSFENLNNINHQQDKSDGIDKSIISELLAQPIYVAKADVPDVDPRVAQNDKAVDEDGLY
               ************************************************************

35000HP        RTRLSYINQNNYLGAKYFFNQLDTEDDKLKGIKRIGDNYFEHQLITRLIEKVADNHLTLK
NZS1           RTRLSYINQNNYLGAKYFFNQLDTEDDKLKGIKRIGDNYFEHQLITRLIEKVADNHLTLK
NZS2           RTRLSYINQNNYLGAKYFFNQLDTEDDKLKGIKRIGDNYFEHQLITRLIEKVADNHLTLK
NZS3           RTRLSYINQNNYLGAKYFFNQLDTEDDKLKGIKRIGDNYFEHQLITRLIEKVADNHLTLK
NZS4           RTRLSYINQNNYLGAKYFFNQLDTEDDKLKGIKRIGDNYFEHQLITRLIEKVADNHLTLK
NZV1           RTRLSYINQNNYLGAKYFFNQLDTEDDKLKGIKRIGDNYFEHQLITRLIEKVADNHLTLK
82-029362      RTRLSYINQNNYLGAKYFFNQLDTEDDKLKGIKRIGDNYFEYQLITRLIEKVADNHLTLK
6644           RTRLSYINQNNYLGAKYFFNQLDTEDDKLKGIKRIGDNYFEYQLITRLIEKVADNHLTLK
HD183          RTRLSYINQNNYLGAKYFFNQLDTEDDKLKGIKRIGDNYFEHQLITRLIEKVADNHLTLK
HMC46          RTRLSYINQNNYLGAKYFFNQLDTEDDKLKGIKRIGDNYFEYQLITRLIEKVADNHLTLK
HMC56          RTRLSYINQNNYLGAKYFFNQLDTEDDKLKGIKRIGDNYFEYQLITRLIEKVADNHLTLK
               *****************************************:******************

35000HP        HGLHDIALVKKLIDSASIQAKDLNLKVGEALTKEQKDNLKEDIVWYVKTEVNGQEVLVPQ
NZS1           HGLHDIALVKKLIDSASIQAKDLNLKVGEALTKEQKDNLKEDIVWYVKTEVNGQEVLVPQ
NZS2           HGLHDIALVKKLIDSASIQAKDLNLKVGEALTKEQKDNLKEDIVWYVKTEVNGQEVLVPQ
NZS3           HGLHDIALVKKLIDSASIQAKDLNLKVGEALTKEQKDNLKEDIVWYVKTEVNGQEVLVPQ
NZS4           HGLHDIALVKKLIDSASIQAKDLNLKVGEALTKEQKDNLKEDIVWYVKTEVNGQEVLVPQ
NZV1           HGLHDIALVKKLIDSASIQAKDLNLKVGEALTKEQKDNLKEDIVWYVKTEVNGQEVLVPQ
82-029362      HGLHDIALVKKLIDSASIQAKDLNLKVGEALTKEQKDNLKEDIVWYVKTEVNGQEVLVPQ
6644           HGLHDIALVKKLIDSASIQAKDLNLKVGEALTKEQKDNLKEDIVWYVKTEVNGQEVLVPQ
HD183          HGLHDIALVKKLIDSASIQAKDLNLKVGEALTKEQKDNLKEDIVWYVKTEVNGQEVLVPQ
HMC46          HGLHDIALVKKLIDSASIQAKDLNLKVGEALTKEQKDNLKEDIVWYVKTEVNGQEVLVPQ
HMC56          HGLHDIALVKKLIDSASIQAKDLNLKVGEALTKEQKDNLKEDIVWYVKTEVNGQEVLVPQ
               ************************************************************

35000HP        VYLAKQTIEEVEKQRGVGTGQIRAGIIDVKVDDVRNTGTIAGYAVGLEAKNKLKNTGDIL
NZS1           VYLAKQTIEEVEKQRGVGTGQIRAGIIDVKVDDVRNTGTIAGYAVGLEAKNKLKNTGDIL
NZS2           VYLAKQTIEEVEKQRGVGTGQIRAGIIDVKVDDVRNTGTIAGYAVGLEAKNKLKNTGDIL
NZS3           VYLAKQTIEEVEKQRGVGTGQIRAGIIDVKVDDVRNTGTIAGYAVGLEAKNKLKNTGDIL
NZS4           VYLAKQTIEEVEKQRGVGTGQIRAGIIDVKVDDVRNTGTIAGYAVGLEAKNKLKNTGDIL
NZV1           VYLAKQTIEEVEKQRGVGTGQIRAGIIDVKVDDVRNTGTIAGYAVGLEAKNKLKNTGDIL
82-029362      VYLAKQTIEEVEKQRGVGTGQIRAGIIDVKVDDVRNTGTIAGYAVGLEAKNKLKNTGDIL
6644           VYLAKQTIEEVEKQRGVGTGQIRAGIIDVKVDDVRNTGTIAGYAVGLEAKNKLKNTGDIL
HD183          VYLAKQTIEEVEKQRGVGTGQIRAGIIDVKVDDVRNTGTIAGYAVGLEAKNKLKNTGDIL
HMC46          VYLAKQTIEEVEKQRGVGTGQIRAGIIDVKVDDVRNTGTIAGYAVGLEAKNKLKNTGDIL
HMC56          VYLAKQTIEEVEKQRGVGTGQIRAGIIDVKVDDVRNTGTIAGYAVGLEAKNKLKNTGDIL
               ************************************************************

35000HP        SQRLSKLVGKKGLESTGVTYVDETGATKVRKARIKSEGHIYLETDKDKNVDLTASELKGN
NZS1           SQRLSKLVGKKGLESTGVTYVDETGATKVRKARIKSEGHIYLETDKDKNVDLTASELKGN
NZS2           SQRLSKLVGKKGLESTGVTYVDETGATKVRKARIKSEGHIYLETDKDKNVDLTASELKGN
NZS3           SQRLSKLVGKKGLESTGVTYVDETGATKVRKARIKSEGHIYLETDKDKNVDLTASELKGN
NZS4           SQRLSKLVGKKGLESTGVTYVDETGATKVRKARIKSEGHIYLETDKDKNVDLTASELKGN
NZV1           SQRLSKLVGKKGLESTGVTYVDETGATKVRKARIKSEGHIYLETDKDKNVDLTASELKGN
82-029362      SQRLSKLVGKKGLESTGVTYVDETGATKVRKARIKSEGHIYLETDKDKNVDLTASELKGS
6644           SQRLSKLVGKKGLESTGVTYVDETGATKVRKARIKSEGHIYLETDKDKNVDLTASELKGS
HD183          SQRLSKLVGKKGLESTGVTYVDETGATKVRKARIKSEGHIYLETDKDKNVDLTASELKGN
HMC46          SQRLSKLVGKKGLESTGVTYVDETGATKVRKARIKSEGHIYLETDKDKNVDLTASELKGS
HMC56          SQRLSKLVGKKGLESTGVTYVDETGATKVRKARIKSEGHIYLETDKDKNVDLTASELKGS
               ***********************************************************.

35000HP        TGQIKAKDLNLNDIYETSYKYKYEKLFGKNGGEIGDRVTQTSQAKSVGTDASFDHLHLSL
NZS1           TGQIKAKDLNLNDIYETSYKYKYEKLFGKNGGEIGDRVTQTSQAKSVGTDASFDHLHLSL
NZS2           TGQIKAKDLNLNDIYETSYKYKYEKLFGKNGGEIGDRVTQTSQAKSVGTDASFDHLHLSL
NZS3           TGQIKAKDLNLNDIYETSYKYKYEKLFGKNGGEIGDRVTQTSQAKSVGTDASFDHLHLSL
NZS4           TGQIKAKDLNLNDIYETSYKYKYEKLFGKNGGEIGDRVTQTSQAKSVGTDASFDHLHLSL
NZV1           TGQIKAKDLNLNDIYETSYKYKYEKLFGKNGGEIGDRVTQTSQAKSVGTDASFDHLHLSL
82-029362      TGQIKAKDLNLNDIYETSYKYKYEKLFGKNGGEIGDRVTQTSQAKSVGTDASFDHLHLSL
6644           TGQIKAKDLNLNDIYETSYKYKYEKLFGKNGGEIGDRVTQTSQAKSVGTDASFDHLHLSL
HD183          TGQIKAKDLNLNDIYETSYKYKYEKLFGKNGGEIGDRVTQTSQAKSVGTDASFDHLHLSL
HMC46          TGQIKAKDLNLNDIYETSYKYKYEKLFGKNGGEIGDRVTQTSQAKSVGTDASFDHLHLSL
HMC56          TGQIKAKDLNLNDIYETSYKYKYEKLFGKNGGEIGDRVTQTSQAKSVGTDASFDHLHLSL
               ************************************************************

35000HP        EGDVNQTGSNLKANRTTGVVKGDFNTKAGKDLFHRQIDTVTSGTVYSASASGGGQSAGIS
```

```
NZS1          EGDVNQTGSNLKANRTTGVVKGDFNTKAGKDLFHRQIDTVTSGTVYSASASGGGQSAGIS
NZS2          EGDVNQTGSNLKANRTTGVVKGDFNTKAGKDLFHRQIDTVTSGTVYSASASGGGQSAGIS
NZS3          EGDVNQTGSNLKANRTTGVVKGDFNTKAGKDLFHRQIDTVTSGTVYSASASGGGQSAGIS
NZS4          EGDVNQTGSNLKANRTTGVVKGDFNTKAGKDLFHRQIDTVTSGTVYSASASGGGQSAGIS
NZV1          EGDVNQTGSNLKANRTTGVVKGDFNTKAGKDLFHRQIDTVTSGTVYSASASGGGQSAGIS
82-029362     EGDVNQTGSNLKANRTTGVVKGDFNTKAGKDLFHRQIDTVTSGTVYSASASGGGQSAGIS
6644          EGDVNQTGSNLKANRTTGVVKGDFNTKAGKDLFHRQIDTVTSGTVYSASASGGGQSAGIS
HD183         EGDVNQTGSNLKANRTTGVVKGDFNTKAGKDLFHRQIDTVTSGTVYSASASGGGQSAGIS
HMC46         EGDVNQTGSNLKANRTTGVVKGDFNTKAGKDLFHRQIDTVTSGTVYSASASGGGQSAGIS
HMC56         EGDVNQTGSNLKANRTTGVVKGDFNTKAGKDLFHRQIDTVTSGTVYSASASGGGQSAGIS
              ************************************************************

35000HP       LTDQGVETYTNKTATAGANADVTNFMKRTRETETSLTHRNSEFNALSGELYVMGKADIGG
NZS1          LTDQGVETYTNKTATAGANADVTNFMKRTRETETSLTHRNSEFNALSGELYVMGKADIGG
NZS2          LTDQGVETYTNKTATAGANADVTNFMKRTRETETSLTHRNSEFNALSGELYVMGKADIGG
NZS3          LTDQGVETYTNKTATAGANADVTNFMKRTRETETSLTHRNSEFNALSGELYVMGKADIGG
NZS4          LTDQGVETYTNKTATAGANADVTNFMKRTRETETSLTHRNSEFNALSGELYVMGKADIGG
NZV1          LTDQGVETYTNKTATAGANADVTNFMKRTRETETSLTHRNSEFNALSGELYVMGKADIGG
82-029362     LTDQGVETYTNKTATAGANADVTNFMKRTRETETSLTHRNSEFNALSGELYVMGKADIGG
6644          LTDQGVETYTNKTATAGANADVTNFMKRTRETETSLTHRNSEFNALSGELYVMGKADIGG
HD183         LTDQGVETYTNKTATAGANADVTNFMKRTRETETSLTHRNSEFNALSGELYVMGKADIGG
HMC46         LTDQGVETYTNKTATAGANADVTNFMKRTRETETSLTHRNSEFNALSGELYVMGKADIGG
HMC56         LTDQGVETYTNKTATAGANADVTNFMKRTRETETSLTHRNSEFNALSGELYVMGKADIGG
              ************************************************************

35000HP       VDINRDVEVIKTPEEIAAEQKAAEEAKKAEVKENEASETAAKETEEAENDNVAEKDKTKP
NZS1          VDINRDVEVIKTPEEIAAEQKAAEEAKKAEVKENEASETAAKETEEAENDNVAEKDKTKP
NZS2          VDINRDVEVIKTPEEIAAEQKAAEEAKKAEVKENEASETAAKETEEAENDNVAEKDKTKP
NZS3          VDINRDVEVIKTPEEIAAEQKAAEEAKKAEVKENEASETAAKETEEAENDNVAEKDKTKP
NZS4          VDINRDVEVIKTPEEIAAEQKAAEEAKKAEVKENEASETAAKETEEAENDNVAEKDKTKP
NZV1          VDINRDVEVIKTPEEIAAEQKAAEEAKKAEVKENEASETAAKETEEAENDNVAEKDKTKP
82-029362     VDINRDVEVIKTPEEIAAEQKAAEEAKKAEVKENEASETAAKETEEAENDNVAEKDKTKP
6644          VDINRDVEVIKTPEEIAAEQKAAEEAKKAEVKENEASETAAKETEEAENDNVAEKDKTKP
HD183         VDINRDVEVIKTPEEIAAEQKAAEEAKKAEVKENEASETAAKETEEAENDNVAEKDKTKP
HMC46         VDINRDVEVIKTPEEIAAEQKAAEEAKKAEVKENEASETAAKETEEAENDNVAEKDKTKP
HMC56         VDINRDVEVIKTPEEIAAEQKAAEEAKKAEVKENEASETAAKETEEAENDNVAEKDKTKP
              ************************************************************

35000HP       KFKKLTDEEIAAAFETKGEDFFAAYKAREEEDRKKGFTLSAEQIESTKARDEKETTYYEL
NZS1          KFKKLTDEEIAAAFETKGEDFFAAYKAREEEDRKKGFTLSAEQIESTKARDEKETTYYEL
NZS2          KFKKLTDEEIAAAFETKGEDFFAAYKAREEEDRKKGFTLSAEQIESTKARDEKETTYYEL
NZS3          KFKKLTDEEIAAAFETKGEDFFAAYKAREEEDRKKGFTLSAEQIESTKARDEKETTYYEL
NZS4          KFKKLTDEEIAAAFETKGEDFFAAYKAREEEDRKKGFTLSAEQIESTKARDEKETTYYEL
NZV1          KFKKLTDEEIAAAFETKGEDFFAAYKAREEEDRKKGFTLSAEQIESTKARDEKETTYYEL
82-029362     KFKKLTDEEIAAAFETKGEDFFAAYKAREEEDRKKGFTLSAEQIESTKARDEKETTYYEL
6644          KFKKLTDEEIAAAFETKGEDFFAAYKAREEEDRKKGFTLSAEQIESTKARDEKETTYYEL
HD183         KFKKLTDEEIAAAFETKGEDFFAAYKAREEEDRKKGFTLSAEQIESTKARDEKETTYYEL
HMC46         KFKKLTDEEIAAAFETKGEDFFAAYKAREEEDRKKGFTLSAEQIESTKARDEKETTYYEL
HMC56         KFKKLTDEEIAAAFETKGEDFFAAYKAREEEDRKKGFTLSAEQIESTKARDEKETTYYEL
              ************************************************************

35000HP       KVGVGAEAEAHSAAADAISNKARQIIDTQNGLKQDGTVALQEASDVLNLATGDLAGASAK
NZS1          KVGVGAEAEAHSAAADAISNKARQIIDTQNGLKQDGTVALQEASDVLNLATGDLAGASAK
NZS2          KVGVGAEAEAHSAAADAISNKARQIIDTQNGLKQDGTVALQEASDVLNLATGDLAGASAK
NZS3          KVGVGAEAEAHSAAADAISNKARQIIDTQNGLKQDGTVALQEASDVLNLATGDLAGASAK
NZS4          KVGVGAEAEAHSAAADAISNKARQIIDTQNGLKQDGTVALQEASDVLNLATGDLAGASAK
NZV1          KVGVGAEAEAHSAAADAISNKARQIIDTQNGLKQDGTVALQEASDVLNLATGDLAGASAK
82-029362     KVGVGAEAEAHSAAADAISNKARQIIDTQNGLKQDGTVALQEASDVLNLATGDLAGASAK
6644          KVGVGAEAEAHSAAADAISNKARQIIDTQNGLKQDGTVALQEASDVLNLATGDLAGASAK
HD183         KVGVGAEAEAHSAAADAISNKARQIIDTQNGLKQDGTVALQEASDVLNLATGDLAGASAK
HMC46         KVGVGAEAEAHSAAADAISNKARQIIDTQNGLKQDGTVALQEASDVLNLATGDLAGASAK
HMC56         KVGVGAEAEAHSAAADAISNKARQIIDTQNGLKQDGTVALQEASDVLNLATGDLAGASAK
              ************************************************************

35000HP       LKFELSTIEKKSRGASDGRSILGGRLNLAARGGDITLNNVETTENSHLSLKARDNVNVNS
NZS1          LKFELSTIEKKSRGASDGRSILGGRLNLAARGGDITLNNVETTENSHLSLKARDNVNVNS
NZS2          LKFELSTIEKKSRGASDGRSILGGRLNLAARGGDITLNNVETTENSHLSLKARDNVNVNS
NZS3          LKFELSTIEKKSRGASDGRSILGGRLNLAARGGDITLNNVETTENSHLSLKARDNVNVNS
NZS4          LKFELSTIEKKSRGASDGRSILGGRLNLAARGGDITLNNVETTENSHLSLKARDNVNVNS
NZV1          LKFELSTIEKKSRGASDGRSILGGRLNLAARGGDITLNNVETTENSHLSLKARDNVNVNS
82-029362     LKFELSTIEKKSRGASDGRSILGGRLNLAARGGDITLNNVETTENSHLSLKARDNVNVNS
6644          LKFELSTIEKKSRGASDGRSILGGRLNLAARGGDITLNNVETTENSHLSLKARDNVNVNS
HD183         LKFELSTIEKKSRGASDGRSILGGRLNLAARGGDITLNNVETTENSHLSLKARDNVNVNS
```

```
HMC46        LKFELSTIEKKSRGASDGRSILGGRLNLAARGGDITLNNVETTENSHLSLKARDNVNVNS
HMC56        LKFELSTIEKKSRGASDGRSILGGRLNLAARGGDITLNNVETTENSHLSLKARDNVNVNS
             ************************************************************

35000HP      GVTEQKDESNSQSLKVTAGASSGCGVMAGGCSAGVSAGVSGSYNESNTESTSHTNSLLRG
NZS1         GVTEQKDESNSQSLKVTAGASSGCGVMAGGCSAGVSAGVSGSYNESNTESTSHTNSLLRG
NZS2         GVTEQKDESNSQSLKVTAGASSGCGVMAGGCSAGVSAGVSGSYNESNTESTSHTNSLLRG
NZS3         GVTEQKDESNSQSLKVTAGASSGCGVMAGGCSAGVSAGVSGSYNESNTESTSHTNSLLRG
NZS4         GVTEQKDESNSQSLKVTAGASSGCGVMAGGCSAGVSAGVSGSYNESNTESTSHTNSLLRG
NZV1         GVTEQKDESNSQSLKVTAGASSGCGVMAGGCSAGVSAGVSGSYNESNTESTSHTNSLLRG
82-029362    GVTEQKDESNSQSLKVTAGASSGCGVMAGGCSAGVSAGVSGSYNESNTESTSHTNSLLRG
6644         GVTEQKDESNSQSLKVTAGASSGCGVMAGGCSAGVSAGVSGSYNESNTESTSHTNSLLRG
HD183        GVTEQKDESNSQSLKVTAGASSGCGVMAGGCSAGVSAGVSGSYNESNTESTSHTNSLLRG
HMC46        GVTEQKDESNSQSLKVTAGASSGCGVMAGGCSAGVSAGVSGSYNESNTESTSHTNSLLRG
HMC56        GVTEQKDESNSQSLKVTAGASSGCGVMAGGCSAGVSAGVSGSYNESNTESTSHTNSLLRG
             ************************************************************

35000HP      KSLRVEAGKDFNLISSNVDVDHLHLDVKGDTNVVSKQDSYSRKERGVNYSVSAGVGVSTA
NZS1         KSLRVEAGKDFNLISSNVDVDHLHLDVKGDTNVVSKQDSYSRKERGVNYSVSAGVGVSTA
NZS2         KSLRVEAGKDFNLISSNVDVDHLHLDVKGDTNVVSKQDSYSRKERGVNYSVSAGVGVSTA
NZS3         KSLRVEAGKDFNLISSNVDVDHLHLDVKGDTNVVSKQDSYSRKERGVNYSVSAGVGVSTA
NZS4         KSLRVEAGKDFNLISSNVDVDHLHLDVKGDTNVVSKQDSYSRKERGVNYSVSAGVGVSTA
NZV1         KSLRVEAGKDFNLISSNVDVDHLHLDVKGDTNVVSKQDSYSRKERGVNYSVSAGVGVSTA
82-029362    KSLRVEAGKDFNLISSNVDVDHLHLDVKGDTNVVSKQDSYSRKERGVNYSVSAGVGVSTA
6644         KSLRVEAGKDFNLISSNVDVDHLHLDVKGDTNVVSKQDSYSRKERGVNYSVSAGVGVSTA
HD183        KSLRVEAGKDFNLISSNVDVDHLHLDVKGDTNVVSKQDSYSRKERGVNYSVSAGVGVSTA
HMC46        KSLRVEAGKDFNLISSNVDVDHLHLDVKGDTNVVSKQDSYSRKERGVNYSVSAGVGVSTA
HMC56        KSLRVEAGKDFNLISSNVDVDHLHLDVKGDTNVVSKQDSYSRKERGVNYSVSAGVGVSTA
             ************************************************************

35000HP      GGARPNGSVGLGVSAENENSKIVKQQAGISAKRITGEINNLNLTGGYIENKGNPDELNVK
NZS1         GGARPNGSVGLGVSAENENSKIVKQQAGISAKRITGEINNLNLTGGYIENKGNPDELNVK
NZS2         GGARPNGSVGLGVSAENENSKIVKQQAGISAKRITGEINNLNLTGGYIENKGNPDELNVK
NZS3         GGARPNGSVGLGVSAENENSKIVKQQAGISAKRITGEINNLNLTGGYIENKGNPDELNVK
NZS4         GGARPNGSVGLGVSAENENSKIVKQQAGISAKRITGEINNLNLTGGYIENKGNPDELNVK
NZV1         GGARPNGSVGLGVSAENENSKIVKQQAGISAKRITGEINNLNLTGGYIENKGNPDELNVK
82-029362    GGARPNGSVGLGVSAENENSKIVKQQAGISAKRITGEINNLNLTGGYIENKGNPDELNVK
6644         GGARPNGSVGLGVSAENENSKIVKQQAGISAKRITGEINNLNLTGGYIENKGNPDELNVK
HD183        GGARPNGSVGLGVSAENENSKIVKQQAGISAKRITGEINNLNLTGGYIENKGNPDELNVK
HMC46        GGARPNGSVGLGVSAENENSKIVKQQAGISAKRITGEINNLNLTGGYIENKGNPDELNVK
HMC56        GGARPNGSVGLGVSAENENSKIVKQQAGISAKRITGEINNLNLTGGYIENKGNPDELNVK
             ************************************************************

35000HP      GDITTHELKDEHHKDGGSFGGSVGVSETGVTQVNVNGGRVEQKHYEATQHSSISGINTKG
NZS1         GDITTHELKDEHHKDGGSFGGSVGVSETGVTQVNVNGGRVEQKHYEATQHSSISGINTKG
NZS2         GDITTHELKDEHHKDGGSFGGSVGVSETGVTQVNVNGGRVEQKHYEATQHSSISGINTKG
NZS3         GDITTHELKDEHHKDGGSFGGSVGVSETGVTQVNVNGGRVEQKHYEATQHSSISGINTKG
NZS4         GDITTHELKDEHHKDGGSFGGSVGVSETGVTQVNVNGGRVEQKHYEATQHSSISGINTKG
NZV1         GDITTHELKDEHHKDGGSFGGSVGVSETGVTQVNVNGGRVEQKHYEATQHSSISGINTKG
82-029362    GDITTHELKDEHHKDGGSFGGSVGVSETGVTQVNVNGGRVEQKHYEATQHSSISGINTKG
6644         GDITTHELKDEHHKDGGSFGGSVGVSETGVTQVNVNGGRVEQKHYEATQHSSISGINTKG
HD183        GDITTHELKDEHHKDGGSFGGSVGVSETGVTQVNVNGGRVEQKHYEATQHSSISGINTKG
HMC46        GDITTHELKDEHHKDGGSFGGSVGVSETGVTQVNVNGGRVEQKHYEATQHSSISGINTKG
HMC56        GDITTHELKDEHHKDGGSFGGSVGVSETGVTQVNVNGGRVEQKHYEATQHSSISGINTKG
             ************************************************************

35000HP      KTVGNFKTDRSQSTEVHRDDTIAATNFNFELGDIAELAKKGKEKWDNRSAKTTSSSQDSA
NZS1         KTVGNFKTDRSQSTEVHRDDTIAATNFNFELGDIAELAKKGKEKWDNRSAKTTSSSQDSA
NZS2         KTVGNFKTDRSQSTEVHRDDTIAATNFNFELGDIAELAKKGKEKWDNRSAKTTSSSQDSA
NZS3         KTVGNFKTDRSQSTEVHRDDTIAATNFNFELGDIAELAKKGKEKWDNRSAKTTSSSQDSA
NZS4         KTVGNFKTDRSQSTEVHRDDTIAATNFNFELGDIAELAKKGKEKWDNRSAKTTSSSQDSA
NZV1         KTVGNFKTDRSQSTEVHRDDTIAATNFNFELGDIAELAKKGKEKWDNRSAKTTSSSQDSA
82-029362    KTVGNFKTDRSQSTEVHRDDTIAATNFNFELGDIAELAKKGKEKWDNRSAKTTSSSQDSA
6644         KTVGNFKTDRSQSTEVHRDDTIAATNFNFELGDIAELAKKGKEKWDNRSAKTTSSSQDSA
HD183        KTVGNFKTDRSQSTEVHRDDTIAATNFNFELGDIAELAKKGKEKWDNRSAKTTSSSQDSA
HMC46        KTVGNFKTDRSQSTEVHRDDTIAATNFNFELGDIAELAKKGKEKWDNRSAKTTSSSQDSA
HMC56        KTVGNFKTDRSQSTEVHRDDTIAATNFNFELGDIAELAKKGKEKWDNRSAKTTSSSQDSA
             ************************************************************

35000HP      HDPRSRSVENGYSELPRFKTADNDAGVDSPRLIKGEAEQAQTLALTKAGNDVIPEVQSLT
NZS1         HDPRSRSVENGYSELPRFKTADNDAGVDSPRLIKGEAEQAQTLALTKAGNDVIPEVQSLT
NZS2         HDPRSRSVENGYSELPRFKTADNDAGVDSPRLIKGEAEQAQTLALTKAGNDVIPEVQSLT
NZS3         HDPRSRSVENGYSELPRFKTADNDAGVDSPRLIKGEAEQAQTLALTKAGNDVIPEVQSLT
```

```
NZS4         HDPRSRSVENGYSELPRFKTADNDAGVDSPRLIKGEAEQAQTLALTKAGNDVIPEVQSLT
NZV1         HDPRSRSVENGYSELPRFKTADNDAGVDSPRLIKGEAEQAQTLALTKAGNDVIPEVQSLT
82-029362    HDPRSRSVENGYSELPRFKTADNDAGVDSPRLIKGEAEQAQTLALTKAGNDVIPEVQSLT
6644         HDPRSRSVENGYSELPRFKTADNDAGVDSPRLIKGEAEQAQTLALTKAGNDVIPEVQSLT
HD183        HDPRSRSVENGYSELPRFKTADNDAGVDSPRLIKGEAEQAQTLALTKAGNDVIPEVQSLT
HMC46        HDPRSRSVENGYSELPRFKTADNDAGVDSPRLIKGEAEQAQTLALTKAGNDVIPEVQSLT
HMC56        HDPRSRSVENGYSELPRFKTADNDAGVDSPRLIKGEAEQAQTLALTKAGNDVIPEVQSLT
             ************************************************************

35000HP      QKARPQSLVDESPYAEIPALTRPQVKSNIAESIEVPQFRTKVSDGDEGNYAEITFPTNKA
NZS1         QKARPQSLVDESPYAEIPALTRPQVKSNIAESIEVPQFRTKVSDGDEGNYAEITFPTNKA
NZS2         QKARPQSLVDESPYAEIPALTRPQVKSNIAESIEVPQFRTKVSDGDEGNYAEITFPTNKA
NZS3         QKARPQSLVDESPYAEIPALTRPQVKSNIAESIEVPQFRTKVSDGDEGNYAEITFPTNKA
NZS4         QKARPQSLVDESPYAEIPALTRPQVKSNIAESIEVPQFRTKVSDGDEGNYAEITFPTNKA
NZV1         QKARPQSLVDESPYAEIPALTRPQVKSNIAESIEVPQFRTKVSDGDEGNYAEITFPTNKA
82-029362    QKARPQSLVDESPYAEIPALTRPQVKSNIAESIEVPQFRTKVSDGDEGNYAEITFPTNKA
6644         QKARPQSLVDESPYAEIPALTRPQVKSNIAESIEVPQFRTKVSDGDEGNYAEITFPTNKA
HD183        QKARPQSLVDESPYAEIPALTRPQVKSNIAESIEVPQFRTKVSDGDEGNYAEITFPTNKA
HMC46        QKARPQSLVDESPYAEIPALTRPQVKSNIAESIEVPQFRTKVSDGDEGNYAEITFPTNKA
HMC56        QKARPQSLVDESPYAEIPALTRPQVKSNIAESIEVPQFRTKVSDGDEGNYAEITFPTNKA
             ************************************************************

35000HP      AISSTQDVGDTPTPRALRLESESGYESAENLGLIPRGFKSSPKGEYEDISDAIEPQTRSR
NZS1         AISSTQDVGDTPTPRALRLESESGYESAENLGLIPRGFKSSPKGEYEDISDAIEPQTRSR
NZS2         AISSTQDVGDTPTPRALRLESESGYESAENLGLIPRGFKSSPKGEYEDISDAIEPQTRSR
NZS3         AISSTQDVGDTPTPRALRLESESGYESAENLGLIPRGFKSSPKGEYEDISDAIEPQTRSR
NZS4         AISSTQDVGDTPTPRALRLESESGYESAENLGLIPRGFKSSPKGEYEDISDAIEPQTRSR
NZV1         AISSTQDVGDTPTPRALRLESESGYESAENLGLIPRGFKSSPKGEYEDISDAIEPQTRSR
82-029362    AISSTQDVGDTPTPRALRLESESGYESAENLGLIPRGFKSSPKGEYEDISDAIEPQTRSR
6644         AISSTQDVGDTPTPRALRLESESGYESAENLGLIPRGFKSSPKGEYEDISDAIEPQTRSR
HD183        AISSTQDVGDTPTPRALRLESESGYESAENLGLIPRGFKSSPKGEYEDISDAIEPQTRSR
HMC46        AISSTQDVGDTPTPRALRLESESGYESAENLGLIPRGFKSSPKGEYEDISDAIEPQTRSR
HMC56        AISSTQDVGDTPTPRALRLESESGYESAENLGLIPRGFKSSPKGEYEDISDAIEPQTRSR
             ************************************************************

35000HP      KLDEPEPIYGTINKSPEAIARANAKADEAIQALGYDPRIKPVVPEEAPPALPPRNLQTKA
NZS1         KLDEPEPIYGTINKSPEAIARANAKADEAIQALGYDPRIKPVVPEEAPPALPPRNLQTKA
NZS2         KLDEPEPIYGTINKSPEAIARANAKADEAIQALGYDPRIKPVVPEEAPPALPPRNLQTKA
NZS3         KLDEPEPIYGTINKSPEAIARANAKADEAIQALGYDPRIKPVVPEEAPPALPPRNLQTKA
NZS4         KLDEPEPIYGTINKSPEAIARANAKADEAIQALGYDPRIKPVVPEEAPPALPPRNLQTKA
NZV1         KLDEPEPIYGTINKSPEAIARANAKADEAIQALGYDPRIKPVVPEEAPPALPPRNLQTKA
82-029362    KLDEPEPIYGTINKSPEAIARANAKADEAIQALGYDPRIKPVVPEEAPPALPPRNLQTKA
6644         KLDEPEPIYGTINKSPEAIARANAKADEAIQALGYDPRIKPVVPEEAPPALPPRNLQTKA
HD183        KLDEPEPIYGTINKSPEAIARANAKADEAIQALGYDPRIKPVVPEEAPPALPPRNLQTKA
HMC46        KLDEPEPIYGTINKSPEAIARANAKADEAIQALGYDPRIKPVVPEEAPPALPPRNLQTKA
HMC56        KLDEPEPIYGTINKSPEAIARANAKADEAIQALGYDPRIKPVVPEEAPPALPPRNLQTKA
             ************************************************************

35000HP      ISDYDDVSYVPDFKVRKTDEPEPIYGTINKSPEAIARANAKADEAIQASGYDPRIKPVVP
NZS1         ISDYDDVSYVPDFKVRKTDEPEPIYGTINKSPEAIARANAKADEAIQASGYDPRIKPVVP
NZS2         ISDYDDVSYVPDFKVRKTDEPEPIYGTINKSPEAIARANAKADEAIQASGYDPRIKPVVP
NZS3         ISDYDDVSYVPDFKVRKTDEPEPIYGTINKSPEAIARANAKADEAIQASGYDPRIKPVVP
NZS4         ISDYDDVSYVPDFKVRKTDEPEPIYGTINKSPEAIARANAKADEAIQASGYDPRIKPVVP
NZV1         ISDYDDVSYVPDFKVRKTDEPEPIYGTINKSPEAIARANAKADEAIQASGYDPRIKPVVP
82-029362    ISDYDDVSYVPDFKVRKTDEPEPIYGTINKSPEAIARANAKADEAIQASGYDPRIKPVVP
6644         ISDYDDVSYVPDFKVRKTDEPEPIYGTINKSPEAIARANAKADEAIQASGYDPRIKPVVP
HD183        ISDYDDVSYVPDFKVRKTDEPEPIYGTINKSPEAIARANAKADEAIQASGYDPRIKPVVP
HMC46        ISDYDDVSYVPDFKVRKTDEPEPIYGTINKSPEAIARANAKADEAIQASGYDPRIKPVVP
HMC56        ISDYDDVSYVPDFKVRKTDEPEPIYGTINKSPEAIARANAKADEAIQASGYDPRIKPVVP
             ************************************************************

35000HP      EDAPPALPPRTQSLIDSTEVPSYRSALANVKFDDASPWPQPSALRSKAFADEPSSETPKS
NZS1         EDAPPALPPRTQSLIDSTEVPSYRSALANVKFDDASPWPQPSALRSKAFADEPSSETPKS
NZS2         EDAPPALPPRTQSLIDSTEVPSYRSALANVKFDDASPWPQPSALRSKAFADEPSSETPKS
NZS3         EDAPPALPPRTQSLIDSTEVPSYRSALANVKFDDASPWPQPSALRSKAFADEPSSETPKS
NZS4         EDAPPALPPRTQSLIDSTEVPSYRSALANVKFDDASPWPQPSALRSKAFADEPSSETPKS
NZV1         EDAPPALPPRTQSLIDSTEVPSYRSALANVKFDDASPWPQPSALRSKAFADEPSSETPKS
82-029362    EDAPPALPPRTQSLIDSTEVPSYRSALANVKFDDASPWPQPSALRSKAFADEPSSETPKS
6644         EDAPPALPPRTQSLIDSTEVPSYRSALANVKFDDASPWPQPSALRSKAFADEPSSETPKS
HD183        EDAPPALPPRTQSLIDSTEVPSYRSALANVKFDDASPWPQPSALRSKAFADEPSSETPKS
HMC46        EDAPPALPPRTQSLIDSTEVPSYRSALANVKFDDASPWPQPSALRSKAFADEPSSETPKS
HMC56        EDAPPALPPRTQSLIDSTEVPSYRSALANVKFDDASPWPQPSALRSKAFADEPSSETPKS
             ************************************************************
```

```
35000HP       RGKRGISEESLSSTVQPRSRKISEEDSSFERLPLRIIDNGSDYAEILPRNVKQTNEPATQ
NZS1          RGKRGISEESLSSTVQPRSRKISEEDSSFERLPLRIIDNGSDYAEILPRNVKQTNEPATQ
NZS2          RGKRGISEESLSSTVQPRSRKISEEDSSFERLPLRIIDNGSDYAEILPRNVKQTNEPATQ
NZS3          RGKRGISEESLSSTVQPRSRKISEEDSSFERLPLRIIDNGSDYAEILPRNVKQTNEPATQ
NZS4          RGKRGISEESLSSTVQPRSRKISEEDSSFERLPLRIIDNGSDYAEILPRNVKQTNEPATQ
NZV1          RGKRGISEESLSSTVQPRSRKISEEDSSFERLPLRIIDNGSDYAEILPRNVKQTNEPATQ
82-029362     RGKRGISEESLSSTVQPRSRKISEEDSSFERLPLRIIDNGSDYAEILPRNVKQTNEPATQ
6644          RGKRGISEESLSSTVQPRSRKISEEDSSFERLPLRIIDNGSDYAEILPRNVKQTNEPATQ
HD183         RGKRGISEESLSSTVQPRSRKISEEDSSFERLPLRIIDNGSDYAEILPRNVKQTNEPATQ
HMC46         RGKRGISEESLSSTVQPRSRKISEEDSSFERLPLRIIDNGSDYAEILPRNVKQTNEPATQ
HMC56         RGKRGISEESLSSTVQPRSRKISEEDSSFERLPLRIIDNGSDYAEILPRNVKQTNEPATQ
              ************************************************************

35000HP       AIRAPKALDNNDVIAERPSFKLRQLDDDVESVNGIYSSIKPKALIEEGTPITRQVKTVQE
NZS1          AIRAPKALDNNDVIAERPSFKLRQLDDDVESVNGIYSSIKPKALIEEGTPITRQVKTVQE
NZS2          AIRAPKALDNNDVIAERPSFKLRQLDDDVESVNGIYSSIKPKALIEEGTPITRQVKTVQE
NZS3          AIRAPKALDNNDVIAERPSFKLRQLDDDVESVNGIYSSIKPKALIEEGTPITRQVKTVQE
NZS4          AIRAPKALDNNDVIAERPSFKLRQLDDDVESVNGIYSSIKPKALIEEGTPITRQVKTVQE
NZV1          AIRAPKALDNNDVIAERPSFKLRQLDDDVESVNGIYSSIKPKALIEEGTPITRQVKTVQE
82-029362     AIRAPKALDNNDVIAERPSFKLRQLDDDVESVNGIYSSIKPKALIEEGTPITRQVKTVQE
6644          AIRAPKALDNNDVIAERPSFKLRQLDDDVESVNGIYSSIKPKALIEEGTPITRQVKTVQE
HD183         AIRAPKALDNNDVIAERPSFKLRQLDDDVESVNGIYSSIKPKALIEEGTPITRQVKTVQE
HMC46         AIRAPKALDNNDVIAERPSFKLRQLDDDVESVNGIYSSIKPKALIEEGTPITRQVKTVQE
HMC56         AIRAPKALDNNDVIAERPSFKLRQLDDDVESVNGIYSSIKPKALIEEGTPITRQVKTVQE
              ************************************************************

35000HP       ETPVTDLVNKRELVKEDRSLLDKVQDTFQPLKVRSKINDVRSSVEEYGGEVTFKYAQSKG
NZS1          ETPVTNLVNKRELVKEDRSLLDKVQDTFQPLKVRSKINDVRSSVEEYGGEVTFKYAQSKG
NZS2          ETPVTNLVNKRELVKEDRSLLDKVQDTFQPLKVRSKINDVRSSVEEYGGEVTFKYAQSKG
NZS3          ETPVTNLVNKRELVKEDRSLLDKVQDTFQPLKVRSKINDVRSSVEEYGGEVTFKYAQSKG
NZS4          ETPVTNLVNKRELVKEDRSLLDKVQDTFQPLKVRSKINDVRSSVEEYGGEVTFKYAQSKG
NZV1          ETPVTNLVNKRELVKEDRSLLDKVQDTFQPLKVRSKINDVRSSVEEYGGEVTFKYAQSKG
82-029362     ETPVTDLVNKRELVKEDRSLLDKVQDTFQPLKVRSKINDVRSSVEEYGGEVTFKYAQSKG
6644          ETPVTDLVNKRELVKEDRSLLDKVQDTFQPLKVRSKINDVRSSVEEYGGEVTFKYAQSKG
HD183         ETPVTDLVNKRELVKEDRSLLDKVQDTFQPLKVRSKINDVRSSVEEYGGEVTFKYAQSKG
HMC46         ETPVTDLVNKRELVKEDRSLLDKVQDTFQPLKVRSKINDVRSSVEEYGGEVTFKYAQSKG
HMC56         ETPVTDLVNKRELVKEDRSLLDKVQDTFQPLKVRSKINDVRSSVEEYGGEVTFKYAQSKG
              *****:******************************************************

35000HP       EVYNEIVKHAETQNGVCEATCSHWIAKKVNDENIWTDLYKDGQKGRKGGLNKDAIESIEK
NZS1          EVYNEIVKHAETQNGVCEATCSHWIAKKVNDENIWTDLYKDGQKGRKGGLNKDAIESIEK
NZS2          EVYNEIVKHAETQNGVCEATCSHWIAKKVNDENIWTDLYKDGQKGRKGGLNKDAIESIEK
NZS3          EVYNEIVKHAETQNGVCEATCSHWIAKKVNDENIWTDLYKDGQKGRKGGLNKDAIESIEK
NZS4          EVYNEIVKHAETQNGVCEATCSHWIAKKVNDENIWTDLYKDGQKGRKGGLNKDAIESIEK
NZV1          EVYNEIVKHAETQNGVCEATCSHWIAKKVNDENIWTDLYKDGQKGRKGGLNKDAIESIEK
82-029362     EVYNEIVKHAETQNGVCEATCSHWIAKKVNDENIWTDLYKDGQKGRKGGLNKDAIESIEK
6644          EVYNEIVKHAETQNGVCEATCSHWIAKKVNDENIWTDLYKDGQKGRKGGLNKDAIESIEK
HD183         EVYNEIVKHAETQNGVCEATCSHWIAKKVNDENIWTDLYKDGQKGRKGGLNKDAIESIEK
HMC46         EVYNEIVKHAETQNGVCEATCSHWIAKKVNDENIWTDLYKDGQKGRKGGLNKDAIESIEK
HMC56         EVYNEIVKHAETQNGVCEATCSHWIAKKVNDENIWTDLYKDGQKGRKGGLNKDAIESIEK
              ************************************************************

35000HP       LQTEFINAGTATQQFKLTNTWLEEQGVVPKQKYFGKLSRADEVAGTVSKNDVSALVKAIL
NZS1          LQTEFINAGTATQQFKLTNTWLEEQGVVPKQKYFGKLSRADEVAGTVSKNDVSALVKAIL
NZS2          LQTEFINAGTATQQFKLTNTWLEEQGVVPKQKYFGKLSRADEVAGTVSKNDVSALVKAIL
NZS3          LQTEFINAGTATQQFKLTNTWLEEQGVVPKQKYFGKLSRADEVAGTVSKNDVSALVKAIL
NZS4          LQTEFINAGTATQQFKLTNTWLEEQGVVPKQKYFGKLSRADEVAGTVSKNDVSALVKAIL
NZV1          LQTEFINAGTATQQFKLTNTWLEEQGVVPKQKYFGKLSRADEVAGTVSKNDVSALVKAIL
82-029362     LQTEFINAGTATQQFKLTNTWLEEQGVVPKQKYFGKLSRADEVAGTVSKNDVSALVKAIL
6644          LQTEFINAGTATQQFKLTNTWLEEQGVVPKQKYFGKLSRADEVAGTVSKNDVSALVKAIL
HD183         LQTEFINAGTATQQFKLTNTWLEEQGVVPKQKYFGKLSRADEVAGTVSKNDVSALVKAIL
HMC46         LQTEFINAGTATQQFKLTNTWLEEQGVVPKQKYFGKLSRADEVAGTVSKNDVSALVKAIL
HMC56         LQTEFINAGTATQQFKLTNTWLEEQGVVPKQKYFGKLSRADEVAGTVSKNDVSALVKAIL
              ************************************************************

35000HP       DTGNESSAVKKISINLEGGSHTVSASIEGQKVVFFDPNFGEITFKDKKSFEKWMKNAFWK
NZS1          DTGNESSAVKKISINLEGGSHTVSASIEGQKVVFFDPNFGEITFKDKKSFEKWMKNAFWK
NZS2          DTGNESSAVKKISINLEGGSHTVSASIEGQKVVFFDPNFGEITFKDKKSFEKWMKNAFWK
NZS3          DTGNESSAVKKISINLEGGSHTVSASIEGQKVVFFDPNFGEITFKDKKSFEKWMKNAFWK
NZS4          DTGNESSAVKKISINLEGGSHTVSASIEGQKVVFFDPNFGEITFKDKKSFEKWMKNAFWK
NZV1          DTGNESSAVKKISINLEGGSHTVSASIEGQKVVFFDPNFGEITFKDKKSFEKWMKNAFWK
82-029362     DTGNESSAVKKISINLEGGSHTVSASIEGQKVVFFDPNFGEITFKDKKSFEKWMKNAFWK
```

```
6644            DTGNESSAVKKISINLEGGSHTVSASIEGQKVVFFDPNFGEITFKDKKSFEKWMKNAFWK
HD183           DTGNESSAVKKISINLEGGSHTVSASIEGQKVVFFDPNFGEITFKDKKSFEKWMKNAFWK
HMC46           DTGNESSAVKKISINLEGGSHTVSASIEGQKVVFFDPNFGEITFKDKKSFEKWMKNAFWK
HMC56           DTGNESSAVKKISINLEGGSHTVSASIEGQKVVFFDPNFGEITFKDKKSFEKWMKNAFWK
                ************************************************************

35000HP         KSGYAGKKDTKRFFNVVNYHKNSKRNKVIDVNQNHIQQLAGSEGFSPSLPTRPQLANAAG
NZS1            KSGYAGKKDTKRFFNVVNYHKNSKRNKVIDVNQNHIQQLAGSEGFSPSLPTRPQLANAAG
NZS2            KSGYAGKKDTKRFFNVVNYHKNSKRNKVIDVNQNHIQQLAGSEGFSPSLPTRPQLANAAG
NZS3            KSGYAGKKDTKRFFNVVNYHKNSKRNKVIDVNQNHIQQLAGSEGFSPSLPTRPQLANAAG
NZS4            KSGYAGKKDTKRFFNVVNYHKNSKRNKVIDVNQNHIQQLAGSEGFSPSLPTRPQLANAAG
NZV1            KSGYAGKKDTKRFFNVVNYHKNSKRNKVIDVNQNHIQQLAGSEGFSPSLPTRPQLANAAG
82-029362       KSGYAGKKDTKRFFNVVNYHKNSKRNKVIDVNQNHIQQLAGSEGFSPSLPTRPQLANAAG
6644            KSGYAGKKDTKRFFNVVNYHKNSKRNKVIDVNQNHIQQLAGSEGFSPSLPTRPQLANAAG
HD183           KSGYAGKKDTKRFFNVVNYHKNSKRNKVIDVNQNHIQQLAGSEGFSPSLPTRPQLANAAG
HMC46           KSGYAGKKDTKRFFNVVNYHKNSKRNKVIDVNQNHIQQLAGSEGFSPSLPTRPQLANAAG
HMC56           KSGYAGKKDTKRFFNVVNYHKNSKRNKVIDVNQNHIQQLAGSEGFSPSLPTRPQLANAAG
                ************************************************************

35000HP         IKSNEMSSLFSWSKLKHLFSRESGKKAQVEGPEIKHLGGVVDKDAFYFPLDKIVTRRDAE
NZS1            IKSNEMSSLFSWSKLKHLFSRESGKKAQVEGPEIKHLGGVVDKDAFYFPLDKIVTRRDAE
NZS2            IKSNEMSSLFSWSKLKHLFSRESGKKAQVEGPEIKHLGGVVDKDAFYFPLDKIVTRRDAE
NZS3            IKSNEMSSLFSWSKLKHLFSRESGKKAQVEGPEIKHLGGVVDKDAFYFPLDKIVTRRDAE
NZS4            IKSNEMSSLFSWSKLKHLFSRESGKKAQVEGPEIKHLGGVVDKDAFYFPLDKIVTRRDAE
NZV1            IKSNEMSSLFSWSKLKHLFSRESGKKAQVEGPEIKHLGGVVDKDAFYFPLDKIVTRRDAE
82-029362       IKSNEMSSLFSWSKLKHLFSRESGKKAQVEGPEIKHLGGVVDKDAFYFPLDKIVTRRDAE
6644            IKSNEMSSLFSWSKLKHLFSRESGKKAQVEGPEIKHLGGVVDKDAFYFPLDKIVTRRDAE
HD183           IKSNEMSSLFSWSKLKHLFSRESGKKAQVEGPEIKHLGGVVDKDAFYFPLDKIVTRRDAE
HMC46           IKSNEMSSLFSWSKLKHLFSRESGKKAQVEGPEIKHLGGVVDKDAFYFPLDKIVTRRDAE
HMC56           IKSNEMSSLFSWSKLKHLFSRESGKKAQVEGPEIKHLGGVVDKDAFYFPLDKIVTRRDAE
                ************************************************************

35000HP         GEIRVNMDNIKKAFNPRDKHYNSQEARSLRSLYNQDPSMSGTRFIIENQVIANPFSSADL
NZS1            GEIRVNMDNIKKAFNPRDKHYNSQEARSLRSLYNQDPSMSGTRFIIENQVIANPFSSADL
NZS2            GEIRVNMDNIKKAFNPRDKHYNSQEARSLRSLYNQDPSMSGTRFIIENQVIANPFSSADL
NZS3            GEIRVNMDNIKKAFNPRDKHYNSQEARSLRSLYNQDPSMSGTRFIIENQVIANPFSSADL
NZS4            GEIRVNMDNIKKAFNPRDKHYNSQEARSLRSLYNQDPSMSGTRFIIENQVIANPFSSADL
NZV1            GEIRVNMDNIKKAFNPRDKHYNSQEARSLRSLYNQDPSMSGTRFIIENQVIANPFSSADL
82-029362       GEIRVNMDNIKKAFNPRDKHYNSQEARSLRSLYNQDPSMSGTRFIIENQVIANPFSSADL
6644            GEIRVNMDNIKKAFNPRDKHYNSQEARSLRSLYNQDPSMSGTRFIIENQVIANPFSSADL
HD183           GEIRVNMDNIKKAFNPRDKHYNSQEARSLRSLYNQDPSMSGTRFIIENQVIANPFSSADL
HMC46           GEIRVNMDNIKKAFNPRDKHYNSQEARSLRSLYNQDPSMSGTRFIIENQVIANPFSSADL
HMC56           GEIRVNMDNIKKAFNPRDKHYNSQEARSLRSLYNQDPSMSGTRFIIENQVIANPFSSADL
                ************************************************************

35000HP         QSYIQAQQSKLPELGRQARRALPELPTAANKGRGSRVEEQNIVTRPRVEDVYATVNKGAK
NZS1            QSYIQAQQSKLPELGRQARRALPELPTAANKGRGSRVEEQNIVTRPRVEDVYATVNKGAK
NZS2            QSYIQAQQSKLPELGRQARRALPELPTAANKGRGSRVEEQNIVTRPRVEDVYATVNKGAK
NZS3            QSYIQAQQSKLPELGRQARRALPELPTAANKGRGSRVEEQNIVTRPRVEDVYATVNKGAK
NZS4            QSYIQAQQSKLPELGRQARRALPELPTAANKGRGSRVEEQNIVTRPRVEDVYATVNKGAK
NZV1            QSYIQAQQSKLPELGRQARRALPELPTAANKGRGSRVEEQNIVTRPRVEDVYATVNKGAK
82-029362       QSYIQAQQSKLPELGRQARRALPELPTAANKGRGSRVEEQNIVTRPRVEDVYATVNKGAK
6644            QSYIQAQQSKLPELGRQARRALPELPTAANKGRGSRVEEQNIVTRPRVEDVYATVNKGAK
HD183           QSYIQAQQSKLPELGRQARRALPELPTAANKGRGSRVEEQNIVTRPRVEDVYATVNKGAK
HMC46           QSYIQAQQSKLPELGRQARRALPELPTAANKGRGSRVEEQNIVTRPRVEDVYATVNKGAK
HMC56           QSYIQAQQSKLPELGRQARRALPELPTAANKGRGSRVEEQNIVTRPRVEDVYATVNKGAK
                ************************************************************

35000HP         HGEAQQPGSFYTKKLVDQVSHVPNTEPVYADLHFNRNGRVVRQTEPEVIYEKIRGQQVEV
NZS1            HGEAQQPGSFYTKKLVDQVSHVPNTEPVYADLHFNRNGRVVRQTEPEVIYEKIRGQQVEV
NZS2            HGEAQQPGSFYTKKLVDQVSHVPNTEPVYADLHFNRNGRVVRQTEPEVIYEKIRGQQVEV
NZS3            HGEAQQPGSFYTKKLVDQVSHVPNTEPVYADLHFNRNGRVVRQTEPEVIYEKIRGQQVEV
NZS4            HGEAQQPGSFYTKKLVDQVSHVPNTEPVYADLHFNRNGRVVRQTEPEVIYEKIRGQQVEV
NZV1            HGEAQQPGSFYTKKLVDQVSHVPNTEPVYADLHFNRNGRVVRQTEPEVIYEKIRGQQVEV
82-029362       HGEAQQPGSFYTKKLVDQVSHVPTTEPVYADLHFNRNGRVVRQTEPEVIYEKIRGQQVEV
6644            HGEAQQPGSFYTKKLVDQVSHVPTTEPVYADLHFNRNGRVVRQTEPEVIYEKIRGQQVEV
HD183           HGEAQQPGSFYTKKLVDQVSHVPNTEPVYADLHFNRNGRVVRQTEPEVIYEKIRGQQVEV
HMC46           HGEAQQPGSFYTKKLVDQVSHVPTTEPVYADLHFNRNGRVVRQTEPEVIYEKIRGQQVEV
HMC56           HGEAQQPGSFYTKKLVDQVSHVPTTEPVYADLHFNRNGRVVRQTEPEVIYEKIRGQQVEV
                ***********************.************************************

35000HP         DDPSSLYAKVNRNRRLDNVEGFYPPEQLRTRSDKLAEQVSRVPTTEPVYADLRFKSAEDD
NZS1            DDPSSLYAKVNRNRRLDNVEGFYPPEQLRTRSDKLAEQVSRVPTTEPVYADLRFKSAEDD
```

```
NZS2          DDPSSLYAKVNRNRRLDNVEGFYPPEQLRTRSDKLAEQVSRVPTTEPVYADLRFKSAEDD
NZS3          DDPSSLYAKVNRNRRLDNVEGFYPPEQLRTRSDKLAEQVSRVPTTEPVYADLRFKSAEDD
NZS4          DDPSSLYAKVNRNRRLDNVEGFYPPEQLRTRSDKLAEQVSRVPTTEPVYADLRFKSAEDD
NZV1          DDPSSLYAKVNRNRRLDNVEGFYPPEQLRTRSDKLAEQVSRVPTTEPVYADLRFKSAEDD
82-029362     DDPSSLYAKVNRNRRLDNVEGFYPPEQLRTRSDKLAEQVSRVPTTEPVYADLRFKSAEDD
6644          DDPSSLYAKVNRNRRLDNVEGFYPPEQLRTRSDKLAEQVSRVPTTEPVYADLRFKSAEDD
HD183         DDPSSLYAKVNRNRRLDNVEGFYPPEQLRTRSDKLAEQVSRVPTTEPVYADLRFKSAEDD
HMC46         DDPSSLYAKVNRNRRLDNVEGFYPPEQLRTRSDKLAEQVSRVPTTEPVYADLRFKSAEDD
HMC56         DDPSSLYAKVNRNRRLDNVEGFYPPEQLRTRSDKLAEQVSRVPTTEPVYADLRFKSAEDD
              ************************************************************

35000HP       YAPALPARPELGNAAGFRKAKVKGEESESTWSRLKHLFSRESGKTKVEEVENEYKSQTNG
NZS1          YAPALPARPELGNAAGFRKAKVKGEESESTWSRLKHLFSRESGKTKVEEVENEYKSQTNG
NZS2          YAPALPARPELGNAAGFRKAKVKGEESESTWSRLKHLFSRESGKTKVEEVENEYKSQTNG
NZS3          YAPALPARPELGNAAGFRKAKVKGEESESTWSRLKHLFSRESGKTKVEEVENEYKSQTNG
NZS4          YAPALPARPELGNAAGFRKAKVKGEESESTWSRLKHLFSRESGKTKVEEVENEYKSQTNG
NZV1          YAPALPARPELGNAAGFRKAKVKGEESESTWSRLKHLFSRESGKTKVEEVENEYKSQTNG
82-029362     YAPALPARPELGNAAGFRKAKVKGEESESTWSRLKHLFSRESGKTKVEEVENEYKSQTNG
6644          YAPALPARPELGNAAGFRKAKVKGEESESTWSRLKHLFSRESGKTKVEEVENEYKSQTNG
HD183         YAPALPARPELGNAAGFRKAKVKGEESESTWSRLKHLFSRESGKTKVEEVENEYKSQTNG
HMC46         YAPALPARPELGNAAGFRKAKVKGEESESTWSRLKHLFSRESGKTKVEEVENEYKSQTNG
HMC56         YAPALPARPELGNAAGFRKAKVKGEESESTWSRLKHLFSRESGKTKVEEVENEYKSQTNG
              ************************************************************

35000HP       ENSLEIKTIEHIPTRLKNLESPREAQSDLGENALIYGLQRGRQALISKANAADKEGKNAI
NZS1          ENSLEIKTIEHIPTRLKNLESPREAQSDLGENALIYGLQRGRQALISKANAADKEGKNAI
NZS2          ENSLEIKTIEHIPTRLKNLESPREAQSDLGENALIYGLQRGRQALISKANAADKEGKNAI
NZS3          ENSLEIKTIEHIPTRLKNLESPREAQSDLGENALIYGLQRGRQALISKANAADKEGKNAI
NZS4          ENSLEIKTIEHIPTRLKNLESPREAQSDLGENALIYGLQRGRQALISKANAADKEGKNAI
NZV1          ENSLEIKTIEHIPTRLKNLESPREAQSDLGENALIYGLQRGRQALISKANAADKEGKNAI
82-029362     ENSLEIKTIEHIPTRLKNLESPREAQSDLGENALIYGLQRGRQALISKANAADKEGKNAI
6644          ENSLEIKTIEHIPTRLKNLESPREAQSDLGENALIYGLQRGRQALISKANAADKEGKNAI
HD183         ENSLEIKTIEHIPTRLKNLESPREAQSDLGENALIYGLQRGRQALISKANAADKEGKNAI
HMC46         ENSLEIKTIEHIPTRLKNLESPREAQSDLGENALIYGLQRGRQALISKANAADKEGKNAI
HMC56         ENSLEIKTIEHIPTRLKNLESPREAQSDLGENALIYGLQRGRQALISKANAADKEGKNAI
              ************************************************************

35000HP       LADSYIGKLNLGFEFGELTKFAKQVKDGKVTEQDIQNIASFNDETAKLARRSEPKNRIND
NZS1          LADSYIGKLNLGFEFGELTKFAKQVKDGKVTEQDIQNIASFNDETAKLARRSEPKNRIND
NZS2          LADSYIGKLNLGFEFGELTKFAKQVKDGKVTEQDIQNIASFNDETAKLARRSEPKNRIND
NZS3          LADSYIGKLNLGFEFGELTKFAKQVKDGKVTEQDIQNIASFNDETAKLARRSEPKNRIND
NZS4          LADSYIGKLNLGFEFGELTKFAKQVKDGKVTEQDIQNIASFNDETAKLARRSEPKNRIND
NZV1          LADSYIGKLNLGFEFGELTKFAKQVKDGKVTEQDIQNIASFNDETAKLARRSEPKNRIND
82-029362     LADSYIGKLNLGFEFGELTKFAKQVKDGKVTEQDIQNIASFNDETAKLARRSEPKNRIND
6644          LADSYIGKLNLGFEFGELTKFAKQVKDGKVTEQDIQNIASFNDETAKLARRSEPKNRIND
HD183         LADSYIGKLNLGFEFGELTKFAKQVKDGKVTEQDIQNIASFNDETAKLARRSEPKNRIND
HMC46         LADSYIGKLNLGFEFGELTKFAKQVKDGKVTEQDIQNIASFNDETAKLARRSEPKNRIND
HMC56         LADSYIGKLNLGFEFGELTKFAKQVKDGKVTEQDIQNIASFNDETAKLARRSEPKNRIND
              ************************************************************

35000HP       ANVDDNQRIIRELINNEAAVDALKRIATLSDQEKAMHSTLRANEKFDMDELEESPNYTTA
NZS1          ANVDDNQRIIRELINNEAAVDALKRIATLSDQEKAMHSTLRANEKFDMDELEESPNYTTA
NZS2          ANVDDNQRIIRELINNEAAVDALKRIATLSDQEKAMHSTLRANEKFDMDELEESPNYTTA
NZS3          ANVDDNQRIIRELINNEAAVDALKRIATLSDQEKAMHSTLRANEKFDMDELEESPNYTTA
NZS4          ANVDDNQRIIRELINNEAAVDALKRIATLSDQEKAMHSTLRANEKFDMDELEESPNYTTA
NZV1          ANVDDNQRIIRELINNEAAVDALKRIATLSDQEKAMHSTLRANEKFDMDELEESPNYTTA
82-029362     ANVDDNQRIIRELINNEAAVDALKRIATLSDQEKAMHSTLRANEKFDMDELEESPNYTTA
6644          ANVDDNQRIIRELINNEAAVDALKRIATLSDQEKAMHSTLRANEKFDMDELEESPNYTTA
HD183         ANVDDNQRIIRELINNEAAVDALKRIATLSDQEKAMHSTLRANEKFDMDELEESPNYTTA
HMC46         ANVDDNQRIIRELINNEAAVDALKRIATLSDQEKAMHSTLRANEKFDMDELEESPNYTTA
HMC56         ANVDDNQRIIRELINNEAAVDALKRIATLSDQEKAMHSTLRANEKFDMDELEESPNYTTA
              ************************************************************

35000HP       ENKSIRDYKDTQKALNDARMDFFTEKTKFIAKETLERGGQLYFALDGLVTNSPGFRADTQ
NZS1          ENKSIRDYKDTQKALNDARMDFFTEKTKFIAKETLERGGQLYFALDGLVTNSPGFRADTQ
NZS2          ENKSIRDYKDTQKALNDARMDFFTEKTKFIAKETLERGGQLYFALDGLVTNSPGFRADTQ
NZS3          ENKSIRDYKDTQKALNDARMDFFTEKTKFIAKETLERGGQLYFALDGLVTNSPGFRADTQ
NZS4          ENKSIRDYKDTQKALNDARMDFFTEKTKFIAKETLERGGQLYFALDGLVTNSPGFRADTQ
NZV1          ENKSIRDYKDTQKALNDARMDFFTEKTKFIAKETLERGGQLYFALDGLVTNSPGFRADTQ
82-029362     ENKSIRDYKDTQKALNDARMDFFTEKTKFIAKETLERGGQLYFALDGLVTNSPGFRADTQ
6644          ENKSIRDYKDTQKALNDARMDFFTEKTKFIAKETLERGGQLYFALDGLVTNSPGFRADTQ
HD183         ENKSIRDYKDTQKALNDARMDFFTEKTKFIAKETLERGGQLYFALDGLVTNSPGFRADTQ
HMC46         ENKSIRDYKDTQKALNDARMDFFTEKTKFIAKETLERGGQLYFALDGLVTNSPGFRADTQ
```

```
HMC56         ENKSIRDYKDTQKALNDARMDFFTEKTKFIAKETLERGGQLYFALDGLVTNSPGFRADTQ
              ************************************************************

35000HP       INMDKLKDVFNPNHEHYDSVTSRELRYLYENYKDNPNLKFTLKDHVIANPLKTLKTSISE
NZS1          INMDKLKDVFNPNHEHYDSVTSRELRYLYENYKDNPNLKFTLKDHVIANPLKTLKTSISE
NZS2          INMDKLKDVFNPNHEHYDSVTSRELRYLYENYKDNPNLKFTLKDHVIANPLKTLKTSISE
NZS3          INMDKLKDVFNPNHEHYDSVTSRELRYLYENYKDNPNLKFTLKDHVIANPLKTLKTSISE
NZS4          INMDKLKDVFNPNHEHYDSVTSRELRYLYENYKDNPNLKFTLKDHVIANPLKTLKTSISE
NZV1          INMDKLKDVFNPNHEHYDSVTSRELRYLYENYKDNPNLKFTLKDHVIANPLKTLKTSISE
82-029362     INMDKLKDVFNPNHEHYDSVTSRELRYLYENYKDNPNLKFTLKDHVIANPLKTLKTSISE
6644          INMDKLKDVFNPNHEHYDSVTSRELRYLYENYKDNPNLKFTLKDHVIANPLKTLKTSISE
HD183         INMDKLKDVFNPNHEHYDSVTSRELRYLYENYKDNPNLKFTLKDHVIANPLKTLKTSISE
HMC46         INMDKLKDVFNPNHEHYDSVTSRELRYLYENYKDNPNLKFTLKDHVIANPLKTLKTSISE
HMC56         INMDKLKDVFNPNHEHYDSVTSRELRYLYENYKDNPNLKFTLKDHVIANPLKTLKTSISE
              ************************************************************

35000HP       SDLKSSPRRARQEGPSLLQRVRNLFDKSSSNKRSEKDTAQTSVGYRNTNIDIINDKTKGV
NZS1          SDLKSSPRRARQEGPSLLQRVRNLFDKSSSNKRSEKDTAQTSVGYRNTNIDIINDKTKGV
NZS2          SDLKSSPRRARQEGPSLLQRVRNLFDKSSSNKRSEKDTAQTSVGYRNTNIDIINDKTKGV
NZS3          SDLKSSPRRARQEGPSLLQRVRNLFDKSSSNKRSEKDTAQTSVGYRNTNIDIINDKTKGV
NZS4          SDLKSSPRRARQEGPSLLQRVRNLFDKSSSNKRSEKDTAQTSVGYRNTNIDIINDKTKGV
NZV1          SDLKSSPRRARQEGPSLLQRVRNLFDKSSSNKRSEKDTAQTSVGYRNTNIDIINDKTKGV
82-029362     SDLKSSPRRARQEGPSLLQRVRNLFDKSSSNKRSEKDTAQTSVGYRNTNIDIINDKTKGV
6644          SDLKSSPRRARQEGPSLLQRVRNLFDKSSSNKRSEKDTAQTSVGYRNTNIDIINDKTKGV
HD183         SDLKSSPRRARQEGPSLLQRVRNLFDKSSSNKRSEKDTAQTSVGYRNTNIDIINDKTKGV
HMC46         SDLKSSPRRARQEGPSLLQRVRNLFDKSSSNKRSEKDTAQTSVGYRNTNIDIINDKTKGV
HMC56         SDLKSSPRRARQEGPSLLQRVRNLFDKSSSNKRSEKDTAQTSVGYRNTNIDIINDKTKGV
              ************************************************************

35000HP       NHIVENGVEVALTNTDYKPLKLPNVEAAFKQTKLKAENIDPHIEAVKKLEIIASSANSIP
NZS1          NHIVENGVEVALTNTDYKPLKLPNVEAAFKQTKLKAENIDPHIEAVKKLEIIASSANSIP
NZS2          NHIVENGVEVALTNTDYKPLKLPNVEAAFKQTKLKAENIDPHIEAVKKLEIIASSANSIP
NZS3          NHIVENGVEVALTNTDYKPLKLPNVEAAFKQTKLKAENIDPHIEAVKKLEIIASSANSIP
NZS4          NHIVENGVEVALTNTDYKPLKLPNVEAAFKQTKLKAENIDPHIEAVKKLEIIASSANSIP
NZV1          NHIVENGVEVALTNTDYKPLKLPNVEAAFKQTKLKAENIDPHIEAVKKLEIIASSANSIP
82-029362     NHIVENGVEVALTNTDYKPLKLPNVEAAFKQTKLKAENIDPHIEAVKKLEIIASSANSIP
6644          NHIVENGVEVALTNTDYKPLKLPNVEAAFKQTKLKAENIDPHIEAVKKLEIIASSANSIP
HD183         NHIVENGVEVALTNTDYKPLKLPNVEAAFKQTKLKAENIDPHIEAVKKLEIIASSANSIP
HMC46         NHIVENGVEVALTNTDYKPLKLPNVEAAFKQTKLKAENIDPHIEAVKKLEIIASSANSIP
HMC56         NHIVENGVEVALTNTDYKPLKLPNVEAAFKQTKLKAENIDPHIEAVKKLEIIASSANSIP
              ************************************************************

35000HP       KEHLLKALIEVTEGKTDEDINVYQKLFNTRQNISNEVAPTYSLRNLDGKDGKQILRSVAE
NZS1          KEHLLKALIEVTEGKTDEDINVYQKLFNTRQNISNEVAPTYSLRNLDGKDGKQILRSVAE
NZS2          KEHLLKALIEVTEGKTDEDINVYQKLFNTRQNISNEVAPTYSLRNLDGKDGKQILRSVAE
NZS3          KEHLLKALIEVTEGKTDEDINVYQKLFNTRQNISNEVAPTYSLRNLDGKDGKQILRSVAE
NZS4          KEHLLKALIEVTEGKTDEDINVYQKLFNTRQNISNEVAPTYSLRNLDGKDGKQILRSVAE
NZV1          KEHLLKALIEVTEGKTDEDINVYQKLFNTRQNISNEVAPTYSLRNLDGKDGKQILRSVAE
82-029362     KEHLLKALIEVTEGKTDEDINVYQKLFNTRQNISNEVAPTYSLRNLDGKDGKQILRSVAE
6644          KEHLLKALIEVTEGKTDEDINVYQKLFNTRQNISNEVAPTYSLRNLDGKDGKQILRSVAE
HD183         KEHLLKALIEVTEGKTDEDINVYQKLFNTRQNISNEVAPTYSLRNLDGKDGKQILRSVAE
HMC46         KEHLLKALIEVTEGKTDEDINVYQKLFNTRQNISNEVAPTYSLRNLDGKDGKQILRSVAE
HMC56         KEHLLKALIEVTEGKTDEDINVYQKLFNTRQNISNEVAPTYSLRNLDGKDGKQILRSVAE
              ************************************************************

35000HP       IYKNLPLSDTYQAVRNYVNNRLIEKLSSNRLLLEHLANSKISGNEYAIKYIFDTVSRAKQ
NZS1          IYKNLPLSDTYQAVRNYVNNRLIEKLSSNRLLLEHLANSKISGNEYAIKYIFDTVSRAKQ
NZS2          IYKNLPLSDTYQAVRNYVNNRLIEKLSSNRLLLEHLANSKISGNEYAIKYIFDTVSRAKQ
NZS3          IYKNLPLSDTYQAVRNYVNNRLIEKLSSNRLLLEHLANSKISGNEYAIKYIFDTVSRAKQ
NZS4          IYKNLPLSDTYQAVRNYVNNRLIEKLSSNRLLLEHLANSKISGNEYAIKYIFDTVSRAKQ
NZV1          IYKNLPLSDTYQAVRNYVNNRLIEKLSSNRLLLEHLANSKISGNEYAIKYIFDTVSRAKQ
82-029362     IYKNLPLSDTYQAVRNYVNNRLIEKLSSNRLLLEHLANSKISGNEYAIKYIFDTVSRAKQ
6644          IYKNLPLSDTYQAVRNYVNNRLIEKLSSNRLLLEHLANSKISGNEYAIKYIFDTVSRAKQ
HD183         IYKNLPLSDTYQAVRNYVNNRLIEKLSSNRLLLEHLANSKISGNEYAIKYIFDTVSRAKQ
HMC46         IYKNLPLSDTYQAVRNYVNNRLIEKLSSNRLLLEHLANSKISGNEYAIKYIFDTVSRAKQ
HMC56         IYKNLPLSDTYQAVRNYVNNRLIEKLSSNRLLLEHLANSKISGNEYAIKYIFDTVSRAKQ
              ************************************************************

35000HP       EIFEQELNTELAPVSLDIMRRKAANILSSEHGSYKDGTLSIYDKPVKSAFHSRLKNNGEV
NZS1          EIFEQELNTELAPVSLDIMRRKAANILSSEHGSYKDGTLSIYDKPVKSAFHSRLKNNGEV
NZS2          EIFEQELNTELAPVSLDIMRRKAANILSSEHGSYKDGTLSIYDKPVKSAFHSRLKNNGEV
NZS3          EIFEQELNTELAPVSLDIMRRKAANILSSEHGSYKDGTLSIYDKPVKSAFHSRLKNNGEV
NZS4          EIFEQELNTELAPVSLDIMRRKAANILSSEHGSYKDGTLSIYDKPVKSAFHSRLKNNGEV
```

```
NZV1          EIFEQELNTELAPVSLDIMRRKAANILSSEHGSYKDGTLSIYDKPVKSAFHSRLKNNGEV
82-029362     EIFEQELNTELAPVSLDIMRRKAANILSSEHGSYKDGTLSIYDKPVKSAFHSRLKNNGEV
6644          EIFEQELNTELAPVSLDIMRRKAANILSSEHGSYKDGTLSIYDKPVKSAFHSRLKNNGEV
HD183         EIFEQELNTELAPVSLDIMRRKAANILSSEHGSYKDGTLSIYDKPVKSAFHSRLKNNGEV
HMC46         EIFEQELNTELAPVSLDIMRRKAANILSSEHGSYKDGTLSIYDKPVKSAFHSRLKNNGEV
HMC56         EIFEQELNTELAPVSLDIMRRKAANILSSEHGSYKDGTLSIYDKPVKSAFHSRLKNNGEV
              ************************************************************

35000HP       LNTIVHELTHHEQDALAKIIDNKGYDAKLFDKNNILYITGGLGYPKQALERDAFLSGDSV
NZS1          LNTIVHELTHHEQDALAKIIDNKGYDAKLFDKNNILYITGGLGYPKQALERDAFLSGDSV
NZS2          LNTIVHELTHHEQDALAKIIDNKGYDAKLFDKNNILYITGGLGYPKQALERDAFLSGDSV
NZS3          LNTIVHELTHHEQDALAKIIDNKGYDAKLFDKNNILYITGGLGYPKQALERDAFLSGDSV
NZS4          LNTIVHELTHHEQDALAKIIDNKGYDAKLFDKNNILYITGGLGYPKQALERDAFLSGDSV
NZV1          LNTIVHELTHHEQDALAKIIDNKGYDAKLFDKNNILYITGGLGYPKQALERDAFLSGDSV
82-029362     LNTIVHELTHHEQDALAKIIDNKGYDAKLFDKNNILYITGGLGYPKQALERDAFLSGDSV
6644          LNTIVHELTHHEQDALAKIIDNKGYDAKLFDKNNILYITGGLGYPKQALERDAFLSGDSV
HD183         LNTIVHELTHHEQDALAKIIDNKGYDAKLFDKNNILYITGGLGYPKQALERDAFLSGDSV
HMC46         LNTIVHELTHHEQDALAKIIDNKGYDAKLFDKNNILYITGGLGYPKQALERDAFLSGDSV
HMC56         LNTIVHELTHHEQDALAKIIDNKGYDAKLFDKNNILYITGGLGYPKQALERDAFLSGDSV
              ************************************************************

35000HP       SEAFMKKAKEYHERTKQERKDAKKDEARIAKLYKQWEQEEANKKSASLNGSSQSLDSRSE
NZS1          SEAFMKKAKEYHERTKQERKDAKKDEARIAKLYKQWEQEEANKKSASLNGSSQSLDSRSE
NZS2          SEAFMKKAKEYHERTKQERKDAKKDEARIAKLYKQWEQEEANKKSASLNGSSQSLDSRSE
NZS3          SEAFMKKAKEYHERTKQERKDAKKDEARIAKLYKQWEQEEANKKSASLNGSSQSLDSRSE
NZS4          SEAFMKKAKEYHERTKQERKDAKKDEARIAKLYKQWEQEEANKKSASLNGSSQSLDSRSE
NZV1          SEAFMKKAKEYHERTKQERKDAKKDEARIAKLYKQWEQEEANKKSASLNGSSQSLDSRSE
82-029362     SEAFMKKAKEYHERTKQERKDAKKDEARIAKLYKQWEQEEANKKSASLNGSSQSLDSRSE
6644          SEAFMKKAKEYHERTKQERKDAKKDEARIAKLYKQWEQEEANKKSASLNGSSQSLDSRSE
HD183         SEAFMKKAKEYHERTKQERKDAKKDEARIAKLYKQWEQEEANKKSASLNGSSQSLDSRSE
HMC46         SEAFMKKAKEYHERTKQERKDAKKDEARIAKLYKQWEQEEANKKSASLNGSSQSLDSRSE
HMC56         SEAFMKKAKEYHERTKQERKDAKKDEARIAKLYKQWEQEEANKKSASLNGSSQSLDSRSE
              ************************************************************

35000HP       VEFNRVSHKSVR*
NZS1          VEFNRVSHKSVR*
NZS2          VEFNRVSHKSVR*
NZS3          VEFNRVSHKSVR*
NZS4          VEFNRVSHKSVR*
NZV1          VEFNRVSHKSVR*
82-029362     VEFNRVSHKSVR*
6644          VEFNRVSHKSVR*
HD183         VEFNRVSHKSVR*
HMC46         VEFNRVSHKSVR*
HMC56         VEFNRVSHKSVR*
              *************
```

**Note:** Due to difficulty in sorting reads of *lspA1* and *lspA2* genes of class II strains, LspA1 sequences of class II strains were excluded from this analysis.

# 13) LspA2 (HD1156)

```
35000HP       MNNKRYKLIFSKVKNCLVPVAENIKSASGNSGSSSNSKIAEDQEEEPDSLACSLSPLSSS
NZS1          MNNKRYKLIFSKVKNCLVPVAENIKSASGNSGSSSNSKIAEDQEEEPDSLACSLSPLSSS
NZS2          MNNKRYKLIFSKVKNCLVPVAENIKSASGNSGSSSNSKIAEDQEEEPDSLACSLSPLSSS
NZS3          MNNKRYKLIFSKVKNCLVPVAENIKSASGNSGSSSNSKIAEDQEEEPDSLACSLSPLSSS
NZS4          MNNKRYKLIFSKVKNCLVPVAENIKSASGNSGSSSNSKIAEDQEEEPDSLACSLSPLSSS
NZV1          MNNKRYKLIFSKVKNCLVPVAENIKSASGNSGSSSNSKIAEDQEEEPDSLACSLSPLSSS
82-029362     MNNKRYKLIFSKVKNCLVPVAENIKSASGNSGSSSNSKIAEDQEEEPDSLACSLSPLSSS
6644          MNNKRYKLIFSKVKNCLVPVAENIKSASGNSGSSSNSKIAEDQEEEPDSLACSLSPLSSS
HD183         MNNKRYKLIFSKVKNCLVPVAENIKSASGNSGSSSNSKIAEDQEEEPDSLACSLSPLSSS
HMC46         MNNKRYKLIFSKVKNCLVPVAENIKSASGNSGSSSNSKIAEDQEEEPDSLACSLSPLSSS
HMC56         MNNKRYKLIFSKVKNCLVPVAENIKSASGNSGSSSNSKIAEDQEEEPDSLACSLSPLSSS
              ************************************************************

35000HP       IHLGLHNHSPLKVFKGKNLSVVLLSLMPAMQVWADSSNAIVDHSHGAKQTAVDERDPKNG
NZS1          IHLGLHNHSPLKVFKGKNLSVVLLSLMPAMQVWADSSNAIVDHSHGAKQTAVDERDPKNG
NZS2          IHLGLHNHSPLKVFKGKNLSVVLLSLMPAMQVWADSSNAIVDHSHGAKQTAVDERDPKNG
NZS3          IHLGLHNHSPLKVFKGKNLSVVLLSLMPAMQVWADSSNAIVDHSHGAKQTAVDERDPKNG
NZS4          IHLGLHNHSPLKVFKGKNLSVVLLSLMPAMQVWADSSNAIVDHSHGAKQTAVDERDPKNG
NZV1          IHLGLHNHSPLKVFKGKNLSVVLLSLMPAMQVWADSSNAIVDHSHGAKQTAVDERDPKNG
82-029362     IHLGLHNHSPLKVFKGKNLSVVLLSLMPAMQVWADSSNAIVDHSHGAKQTAVDERDPKNG
```

```
6644         IHLGLHNHSPLKVFKGKNLSVVLLSLMPAMQVWADSSNAIVDHSHGAKQTAVDERDPKNG
HD183        IHLGLHNHSPLKVFKGKNLSVVLLSLMPAMQVWADSSNAIVDHSHGAKQTAVDERDPKNG
HMC46        IHLGLHNHSPLKVFKGKNLSVVLLSLMPAMQVWADSSNAIVDHSHGAKQTAVDERDPKNG
HMC56        IHLGLHNHSPLKVFKGKNLSVVLLSLMPAMQVWADSSNAIVDHSHGAKQTAVDERDPKNG
             ************************************************************

35000HP      KEKVVVINIAKPDEQGISDNHFSKFNIPNSAVFNNSIKEGNSQLVGLLGENKNLGSQAAK
NZS1         KEKVVVINIAKPDEQGISDNHFSKFNIPNSAVFNNSIKEGNSQLVGLLGENKNLGSQAAK
NZS2         KEKVVVINIAKPDEQGISDNHFSKFNIPNSAVFNNSIKEGNSQLVGLLGENKNLGSQAAK
NZS3         KEKVVVINIAKPDEQGISDNHFSKFNIPNSAVFNNSIKEGNSQLVGLLGENKNLGSQAAK
NZS4         KEKVVVINIAKPDEQGISDNHFSKFNIPNSAVFNNSIKEGNSQLVGLLGENKNLGSQAAK
NZV1         KEKVVVINIAKPDEQGISDNHFSKFNIPNSAVFNNSIKEGNSQLVGLLGENKNLGSQAAK
82-029362    KEKVVVINIAKPDEQGISDNHFSKFNIPNSAVFNNSIKEGNSQLVGLLGENKNLGSQAAK
6644         KEKVVVINIAKPDEQGISDNHFSKFNIPNSAVFNNSIKEGNSQLVGLLGENKNLGSQAAK
HD183        KEKVVVINIAKPDEQGISDNHFSKFNIPNSAVFNNSIKEGNSQLVGLLGENKNLGSQAAK
HMC46        KEKVVVINIAKPDEQGISDNHFSKFNIPNSAVFNNSIKEGNSQLVGLLGENKNLGSQAAK
HMC56        KEKVVVINIAKPDEQGISDNHFSKFNIPNSAVFNNSIKEGNSQLVGLLGENKNLGSQAAK
             ************************************************************

35000HP      TIFNQVTGDQESKISGGLEVFGEKADLFIINPNGVTLNGVKTINTDRFVASTSEVVEPHI
NZS1         TIFNQVTGDQESKISGGLEVFGEKADLFIINPNGVTLNGVKTINTDRFVASTSEVVEPHI
NZS2         TIFNQVTGDQESKISGGLEVFGEKADLFIINPNGVTLNGVKTINTDRFVASTSEVVEPHI
NZS3         TIFNQVTGDQESKISGGLEVFGEKADLFIINPNGVTLNGVKTINTDRFVASTSEVVEPHI
NZS4         TIFNQVTGDQESKISGGLEVFGEKADLFIINPNGVTLNGVKTINTDRFVASTSEVVEPHI
NZV1         TIFNQVTGDQESKISGGLEVFGEKADLFIINPNGVTLNGVKTINTDRFVASTSEVVEPHI
82-029362    TIFNQVTGDQESKISGGLEVFGEKADLFIINPNGVTLNGVKTINTDRFVASTSEVVEPHI
6644         TIFNQVTGDQESKISGGLEVFGEKADLFIINPNGVTLNGVKTINTDRFVASTSEVVEPHI
HD183        TIFNQVTGDQESKISGGLEVFGEKADLFIINPNGVTLNGVKTINTDRFVASTSEVVEPHI
HMC46        TIFNQVTGDQESKISGGLEVFGEKADLFIINPNGVTLNGVKTINTDRFVASTSEVVEPHI
HMC56        TIFNQVTGDQESKISGGLEVFGEKADLFIINPNGVTLNGVKTINTDRFVASTSEVVEPHI
             ************************************************************

35000HP      KQLNVQRGKVIIGKDGVATNGLSHFDVVAKNIEQQGKVSIEGDSKPAKLANVTFAAGNLT
NZS1         KQLNVQRGKVIIGKDGVATNGLSHFDVVAKNIEQQGKVSIEGDSKPAKLANVTFAAGNLT
NZS2         KQLNVQRGKVIIGKDGVATNGLSHFDVVAKNIEQQGKVSIEGDSKPAKLANVTFAAGNLT
NZS3         KQLNVQRGKVIIGKDGVATNGLSHFDVVAKNIEQQGKVSIEGDSKPAKLANVTFAAGNLT
NZS4         KQLNVQRGKVIIGKDGVATNGLSHFDVVAKNIEQQGKVSIEGDSKPAKLANVTFAAGNLT
NZV1         KQLNVQRGKVIIGKDGVATNGLSHFDVVAKNIEQQGKVSIEGDSKPAKLANVTFAAGNLT
82-029362    KQLNVQRGKVIIGKDGVATNGLSHFDVVAKNIEQQGKVSIEGDSKPAKLANVTFAAGNLT
6644         KQLNVQRGKVIIGKDGVATNGLSHFDVVAKNIEQQGKVSIEGDSKPAKLANVTFAAGNLT
HD183        KQLNVQRGKVIIGKDGVATNGLSHFDVVAKNIEQQGKVSIEGDSKPAKLANVTFAAGNLT
HMC46        KQLNVQRGKVIIGKDGVATNGLSHFDVVAKNIEQQGKVSIEGDSKPAKLANVTFAAGNLT
HMC56        KQLNVQRGKVIIGKDGVATNGLSHFDVVAKNIEQQGKVSIEGDSKPAKLANVTFAAGNLT
             ************************************************************

35000HP      YDVNTRDVNRNTNPKKPITDNTRKDNIAISGESAGSMYGRNIKFIVTDKGAGVNHQGVIF
NZS1         YDVNTRDVNRNTNPKKPITDNTRKDNIAISGESAGSMYGRNIKFIVTDKGAGVNHQGVIF
NZS2         YDVNTRDVNRNTNPKKPITDNTRKDNIAISGESAGSMYGRNIKFIVTDKGAGVNHQGVIF
NZS3         YDVNTRDVNRNTNPKKPITDNTRKDNIAISGESAGSMYGRNIKFIVTDKGAGVNHQGVIF
NZS4         YDVNTRDVNRNTNPKKPITDNTRKDNIAISGESAGSMYGRNIKFIVTDKGAGVNHQGVIF
NZV1         YDVNTRDVNRNTNPKKPITDNTRKDNIAISGESAGSMYGRNIKFIVTDKGAGVNHQGVIF
82-029362    YDVNTRDVNRNTNPKKPITDNTRKDNIAISGESAGSMYGRNIKFIVTDKGAGVNHQGVIF
6644         YDVNTRDVNRNTNPKKPITDNTRKDNIAISGESAGSMYGRNIKFIVTDKGAGVNHQGVIF
HD183        YDVNTRDVNRNTNPKKPITDNTRKDNIAISGESAGSMYGRNIKFIVTDKGAGVNHQGVIF
HMC46        YDVNTRDVNRNTNPKKPITDNTRKDNIAISGESAGSMYGRNIKFIVTDKGAGVNHQGVIF
HMC56        YDVNTRDVNRNTNPKKPITDNTRKDNIAISGESAGSMYGRNIKFIVTDKGAGVNHQGVIF
             ************************************************************

35000HP      AEDDINILTDDGNSRLNKVYADYVRVVGKDIELANNGQIHADQQLILNATGHVKLNDGSS
NZS1         AEDDINILTDDGNSRLNKVYADYVRVVGKDIELANNGQIHADQQLILNATGHVKLNDGSS
NZS2         AEDDINILTDDGNSRLNKVYADYVRVVGKDIELANNGQIHADQQLILNATGHVKLNDGSS
NZS3         AEDDINILTDDGNSRLNKVYADYVRVVGKDIELANNGQIHADQQLILNATGHVKLNDGSS
NZS4         AEDDINILTDDGNSRLNKVYADYVRVVGKDIELANNGQIHADQQLILNATGHVKLNDGSS
NZV1         AEDDINILTDDGNSRLNKVYADYVRVVGKDIELANNGQIHADQQLILNATGHVKLNDGSS
82-029362    AEDDINILTDDGNSRLNKVYADYVRVVGKDIELANNGQIHADQQLILNATGHVKLNDGSS
6644         AEDDINILTDDGNSRLNKVYADYVRVVGKDIELANNGQIHADQQLILNATGHVKLNDGSS
HD183        AEDDINILTDDGNSRLNKVYADYVRVVGKDIELANNGQIHADQQLILNATGHVKLNDGSS
HMC46        AEDDINILTDDGNSRLNKVYADYVRVVGKDIELANNGQIHADQQLILNATGHVKLNDGSS
HMC56        AEDDINILTDDGNSRLNKVYADYVRVVGKDIELANNGQIHADQQLILNATGHVKLNDGSS
             ************************************************************

35000HP      VISNNNLGISALNLTLENATVSANNLSFRVTNDTKLNNLSKVSARAADLQSGNLNLDKAS
NZS1         VISNNNLGISALNLTLENATVSANNLSFRVTNDTKLNNLSKVSARAADLQSGNLNLDKAS
```

```
NZS2          VISNNNLGISALNLTLENATVSANNLSFRVTNDTKLNNLSKVSARAADLQSGNLNLDKAS
NZS3          VISNNNLGISALNLTLENATVSANNLSFRVTNDTKLNNLSKVSARAADLQSGNLNLDKAS
NZS4          VISNNNLGISALNLTLENATVSANNLSFRVTNDTKLNNLSKVSARAADLQSGNLNLDKAS
NZV1          VISNNNLGISALNLTLENATVSANNLSFRVTNDTKLNNLSKVSARAADLQSGNLNLDKAS
82-029362     VISNNNLGISALNLTLENATVSANNLSFRVTNDTKLNNLSKVSARAADLQSGNLNLDKAS
6644          VISNNNLGISALNLTLENATVSANNLSFRVTNDTKLNNLSKVSARAADLQSGNLNLDKAS
HD183         VISNNNLGISALNLTLENATVSANNLSFRVTNDTKLNNLSKVSARAADLQSGNLNLDKAS
HMC46         VISNNNLGISALNLTLENATVSANNLSFRVTNDTKLNNLSKVSARAADLQSGNLNLDKAS
HMC56         VISNNNLGISALNLTLENATVSANNLSFRVTNDTKLNNLSKVSARAADLQSGNLNLDKAS
              ************************************************************

35000HP       VLAHKLTLNISNDVSLNNQSKLSANNLKIKKVRDLNLNNSELSANNLTLNTSNNITLKNK
NZS1          VLAHKLTLNISNDVSLNNQSKLSANNLKIKKVRDLNLNNSELSANNLTLNTSNNITLKNK
NZS2          VLAHKLTLNISNDVSLNNQSKLSANNLKIKKVRDLNLNNSELSANNLTLNTSNNITLKNK
NZS3          VLAHKLTLNISNDVSLNNQSKLSANNLKIKKVRDLNLNNSELSANNLTLNTSNNITLKNK
NZS4          VLAHKLTLNISNDVSLNNQSKLSANNLKIKKVRDLNLNNSELSANNLTLNTSNNITLKNK
NZV1          VLAHKLTLNISNDVSLNNQSKLSANNLKIKKVRDLNLNNSELSANNLTLNTSNNITLKNK
82-029362     VLAHKLTLNISNDVSLNNQSKLSANNLKIKKVRDLNLNNSELSANNLTLNTSNNITLKNK
6644          VLAHKLTLNISNDVSLNNQSKLSANNLKIKKVRDLNLNNSELSANNLTLNTSNNITLKNK
HD183         VLAHKLTLNISNDVSLNNQSKLSANNLKIKKVRDLNLNNSELSANNLTLNTSNNITLKNK
HMC46         VLAHKLTLNISNDVSLNNQSKLSANNLKIKKVRDLNLNNSELSANNLTLNTSNNITLKNK
HMC56         VLAHKLTLNISNDVSLNNQSKLSANNLKIKKVRDLNLNNSELSANNLTLNTSNNITLKNK
              ************************************************************

35000HP       SKFTAGNMTLNVTNNVTLNNDSELAANNLTLNVTKNVTLNDASKLSANKLDLNVTDNVTL
NZS1          SKFTAGNMTLNVTNNVTLNNDSELAANNLTLNVTKNVTLNDASKLSANKLDLNVTDNVTL
NZS2          SKFTAGNMTLNVTNNVTLNNDSELAANNLTLNVTKNVTLNDASKLSANKLDLNVTDNVTL
NZS3          SKFTAGNMTLNVTNNVTLNNDSELAANNLTLNVTKNVTLNDASKLSANKLDLNVTDNVTL
NZS4          SKFTAGNMTLNVTNNVTLNNDSELAANNLTLNVTKNVTLNDASKLSANKLDLNVTDNVTL
NZV1          SKFTAGNMTLNVTNNVTLNNDSELAANNLTLNVTKNVTLNDASKLSANKLDLNVTDNVTL
82-029362     SKFTAGNMTLNVTNNVTLNNDSELAANNLTLNVTKNVTLNDASKLSANKLDLNVTDNVTL
6644          SKFTAGNMTLNVTNNVTLNNDSELAANNLTLNVTKNVTLNDASKLSANKLDLNVTDNVTL
HD183         SKFTAGNMTLNVTNNVTLNNDSELAANNLTLNVTKNVTLNDASKLSANKLDLNVTDNVTL
HMC46         SKFTAGNMTLNVTNNVTLNNDSELAANNLTLNVTKNVTLNDASKLSANKLDLNVTDNVTL
HMC56         SKFTAGNMTLNVTNNVTLNNDSELAANNLTLNVTKNVTLNDASKLSANKLDLNVTDNVTL
              ************************************************************

35000HP       NSKSTLSAGELTFKKVKNVTLNNDSELAANNLSLNASHNVTLNNKSKLSAQKADIKAVNL
NZS1          NSKSTLSAGELTFKKVKNVTLNNDSELAANNLSLNASHNVTLNNKSKLSAQKADIKAVNL
NZS2          NSKSTLSAGELTFKKVKNVTLNNDSELAANNLSLNASHNVTLNNKSKLSAQKADIKAVNL
NZS3          NSKSTLSAGELTFKKVKNVTLNNDSELAANNLSLNASHNVTLNNKSKLSAQKADIKAVNL
NZS4          NSKSTLSAGELTFKKVKNVTLNNDSELAANNLSLNASHNVTLNNKSKLSAQKADIKAVNL
NZV1          NSKSTLSAGELTFKKVKNVTLNNDSELAANNLSLNASHNVTLNNKSKLSAQKADIKAVNL
82-029362     NSKSTLSAGELTFKKVKNVTLNNDSELAANNLSLNASHNVTLNNKSKLSAQKADIKAVNL
6644          NSKSTLSAGELTFKKVKNVTLNNDSELAANNLSLNASHNVTLNNKSKLSAQKADIKAVNL
HD183         NSKSTLSAGELTFKKVKNVTLNNDSELAANNLSLNASHNVTLNNKSKLSAQKADIKAVNL
HMC46         NSKSTLSAGELTFKKVKNVTLNNDSELAANNLSLNASHNVTLNNKSKLSAQKADIKAVNL
HMC56         NSKSTLSAGELTFKKVKNVTLNNDSELAANNLSLNASHNVTLNNKSKLSAQKADIKAVNL
              ************************************************************

35000HP       TLNDTTELTAKNLDINSTTITNNGTIAGIFANITTEKLNNKEKALILAEQNLNFTVNGSH
NZS1          TLNDTTELTAKNLDINSTTITNNGTIAGIFANITTEKLNNKEKALILAEQNLNFTVNGSH
NZS2          TLNDTTELTAKNLDINSTTITNNGTIAGIFANITTEKLNNKEKALILAEQNLNFTVNGSH
NZS3          TLNDTTELTAKNLDINSTTITNNGTIAGIFANITTEKLNNKEKALILAEQNLNFTVNGSH
NZS4          TLNDTTELTAKNLDINSTTITNNGTIAGIFANITTEKLNNKEKALILAEQNLNFTVNGSH
NZV1          TLNDTTELTAKNLDINSTTITNNGTIAGIFANITTEKLNNKEKALILAEQNLNFTVNGSH
82-029362     TLNDTTELTAKNLDINSTTITNNGTIAGIFANITTEKLNNKEKALILAEQNLNFTVNGSH
6644          TLNDTTELTAKNLDINSTTITNNGTIAGIFANITTEKLNNKEKALILAEQNLNFTVNGSH
HD183         TLNDTTELTAKNLDINSTTITNNGTIAGIFANITTEKLNNKEKALILAEQNLNFTVNGSH
HMC46         TLNDTTELTAKNLDINSTTITNNGTIAGIFANITTEKLNNKEKALILAEQNLNFTVNGSH
HMC56         TLNDTTELTAKNLDINSTTITNNGTIAGIFANITTEKLNNKEKALILAEQNLNFTVNGSH
              ************************************************************

35000HP       YENKGDIVSKDKATVTFSKNSDFTSNGSKLVNAQNQLKVNVNNFTISQGDDITLIGNVTL
NZS1          YENKGDIVSKDKATVTFSKNSDFTSNGSKLVNAQNQLKVNVNNFTISQGDDITLIGNVTL
NZS2          YENKGDIVSKDKATVTFSKNSDFTSNGSKLVNAQNQLKVNVNNFTISQGDDITLIGNVTL
NZS3          YENKGDIVSKDKATVTFSKNSDFTSNGSKLVNAQNQLKVNVNNFTISQGDDITLIGNVTL
NZS4          YENKGDIVSKDKATVTFSKNSDFTSNGSKLVNAQNQLKVNVNNFTISQGDDITLIGNVTL
NZV1          YENKGDIVSKDKATVTFSKNSDFTSNGSKLVNAQNQLKVNVNNFTISQGDDITLIGNVTL
82-029362     YENKGDIVSKDKATVTFSKNSDFTSNGSKLVNAQNQLKVNVNNFTISQGDDITLIGNVTL
6644          YENKGDIVSKDKATVTFSKNSDFTSNGSKLVNAQNQLKVNVNNFTISQGDDITLIGNVTL
HD183         YENKGDIVSKDKATVTFSKNSDFTSNGSKLVNAQNQLKVNVNNFTISQGDDITLIGNVTL
HMC46         YENKGDIVSKDKATVTFSKNSDFTSNGSKLVNAQNQLKVNVNNFTISQGDDITLIGNVTL
```

```
HMC56         YENKGDIVSKDKATVTFSKNSDFTSNGSKLVNAQNQLKVNVNNFTISQGDDITLIGNVTL
              ************************************************************

35000HP       NASGTFTNSGNLTTVKTLDVGDIQNFTNKGNLTVGEDLHIKSKTKITNDGKLISIKNLNI
NZS1          NASGTFTNSGNLTTVKTLDVGDIQNFTNKGNLTVGEDLHIKSKTKITNDGKLISIKNLNI
NZS2          NASGTFTNSGNLTTVKTLDVGDIQNFTNKGNLTVGEDLHIKSKTKITNDGKLISIKNLNI
NZS3          NASGTFTNSGNLTTVKTLDVGDIQNFTNKGNLTVGEDLHIKSKTKITNDGKLISIKNLNI
NZS4          NASGTFTNSGNLTTVKTLDVGDIQNFTNKGNLTVGEDLHIKSKTKITNDGKLISIKNLNI
NZV1          NASGTFTNSGNLTTVKTLDVGDIQNFTNKGNLTVGEDLHIKSKTKITNDGKLISIKNLNI
82-029362     NASGTFTNSGNLTTVKTLDVGDIQNFTNKGNLTVGEDLHIKSKTKITNDGKLISIKNLNI
6644          NASGTFTNSGNLTTVKTLDVGDIQNFTNKGNLTVGEDLHIKSKTKITNDGKLISIKNLNI
HD183         NASGTFTNSGNLTTVKTLDVGDIQNFTNKGNLTVGEDLHIKSKTKITNDGKLISIKNLNI
HMC46         NASGTFTNSGNLTTVKTLDVGDIQNFTNKGNLTVGEDLHIKSKTKITNDGKLISIKNLNI
HMC56         NASGTFTNSGNLTTVKTLDVGDIQNFTNKGNLTVGEDLHIKSKTKITNDGKLISIKNLNI
              ************************************************************

35000HP       SSEADFINNGTLLGIEALKIATKGNFTNKEKAILASNSLLDISVAEGKKTFNNGTIESGK
NZS1          SSEADFINNGTLLGIEALKIATKGNFTNKEKAILASNSLLDISVAEGKKTFNNGTIESGK
NZS2          SSEADFINNGTLLGIEALKIATKGNFTNKEKAILASNSLLDISVAEGKKTFNNGTIESGK
NZS3          SSEADFINNGTLLGIEALKIATKGNFTNKEKAILASNSLLDISVAEGKKTFNNGTIESGK
NZS4          SSEADFINNGTLLGIEALKIATKGNFTNKEKAILASNSLLDISVAEGKKTFNNGTIESGK
NZV1          SSEADFINNGTLLGIEALKIATKGNFTNKEKAILASNSLLDISVAEGKKTFNNGTIESGK
82-029362     SSEADFINNGTLLGIEALKIATKGNFTNKEKAILASNSLLDISVAEGKKTFNNGTIESGK
6644          SSEADFINNGTLLGIEALKIATKGNFTNKEKAILASNSLLDISVAEGKKTFNNGTIESGK
HD183         SSEADFINNGTLLGIEALKIATKGNFTNKEKAILASNSLLDISVAEGKKTFNNGTIESGK
HMC46         SSEADFINNGTLLGIEALKIATKGNFTNKEKAILASNSLLDISVAEGKKTFNNGTIESGK
HMC56         SSEADFINNGTLLGIEALKIATKGNFTNKEKAILASNSLLDISVAEGKKTFNNGTIESGK
              ************************************************************

35000HP       NLNITNTGAFLNVDNATIRSFGVLNITSTGNVSNNGTLISNERLNITSAANFTNESNGTV
NZS1          NLNITNTGAFLNVDNATIRSFGVLNITSTGNVSNNGTLISNERLNITSAANFTNESNGTV
NZS2          NLNITNTGAFLNVDNATIRSFGVLNITSTGNVSNNGTLISNERLNITSAANFTNESNGTV
NZS3          NLNITNTGAFLNVDNATIRSFGVLNITSTGNVSNNGTLISNERLNITSAANFTNESNGTV
NZS4          NLNITNTGAFLNVDNATIRSFGVLNITSTGNVSNNGTLISNERLNITSAANFTNESNGTV
NZV1          NLNITNTGAFLNVDNATIRSFGVLNITSTGNVSNNGTLISNERLNITSAANFTNESNGTV
82-029362     NLNITNTGAFLNVDNATIRSFGVLNITSTGNVSNNGTLISNERLNITSAANFTNESNGTV
6644          NLNITNTGAFLNVDNATIRSFGVLNITSTGNVSNNGTLISNERLNITSAANFTNESNGTV
HD183         NLNITNTGAFLNVDNATIRSFGVLNITSTGNVSNNGTLISNERLNITSAANFTNESNGTV
HMC46         NLNITNTGAFLNVDNATIRSFGVLNITSTGNVSNNGTLISNERLNITSAANFTNESNGTV
HMC56         NLNITNTGAFLNVDNATIRSFGVLNITSTGNVSNNGTLISNERLNITSAANFTNESNGTV
              ************************************************************

35000HP       MSNGLLNIIAKQGNITNKNLIASRQQLNLTAVADNITNDSNISNKIAVLHSLGNISLNSK
NZS1          MSNGLLNIIAKQGNITNKNLIASRQQLNLTAVADNITNDSNISNKIAVLHSLGNISLNSK
NZS2          MSNGLLNIIAKQGNITNKNLIASRQQLNLTAVADNITNDSNISNKIAVLHSLGNISLNSK
NZS3          MSNGLLNIIAKQGNITNKNLIASRQQLNLTAVADNITNDSNISNKIAVLHSLGNISLNSK
NZS4          MSNGLLNIIAKQGNITNKNLIASRQQLNLTAVADNITNDSNISNKIAVLHSLGNISLNSK
NZV1          MSNGLLNIIAKQGNITNKNLIASRQQLNLTAVADNITNDSNISNKIAVLHSLGNISLNSK
82-029362     MSNGLLNIIAKQGNITNKNLIASRQQLNLTAVADNITNDSNISNKIAVLHSLGNISLNSK
6644          MSNGLLNIIAKQGNITNKNLIASRQQLNLTAVADNITNDSNISNKIAVLHSLGNISLNSK
HD183         MSNGLLNIIAKQGNITNKNLIASRQQLNLTAVADNITNDSNISNKIAVLHSLGNISLNSK
HMC46         MSNGLLNIIAKQGNITNKNLIASRQQLNLTAVADNITNDSNISNKIAVLHSLGNISLNSK
HMC56         MSNGLLNIIAKQGNITNKNLIASRQQLNLTAVADNITNDSNISNKIAVLHSLGNISLNSK
              ************************************************************

35000HP       DQVYNLGEIYAGNNISVKAHQLKNDVKLMGDITTKTKEGQASYKLYQASNGGHFGNDGSS
NZS1          DQVYNLGEIYAGNNISVKAHQLKNDVKLMGDITTKTKEGQASYKLYQASNGGHFGNDGSS
NZS2          DQVYNLGEIYAGNNISVKAHQLKNDVKLMGDITTKTKEGQASYKLYQASNGGHFGNDGSS
NZS3          DQVYNLGEIYAGNNISVKAHQLKNDVKLMGDITTKTKEGQASYKLYQASNGGHFGNDGSS
NZS4          DQVYNLGEIYAGNNISVKAHQLKNDVKLMGDITTKTKEGQASYKLYQASNGGHFGNDGSS
NZV1          DQVYNLGEIYAGNNISVKAHQLKNDVKLMGDITTKTKEGQASYKLYQASNGGHFGNDGSS
82-029362     DQVYNLGEIYAGNNISVKAHQLKNDVKLMGDITTKTKEGQASYKLYQASNGGHFGNDGSS
6644          DQVYNLGEIYAGNNISVKAHQLKNDVKLMGDITTKTKEGQASYKLYQASNGGHFGNDGSS
HD183         DQVYNLGEIYAGNNISVKAHQLKNDVKLMGDITTKTKEGQASYKLYQASNGGHFGNDGSS
HMC46         DQVYNLGEIYAGNNISVKAHQLKNDVKLMGDITTKTKEGQASYKLYQASNGGHFGNDGSS
HMC56         DQVYNLGEIYAGNNISVKAHQLKNDVKLMGDITTKTKEGQASYKLYQASNGGHFGNDGSS
              ************************************************************

35000HP       GYSEGDLNIKGKFADLDNKLTVQRIGKIYAGRDLTFNKSNAGGKSEIINRGTINVKNKLS
NZS1          GYSEGDLNIKGKFADLDNKLTVQRIGKIYAGRDLTFNKSNAGGKSEIINRGTINVKNKLS
NZS2          GYSEGDLNIKGKFADLDNKLTVQRIGKIYAGRDLTFNKSNAGGKSEIINRGTINVKNKLS
NZS3          GYSEGDLNIKGKFADLDNKLTVQRIGKIYAGRDLTFNKSNAGGKSEIINRGTINVKNKLS
NZS4          GYSEGDLNIKGKFADLDNKLTVQRIGKIYAGRDLTFNKSNAGGKSEIINRGTINVKNKLS
```

```
NZV1           GYSEGDLNIKGKFADLDNKLTVQRIGKIYAGRDLTFNKSNAGGKSEIINRGTINVKNKLS
82-029362      GYSEGDLNIKGKFADLDNKLTVQRIGKIYAGRDLTFNKSNAGGKSEIINRGTINVKNKLS
6644           GYSEGDLNIKGKFADLDNKLTVQRIGKIYAGRDLTFNKSNAGGKSEIINRGTINVKNKLS
HD183          GYSEGDLNIKGKFADLDNKLTVQRIGKIYAGRDLTFNKSNAGGKSEIINRGTINVKNKLS
HMC46          GYSEGDLNIKGKFADLDNKLTVQRIGKIYAGRDLTFNKSNAGGKSEIINRGTINVKNKLS
HMC56          GYSEGDLNIKGKFADLDNKLTVQRIGKIYAGRDLTFNKSNAGGKSEIINRGTINVKNKLS
               ************************************************************

35000HP        YDSDVSFENNMQSQKVDLYTKIFEAKSDIELTFKTNGTHPVYLNFKSNNNEKKYRNSENT
NZS1           YDSDVSFENNMQSQKVDLYTKIFEAKSDIELTFKTNGTHPVYLNFKSNNNEKKYRNSENT
NZS2           YDSDVSFENNMQSQKVDLYTKIFEAKSDIELTFKTNGTHPVYLNFKSNNNEKKYRNSENT
NZS3           YDSDVSFENNMQSQKVDLYTKIFEAKSDIELTFKTNGTHPVYLNFKSNNNEKKYRNSENT
NZS4           YDSDVSFENNMQSQKVDLYTKIFEAKSDIELTFKTNGTHPVYLNFKSNNNEKKYRNSENT
NZV1           YDSDVSFENNMQSQKVDLYTKIFEAKSDIELTFKTNGTHPVYLNFKSNNNEKKYRNSENT
82-029362      YDSDVSFENNMQSQKVDLYTKIFEAKSDIELTFKTNGTHPVYLNFKSNNNEKKYRNSENT
6644           YDSDVSFENNMQSQKVDLYTKIFEAKSDIELTFKTNGTHPVYLNFKSNNNEKKYRNSENT
HD183          YDSDVSFENNMQSQKVDLYTKIFEAKSDIELTFKTNGTHPVYLNFKSNNNEKKYRNSENT
HMC46          YDSDVSFENNMQSQKVDLYTKIFEAKSDIELTFKTNGTHPVYLNFKSNNNEKKYRNSENT
HMC56          YDSDVSFENNMQSQKVDLYTKIFEAKSDIELTFKTNGTHPVYLNFKSNNNEKKYRNSENT
               ************************************************************

35000HP        KNFKSIGDLINEALSDSAPEAIEAYYSGSSSNYINPVSYLAALGNANNSSNPHYLNTALK
NZS1           KNFKSIGDLINEALSDSAPEAIEAYYSGSSSNYINPVSYLAALGNANNSSNPHYLNTALK
NZS2           KNFKSIGDLINEALSDSAPEAIEAYYSGSSSNYINPVSYLAALGNANNSSNPHYLNTALK
NZS3           KNFKSIGDLINEALSDSAPEAIEAYYSGSSSNYINPVSYLAALGNANNSSNPHYLNTALK
NZS4           KNFKSIGDLINEALSDSAPEAIEAYYSGSSSNYINPVSYLAALGNANNSSNPHYLNTALK
NZV1           KNFKSIGDLINEALSDSAPEAIEAYYSGSSSNYINPVSYLAALGNANNSSNPHYLNTALK
82-029362      KNFKSIGDLINEALSDSAPEAIEAYYSGSSSNCINPVSYLAALGNANNSSNPHYLNTALK
6644           KNFKSIGDLINEALSDSAPEAIEAYYSGSSSNCINPVSYLAALGNANNSSNPHYLNTALK
HD183          KNFKSIGDLINEALSDSAPEAIEAYYSGSSSNYINPVSYLAALGNANNSSNPHYLNTALK
HMC46          KNFKSIGDLINEALSDSAPEAIEAYYSGSSSNCINPVSYLAALGNANNSSNPHYLNTALK
HMC56          KNFKSIGDLINEALSDSAPEAIEAYYSGSSSNCINPVSYLAALGNANNSSNPHYLNTALK
               ******************************** ***************************

35000HP        HILGNGWQDDLKKQENIKVLKQKWEDFKKDKGASKMLDLYPNTDKEKAKIFAGIIRNGND
NZS1           HILGNGWQDDLKKQENIKVLKQKWEDFKKDKGASKMLDLYPNTDKEKAKIFAGIIRNGND
NZS2           HILGNGWQDDLKKQENIKVLKQKWEDFKKDKGASKMLDLYPNTDKEKAKIFAGIIRNGND
NZS3           HILGNGWQDDLKKQENIKVLKQKWEDFKKDKGASKMLDLYPNTDKEKAKIFAGIIRNGND
NZS4           HILGNGWQDDLKKQENIKVLKQKWEDFKKDKGASKMLDLYPNTDKEKAKIFAGIIRNGND
NZV1           HILGNGWQDDLKKQENIKVLKQKWEDFKKDKGASKMLDLYPNTDKEKAKIFAGIIRNGND
82-029362      HILGNGWQDDLKKQENIKVLKQKWEDFKKDKGASKMLDLYPNTDKEKAKIFAGIIRNGND
6644           HILGNGWQDDLKKQENIKVLKQKWEDFKKDKGASKMLDLYPNTDKEKAKIFAGIIRNGND
HD183          HILGNGWQDDLKKQENIKVLKQKWEDFKKDKGASKMLDLYPNTDKEKAKIFAGIIRNGND
HMC46          HILGNGWQDDLKKQENIKVLKQKWEDFKKDKGASKMLDLYPNTDKEKAKIFAGIIRNGND
HMC56          HILGNGWQDDLKKQENIKVLKQKWEDFKKDKGASKMLDLYPNTDKEKAKIFAGIIRNGND
               ************************************************************

35000HP        TISDVESEDFKKKYSKFQNGEWAKNDTGTDSYDSTKASEKYKKVENVDHKENIDEHKLNI
NZS1           TISDVESEDFKKKYSKFQNGEWAKNDTGTDSYDSTKASEKYKKVENVDHKENIDEHKLNI
NZS2           TISDVESEDFKKKYSKFQNGEWAKNDTGTDSYDSTKASEKYKKVENVDHKENIDEHKLNI
NZS3           TISDVESEDFKKKYSKFQNGEWAKNDTGTDSYDSTKASEKYKKVENVDHKENIDEHKLNI
NZS4           TISDVESEDFKKKYSKFQNGEWAKNDTGTDSYDSTKASEKYKKVENVDHKENIDEHKLNI
NZV1           TISDVESEDFKKKYSKFQNGEWAKNDTGTDSYDSTKASEKYKKVENVDHKENIDEHKLNI
82-029362      TISDVESEDFKKKYSKFQNGEWAKNDTGTDSYDSTKASEKYKKVENVDHKENIDEHKLNI
6644           TISDVESEDFKKKYSKFQNGEWAKNDTGTDSYDSTKASEKYKKVENVDHKENIDEHKLNI
HD183          TISDVESEDFKKKYSKFQNGEWAKNDTGTDSYDSTKASEKYKKVENVDHKENIDEHKLNI
HMC46          TISDVESEDFKKKYSKFQNGEWAKNDTGTDSYDSTKASEKYKKVENVDHKENIDEHKLNI
HMC56          TISDVESEDFKKKYSKFQNGEWAKNDTGTDSYDSTKASEKYKKVENVDHKENIDEHKLNI
               ************************************************************

35000HP        GKHEITVPGVSFENLNNKNMDHQPDKLGEIDKSIISELLAQPVYTEKSAARDSDPRVNQN
NZS1           GKHEITVPGVSFENLNNKNMDHQPDKLGEIDKSIISELLAQPVYTEKSAARDSDPRVNQN
NZS2           GKHEITVPGVSFENLNNKNMDHQPDKLGEIDKSIISELLAQPVYTEKSAARDSDPRVNQN
NZS3           GKHEITVPGVSFENLNNKNMDHQPDKLGEIDKSIISELLAQPVYTEKSAARDSDPRVNQN
NZS4           GKHEITVPGVSFENLNNKNMDHQPDKLGEIDKSIISELLAQPVYTEKSAARDSDPRVNQN
NZV1           GKHEITVPGVSFENLNNKNMDHQPDKLGEIDKSIISELLAQPVYTEKSAARDSDPRVNQN
82-029362      GKHEITVPGVSFENLNNKNMDHQPDKLGEIDKSIISELLAQPVYTEKSAARDSDPRVNQN
6644           GKHEITVPGVSFENLNNKNMDHQPDKLGEIDKSIISELLAQPVYTEKSAARDSDPRVNQN
HD183          GKHEITVPGVSFENLNNKNMDHQPDKLGEIDKSIISELLAQPVYTEKSAARDSDPRVNQN
HMC46          GKHEITVPGVSFENLNNKNMDHQPDKLGEIDKSIISELLAQPVYTEKSAARDSDPRVNQN
HMC56          GKHEITVPGVSFENLNNKNMDHQPDKLGEIDKSIISELLAQPVYTEKSAARDSDPRVNQN
               ************************************************************
```

```
35000HP         DKEALDNLYRTRLSYINQNNYLGAKYFFNQLDTEDDKLKGIKRIGDNYFEHQLITRLIEK
NZS1            DKEALDNLYRTRLSYINQNNYLGAKYFFNQLDTEDDKLKGIKRIGDNYFEHQLITRLIEK
NZS2            DKEALDNLYRTRLSYINQNNYLGAKYFFNQLDTEDDKLKGIKRIGDNYFEHQLITRLIEK
NZS3            DKEALDNLYRTRLSYINQNNYLGAKYFFNQLDTEDDKLKGIKRIGDNYFEHQLITRLIEK
NZS4            DKEALDNLYRTRLSYINQNNYLGAKYFFNQLDTEDDKLKGIKRIGDNYFEHQLITRLIEK
NZV1            DKEALDNLYRTRLSYINQNNYLGAKYFFNQLDTEDDKLKGIKRIGDNYFEHQLITRLIEK
82-029362       DKEALDNLYRTRLSYINQNNYLGAKYFFNQLDTEDDKLKGIKRIGDNYFEYQLITRLIEK
6644            DKEALDNLYRTRLSYINQNNYLGAKYFFNQLDTEDDKLKGIKRIGDNYFEYQLITRLIEK
HD183           DKEALDNLYRTRLSYINQNNYLGAKYFFNQLDTEDDKLKGIKRIGDNYFEHQLITRLIEK
HMC46           DKEALDNLYRTRLSYINQNNYLGAKYFFNQLDTEDDKLKGIKRIGDNYFEYQLITRLIEK
HMC56           DKEALDNLYRTRLSYINQNNYLGAKYFFNQLDTEDDKLKGIKRIGDNYFEYQLITRLIEK
                **************************************************:*********

35000HP         VADNHLTLKHGLHDIALVKKLIDSASIQAKDLNLKVGEALTKEQKDNLKEDIVWYVKTEV
NZS1            VADNHLTLKHGLHDIALVKKLIDSASIQAKDLNLKVGEALTKEQKDNLKEDIVWYVKTEV
NZS2            VADNHLTLKHGLHDIALVKKLIDSASIQAKDLNLKVGEALTKEQKDNLKEDIVWYVKTEV
NZS3            VADNHLTLKHGLHDIALVKKLIDSASIQAKDLNLKVGEALTKEQKDNLKEDIVWYVKTEV
NZS4            VADNHLTLKHGLHDIALVKKLIDSASIQAKDLNLKVGEALTKEQKDNLKEDIVWYVKTEV
NZV1            VADNHLTLKHGLHDIALVKKLIDSASIQAKDLNLKVGEALTKEQKDNLKEDIVWYVKTEV
82-029362       VADNHLTLKHGLHDIALVKKLIDSASIQAKDLNLKVGEALTKEQKDNLKEDIVWYVKTEV
6644            VADNHLTLKHGLHDIALVKKLIDSASIQAKDLNLKVGEALTKEQKDNLKEDIVWYVKTEV
HD183           VADNHLTLKHGLHDIALVKKLIDSASIQAKDLNLKVGEALTKEQKDNLKEDIVWYVKTEV
HMC46           VADNHLTLKHGLHDIALVKKLIDSASIQAKDLNLKVGEALTKEQKDNLKEDIVWYVKTEV
HMC56           VADNHLTLKHGLHDIALVKKLIDSASIQAKDLNLKVGEALTKEQKDNLKEDIVWYVKTEV
                ************************************************************

35000HP         NGQEVLVPQVYLAKQTIEEVEKQRGVGTGQIRAGIIDVKVDDVRNTGTIAGYAVGLEAKN
NZS1            NGQEVLVPQVYLAKQTIEEVEKQRGVGTGQIRAGIIDVKVDDVRNTGTIAGYAVGLEAKN
NZS2            NGQEVLVPQVYLAKQTIEEVEKQRGVGTGQIRAGIIDVKVDDVRNTGTIAGYAVGLEAKN
NZS3            NGQEVLVPQVYLAKQTIEEVEKQRGVGTGQIRAGIIDVKVDDVRNTGTIAGYAVGLEAKN
NZS4            NGQEVLVPQVYLAKQTIEEVEKQRGVGTGQIRAGIIDVKVDDVRNTGTIAGYAVGLEAKN
NZV1            NGQEVLVPQVYLAKQTIEEVEKQRGVGTGQIRAGIIDVKVDDVRNTGTIAGYAVGLEAKN
82-029362       NGQEVLVPQVYLAKQTIEEVEKQRGVGTGQIRAGIIDVKVDDVRNTGTIAGYAVGLEAKN
6644            NGQEVLVPQVYLAKQTIEEVEKQRGVGTGQIRAGIIDVKVDDVRNTGTIAGYAVGLEAKN
HD183           NGQEVLVPQVYLAKQTIEEVEKQRGVGTGQIRAGIIDVKVDDVRNTGTIAGYAVGLEAKN
HMC46           NGQEVLVPQVYLAKQTIEEVEKQRGVGTGQIRAGIIDVKVDDVRNTGTIAGYAVGLEAKN
HMC56           NGQEVLVPQVYLAKQTIEEVEKQRGVGTGQIRAGIIDVKVDDVRNTGTIAGYAVGLEAKN
                ************************************************************

35000HP         KLKNTGDILSQRLSKLVGKKGLESTGVTYVDETGATKVRKARIKSEGHIYLETDKDKNVD
NZS1            KLKNTGDILSQRLSKLVGKKGLESTGVTYVDETGATKVRKARIKSEGHIYLETDKDKNVD
NZS2            KLKNTGDILSQRLSKLVGKKGLESTGVTYVDETGATKVRKARIKSEGHIYLETDKDKNVD
NZS3            KLKNTGDILSQRLSKLVGKKGLESTGVTYVDETGATKVRKARIKSEGHIYLETDKDKNVD
NZS4            KLKNTGDILSQRLSKLVGKKGLESTGVTYVDETGATKVRKARIKSEGHIYLETDKDKNVD
NZV1            KLKNTGDILSQRLSKLVGKKGLESTGVTYVDETGATKVRKARIKSEGHIYLETDKDKNVD
82-029362       KLKNTGDILSQRLSKLVGKKGLESTGVTYVDETGATKVRKARIKSEGHIYLETDKDKNVD
6644            KLKNTGDILSQRLSKLVGKKGLESTGVTYVDETGATKVRKARIKSEGHIYLETDKDKNVD
HD183           KLKNTGDILSQRLSKLVGKKGLESTGVTYVDETGATKVRKARIKSEGHIYLETDKDKNVD
HMC46           KLKNTGDILSQRLSKLVGKKGLESTGVTYVDETGATKVRKARIKSEGHIYLETDKDKNVD
HMC56           KLKNTGDILSQRLSKLVGKKGLESTGVTYVDETGATKVRKARIKSEGHIYLETDKDKNVD
                ************************************************************

35000HP         LTASELKGNTGQIKAKDLNLNDIYETSYKYKYEKLFGKNGGEIGDRVTQTSQAKSVGTDA
NZS1            LTASELKGNTGQIKAKDLNLNDIYETSYKYKYEKLFGKNGGEIGDRVTQTSQAKSVGTDA
NZS2            LTASELKGNTGQIKAKDLNLNDIYETSYKYKYEKLFGKNGGEIGDRVTQTSQAKSVGTDA
NZS3            LTASELKGNTGQIKAKDLNLNDIYETSYKYKYEKLFGKNGGEIGDRVTQTSQAKSVGTDA
NZS4            LTASELKGNTGQIKAKDLNLNDIYETSYKYKYEKLFGKNGGEIGDRVTQTSQAKSVGTDA
NZV1            LTASELKGNTGQIKAKDLNLNDIYETSYKYKYEKLFGKNGGEIGDRVTQTSQAKSVGTDA
82-029362       LTASELKGSTGQIKAKDLNLNDIYETSYKYKYEKLFGKNGGEIGDRVTQTSQAKSVGTDA
6644            LTASELKGSTGQIKAKDLNLNDIYETSYKYKYEKLFGKNGGEIGDRVTQTSQAKSVGTDA
HD183           LTASELKGNTGQIKAKDLNLNDIYETSYKYKYEKLFGKNGGEIGDRVTQTSQAKSVGTDA
HMC46           LTASELKGSTGQIKAKDLNLNDIYETSYKYKYEKLFGKNGGEIGDRVTQTSQAKSVGTDA
HMC56           LTASELKGSTGQIKAKDLNLNDIYETSYKYKYEKLFGKNGGEIGDRVTQTSQAKSVGTDA
                ********.***************************************************

35000HP         SFDHLHLSLEGDVNQTGSNLKANRTTGVVKGDFNTKAGKDLFHRQIDTVTSGTVYSASAS
NZS1            SFDHLHLSLEGDVNQTGSNLKANRTTGVVKGDFNTKAGKDLFHRQIDTVTSGTVYSASAS
NZS2            SFDHLHLSLEGDVNQTGSNLKANRTTGVVKGDFNTKAGKDLFHRQIDTVTSGTVYSASAS
NZS3            SFDHLHLSLEGDVNQTGSNLKANRTTGVVKGDFNTKAGKDLFHRQIDTVTSGTVYSASAS
NZS4            SFDHLHLSLEGDVNQTGSNLKANRTTGVVKGDFNTKAGKDLFHRQIDTVTSGTVYSASAS
NZV1            SFDHLHLSLEGDVNQTGSNLKANRTTGVVKGDFNTKAGKDLFHRQIDTVTSGTVYSASAS
82-029362       SFDHLHLSLEGDVNQTGSNLKANRTTGVVKGDFNTKAGKDLFHRQIDTVTSGTVYSASAS
6644            SFDHLHLSLEGDVNQTGSNLKANRTTGVVKGDFNTKAGKDLFHRQIDTVTSGTVYSASAS
```

```
HD183          SFDHLHLSLEGDVNQTGSNLKANRTTGVVKGDFNTKAGKDLFHRQIDTVTSGTVYSASAS
HMC46          SFDHLHLSLEGDVNQTGSNLKANRTTGVVKGDFNTKAGKDLFHRQIDTVTSGTVYSASAS
HMC56          SFDHLHLSLEGDVNQTGSNLKANRTTGVVKGDFNTKAGKDLFHRQIDTVTSGTVYSASAS
               ************************************************************

35000HP        GGGQSAGISLTDQGVETYTNKTATAGANADVTNFMKRTRETETSLTHRNSEFNALSGELY
NZS1           GGGQSAGISLTDQGVETYTNKTATAGANADVTNFMKRTRETETSLTHRNSEFNALSGELY
NZS2           GGGQSAGISLTDQGVETYTNKTATAGANADVTNFMKRTRETETSLTHRNSEFNALSGELY
NZS3           GGGQSAGISLTDQGVETYTNKTATAGANADVTNFMKRTRETETSLTHRNSEFNALSGELY
NZS4           GGGQSAGISLTDQGVETYTNKTATAGANADVTNFMKRTRETETSLTHRNSEFNALSGELY
NZV1           GGGQSAGISLTDQGVETYTNKTATAGANADVTNFMKRTRETETSLTHRNSEFNALSGELY
82-029362      GGGQSAGISLTDQGVETYTNKTATAGANADVTNFMKRTRETETSLTHRNSEFNALSGELY
6644           GGGQSAGISLTDQGVETYTNKTATAGANADVTNFMKRTRETETSLTHRNSEFNALSGELY
HD183          GGGQSAGISLTDQGVETYTNKTATAGANADVTNFMKRTRETETSLTHRNSEFNALSGELY
HMC46          GGGQSAGISLTDQGVETYTNKTATAGANADVTNFMKRTRETETSLTHRNSEFNALSGELY
HMC56          GGGQSAGISLTDQGVETYTNKTATAGANADVTNFMKRTRETETSLTHRNSEFNALSGELY
               ************************************************************

35000HP        VMGKADIGGVDINRDVEVIKTPEEIAAEQKAAEEAKKAEVKENEASETAAKETEEAENDN
NZS1           VMGKADIGGVDINRDVEVIKTPEEIAAEQKAAEEAKKAEVKENEASETAAKETEEAENDN
NZS2           VMGKADIGGVDINRDVEVIKTPEEIAAEQKAAEEAKKAEVKENEASETAAKETEEAENDN
NZS3           VMGKADIGGVDINRDVEVIKTPEEIAAEQKAAEEAKKAEVKENEASETAAKETEEAENDN
NZS4           VMGKADIGGVDINRDVEVIKTPEEIAAEQKAAEEAKKAEVKENEASETAAKETEEAENDN
NZV1           VMGKADIGGVDINRDVEVIKTPEEIAAEQKAAEEAKKAEVKENEASETAAKETEEAENDN
82-029362      VMGKADIGGVDINRDVEVIKTPEEIAAEQKAAEEAKKAEVKENEASETAAKETEEAENDN
6644           VMGKADIGGVDINRDVEVIKTPEEIAAEQKAAEEAKKAEVKENEASETAAKETEEAENDN
HD183          VMGKADIGGVDINRDVEVIKTPEEIAAEQKAAEEAKKAEVKENEASETAAKETEEAENDN
HMC46          VMGKADIGGVDINRDVEVIKTPEEIAAEQKAAEEAKKAEVKENEASETAAKETEEAENDN
HMC56          VMGKADIGGVDINRDVEVIKTPEEIAAEQKAAEEAKKAEVKENEASETAAKETEEAENDN
               ************************************************************

35000HP        VAEKDKTKPKFKKLTDEEIAAAFETKGEDFFAAYKAREEEDRKKGFTLSAEQIESTKARD
NZS1           VAEKDKTKPKFKKLTDEEIAAAFETKGEDFFAAYKAREEEDRKKGFTLSAEQIESTKARD
NZS2           VAEKDKTKPKFKKLTDEEIAAAFETKGEDFFAAYKAREEEDRKKGFTLSAEQIESTKARD
NZS3           VAEKDKTKPKFKKLTDEEIAAAFETKGEDFFAAYKAREEEDRKKGFTLSAEQIESTKARD
NZS4           VAEKDKTKPKFKKLTDEEIAAAFETKGEDFFAAYKAREEEDRKKGFTLSAEQIESTKARD
NZV1           VAEKDKTKPKFKKLTDEEIAAAFETKGEDFFAAYKAREEEDRKKGFTLSAEQIESTKARD
82-029362      VAEKDKTKPKFKKLTDEEIAAAFETKGEDFFAAYKAREEEDRKKGFTLSAEQIESTKARD
6644           VAEKDKTKPKFKKLTDEEIAAAFETKGEDFFAAYKAREEEDRKKGFTLSAEQIESTKARD
HD183          VAEKDKTKPKFKKLTDEEIAAAFETKGEDFFAAYKAREEEDRKKGFTLSAEQIESTKARD
HMC46          VAEKDKTKPKFKKLTDEEIAAAFETKGEDFFAAYKAREEEDRKKGFTLSAEQIESTKARD
HMC56          VAEKDKTKPKFKKLTDEEIAAAFETKGEDFFAAYKAREEEDRKKGFTLSAEQIESTKARD
               ************************************************************

35000HP        EKETTYYELKVGVGAEAEAHSAAADAISNKARQIIDTQNGLKQDGTVALQEASDVLNLAT
NZS1           EKETTYYELKVGVGAEAEAHSAAADAISNKARQIIDTQNGLKQDGTVALQEASDVLNLAT
NZS2           EKETTYYELKVGVGAEAEAHSAAADAISNKARQIIDTQNGLKQDGTVALQEASDVLNLAT
NZS3           EKETTYYELKVGVGAEAEAHSAAADAISNKARQIIDTQNGLKQDGTVALQEASDVLNLAT
NZS4           EKETTYYELKVGVGAEAEAHSAAADAISNKARQIIDTQNGLKQDGTVALQEASDVLNLAT
NZV1           EKETTYYELKVGVGAEAEAHSAAADAISNKARQIIDTQNGLKQDGTVALQEASDVLNLAT
82-029362      EKETTYYELKVGVGAEAEAHSAAADAISNKARQIIDTQNGLKQDGTVALQEASDVLNLAT
6644           EKETTYYELKVGVGAEAEAHSAAADAISNKARQIIDTQNGLKQDGTVALQEASDVLNLAT
HD183          EKETTYYELKVGVGAEAEAHSAAADAISNKARQIIDTQNGLKQDGTVALQEASDVLNLAT
HMC46          EKETTYYELKVGVGAEAEAHSAAADAISNKARQIIDTQNGLKQDGTVALQEASDVLNLAT
HMC56          EKETTYYELKVGVGAEAEAHSAAADAISNKARQIIDTQNGLKQDGTVALQEASDVLNLAT
               ************************************************************

35000HP        GDLAGASAKLKFELSTIEKKSRGASDGRSILGGRLNLAARGGDITLNNVETTENSHLSLK
NZS1           GDLAGASAKLKFELSTIEKKSRGASDGRSILGGRLNLAARGGDITLNNVETTENSHLSLK
NZS2           GDLAGASAKLKFELSTIEKKSRGASDGRSILGGRLNLAARGGDITLNNVETTENSHLSLK
NZS3           GDLAGASAKLKFELSTIEKKSRGASDGRSILGGRLNLAARGGDITLNNVETTENSHLSLK
NZS4           GDLAGASAKLKFELSTIEKKSRGASDGRSILGGRLNLAARGGDITLNNVETTENSHLSLK
NZV1           GDLAGASAKLKFELSTIEKKSRGASDGRSILGGRLNLAARGGDITLNNVETTENSHLSLK
82-029362      GDLAGASAKLKFELSTIEKKSRGASDGRSILGGRLNLAARGGDITLNNVETTENSHLSLK
6644           GDLAGASAKLKFELSTIEKKSRGASDGRSILGGRLNLAARGGDITLNNVETTENSHLSLK
HD183          GDLAGASAKLKFELSTIEKKSRGASDGRSILGGRLNLAARGGDITLNNVETTENSHLSLK
HMC46          GDLAGASAKLKFELSTIEKKSRGASDGRSILGGRLNLAARGGDITLNNVETTENSHLSLK
HMC56          GDLAGASAKLKFELSTIEKKSRGASDGRSILGGRLNLAARGGDITLNNVETTENSHLSLK
               ************************************************************

35000HP        ARDNVNVNSGVTEQKDESNSQSLKVTAGASSGCGVMAGGCSAGVSAGVSGSYNESNTEST
NZS1           ARDNVNVNSGVTEQKDESNSQSLKVTAGASSGCGVMAGGCSAGVSAGVSGSYNESNTEST
NZS2           ARDNVNVNSGVTEQKDESNSQSLKVTAGASSGCGVMAGGCSAGVSAGVSGSYNESNTEST
```

```
NZS3          ARDNVNVNSGVTEQKDESNSQSLKVTAGASSGCGVMAGGCSAGVSAGVSGSYNESNTEST
NZS4          ARDNVNVNSGVTEQKDESNSQSLKVTAGASSGCGVMAGGCSAGVSAGVSGSYNESNTEST
NZV1          ARDNVNVNSGVTEQKDESNSQSLKVTAGASSGCGVMAGGCSAGVSAGVSGSYNESNTEST
82-029362     ARDNVNVNSGVTEQKDESNSQSLKVTAGASSGCGVMAGGCSAGVSAGVSGSYNESNTEST
6644          ARDNVNVNSGVTEQKDESNSQSLKVTAGASSGCGVMAGGCSAGVSAGVSGSYNESNTEST
HD183         ARDNVNVNSGVTEQKDESNSQSLKVTAGASSGCGVMAGGCSAGVSAGVSGSYNESNTEST
HMC46         ARDNVNVNSGVTEQKDESNSQSLKVTAGASSGCGVMAGGCSAGVSAGVSGSYNESNTEST
HMC56         ARDNVNVNSGVTEQKDESNSQSLKVTAGASSGCGVMAGGCSAGVSAGVSGSYNESNTEST
              ************************************************************

35000HP       SHTNSLLRGKSLRVEAGKDFNLISSNVDVDHLHLDVKGDTNVVSKQDSYSRKERGVNYSV
NZS1          SHTNSLLRGKSLRVEAGKDFNLISSNVDVDHLHLDVKGDTNVVSKQDSYSRKERGVNYSV
NZS2          SHTNSLLRGKSLRVEAGKDFNLISSNVDVDHLHLDVKGDTNVVSKQDSYSRKERGVNYSV
NZS3          SHTNSLLRGKSLRVEAGKDFNLISSNVDVDHLHLDVKGDTNVVSKQDSYSRKERGVNYSV
NZS4          SHTNSLLRGKSLRVEAGKDFNLISSNVDVDHLHLDVKGDTNVVSKQDSYSRKERGVNYSV
NZV1          SHTNSLLRGKSLRVEAGKDFNLISSNVDVDHLHLDVKGDTNVVSKQDSYSRKERGVNYSV
82-029362     SHTNSLLRGKSLRVEAGKDFNLISSNVDVDHLHLDVKGDTNVVSKQDSYSRKERGVNYSV
6644          SHTNSLLRGKSLRVEAGKDFNLISSNVDVDHLHLDVKGDTNVVSKQDSYSRKERGVNYSV
HD183         SHTNSLLRGKSLRVEAGKDFNLISSNVDVDHLHLDVKGDTNVVSKQDSYSRKERGVNYSV
HMC46         SHTNSLLRGKSLRVEAGKDFNLISSNVDVDHLHLDVKGDTNVVSKQDSYSRKERGVNYSV
HMC56         SHTNSLLRGKSLRVEAGKDFNLISSNVDVDHLHLDVKGDTNVVSKQDSYSRKERGVNYSV
              ************************************************************

35000HP       SAGVGVSTAGGARPNGSVGLGVSAENENSKIVKQQAGISAKRITGEINNLNLTGGYIENK
NZS1          SAGVGVSTAGGARPNGSVGLGVSAENENSKIVKQQAGISAKRITGEINNLNLTGGYIENK
NZS2          SAGVGVSTAGGARPNGSVGLGVSAENENSKIVKQQAGISAKRITGEINNLNLTGGYIENK
NZS3          SAGVGVSTAGGARPNGSVGLGVSAENENSKIVKQQAGISAKRITGEINNLNLTGGYIENK
NZS4          SAGVGVSTAGGARPNGSVGLGVSAENENSKIVKQQAGISAKRITGEINNLNLTGGYIENK
NZV1          SAGVGVSTAGGARPNGSVGLGVSAENENSKIVKQQAGISAKRITGEINNLNLTGGYIENK
82-029362     SAGVGVSTAGGARPNGSVGLGVSAENENSKIVKQQAGISAKRITGEINNLNLTGGYIENK
6644          SAGVGVSTAGGARPNGSVGLGVSAENENSKIVKQQAGISAKRITGEINNLNLTGGYIENK
HD183         SAGVGVSTAGGARPNGSVGLGVSAENENSKIVKQQAGISAKRITGEINNLNLTGGYIENK
HMC46         SAGVGVSTAGGARPNGSVGLGVSAENENSKIVKQQAGISAKRITGEINNLNLTGGYIENK
HMC56         SAGVGVSTAGGARPNGSVGLGVSAENENSKIVKQQAGISAKRITGEINNLNLTGGYIENK
              ************************************************************

35000HP       GNPDELNVKGDITTHELKDEHHKDGGSFGGSVGVSETGVTQVNVNGGRVEQKHYEATQHS
NZS1          GNPDELNVKGDITTHELKDEHHKDGGSFGGSVGVSETGVTQVNVNGGRVEQKHYEATQHS
NZS2          GNPDELNVKGDITTHELKDEHHKDGGSFGGSVGVSETGVTQVNVNGGRVEQKHYEATQHS
NZS3          GNPDELNVKGDITTHELKDEHHKDGGSFGGSVGVSETGVTQVNVNGGRVEQKHYEATQHS
NZS4          GNPDELNVKGDITTHELKDEHHKDGGSFGGSVGVSETGVTQVNVNGGRVEQKHYEATQHS
NZV1          GNPDELNVKGDITTHELKDEHHKDGGSFGGSVGVSETGVTQVNVNGGRVEQKHYEATQHS
82-029362     GNPDELNVKGDITTHELKDEHHKDGGSFGGSVGVSETGVTQVNVNGGRVEQKHYEATQHS
6644          GNPDELNVKGDITTHELKDEHHKDGGSFGGSVGVSETGVTQVNVNGGRVEQKHYEATQHS
HD183         GNPDELNVKGDITTHELKDEHHKDGGSFGGSVGVSETGVTQVNVNGGRVEQKHYEATQHS
HMC46         GNPDELNVKGDITTHELKDEHHKDGGSFGGSVGVSETGVTQVNVNGGRVEQKHYEATQHS
HMC56         GNPDELNVKGDITTHELKDEHHKDGGSFGGSVGVSETGVTQVNVNGGRVEQKHYEATQHS
              ************************************************************

35000HP       SISGINTKGKTVGNFKTDRSQSTEVHRDDTIAATNFNFELGDIAELAKKGKEKWDNRSAK
NZS1          SISGINTKGKTVGNFKTDRSQSTEVHRDDTIAATNFNFELGDIAELAKKGKEKWDNRSAK
NZS2          SISGINTKGKTVGNFKTDRSQSTEVHRDDTIAATNFNFELGDIAELAKKGKEKWDNRSAK
NZS3          SISGINTKGKTVGNFKTDRSQSTEVHRDDTIAATNFNFELGDIAELAKKGKEKWDNRSAK
NZS4          SISGINTKGKTVGNFKTDRSQSTEVHRDDTIAATNFNFELGDIAELAKKGKEKWDNRSAK
NZV1          SISGINTKGKTVGNFKTDRSQSTEVHRDDTIAATNFNFELGDIAELAKKGKEKWDNRSAK
82-029362     SISGINTKGKTVGNFKTDRSQSTEVHRDDTIAATNFNFELGDIAELAKKGKEKWDNRSAK
6644          SISGINTKGKTVGNFKTDRSQSTEVHRDDTIAATNFNFELGDIAELAKKGKEKWDNRSAK
HD183         SISGINTKGKTVGNFKTDRSQSTEVHRDDTIAATNFNFELGDIAELAKKGKEKWDNRSAK
HMC46         SISGINTKGKTVGNFKTDRSQSTEVHRDDTIAATNFNFELGDIAELAKKGKEKWDNRSAK
HMC56         SISGINTKGKTVGNFKTDRSQSTEVHRDDTIAATNFNFELGDIAELAKKGKEKWDNRSAK
              ************************************************************

35000HP       TTSSSQDSAHDPRSRSVENGYSELPRFKTADNDAGVDSPRLIKGEAEQAQTLALTKAGND
NZS1          TTSSSQDSAHDPRSRSVENGYSELPRFKTADNDAGVDSPRLIKGEAEQAQTLALTKAGND
NZS2          TTSSSQDSAHDPRSRSVENGYSELPRFKTADNDAGVDSPRLIKGEAEQAQTLALTKAGND
NZS3          TTSSSQDSAHDPRSRSVENGYSELPRFKTADNDAGVDSPRLIKGEAEQAQTLALTKAGND
NZS4          TTSSSQDSAHDPRSRSVENGYSELPRFKTADNDAGVDSPRLIKGEAEQAQTLALTKAGND
NZV1          TTSSSQDSAHDPRSRSVENGYSELPRFKTADNDAGVDSPRLIKGEAEQAQTLALTKAGND
82-029362     TTSSSQDSAHDPRSRSVENGYSELPRFKTADNDAGVDSPRLIKGEAEQAQTLALTKAGND
6644          TTSSSQDSAHDPRSRSVENGYSELPRFKTADNDAGVDSPRLIKGEAEQAQTLALTKAGND
HD183         TTSSSQDSAHDPRSRSVENGYSELPRFKTADNDAGVDSPRLIKGEAEQAQTLALTKAGND
HMC46         TTSSSQDSAHDPRSRSVENGYSELPRFKTADNDAGVDSPRLIKGEAEQAQTLALTKAGND
HMC56         TTSSSQDSAHDPRSRSVENGYSELPRFKTADNDAGVDSPRLIKGEAEQAQTLALTKAGND
```

```
               ************************************************************

35000HP        VIPEVQSLTQKARPQSLVDESPYAEIPALTRPQVKSNIAESIEVPQFRTKVSDGDEGNYA
NZS1           VIPEVQSLTQKARPQSLVDESPYAEIPALTRPQVKSNIAESIEVPQFRTKVSDGDEGNYA
NZS2           VIPEVQSLTQKARPQSLVDESPYAEIPALTRPQVKSNIAESIEVPQFRTKVSDGDEGNYA
NZS3           VIPEVQSLTQKARPQSLVDESPYAEIPALTRPQVKSNIAESIEVPQFRTKVSDGDEGNYA
NZS4           VIPEVQSLTQKARPQSLVDESPYAEIPALTRPQVKSNIAESIEVPQFRTKVSDGDEGNYA
NZV1           VIPEVQSLTQKARPQSLVDESPYAEIPALTRPQVKSNIAESIEVPQFRTKVSDGDEGNYA
82-029362      VIPEVQSLTQKARPQSLVDESPYAEIPALTRPQVKSNIAESIEVPQFRTKVSDGDEGNYA
6644           VIPEVQSLTQKARPQSLVDESPYAEIPALTRPQVKSNIAESIEVPQFRTKVSDGDEGNYA
HD183          VIPEVQSLTQKARPQSLVDESPYAEIPALTRPQVKSNIAESIEVPQFRTKVSDGDEGNYA
HMC46          VIPEVQSLTQKARPQSLVDESPYAEIPALTRPQVKSNIAESIEVPQFRTKVSDGDEGNYA
HMC56          VIPEVQSLTQKARPQSLVDESPYAEIPALTRPQVKSNIAESIEVPQFRTKVSDGDEGNYA
               ************************************************************

35000HP        EITFPTNKAAISSTQDVGDTPTPRALRLESESGYESAENLGLIPRGFKSSPKGEYEDISD
NZS1           EITFPTNKAAISSTQDVGDTPTPRALRLESESGYESAENLGLIPRGFKSSPKGEYEDISD
NZS2           EITFPTNKAAISSTQDVGDTPTPRALRLESESGYESAENLGLIPRGFKSSPKGEYEDISD
NZS3           EITFPTNKAAISSTQDVGDTPTPRALRLESESGYESAENLGLIPRGFKSSPKGEYEDISD
NZS4           EITFPTNKAAISSTQDVGDTPTPRALRLESESGYESAENLGLIPRGFKSSPKGEYEDISD
NZV1           EITFPTNKAAISSTQDVGDTPTPRALRLESESGYESAENLGLIPRGFKSSPKGEYEDISD
82-029362      EITFPTNKAAISSTQDVGDTPTPRALRLESESGYESAENLGLIPRGFKSSPKGEYEDISD
6644           EITFPTNKAAISSTQDVGDTPTPRALRLESESGYESAENLGLIPRGFKSSPKGEYEDISD
HD183          EITFPTNKAAISSTQDVGDTPTPRALRLESESGYESAENLGLIPRGFKSSPKGEYEDISD
HMC46          EITFPTNKAAISSTQDVGDTPTPRALRLESESGYESAENLGLIPRGFKSSPKGEYEDISD
HMC56          EITFPTNKAAISSTQDVGDTPTPRALRLESESGYESAENLGLIPRGFKSSPKGEYEDISD
               ************************************************************

35000HP        AIEPQTRSRKLDEPEPIYGTINKSPEAIARANAKADEAIQALGYDPRIKPVVPEEAPPAL
NZS1           AIEPQTRSRKLDEPEPIYGTINKSPEAIARANAKADEAIQALGYDPRIKPVVPEEAPPAL
NZS2           AIEPQTRSRKLDEPEPIYGTINKSPEAIARANAKADEAIQALGYDPRIKPVVPEEAPPAL
NZS3           AIEPQTRSRKLDEPEPIYGTINKSPEAIARANAKADEAIQALGYDPRIKPVVPEEAPPAL
NZS4           AIEPQTRSRKLDEPEPIYGTINKSPEAIARANAKADEAIQALGYDPRIKPVVPEEAPPAL
NZV1           AIEPQTRSRKLDEPEPIYGTINKSPEAIARANAKADEAIQALGYDPRIKPVVPEEAPPAL
82-029362      AIEPQTRSRKLDEPEPIYGTINKSPEAIARANAKADEAIQALGYDPRIKPVVPEEAPPAL
6644           AIEPQTRSRKLDEPEPIYGTINKSPEAIARANAKADEAIQALGYDPRIKPVVPEEAPPAL
HD183          AIEPQTRSRKLDEPEPIYGTINKSPEAIARANAKADEAIQALGYDPRIKPVVPEEAPPAL
HMC46          AIEPQTRSRKLDEPEPIYGTINKSPEAIARANAKADEAIQALGYDPRIKPVVPEEAPPAL
HMC56          AIEPQTRSRKLDEPEPIYGTINKSPEAIARANAKADEAIQALGYDPRIKPVVPEEAPPAL
               ************************************************************

35000HP        PPRNLQTKAISDYDDVSYVPDFKVRKTDEPEPIYGTINKSPEAIARANAKADEAIQASGY
NZS1           PPRNLQTKAISDYDDVSYVPDFKVRKTDEPEPIYGTINKSPEAIARANAKADEAIQASGY
NZS2           PPRNLQTKAISDYDDVSYVPDFKVRKTDEPEPIYGTINKSPEAIARANAKADEAIQASGY
NZS3           PPRNLQTKAISDYDDVSYVPDFKVRKTDEPEPIYGTINKSPEAIARANAKADEAIQASGY
NZS4           PPRNLQTKAISDYDDVSYVPDFKVRKTDEPEPIYGTINKSPEAIARANAKADEAIQASGY
NZV1           PPRNLQTKAISDYDDVSYVPDFKVRKTDEPEPIYGTINKSPEAIARANAKADEAIQASGY
82-029362      PPRNLQTKAISDYDDVSYVPDFKVRKTDEPEPIYGTINKSPEAIARANAKADEAIQASGY
6644           PPRNLQTKAISDYDDVSYVPDFKVRKTDEPEPIYGTINKSPEAIARANAKADEAIQASGY
HD183          PPRNLQTKAISDYDDVSYVPDFKVRKTDEPEPIYGTINKSPEAIARANAKADEAIQASGY
HMC46          PPRNLQTKAISDYDDVSYVPDFKVRKTDEPEPIYGTINKSPEAIARANAKADEAIQASGY
HMC56          PPRNLQTKAISDYDDVSYVPDFKVRKTDEPEPIYGTINKSPEAIARANAKADEAIQASGY
               ************************************************************

35000HP        DPRIKPVVPEDAPPALPPRTQSLIDSTEVPSYRSALANVKFDDASPWPQPSALRSKAFAD
NZS1           DPRIKPVVPEDAPPALPPRTQSLIDSTEVPSYRSALANVKFDDASPWPQPSALRSKAFAD
NZS2           DPRIKPVVPEDAPPALPPRTQSLIDSTEVPSYRSALANVKFDDASPWPQPSALRSKAFAD
NZS3           DPRIKPVVPEDAPPALPPRTQSLIDSTEVPSYRSALANVKFDDASPWPQPSALRSKAFAD
NZS4           DPRIKPVVPEDAPPALPPRTQSLIDSTEVPSYRSALANVKFDDASPWPQPSALRSKAFAD
NZV1           DPRIKPVVPEDAPPALPPRTQSLIDSTEVPSYRSALANVKFDDASPWPQPSALRSKAFAD
82-029362      DPRIKPVVPEDAPPALPPRTQSLIDSTEVPSYRSALANVKFDDASPWPQPSALRSKAFAD
6644           DPRIKPVVPEDAPPALPPRTQSLIDSTEVPSYRSALANVKFDDASPWPQPSALRSKAFAD
HD183          DPRIKPVVPEDAPPALPPRTQSLIDSTEVPSYRSALANVKFDDASPWPQPSALRSKAFAD
HMC46          DPRIKPVVPEDAPPALPPRTQSLIDSTEVPSYRSALANVKFDDASPWPQPSALRSKAFAD
HMC56          DPRIKPVVPEDAPPALPPRTQSLIDSTEVPSYRSALANVKFDDASPWPQPSALRSKAFAD
               ************************************************************

35000HP        EPSSETPKSRGKRGISEESLSSTVQPRSRKISEEDSSFERLPLRIIDNGSDYAEILPRNV
NZS1           EPSSETPKSRGKRGISEESLSSTVQPRSRKISEEDSSFERLPLRIIDNGSDYAEILPRNV
NZS2           EPSSETPKSRGKRGISEESLSSTVQPRSRKISEEDSSFERLPLRIIDNGSDYAEILPRNV
NZS3           EPSSETPKSRGKRGISEESLSSTVQPRSRKISEEDSSFERLPLRIIDNGSDYAEILPRNV
NZS4           EPSSETPKSRGKRGISEESLSSTVQPRSRKISEEDSSFERLPLRIIDNGSDYAEILPRNV
NZV1           EPSSETPKSRGKRGISEESLSSTVQPRSRKISEEDSSFERLPLRIIDNGSDYAEILPRNV
```

```
82-029362      EPSSETPKSRGKRGISEESLSSTVQPRSRKISEEDSSFERLPLRIIDNGSDYAEILPRNV
6644           EPSSETPKSRGKRGISEESLSSTVQPRSRKISEEDSSFERLPLRIIDNGSDYAEILPRNV
HD183          EPSSETPKSRGKRGISEESLSSTVQPRSRKISEEDSSFERLPLRIIDNGSDYAEILPRNV
HMC46          EPSSETPKSRGKRGISEESLSSTVQPRSRKISEEDSSFERLPLRIIDNGSDYAEILPRNV
HMC56          EPSSETPKSRGKRGISEESLSSTVQPRSRKISEEDSSFERLPLRIIDNGSDYAEILPRNV
               ************************************************************

35000HP        KQTNEPATQAIRAPKALDNNDVIAERPSFKLRQLDDDVESVNGIYSSIKPKALIEEGTPI
NZS1           KQTNEPATQAIRAPKALDNNDVIAERPSFKLRQLDDDVESVNGIYSSIKPKALIEEGTPI
NZS2           KQTNEPATQAIRAPKALDNNDVIAERPSFKLRQLDDDVESVNGIYSSIKPKALIEEGTPI
NZS3           KQTNEPATQAIRAPKALDNNDVIAERPSFKLRQLDDDVESVNGIYSSIKPKALIEEGTPI
NZS4           KQTNEPATQAIRAPKALDNNDVIAERPSFKLRQLDDDVESVNGIYSSIKPKALIEEGTPI
NZV1           KQTNEPATQAIRAPKALDNNDVIAERPSFKLRQLDDDVESVNGIYSSIKPKALIEEGTPI
82-029362      KQTNEPATQAIRAPKALDNNDVIAERPSFKLRQLDDDVESVNGIYSSIKPKALIEEGTPI
6644           KQTNEPATQAIRAPKALDNNDVIAERPSFKLRQLDDDVESVNGIYSSIKPKALIEEGTPI
HD183          KQTNEPATQAIRAPKALDNNDVIAERPSFKLRQLDDDVESVNGIYSSIKPKALIEEGTPI
HMC46          KQTNEPATQAIRAPKALDNNDVIAERPSFKLRQLDDDVESVNGIYSSIKPKALIEEGTPI
HMC56          KQTNEPATQAIRAPKALDNNDVIAERPSFKLRQLDDDVESVNGIYSSIKPKALIEEGTPI
               ************************************************************

35000HP        TRQVKTVQEETPVTDLVNKRELVKEDRSLLDKVQDTFQPLKVRSKINDVRSSVEEYGGEV
NZS1           TRQVKTVQEETPVTNLVNKRELVKEDRSLLDKVQDTFQPLKVRSKINDVRSSVEEYGGEV
NZS2           TRQVKTVQEETPVTNLVNKRELVKEDRSLLDKVQDTFQPLKVRSKINDVRSSVEEYGGEV
NZS3           TRQVKTVQEETPVTNLVNKRELVKEDRSLLDKVQDTFQPLKVRSKINDVRSSVEEYGGEV
NZS4           TRQVKTVQEETPVTNLVNKRELVKEDRSLLDKVQDTFQPLKVRSKINDVRSSVEEYGGEV
NZV1           TRQVKTVQEETPVTNLVNKRELVKEDRSLLDKVQDTFQPLKVRSKINDVRSSVEEYGGEV
82-029362      TRQVKTVQEETPVTDLVNKRELVKEDRSLLDKVQDTFQPLKVRSKINDVRSSVEEYGGEV
6644           TRQVKTVQEETPVTDLVNKRELVKEDRSLLDKVQDTFQPLKVRSKINDVRSSVEEYGGEV
HD183          TRQVKTVQEETPVTDLVNKRELVKEDRSLLDKVQDTFQPLKVRSKINDVRSSVEEYGGEV
HMC46          TRQVKTVQEETPVTDLVNKRELVKEDRSLLDKVQDTFQPLKVRSKINDVRSSVEEYGGEV
HMC56          TRQVKTVQEETPVTDLVNKRELVKEDRSLLDKVQDTFQPLKVRSKINDVRSSVEEYGGEV
               **************:*********************************************

35000HP        TFKYAQSKGEVYNEIVKHAETQNGVCEATCSHWIAKKVNDENIWTDLYKDGQKGRKGGLN
NZS1           TFKYAQSKGEVYNEIVKHAETQNGVCEATCSHWIAKKVNDENIWTDLYKDGQKGRKGGLN
NZS2           TFKYAQSKGEVYNEIVKHAETQNGVCEATCSHWIAKKVNDENIWTDLYKDGQKGRKGGLN
NZS3           TFKYAQSKGEVYNEIVKHAETQNGVCEATCSHWIAKKVNDENIWTDLYKDGQKGRKGGLN
NZS4           TFKYAQSKGEVYNEIVKHAETQNGVCEATCSHWIAKKVNDENIWTDLYKDGQKGRKGGLN
NZV1           TFKYAQSKGEVYNEIVKHAETQNGVCEATCSHWIAKKVNDENIWTDLYKDGQKGRKGGLN
82-029362      TFKYAQSKGEVYNEIVKHAETQNGVCEATCSHWIAKKVNDENIWTDLYKDGQKGRKGGLN
6644           TFKYAQSKGEVYNEIVKHAETQNGVCEATCSHWIAKKVNDENIWTDLYKDGQKGRKGGLN
HD183          TFKYAQSKGEVYNEIVKHAETQNGVCEATCSHWIAKKVNDENIWTDLYKDGQKGRKGGLN
HMC46          TFKYAQSKGEVYNEIVKHAETQNGVCEATCSHWIAKKVNDENIWTDLYKDGQKGRKGGLN
HMC56          TFKYAQSKGEVYNEIVKHAETQNGVCEATCSHWIAKKVNDENIWTDLYKDGQKGRKGGLN
               ************************************************************

35000HP        KDAIESIEKLQTEFINAGTATQQFKLTNTWLEEQGVVPKQKYFGKLSRADEVAGTVSKND
NZS1           KDAIESIEKLQTEFINAGTATQQFKLTNTWLEEQGVVPKQKYFGKLSRADEVAGTVSKND
NZS2           KDAIESIEKLQTEFINAGTATQQFKLTNTWLEEQGVVPKQKYFGKLSRADEVAGTVSKND
NZS3           KDAIESIEKLQTEFINAGTATQQFKLTNTWLEEQGVVPKQKYFGKLSRADEVAGTVSKND
NZS4           KDAIESIEKLQTEFINAGTATQQFKLTNTWLEEQGVVPKQKYFGKLSRADEVAGTVSKND
NZV1           KDAIESIEKLQTEFINAGTATQQFKLTNTWLEEQGVVPKQKYFGKLSRADEVAGTVSKND
82-029362      KDAIESIEKLQTEFINAGTATQQFKLTNTWLEEQGVVPKQKYFGKLSRADEVAGTVSKND
6644           KDAIESIEKLQTEFINAGTATQQFKLTNTWLEEQGVVPKQKYFGKLSRADEVAGTVSKND
HD183          KDAIESIEKLQTEFINAGTATQQFKLTNTWLEEQGVVPKQKYFGKLSRADEVAGTVSKND
HMC46          KDAIESIEKLQTEFINAGTATQQFKLTNTWLEEQGVVPKQKYFGKLSRADEVAGTVSKND
HMC56          KDAIESIEKLQTEFINAGTATQQFKLTNTWLEEQGVVPKQKYFGKLSRADEVAGTVSKND
               ************************************************************

35000HP        VSALVKAILDTGNESSAVKKISINLEGGSHTVSASIEGQKVVFFDPNFGEITFKDKKSFE
NZS1           VSALVKAILDTGNESSAVKKISINLEGGSHTVSASIEGQKVVFFDPNFGEITFKDKKSFE
NZS2           VSALVKAILDTGNESSAVKKISINLEGGSHTVSASIEGQKVVFFDPNFGEITFKDKKSFE
NZS3           VSALVKAILDTGNESSAVKKISINLEGGSHTVSASIEGQKVVFFDPNFGEITFKDKKSFE
NZS4           VSALVKAILDTGNESSAVKKISINLEGGSHTVSASIEGQKVVFFDPNFGEITFKDKKSFE
NZV1           VSALVKAILDTGNESSAVKKISINLEGGSHTVSASIEGQKVVFFDPNFGEITFKDKKSFE
82-029362      VSALVKAILDTGNESSAVKKISINLEGGSHTVSASIEGQKVVFFDPNFGEITFKDKKSFE
6644           VSALVKAILDTGNESSAVKKISINLEGGSHTVSASIEGQKVVFFDPNFGEITFKDKKSFE
HD183          VSALVKAILDTGNESSAVKKISINLEGGSHTVSASIEGQKVVFFDPNFGEITFKDKKSFE
HMC46          VSALVKAILDTGNESSAVKKISINLEGGSHTVSASIEGQKVVFFDPNFGEITFKDKKSFE
HMC56          VSALVKAILDTGNESSAVKKISINLEGGSHTVSASIEGQKVVFFDPNFGEITFKDKKSFE
               ************************************************************

35000HP        KWMKNAFWKKSGYAGKKDTKRFFNVVNYHKNSKRNKVIDVNQNHIQQLAGSEGFSPSLPT
```

```
NZS1         KWMKNAFWKKSGYAGKKDTKRFFNVVNYHKNSKRNKVIDVNQNHIQQLAGSEGFSPSLPT
NZS2         KWMKNAFWKKSGYAGKKDTKRFFNVVNYHKNSKRNKVIDVNQNHIQQLAGSEGFSPSLPT
NZS3         KWMKNAFWKKSGYAGKKDTKRFFNVVNYHKNSKRNKVIDVNQNHIQQLAGSEGFSPSLPT
NZS4         KWMKNAFWKKSGYAGKKDTKRFFNVVNYHKNSKRNKVIDVNQNHIQQLAGSEGFSPSLPT
NZV1         KWMKNAFWKKSGYAGKKDTKRFFNVVNYHKNSKRNKVIDVNQNHIQQLAGSEGFSPSLPT
82-029362    KWMKNAFWKKSGYAGKKDTKRFFNVVNYHKNSKRNKVIDVNQNHIQQLAGSEGFSPSLPT
6644         KWMKNAFWKKSGYAGKKDTKRFFNVVNYHKNSKRNKVIDVNQNHIQQLAGSEGFSPSLPT
HD183        KWMKNAFWKKSGYAGKKDTKRFFNVVNYHKNSKRNKVIDVNQNHIQQLAGSEGFSPSLPT
HMC46        KWMKNAFWKKSGYAGKKDTKRFFNVVNYHKNSKRNKVIDVNQNHIQQLAGSEGFSPSLPT
HMC56        KWMKNAFWKKSGYAGKKDTKRFFNVVNYHKNSKRNKVIDVNQNHIQQLAGSEGFSPSLPT
             ************************************************************

35000HP      RPQLANAAGIKSNEMSSLFSWSKLKHLFSRESGKKAQVEGPEIKHLGGVVDKDAFYFPLD
NZS1         RPQLANAAGIKSNEMSSLFSWSKLKHLFSRESGKKAQVEGPEIKHLGGVVDKDAFYFPLD
NZS2         RPQLANAAGIKSNEMSSLFSWSKLKHLFSRESGKKAQVEGPEIKHLGGVVDKDAFYFPLD
NZS3         RPQLANAAGIKSNEMSSLFSWSKLKHLFSRESGKKAQVEGPEIKHLGGVVDKDAFYFPLD
NZS4         RPQLANAAGIKSNEMSSLFSWSKLKHLFSRESGKKAQVEGPEIKHLGGVVDKDAFYFPLD
NZV1         RPQLANAAGIKSNEMSSLFSWSKLKHLFSRESGKKAQVEGPEIKHLGGVVDKDAFYFPLD
82-029362    RPQLANAAGIKSNEMSSLFSWSKLKHLFSRESGKKAQVEGPEIKHLGGVVDKDAFYFPLD
6644         RPQLANAAGIKSNEMSSLFSWSKLKHLFSRESGKKAQVEGPEIKHLGGVVDKDAFYFPLD
HD183        RPQLANAAGIKSNEMSSLFSWSKLKHLFSRESGKKAQVEGPEIKHLGGVVDKDAFYFPLD
HMC46        RPQLANAAGIKSNEMSSLFSWSKLKHLFSRESGKKAQVEGPEIKHLGGVVDKDAFYFPLD
HMC56        RPQLANAAGIKSNEMSSLFSWSKLKHLFSRESGKKAQVEGPEIKHLGGVVDKDAFYFPLD
             ************************************************************

35000HP      KIVTRRDAEGEIRVNMDNIKKAFNPRDKHYNSQEARSLRSLYNQDPSMSGTRFIIENQVI
NZS1         KIVTRRDAEGEIRVNMDNIKKAFNPRDKHYNSQEARSLRSLYNQDPSMSGTRFIIENQVI
NZS2         KIVTRRDAEGEIRVNMDNIKKAFNPRDKHYNSQEARSLRSLYNQDPSMSGTRFIIENQVI
NZS3         KIVTRRDAEGEIRVNMDNIKKAFNPRDKHYNSQEARSLRSLYNQDPSMSGTRFIIENQVI
NZS4         KIVTRRDAEGEIRVNMDNIKKAFNPRDKHYNSQEARSLRSLYNQDPSMSGTRFIIENQVI
NZV1         KIVTRRDAEGEIRVNMDNIKKAFNPRDKHYNSQEARSLRSLYNQDPSMSGTRFIIENQVI
82-029362    KIVTRRDAEGEIRVNMDNIKKAFNPRDKHYNSQEARSLRSLYNQDPSMSGTRFIIENQVI
6644         KIVTRRDAEGEIRVNMDNIKKAFNPRDKHYNSQEARSLRSLYNQDPSMSGTRFIIENQVI
HD183        KIVTRRDAEGEIRVNMDNIKKAFNPRDKHYNSQEARSLRSLYNQDPSMSGTRFIIENQVI
HMC46        KIVTRRDAEGEIRVNMDNIKKAFNPRDKHYNSQEARSLRSLYNQDPSMSGTRFIIENQVI
HMC56        KIVTRRDAEGEIRVNMDNIKKAFNPRDKHYNSQEARSLRSLYNQDPSMSGTRFIIENQVI
             ************************************************************

35000HP      ANPFSSADLQSYIQAQQSKLPELGRQARRALPELPTAANKGRGSRVEEQNIVTRPRVEDV
NZS1         ANPFSSADLQSYIQAQQSKLPELGRQARRALPELPTAANKGRGSRVEEQNIVTRPRVEDV
NZS2         ANPFSSADLQSYIQAQQSKLPELGRQARRALPELPTAANKGRGSRVEEQNIVTRPRVEDV
NZS3         ANPFSSADLQSYIQAQQSKLPELGRQARRALPELPTAANKGRGSRVEEQNIVTRPRVEDV
NZS4         ANPFSSADLQSYIQAQQSKLPELGRQARRALPELPTAANKGRGSRVEEQNIVTRPRVEDV
NZV1         ANPFSSADLQSYIQAQQSKLPELGRQARRALPELPTAANKGRGSRVEEQNIVTRPRVEDV
82-029362    ANPFSSADLQSYIQAQQSKLPELGRQARRALPELPTAANKGRGSRVEEQNIVTRPRVEDV
6644         ANPFSSADLQSYIQAQQSKLPELGRQARRALPELPTAANKGRGSRVEEQNIVTRPRVEDV
HD183        ANPFSSADLQSYIQAQQSKLPELGRQARRALPELPTAANKGRGSRVEEQNIVTRPRVEDV
HMC46        ANPFSSADLQSYIQAQQSKLPELGRQARRALPELPTAANKGRGSRVEEQNIVTRPRVEDV
HMC56        ANPFSSADLQSYIQAQQSKLPELGRQARRALPELPTAANKGRGSRVEEQNIVTRPRVEDV
             ************************************************************

35000HP      YATVNKGAKHGEAQQPGSFYTKKLVDQVSHVPNTEPVYADLHFNRNGRVVRQTEPEVIYE
NZS1         YATVNKGAKHGEAQQPGSFYTKKLVDQVSHVPNTEPVYADLHFNRNGRVVRQTEPEVIYE
NZS2         YATVNKGAKHGEAQQPGSFYTKKLVDQVSHVPNTEPVYADLHFNRNGRVVRQTEPEVIYE
NZS3         YATVNKGAKHGEAQQPGSFYTKKLVDQVSHVPNTEPVYADLHFNRNGRVVRQTEPEVIYE
NZS4         YATVNKGAKHGEAQQPGSFYTKKLVDQVSHVPNTEPVYADLHFNRNGRVVRQTEPEVIYE
NZV1         YATVNKGAKHGEAQQPGSFYTKKLVDQVSHVPNTEPVYADLHFNRNGRVVRQTEPEVIYE
82-029362    YATVNKGAKHGEAQQPGSFYTKKLVDQVSHVPTTEPVYADLHFNRNGRVVRQTEPEVIYE
6644         YATVNKGAKHGEAQQPGSFYTKKLVDQVSHVPTTEPVYADLHFNRNGRVVRQTEPEVIYE
HD183        YATVNKGAKHGEAQQPGSFYTKKLVDQVSHVPNTEPVYADLHFNRNGRVVRQTEPEVIYE
HMC46        YATVNKGAKHGEAQQPGSFYTKKLVDQVSHVPTTEPVYADLHFNRNGRVVRQTEPEVIYE
HMC56        YATVNKGAKHGEAQQPGSFYTKKLVDQVSHVPTTEPVYADLHFNRNGRVVRQTEPEVIYE
             ********************************.***************************

35000HP      KIRGQQVEVDDPSSLYAKVNRNRRLDNVEGFYPPEQLRTRSDKLAEQVSRVPTTEPVYAD
NZS1         KIRGQQVEVDDPSSLYAKVNRNRRLDNVEGFYPPEQLRTRSDKLAEQVSRVPTTEPVYAD
NZS2         KIRGQQVEVDDPSSLYAKVNRNRRLDNVEGFYPPEQLRTRSDKLAEQVSRVPTTEPVYAD
NZS3         KIRGQQVEVDDPSSLYAKVNRNRRLDNVEGFYPPEQLRTRSDKLAEQVSRVPTTEPVYAD
NZS4         KIRGQQVEVDDPSSLYAKVNRNRRLDNVEGFYPPEQLRTRSDKLAEQVSRVPTTEPVYAD
NZV1         KIRGQQVEVDDPSSLYAKVNRNRRLDNVEGFYPPEQLRTRSDKLAEQVSRVPTTEPVYAD
82-029362    KIRGQQVEVDDPSSLYAKVNRNRRLDNVEGFYPPEQLRTRSDKLAEQVSRVPTTEPVYAD
6644         KIRGQQVEVDDPSSLYAKVNRNRRLDNVEGFYPPEQLRTRSDKLAEQVSRVPTTEPVYAD
HD183        KIRGQQVEVDDPSSLYAKVNRNRRLDNVEGFYPPEQLRTRSDKLAEQVSRVPTTEPVYAD
```

```
HMC46        KIRGQQVEVDDPSSLYAKVNRNRRLDNVEGFYPPEQLRTRSDKLAEQVSRVPTTEPVYAD
HMC56        KIRGQQVEVDDPSSLYAKVNRNRRLDNVEGFYPPEQLRTRSDKLAEQVSRVPTTEPVYAD
             ************************************************************

35000HP      LRFKSAEDDYAPALPARPELGNAAGFRKAKVKGEESESTWSRLKHLFSRESGKKAQVEGP
NZS1         LRFKSAEDDYAPALPARPELGNAAGFRKAKVKGEESESTWSRLKHLFSRESGKKAQVEGP
NZS2         LRFKSAEDDYAPALPARPELGNAAGFRKAKVKGEESESTWSRLKHLFSRESGKKAQVEGP
NZS3         LRFKSAEDDYAPALPARPELGNAAGFRKAKVKGEESESTWSRLKHLFSRESGKKAQVEGP
NZS4         LRFKSAEDDYAPALPARPELGNAAGFRKAKVKGEESESTWSRLKHLFSRESGKKAQVEGP
NZV1         LRFKSAEDDYAPALPARPELGNAAGFRKAKVKGEESESTWSRLKHLFSRESGKKAQVEGP
82-029362    LRFKSAEDDYAPALPARPELGNAAGFRKAKVKGEESESTWSRLKHLFSRESGKKAQVEGP
6644         LRFKSAEDDYAPALPARPELGNAAGFRKAKVKGEESESTWSRLKHLFSRESGKKAQVEGP
HD183        LRFKSAEDDYAPALPARPELGNAAGFRKAKVKGEESESTWSRLKHLFSRESGKKAQVEGP
HMC46        LRFKSAEDDYAPALPARPELGNAAGFRKAKVKGEESESTWSRLKHLFSRESGKKAQVEGP
HMC56        LRFKSAEDDYAPALPARPELGNAAGFRKAKVKGEESESTWSRLKHLFSRESGKKAQVEGP
             ************************************************************

35000HP      EIKHLGGVVDKDAFYFPLDKIVTRRDAEGEIRVNMDNIKKAFNPRDKHYNSQEARSLRSL
NZS1         EIKHLGGVVDKDAFYFPLDKIVTRRDAEGEIRVNMDNIKKAFNPRDKHYNSQEARSLRSL
NZS2         EIKHLGGVVDKDAFYFPLDKIVTRRDAEGEIRVNMDNIKKAFNPRDKHYNSQEARSLRSL
NZS3         EIKHLGGVVDKDAFYFPLDKIVTRRDAEGEIRVNMDNIKKAFNPRDKHYNSQEARSLRSL
NZS4         EIKHLGGVVDKDAFYFPLDKIVTRRDAEGEIRVNMDNIKKAFNPRDKHYNSQEARSLRSL
NZV1         EIKHLGGVVDKDAFYFPLDKIVTRRDAEGEIRVNMDNIKKAFNPRDKHYNSQEARSLRSL
82-029362    EIKHLGGVVDKDAFYFPLDKIVTRRDAEGEIRVNMDNIKKAFNPRDKHYNSQEARSLRSL
6644         EIKHLGGVVDKDAFYFPLDKIVTRRDAEGEIRVNMDNIKKAFNPRDKHYNSQEARSLRSL
HD183        EIKHLGGVVDKDAFYFPLDKIVTRRDAEGEIRVNMDNIKKAFNPRDKHYNSQEARSLRSL
HMC46        EIKHLGGVVDKDAFYFPLDKIVTRRDAEGEIRVNMDNIKKAFNPRDKHYNSQEARSLRSL
HMC56        EIKHLGGVVDKDAFYFPLDKIVTRRDAEGEIRVNMDNIKKAFNPRDKHYNSQEARSLRSL
             ************************************************************

35000HP      YNQDPSMSGTRFIIENQVIANPFSSADLQSYIQAQQSKLPELGRQARRALPELPTAANKG
NZS1         YNQDPSMSGTRFIIENQVIANPFSSADLQSYIQAQQSKLPELGRQARRALPELPTAANKG
NZS2         YNQDPSMSGTRFIIENQVIANPFSSADLQSYIQAQQSKLPELGRQARRALPELPTAANKG
NZS3         YNQDPSMSGTRFIIENQVIANPFSSADLQSYIQAQQSKLPELGRQARRALPELPTAANKG
NZS4         YNQDPSMSGTRFIIENQVIANPFSSADLQSYIQAQQSKLPELGRQARRALPELPTAANKG
NZV1         YNQDPSMSGTRFIIENQVIANPFSSADLQSYIQAQQSKLPELGRQARRALPELPTAANKG
82-029362    YNQDPSMSGTRFIIENQVIANPFSSADLQSYIQAQQSKLPELGRQARRALPELPTAANKG
6644         YNQDPSMSGTRFIIENQVIANPFSSADLQSYIQAQQSKLPELGRQARRALPELPTAANKG
HD183        YNQDPSMSGTRFIIENQVIANPFSSADLQSYIQAQQSKLPELGRQARRALPELPTAANKG
HMC46        YNQDPSMSGTRFIIENQVIANPFSSADLQSYIQAQQSKLPELGRQARRALPELPTAANKG
HMC56        YNQDPSMSGTRFIIENQVIANPFSSADLQSYIQAQQSKLPELGRQARRALPELPTAANKG
             ************************************************************

35000HP      RGSRVEEQNIVTRPRVEDVYATVNKGAKHGEAQQPGSFYTKKLVDQVSHVPNTEPVYADL
NZS1         RGSRVEEQNIVTRPRVEDVYATVNKGAKHGEAQQPGSFYTKKLVDQVSHVPNTEPVYADL
NZS2         RGSRVEEQNIVTRPRVEDVYATVNKGAKHGEAQQPGSFYTKKLVDQVSHVPNTEPVYADL
NZS3         RGSRVEEQNIVTRPRVEDVYATVNKGAKHGEAQQPGSFYTKKLVDQVSHVPNTEPVYADL
NZS4         RGSRVEEQNIVTRPRVEDVYATVNKGAKHGEAQQPGSFYTKKLVDQVSHVPNTEPVYADL
NZV1         RGSRVEEQNIVTRPRVEDVYATVNKGAKHGEAQQPGSFYTKKLVDQVSHVPNTEPVYADL
82-029362    RGSRVEEQNIVTRPRVEDVYATVNKGAKHGEAQQPGSFYTKKLVDQVSHVPTTEPVYADL
6644         RGSRVEEQNIVTRPRVEDVYATVNKGAKHGEAQQPGSFYTKKLVDQVSHVPTTEPVYADL
HD183        RGSRVEEQNIVTRPRVEDVYATVNKGAKHGEAQQPGSFYTKKLVDQVSHVPNTEPVYADL
HMC46        RGSRVEEQNIVTRPRVEDVYATVNKGAKHGEAQQPGSFYTKKLVDQVSHVPTTEPVYADL
HMC56        RGSRVEEQNIVTRPRVEDVYATVNKGAKHGEAQQPGSFYTKKLVDQVSHVPTTEPVYADL
             ***************************************************.********

35000HP      HFNRNGRVVRQTEPEVIYEKIRGQQVEVDDPSSLYAKVNRNRRLDNVEGFYPPEQLRTRS
NZS1         HFNRNGRVVRQTEPEVIYEKIRGQQVEVDDPSSLYAKVNRNRRLDNVEGFYPPEQLRTRS
NZS2         HFNRNGRVVRQTEPEVIYEKIRGQQVEVDDPSSLYAKVNRNRRLDNVEGFYPPEQLRTRS
NZS3         HFNRNGRVVRQTEPEVIYEKIRGQQVEVDDPSSLYAKVNRNRRLDNVEGFYPPEQLRTRS
NZS4         HFNRNGRVVRQTEPEVIYEKIRGQQVEVDDPSSLYAKVNRNRRLDNVEGFYPPEQLRTRS
NZV1         HFNRNGRVVRQTEPEVIYEKIRGQQVEVDDPSSLYAKVNRNRRLDNVEGFYPPEQLRTRS
82-029362    HFNRNGRVVRQTEPEVIYEKIRGQQVEVDDPSSLYAKVNRNRRLDNVEGFYPPEQLRTRS
6644         HFNRNGRVVRQTEPEVIYEKIRGQQVEVDDPSSLYAKVNRNRRLDNVEGFYPPEQLRTRS
HD183        HFNRNGRVVRQTEPEVIYEKIRGQQVEVDDPSSLYAKVNRNRRLDNVEGFYPPEQLRTRS
HMC46        HFNRNGRVVRQTEPEVIYEKIRGQQVEVDDPSSLYAKVNRNRRLDNVEGFYPPEQLRTRS
HMC56        HFNRNGRVVRQTEPEVIYEKIRGQQVEVDDPSSLYAKVNRNRRLDNVEGFYPPEQLRTRS
             ************************************************************

35000HP      DKLAEQVSRVPTTEPVYADLRFKSAEDDYAPALPARPELGNAAGFRKAKVKGEESESTWS
NZS1         DKLAEQVSRVPTTEPVYADLRFKSAEDDYAPALPARPELGNAAGFRKAKVKGEESESTWS
NZS2         DKLAEQVSRVPTTEPVYADLRFKSAEDDYAPALPARPELGNAAGFRKAKVKGEESESTWS
NZS3         DKLAEQVSRVPTTEPVYADLRFKSAEDDYAPALPARPELGNAAGFRKAKVKGEESESTWS
```

```
NZS4          DKLAEQVSRVPTTEPVYADLRFKSAEDDYAPALPARPELGNAAGFRKAKVKGEESESTWS
NZV1          DKLAEQVSRVPTTEPVYADLRFKSAEDDYAPALPARPELGNAAGFRKAKVKGEESESTWS
82-029362     DKLAEQVSRVPTTEPVYADLRFKSAEDDYAPALPARPELGNAAGFRKAKVKGEESESTWS
6644          DKLAEQVSRVPTTEPVYADLRFKSAEDDYAPALPARPELGNAAGFRKAKVKGEESESTWS
HD183         DKLAEQVSRVPTTEPVYADLRFKSAEDDYAPALPARPELGNAAGFRKAKVKGEESESTWS
HMC46         DKLAEQVSRVPTTEPVYADLRFKSAEDDYAPALPARPELGNAAGFRKAKVKGEESESTWS
HMC56         DKLAEQVSRVPTTEPVYADLRFKSAEDDYAPALPARPELGNAAGFRKAKVKGEESESTWS
              ************************************************************

35000HP       RLKHLFSRESGKKAQVEGPEIKHLGGVVDKDAFYFPLDKIVTRRDAEGEIRVNMDNIKKA
NZS1          RLKHLFSRESGKKAQVEGPEIKHLGGVVDKDAFYFPLDKIVTRRDAEGEIRVNMDNIKKA
NZS2          RLKHLFSRESGKKAQVEGPEIKHLGGVVDKDAFYFPLDKIVTRRDAEGEIRVNMDNIKKA
NZS3          RLKHLFSRESGKKAQVEGPEIKHLGGVVDKDAFYFPLDKIVTRRDAEGEIRVNMDNIKKA
NZS4          RLKHLFSRESGKKAQVEGPEIKHLGGVVDKDAFYFPLDKIVTRRDAEGEIRVNMDNIKKA
NZV1          RLKHLFSRESGKKAQVEGPEIKHLGGVVDKDAFYFPLDKIVTRRDAEGEIRVNMDNIKKA
82-029362     RLKHLFSRESGKKAQVEGPEIKHLGGVVDKDAFYFPLDKIVTRRDAEGEIRVNMDNIKKA
6644          RLKHLFSRESGKKAQVEGPEIKHLGGVVDKDAFYFPLDKIVTRRDAEGEIRVNMDNIKKA
HD183         RLKHLFSRESGKKAQVEGPEIKHLGGVVDKDAFYFPLDKIVTRRDAEGEIRVNMDNIKKA
HMC46         RLKHLFSRESGKKAQVEGPEIKHLGGVVDKDAFYFPLDKIVTRRDAEGEIRVNMDNIKKA
HMC56         RLKHLFSRESGKKAQVEGPEIKHLGGVVDKDAFYFPLDKIVTRRDAEGEIRVNMDNIKKA
              ************************************************************

35000HP       FNPRDKHYNSQEARSLRSLYNQDPSMSGTRFIIENQVIANPFSSADLQSYIQAQQSKLPE
NZS1          FNPRDKHYNSQEARSLRSLYNQDPSMSGTRFIIENQVIANPFSSADLQSYIQAQQSKLPE
NZS2          FNPRDKHYNSQEARSLRSLYNQDPSMSGTRFIIENQVIANPFSSADLQSYIQAQQSKLPE
NZS3          FNPRDKHYNSQEARSLRSLYNQDPSMSGTRFIIENQVIANPFSSADLQSYIQAQQSKLPE
NZS4          FNPRDKHYNSQEARSLRSLYNQDPSMSGTRFIIENQVIANPFSSADLQSYIQAQQSKLPE
NZV1          FNPRDKHYNSQEARSLRSLYNQDPSMSGTRFIIENQVIANPFSSADLQSYIQAQQSKLPE
82-029362     FNPRDKHYNSQEARSLRSLYNQDPSMSGTRFIIENQVIANPFSSADLQSYIQAQQSKLPE
6644          FNPRDKHYNSQEARSLRSLYNQDPSMSGTRFIIENQVIANPFSSADLQSYIQAQQSKLPE
HD183         FNPRDKHYNSQEARSLRSLYNQDPSMSGTRFIIENQVIANPFSSADLQSYIQAQQSKLPE
HMC46         FNPRDKHYNSQEARSLRSLYNQDPSMSGTRFIIENQVIANPFSSADLQSYIQAQQSKLPE
HMC56         FNPRDKHYNSQEARSLRSLYNQDPSMSGTRFIIENQVIANPFSSADLQSYIQAQQSKLPE
              ************************************************************

35000HP       LGRQARRALPELPTAANKGRGSRVEEQNIVTRPRVEDVYATVNKGAKHGEAQQPGSFYTK
NZS1          LGRQARRALPELPTAANKGRGSRVEEQNIVTRPRVEDVYATVNKGAKHGEAQQPGSFYTK
NZS2          LGRQARRALPELPTAANKGRGSRVEEQNIVTRPRVEDVYATVNKGAKHGEAQQPGSFYTK
NZS3          LGRQARRALPELPTAANKGRGSRVEEQNIVTRPRVEDVYATVNKGAKHGEAQQPGSFYTK
NZS4          LGRQARRALPELPTAANKGRGSRVEEQNIVTRPRVEDVYATVNKGAKHGEAQQPGSFYTK
NZV1          LGRQARRALPELPTAANKGRGSRVEEQNIVTRPRVEDVYATVNKGAKHGEAQQPGSFYTK
82-029362     LGRQARRALPELPTAANKGRGSRVEEQNIVTRPRVEDVYATVNKGAKHGEAQQPGSFYTK
6644          LGRQARRALPELPTAANKGRGSRVEEQNIVTRPRVEDVYATVNKGAKHGEAQQPGSFYTK
HD183         LGRQARRALPELPTAANKGRGSRVEEQNIVTRPRVEDVYATVNKGAKHGEAQQPGSFYTK
HMC46         LGRQARRALPELPTAANKGRGSRVEEQNIVTRPRVEDVYATVNKGAKHGEAQQPGSFYTK
HMC56         LGRQARRALPELPTAANKGRGSRVEEQNIVTRPRVEDVYATVNKGAKHGEAQQPGSFYTK
              ************************************************************

35000HP       KLVDQVSHVPNTEPVYADLHFNRNGRVVRQTEPEVIYEKIRGQQVEVDDPSSLYAKVNRN
NZS1          KLVDQVSHVPNTEPVYADLHFNRNGRVVRQTEPEVIYEKIRGQQVEVDDPSSLYAKVNRN
NZS2          KLVDQVSHVPNTEPVYADLHFNRNGRVVRQTEPEVIYEKIRGQQVEVDDPSSLYAKVNRN
NZS3          KLVDQVSHVPNTEPVYADLHFNRNGRVVRQTEPEVIYEKIRGQQVEVDDPSSLYAKVNRN
NZS4          KLVDQVSHVPNTEPVYADLHFNRNGRVVRQTEPEVIYEKIRGQQVEVDDPSSLYAKVNRN
NZV1          KLVDQVSHVPNTEPVYADLHFNRNGRVVRQTEPEVIYEKIRGQQVEVDDPSSLYAKVNRN
82-029362     KLVDQVSHVPTTEPVYADLHFNRNGRVVRQTEPEVIYEKIRGQQVEVDDPSSLYAKVNRN
6644          KLVDQVSHVPTTEPVYADLHFNRNGRVVRQTEPEVIYEKIRGQQVEVDDPSSLYAKVNRN
HD183         KLVDQVSHVPNTEPVYADLHFNRNGRVVRQTEPEVIYEKIRGQQVEVDDPSSLYAKVNRN
HMC46         KLVDQVSHVPTTEPVYADLHFNRNGRVVRQTEPEVIYEKIRGQQVEVDDPSSLYAKVNRN
HMC56         KLVDQVSHVPTTEPVYADLHFNRNGRVVRQTEPEVIYEKIRGQQVEVDDPSSLYAKVNRN
              **********.*************************************************

35000HP       RRLDNVEGFYPPEQLRTRSDKLAEQVSRVPTTEPVYADLRFKSAEDDYAPALPARPELGN
NZS1          RRLDNVEGFYPPEQLRTRSDKLAEQVSRVPTTEPVYADLRFKSAEDDYAPALPARPELGN
NZS2          RRLDNVEGFYPPEQLRTRSDKLAEQVSRVPTTEPVYADLRFKSAEDDYAPALPARPELGN
NZS3          RRLDNVEGFYPPEQLRTRSDKLAEQVSRVPTTEPVYADLRFKSAEDDYAPALPARPELGN
NZS4          RRLDNVEGFYPPEQLRTRSDKLAEQVSRVPTTEPVYADLRFKSAEDDYAPALPARPELGN
NZV1          RRLDNVEGFYPPEQLRTRSDKLAEQVSRVPTTEPVYADLRFKSAEDDYAPALPARPELGN
82-029362     RRLDNVEGFYPPEQLRTRSDKLAEQVSRVPTTEPVYADLRFKSAEDDYAPALPARPELGN
6644          RRLDNVEGFYPPEQLRTRSDKLAEQVSRVPTTEPVYADLRFKSAEDDYAPALPARPELGN
HD183         RRLDNVEGFYPPEQLRTRSDKLAEQVSRVPTTEPVYADLRFKSAEDDYAPALPARPELGN
HMC46         RRLDNVEGFYPPEQLRTRSDKLAEQVSRVPTTEPVYADLRFKSAEDDYAPALPARPELGN
HMC56         RRLDNVEGFYPPEQLRTRSDKLAEQVSRVPTTEPVYADLRFKSAEDDYAPALPARPELGN
              ************************************************************
```

```
35000HP       AAGFRKAKVKGEESESTWSRLKHLFSRESGKTKVEEVENEYKSQTNGENSLEIKTIEHIP
NZS1          AAGFRKAKVKGEESESTWSRLKHLFSRESGKTKVEEVENEYKSQTNGENSLEIKTIEHIP
NZS2          AAGFRKAKVKGEESESTWSRLKHLFSRESGKTKVEEVENEYKSQTNGENSLEIKTIEHIP
NZS3          AAGFRKAKVKGEESESTWSRLKHLFSRESGKTKVEEVENEYKSQTNGENSLEIKTIEHIP
NZS4          AAGFRKAKVKGEESESTWSRLKHLFSRESGKTKVEEVENEYKSQTNGENSLEIKTIEHIP
NZV1          AAGFRKAKVKGEESESTWSRLKHLFSRESGKTKVEEVENEYKSQTNGENSLEIKTIEHIP
82-029362     AAGFRKAKVKGEESESTWSRLKHLFSRESGKTKVEEVENEYKSQTNGENSLEIKTIEHIP
6644          AAGFRKAKVKGEESESTWSRLKHLFSRESGKTKVEEVENEYKSQTNGENSLEIKTIEHIP
HD183         AAGFRKAKVKGEESESTWSRLKHLFSRESGKTKVEEVENEYKSQTNGENSLEIKTIEHIP
HMC46         AAGFRKAKVKGEESESTWSRLKHLFSRESGKTKVEEVENEYKSQTNGENSLEIKTIEHIP
HMC56         AAGFRKAKVKGEESESTWSRLKHLFSRESGKTKVEEVENEYKSQTNGENSLEIKTIEHIP
              ************************************************************

35000HP       TRLKNLESPREAQSDLGENALIYGLQRGRQALISKANAADKEGKNAILADSYIGKLNLGF
NZS1          TRLKNLESPREAQSDLGENALIYGLQRGRQALISKANAADKEGKNAILADSYIGKLNLGF
NZS2          TRLKNLESPREAQSDLGENALIYGLQRGRQALISKANAADKEGKNAILADSYIGKLNLGF
NZS3          TRLKNLESPREAQSDLGENALIYGLQRGRQALISKANAADKEGKNAILADSYIGKLNLGF
NZS4          TRLKNLESPREAQSDLGENALIYGLQRGRQALISKANAADKEGKNAILADSYIGKLNLGF
NZV1          TRLKNLESPREAQSDLGENALIYGLQRGRQALISKANAADKEGKNAILADSYIGKLNLGF
82-029362     TRLKNLESPREAQSDLGENALIYGLQRGRQALISKANAADKEGKNAILADSYIGKLNLGF
6644          TRLKNLESPREAQSDLGENALIYGLQRGRQALISKANAADKEGKNAILADSYIGKLNLGF
HD183         TRLKNLESPREAQSDLGENALIYGLQRGRQALISKANAADKEGKNAILADSYIGKLNLGF
HMC46         TRLKNLESPREAQSDLGENALIYGLQRGRQALISKANAADKEGKNAILADSYIGKLNLGF
HMC56         TRLKNLESPREAQSDLGENALIYGLQRGRQALISKANAADKEGKNAILADSYIGKLNLGF
              ************************************************************

35000HP       EFGELTKFAKQVKDGKVTEQDIQNIASFNDETAKLARRSEPKNRINDANVDDNQRIIREL
NZS1          EFGELTKFAKQVKDGKVTEQDIQNIASFNDETAKLARRSEPKNRINDANVDDNQRIIREL
NZS2          EFGELTKFAKQVKDGKVTEQDIQNIASFNDETAKLARRSEPKNRINDANVDDNQRIIREL
NZS3          EFGELTKFAKQVKDGKVTEQDIQNIASFNDETAKLARRSEPKNRINDANVDDNQRIIREL
NZS4          EFGELTKFAKQVKDGKVTEQDIQNIASFNDETAKLARRSEPKNRINDANVDDNQRIIREL
NZV1          EFGELTKFAKQVKDGKVTEQDIQNIASFNDETAKLARRSEPKNRINDANVDDNQRIIREL
82-029362     EFGELTKFAKQVKDGKVTEQDIQNIASFNDETAKLARRSEPKNRINDANVDDNQRIIREL
6644          EFGELTKFAKQVKDGKVTEQDIQNIASFNDETAKLARRSEPKNRINDANVDDNQRIIREL
HD183         EFGELTKFAKQVKDGKVTEQDIQNIASFNDETAKLARRSEPKNRINDANVDDNQRIIREL
HMC46         EFGELTKFAKQVKDGKVTEQDIQNIASFNDETAKLARRSEPKNRINDANVDDNQRIIREL
HMC56         EFGELTKFAKQVKDGKVTEQDIQNIASFNDETAKLARRSEPKNRINDANVDDNQRIIREL
              ************************************************************

35000HP       INNEAAVDALKRIATLSDQEKAMHSTLRANEKFDMDELEESPNYTTAENKSIRDYKDTQK
NZS1          INNEAAVDALKRIATLSDQEKAMHSTLRANEKFDMDELEESPNYTTAENKSIRDYKDTQK
NZS2          INNEAAVDALKRIATLSDQEKAMHSTLRANEKFDMDELEESPNYTTAENKSIRDYKDTQK
NZS3          INNEAAVDALKRIATLSDQEKAMHSTLRANEKFDMDELEESPNYTTAENKSIRDYKDTQK
NZS4          INNEAAVDALKRIATLSDQEKAMHSTLRANEKFDMDELEESPNYTTAENKSIRDYKDTQK
NZV1          INNEAAVDALKRIATLSDQEKAMHSTLRANEKFDMDELEESPNYTTAENKSIRDYKDTQK
82-029362     INNEAAVDALKRIATLSDQEKAMHSTLRANEKFDMDELEESPNYTTAENKSIRDYKDTQK
6644          INNEAAVDALKRIATLSDQEKAMHSTLRANEKFDMDELEESPNYTTAENKSIRDYKDTQK
HD183         INNEAAVDALKRIATLSDQEKAMHSTLRANEKFDMDELEESPNYTTAENKSIRDYKDTQK
HMC46         INNEAAVDALKRIATLSDQEKAMHSTLRANEKFDMDELEESPNYTTAENKSIRDYKDTQK
HMC56         INNEAAVDALKRIATLSDQEKAMHSTLRANEKFDMDELEESPNYTTAENKSIRDYKDTQK
              ************************************************************

35000HP       ALNDARMDFFTEKTKFIAKETLERGGQLYFALDGLVTNSPGFRADTQINMDKLKDVFNPN
NZS1          ALNDARMDFFTEKTKFIAKETLERGGQLYFALDGLVTNSPGFRADTQINMDKLKDVFNPN
NZS2          ALNDARMDFFTEKTKFIAKETLERGGQLYFALDGLVTNSPGFRADTQINMDKLKDVFNPN
NZS3          ALNDARMDFFTEKTKFIAKETLERGGQLYFALDGLVTNSPGFRADTQINMDKLKDVFNPN
NZS4          ALNDARMDFFTEKTKFIAKETLERGGQLYFALDGLVTNSPGFRADTQINMDKLKDVFNPN
NZV1          ALNDARMDFFTEKTKFIAKETLERGGQLYFALDGLVTNSPGFRADTQINMDKLKDVFNPN
82-029362     ALNDARMDFFTEKTKFIAKETLERGGQLYFALDGLVTNSPGFRADTQINMDKLKDVFNPN
6644          ALNDARMDFFTEKTKFIAKETLERGGQLYFALDGLVTNSPGFRADTQINMDKLKDVFNPN
HD183         ALNDARMDFFTEKTKFIAKETLERGGQLYFALDGLVTNSPGFRADTQINMDKLKDVFNPN
HMC46         ALNDARMDFFTEKTKFIAKETLERGGQLYFALDGLVTNSPGFRADTQINMDKLKDVFNPN
HMC56         ALNDARMDFFTEKTKFIAKETLERGGQLYFALDGLVTNSPGFRADTQINMDKLKDVFNPN
              ************************************************************

35000HP       HEHYDSVTSRELRYLYENYKDNPNLKFTLKDHVIANPLKTLKTSISESDLKSSPRRARQE
NZS1          HEHYDSVTSRELRYLYENYKDNPNLKFTLKDHVIANPLKTLKTSISESDLKSSPRRARQE
NZS2          HEHYDSVTSRELRYLYENYKDNPNLKFTLKDHVIANPLKTLKTSISESDLKSSPRRARQE
NZS3          HEHYDSVTSRELRYLYENYKDNPNLKFTLKDHVIANPLKTLKTSISESDLKSSPRRARQE
NZS4          HEHYDSVTSRELRYLYENYKDNPNLKFTLKDHVIANPLKTLKTSISESDLKSSPRRARQE
NZV1          HEHYDSVTSRELRYLYENYKDNPNLKFTLKDHVIANPLKTLKTSISESDLKSSPRRARQE
82-029362     HEHYDSVTSRELRYLYENYKDNPNLKFTLKDHVIANPLKTLKTSISESDLKSSPRRARQE
```

```
6644          HEHYDSVTSRELRYLYENYKDNPNLKFTLKDHVIANPLKTLKTSISESDLKSSPRRARQE
HD183         HEHYDSVTSRELRYLYENYKDNPNLKFTLKDHVIANPLKTLKTSISESDLKSSPRRARQE
HMC46         HEHYDSVTSRELRYLYENYKDNPNLKFTLKDHVIANPLKTLKTSISESDLKSSPRRARQE
HMC56         HEHYDSVTSRELRYLYENYKDNPNLKFTLKDHVIANPLKTLKTSISESDLKSSPRRARQE
              ************************************************************

35000HP       GPSLLQRVRNLFDKSSSNKRSEKDTAQTSVGYRNTNIDIINDKTKGVNHIVENGVEVALT
NZS1          GPSLLQRVRNLFDKSSSNKRSEKDTAQTSVGYRNTNIDIINDKTKGVNHIVENGVEVALT
NZS2          GPSLLQRVRNLFDKSSSNKRSEKDTAQTSVGYRNTNIDIINDKTKGVNHIVENGVEVALT
NZS3          GPSLLQRVRNLFDKSSSNKRSEKDTAQTSVGYRNTNIDIINDKTKGVNHIVENGVEVALT
NZS4          GPSLLQRVRNLFDKSSSNKRSEKDTAQTSVGYRNTNIDIINDKTKGVNHIVENGVEVALT
NZV1          GPSLLQRVRNLFDKSSSNKRSEKDTAQTSVGYRNTNIDIINDKTKGVNHIVENGVEVALT
82-029362     GPSLLQRVRNLFDKSSSNKRSEKDTAQTSVGYRNTNIDIINDKTKGVNHIVENGVEVALT
6644          GPSLLQRVRNLFDKSSSNKRSEKDTAQTSVGYRNTNIDIINDKTKGVNHIVENGVEVALT
HD183         GPSLLQRVRNLFDKSSSNKRSEKDTAQTSVGYRNTNIDIINDKTKGVNHIVENGVEVALT
HMC46         GPSLLQRVRNLFDKSSSNKRSEKDTAQTSVGYRNTNIDIINDKTKGVNHIVENGVEVALT
HMC56         GPSLLQRVRNLFDKSSSNKRSEKDTAQTSVGYRNTNIDIINDKTKGVNHIVENGVEVALT
              ************************************************************

35000HP       NTDYKPLKLPNVEAAFKQTKLKAENIDPHIEAVKKLEIIASSANSIPKEHLLKALIEVTE
NZS1          NTDYKPLKLPNVEAAFKQTKLKAENIDPHIEAVKKLEIIASSANSIPKEHLLKALIEVTE
NZS2          NTDYKPLKLPNVEAAFKQTKLKAENIDPHIEAVKKLEIIASSANSIPKEHLLKALIEVTE
NZS3          NTDYKPLKLPNVEAAFKQTKLKAENIDPHIEAVKKLEIIASSANSIPKEHLLKALIEVTE
NZS4          NTDYKPLKLPNVEAAFKQTKLKAENIDPHIEAVKKLEIIASSANSIPKEHLLKALIEVTE
NZV1          NTDYKPLKLPNVEAAFKQTKLKAENIDPHIEAVKKLEIIASSANSIPKEHLLKALIEVTE
82-029362     NTDYKPLKLPNVEAAFKQTKLKAENIDPHIEAVKKLEIIASSANSIPKEHLLKALIEVTE
6644          NTDYKPLKLPNVEAAFKQTKLKAENIDPHIEAVKKLEIIASSANSIPKEHLLKALIEVTE
HD183         NTDYKPLKLPNVEAAFKQTKLKAENIDPHIEAVKKLEIIASSANSIPKEHLLKALIEVTE
HMC46         NTDYKPLKLPNVEAAFKQTKLKAENIDPHIEAVKKLEIIASSANSIPKEHLLKALIEVTE
HMC56         NTDYKPLKLPNVEAAFKQTKLKAENIDPHIEAVKKLEIIASSANSIPKEHLLKALIEVTE
              ************************************************************

35000HP       GKTDEDINVYQKLFNTRQNISNEVAPTYSLRNLDGKDGKQILRSVAEIYKNLPLSDTYQA
NZS1          GKTDEDINVYQKLFNTRQNISNEVAPTYSLRNLDGKDGKQILRSVAEIYKNLPLSDTYQA
NZS2          GKTDEDINVYQKLFNTRQNISNEVAPTYSLRNLDGKDGKQILRSVAEIYKNLPLSDTYQA
NZS3          GKTDEDINVYQKLFNTRQNISNEVAPTYSLRNLDGKDGKQILRSVAEIYKNLPLSDTYQA
NZS4          GKTDEDINVYQKLFNTRQNISNEVAPTYSLRNLDGKDGKQILRSVAEIYKNLPLSDTYQA
NZV1          GKTDEDINVYQKLFNTRQNISNEVAPTYSLRNLDGKDGKQILRSVAEIYKNLPLSDTYQA
82-029362     GKTDEDINVYQKLFNTRQNISNEVAPTYSLRNLDGKDGKQILRSVAEIYKNLPLSDTYQA
6644          GKTDEDINVYQKLFNTRQNISNEVAPTYSLRNLDGKDGKQILRSVAEIYKNLPLSDTYQA
HD183         GKTDEDINVYQKLFNTRQNISNEVAPTYSLRNLDGKDGKQILRSVAEIYKNLPLSDTYQA
HMC46         GKTDEDINVYQKLFNTRQNISNEVAPTYSLRNLDGKDGKQILRSVAEIYKNLPLSDTYQA
HMC56         GKTDEDINVYQKLFNTRQNISNEVAPTYSLRNLDGKDGKQILRSVAEIYKNLPLSDTYQA
              ************************************************************

35000HP       VRNYVNNRLIEKLSSNRLLLEHLANSKISGNEYAIKYIFDTVSRAKQEIFEQELNTELAP
NZS1          VRNYVNNRLIEKLSSNRLLLEHLANSKISGNEYAIKYIFDTVSRAKQEIFEQELNTELAP
NZS2          VRNYVNNRLIEKLSSNRLLLEHLANSKISGNEYAIKYIFDTVSRAKQEIFEQELNTELAP
NZS3          VRNYVNNRLIEKLSSNRLLLEHLANSKISGNEYAIKYIFDTVSRAKQEIFEQELNTELAP
NZS4          VRNYVNNRLIEKLSSNRLLLEHLANSKISGNEYAIKYIFDTVSRAKQEIFEQELNTELAP
NZV1          VRNYVNNRLIEKLSSNRLLLEHLANSKISGNEYAIKYIFDTVSRAKQEIFEQELNTELAP
82-029362     VRNYVNNRLIEKLSSNRLLLEHLANSKISGNEYAIKYIFDTVSRAKQEIFEQELNTELAP
6644          VRNYVNNRLIEKLSSNRLLLEHLANSKISGNEYAIKYIFDTVSRAKQEIFEQELNTELAP
HD183         VRNYVNNRLIEKLSSNRLLLEHLANSKISGNEYAIKYIFDTVSRAKQEIFEQELNTELAP
HMC46         VRNYVNNRLIEKLSSNRLLLEHLANSKISGNEYAIKYIFDTVSRAKQEIFEQELNTELAP
HMC56         VRNYVNNRLIEKLSSNRLLLEHLANSKISGNEYAIKYIFDTVSRAKQEIFEQELNTELAP
              ************************************************************

35000HP       VSLDIMRRKAANILSSEHGSYKDGTLSIYDKPVKSAFHSRLKNNGEVLNTIVHELTHHEQ
NZS1          VSLDIMRRKAANILSSEHGSYKDGTLSIYDKPVKSAFHSRLKNNGEVLNTIVHELTHHEQ
NZS2          VSLDIMRRKAANILSSEHGSYKDGTLSIYDKPVKSAFHSRLKNNGEVLNTIVHELTHHEQ
NZS3          VSLDIMRRKAANILSSEHGSYKDGTLSIYDKPVKSAFHSRLKNNGEVLNTIVHELTHHEQ
NZS4          VSLDIMRRKAANILSSEHGSYKDGTLSIYDKPVKSAFHSRLKNNGEVLNTIVHELTHHEQ
NZV1          VSLDIMRRKAANILSSEHGSYKDGTLSIYDKPVKSAFHSRLKNNGEVLNTIVHELTHHEQ
82-029362     VSLDIMRRKAANILSSEHGSYKDGTLSIYDKPVKSAFHSRLKNNGEVLNTIVHELTHHEQ
6644          VSLDIMRRKAANILSSEHGSYKDGTLSIYDKPVKSAFHSRLKNNGEVLNTIVHELTHHEQ
HD183         VSLDIMRRKAANILSSEHGSYKDGTLSIYDKPVKSAFHSRLKNNGEVLNTIVHELTHHEQ
HMC46         VSLDIMRRKAANILSSEHGSYKDGTLSIYDKPVKSAFHSRLKNNGEVLNTIVHELTHHEQ
HMC56         VSLDIMRRKAANILSSEHGSYKDGTLSIYDKPVKSAFHSRLKNNGEVLNTIVHELTHHEQ
              ************************************************************

35000HP       DALAKIIDNKGYDAKLFDKNNILYITGGLGYPKQALERDAFLSGDSVSEAFMKKAKEYHE
NZS1          DALAKIIDNKGYDAKLFDKNNILYITGGLGYPKQALERDAFLSGDSVSEAFMKKAKEYHE
```

```
NZS2           DALAKIIDNKGYDAKLFDKNNILYITGGLGYPKQALERDAFLSGDSVSEAFMKKAKEYHE
NZS3           DALAKIIDNKGYDAKLFDKNNILYITGGLGYPKQALERDAFLSGDSVSEAFMKKAKEYHE
NZS4           DALAKIIDNKGYDAKLFDKNNILYITGGLGYPKQALERDAFLSGDSVSEAFMKKAKEYHE
NZV1           DALAKIIDNKGYDAKLFDKNNILYITGGLGYPKQALERDAFLSGDSVSEAFMKKAKEYHE
82-029362      DALAKIIDNKGYDAKLFDKNNILYITGGLGYPKQALERDAFLSGDSVSEAFMKKAKEYHE
6644           DALAKIIDNKGYDAKLFDKNNILYITGGLGYPKQALERDAFLSGDSVSEAFMKKAKEYHE
HD183          DALAKIIDNKGYDAKLFDKNNILYITGGLGYPKQALERDAFLSGDSVSEAFMKKAKEYHE
HMC46          DALAKIIDNKGYDAKLFDKNNILYITGGLGYPKQALERDAFLSGDSVSEAFMKKAKEYHE
HMC56          DALAKIIDNKGYDAKLFDKNNILYITGGLGYPKQALERDAFLSGDSVSEAFMKKAKEYHE
               ************************************************************

35000HP        RTKQERKDAKKDEARIAKLYKQWEQEEANKKSASLNGSSQSLDSRSEVEFNRVSHKSVR*
NZS1           RTKQERKDAKKDEARIAKLYKQWEQEEANKKSASLNGSSQSLDSRSEVEFNRVSHKSVR*
NZS2           RTKQERKDAKKDEARIAKLYKQWEQEEANKKSASLNGSSQSLDSRSEVEFNRVSHKSVR*
NZS3           RTKQERKDAKKDEARIAKLYKQWEQEEANKKSASLNGSSQSLDSRSEVEFNRVSHKSVR*
NZS4           RTKQERKDAKKDEARIAKLYKQWEQEEANKKSASLNGSSQSLDSRSEVEFNRVSHKSVR*
NZV1           RTKQERKDAKKDEARIAKLYKQWEQEEANKKSASLNGSSQSLDSRSEVEFNRVSHKSVR*
82-029362      RTKQERKDAKKDEARIAKLYKQWEQEEANKKSASLNGSSQSLDSRSEVEFNRVSHKSVR*
6644           RTKQERKDAKKDEARIAKLYKQWEQEEANKKSASLNGSSQSLDSRSEVEFNRVSHKSVR*
HD183          RTKQERKDAKKDEARIAKLYKQWEQEEANKKSASLNGSSQSLDSRSEVEFNRVSHKSVR*
HMC46          RTKQERKDAKKDEARIAKLYKQWEQEEANKKSASLNGSSQSLDSRSEVEFNRVSHKSVR*
HMC56          RTKQERKDAKKDEARIAKLYKQWEQEEANKKSASLNGSSQSLDSRSEVEFNRVSHKSVR*
               ************************************************************
```

**Note:** Due to difficulty in sorting the reads of *lspA1* and *lspA2* genes of class II strains, LspA2 sequences of class II strains were excluded from this analysis.

# 14) LuxS (HD0370)

```
35000HP        MPLLDSFKVDHTRMNAPAVRVAKTITTPKGDLITVFDLRFCRPNMEIMSSKGIHTLEHLY
NZS1           MPLLDSFKVDHTRMNAPAVRVAKTITTPKGDLITVFDLRFCRPNMEIMSSKGIHTLEHLY
NZS2           MPLLDSFKVDHTRMNAPAVRVAKTITTPKGDLITVFDLRFCRPNMEIMSSKGIHTLEHLY
NZS3           MPLLDSFKVDHTRMNAPAVRVAKTITTPKGDLITVFDLRFCRPNMEIMSSKGIHTLEHLY
NZS4           MPLLDSFKVDHTRMNAPAVRVAKTITTPKGDLITVFDLRFCRPNMEIMSSKGIHTLEHLY
NZV1           MPLLDSFKVDHTRMNAPAVRVAKTITTPKGDLITVFDLRFCRPNMEIMSSKGIHTLEHLY
82-029362      MPLLDSFKVDHTRMNAPAVRVAKTITTPKGDLITVFDLRFCRPNMEIMSSKGIHTLEHLY
6644           MPLLDSFKVDHTRMNAPAVRVAKTITTPKGDLITVFDLRFCRPNMEIMSSKGIHTLEHLY
HMC46          MPLLDSFKVDHTRMNAPAVRVAKTITTPKGDLITVFDLRFCRPNMEIMSSKGIHTLEHLY
HMC56          MPLLDSFKVDHTRMNAPAVRVAKTITTPKGDLITVFDLRFCRPNMEIMSSKGIHTLEHLY
33921          MPLLDSFKVDHTRMNAPAVRVAKTITTPKGDLITVFDLRFCRPNMEIMSSKGIHTLEHLY
CIP542         MPLLDSFKVDHTRMNAPAVRVAKTITTPKGDLITVFDLRFCRPNMEIMSSKGIHTLEHLY
DMC64          MPLLDSFKVDHTRMNAPAVRVAKTITTPKGDLITVFDLRFCRPNMEIMSSKGIHTLEHLY
DMC111         MPLLDSFKVDHTRMNAPAVRVAKTITTPKGDLITVFDLRFCRPNMEIMSSKGIHTLEHLY
               ************************************************************

35000HP        AGFMRDHLNSDKVEIIDISPMGCRTGFYMSLIGEPSAQAVANAWKNAMHDILTKVSDVTQ
NZS1           AGFMRDHLNSDKVEIIDISPMGCRTGFYMSLIGEPSAQAVANAWKNAMHDILTKVSDVTQ
NZS2           AGFMRDHLNSDKVEIIDISPMGCRTGFYMSLIGEPSAQAVANAWKNAMHDILTKVSDVTQ
NZS3           AGFMRDHLNSDKVEIIDISPMGCRTGFYMSLIGEPSAQAVANAWKNAMHDILTKVSDVTQ
NZS4           AGFMRDHLNSDKVEIIDISPMGCRTGFYMSLIGEPSAQAVANAWKNAMHDILTKVSDVTQ
NZV1           AGFMRDHLNSDKVEIIDISPMGCRTGFYMSLIGEPSAQAVANAWKNAMHDILTKVSDVTQ
82-029362      AGFMRDHLNSDKVEIIDISPMGCRTGFYMSLIGEPSAQAVTNAWKNAMHDILTKVSDVTQ
6644           AGFMRDHLNSDKVEIIDISPMGCRTGFYMSLIGEPSAQAVTNAWKNAMHDILTKVSDVTQ
HMC46          AGFMRDHLNSDKVEIIDISPMGCRTGFYMSLIGEPSAQAVTNAWKNAMHDILTKVSDVTQ
HMC56          AGFMRDHLNSDKVEIIDISPMGCRTGFYMSLIGEPSAQAVTNAWKNAMHDILTKVSDVTQ
33921          AGFMRDHLNSDKVEIIDISPMGCRTGFYMSLIGEPSAQAVANAWKNAMHDILTKVSDVTQ
CIP542         AGFMRDHLNSDKVEIIDISPMGCRTGFYMSLIGEPSAQAVANAWKNAMHDILTKVSDVTQ
DMC64          AGFMRDHLNSDKVEIIDISPMGCRTGFYMSLIGEPSAQAVANAWKNAMHDILTKVSDVTQ
DMC111         AGFMRDHLNSDKVEIIDISPMGCRTGFYMSLIGEPSAQAVANAWKNAMHDILTKVSDVTQ
               *****************************************:******************

35000HP        IPELNIYQCGTFSEHSLEDAHQIARDVLAKGISVNLNEDLTLDEEWLNR*
NZS1           IPELNIYQCGTFSEHSLEDAHQIARDVLAKGISVNLNEDLTLDEEWLNR*
NZS2           IPELNIYQCGTFSEHSLEDAHQIARDVLAKGISVNLNEDLTLDEEWLNR*
NZS3           IPELNIYQCGTFSEHSLEDAHQIARDVLAKGISVNLNEDLTLDEEWLNR*
NZS4           IPELNIYQCGTFSEHSLEDAHQIARDVLAKGISVNLNEDLTLDEEWLNR*
NZV1           IPELNIYQCGTFSEHSLEDAHQIARDVLAKGISVNLNEDLTLDEEWLNR*
82-029362      IPELNIYQCGTFSEHSLEDAHQIARDVLAKGISVNLNEDLTLDEEWLNR*
6644           IPELNIYQCGTFSEHSLEDAHQIARDVLAKGISVNLNEDLTLDEEWLNR*
HMC46          IPELNIYQCGTFSEHSLEDAHQIARDVLAKGISVNLNEDLTLDEEWLNR*
HMC56          IPELNIYQCGTFSEHSLEDAHQIARDVLAKGISVNLNEDLTLDEEWLNR*
33921          IPELNIYQCGTFSEHSLEDAHQIARDVLAKGISINLNEDLTLDEEWLNR*
```

```
CIP542         IPELNIYQCGTFSEHSLEDAHQIARDVLAKGISINLNEDLTLDEEWLNR*
DMC64          IPELNIYQCGTFSEHSLEDAHQIARDVLAKGISINLNEDLTLDEEWLNR*
DMC111         IPELNIYQCGTFSEHSLEDAHQIARDVLAKGISINLNEDLTLDEEWLNR*
               ********************************:*****************
```

# 15) NcaA (HD1920)

```
33921        MKKIITILCLACGFSNFGHAIVTPGGIPPKCYEFLYPKGTNVLSKLQTTPNDNQLEQINL
CIP542       MKKIITILCLACGFSNFGHAIVTPGGIPPKCYEFLYPKGTNVLSKLQTTPNDNQLEQINL
DMC64        MKKIITILCLACGFSNFGHAIVTPGGIPPKCYEFLYPKGTNVLSKLQTTPNDNQLEQINL
DMC111       MKKIITILCLACGFSNFGHAIVTPGGIPPKCYEFLYPKGTNVLSKLQTTPNDNQLEQINL
35000HP      MKKIIIVSCLMSGVLNFGYSITTES--IPTCSSFL--------SKLLSTAKSDTLSKVKL
82-0369      MKKIIIVSCLMSGVLNFGYSITTES--IPTCSSFL--------SKLLSTAKSDTLSKVKL
6644         MKKIIIVSCLMSGVLNFGYSITTES--IPTCSSFL--------SKLLSTAKSDTLSKVKL
HMC46        MKKIIIVSCLMSGVLNFGYSITTES--IPTCSSFL--------SKLLSTAKSDTLSKVKL
HMC56        MKKIIIVSCLMSGVLNFGYSITTES--IPTCSSFL--------SKLLSTAKSDTLSKVKL
             ***** : ** .*. ***::*.* .   *.* .**        *** :* :.: *.:::*

33921        SDCKLDF----------PWSP---------LNTFADYPENRRIETLLDLWRVVRWNRGYL
CIP542       SDCKLDF----------PWSP---------LNTFADYPENRRIETLLDLWRVVRWNRGYL
DMC64        SDCKLDF----------PWSP---------LNTFADYPENRRIETLLDLWRVVRWNRGYL
DMC111       SDCKLDF----------PWSP---------LNTFADYPENRRIETLLDLWRVVRWNRGYL
35000HP      ENCTFEEFKNNYDKDKFPVEFRNNQEEKSFYRHLKERDNYAGISNLYDMWKWVRFNRGFL
82-0369      ENCTFEEFKNNYDKDKFPVEFRNNQEEKSFYRHLKERDNYAGISNLYDMWKWVRFNRGFL
6644         ENCTFEEFKNNYDKDKFPVEFRNNQEEKSFYRHLKERDNYAGISNLYDMWKWVRFNRGFL
HMC46        ENCTFEEFKNNYDKDKFPVEFRNNQEEKSFYRHLKERDNYAGISNLYDMWKWVRFNRGFL
HMC56        ENCTFEEFKNNYDKDKFPVEFRNNQEEKSFYRHLKERDNYAGISNLYDMWKWVRFNRGFL
             .:*.::           * .           . : :  :   *..* *:*: **:***:*

33921        QEHNANLYDLYQRMNALESLSG----------LGNSESIAEFTKTIYPLIG---------
CIP542       QEHNANLYDLYQRMNALESLSG----------LGNSESIAEFTKTIYPLIG---------
DMC64        QEHNANLYDLYQRMNALESLSG----------LGNSESIAEFTKTIYPLIG---------
DMC111       QEHNANLYDLYQRMNALESLSG----------LGNSESIAEFTKTIYPLIG---------
35000HP      EQQNEKLFHVYDKLNEVGINPESLDSMSYLNLKGINENINALTESINPSVEAIKYLLPDF
82-0369      EQQNEKLFHVYDKLNEVGINPESLDSMSYLNLKRINENMNALTESINPSVEAIKYLLPDF
6644         EQQNEKLFHVYDKLNEVGINPESLDSMSYLNLKRINENMNALTESINPSVEAIKYLLPDF
HMC46        EQQNEKLFHVYDKLNEVGINPESLDSMSYLNLKRINENMNALTESINPSVEAIKYLLPDF
HMC56        EQQNEKLFHVYDKLNEVGINPESLDSMSYLNLKRINENMNALTESINPSVEAIKYLLPDF
             :::* :*:.:*:::* :                  .*.:  :*::* * :

33921        --------------------------------GEILKQVNQKVHELRKETYMNTANTAAM
CIP542       --------------------------------GEILKQVNQKVHELRKETYMNTANTAAM
DMC64        --------------------------------GEILKQVNQKVHELRKETYMNTANTAAM
DMC111       --------------------------------GEILKQVNQKVHELRKETYMNTANTAAM
35000HP      EKYVAAYEKLAKEGDSLSYSDFKKASGRGATTANIQQIDQRILHQFRKEMHMNTANTAAM
82-0369      EKYVAAYEKLAKEGDSLSYSDFKKASGRGATTANIQQIDQRILHQFRKEMHMNTANTAAM
6644         EKYVAAYEKLAKEGDSLSYSDFKKASGRGATTANIQQIDQRILHQFRKEMHMNTANTAAM
HMC46        EKYVAAYEKLAKEGDSLSYSDFKKASGRGATTANIQQIDQRILHQFRKEMHMNTANTAAM
HMC56        EKYVAAYEKLAKEGDSLSYSDFKKASGRGATTANIQQIDQRILHQFRKEMHMNTANTAAM
                                             .:* :  :: :*::*** :*********

33921        SSLNFGNSQGISFGAAIGGHKGQHSLALGTAYTDYQTQVNVKIALPVRQPKPSNITYGIG
CIP542       SSLNFGNSQGISFGAAIGGHKGQHSLALGTAYTDYQTQVNVKIALPVRQPKPSNITYGIG
DMC64        SSLNFGNSQGISFGAAIGGHKGQHSLALGTAYTDYQTQVNVKIALPVRQPKPSNITYGIG
DMC111       SSLNFGNSQGISFGAAIGGHKGQHSLALGTAYTDYQTQVNVKIALPVRQPKPSNITYGIG
35000HP      SSLNFGNGYGVSVGAAIGGHKGQYSLALGTAYTDYQTQVNVKIALPVKQPKPSNITYGVG
82-0369      SSLNFGNGYGVSVGAAIGGHKGQYSLALGTAYTDYQTQVNVKIALPVKQPKPSNITYGVG
6644         SSLNFGNGYGVSVGAAIGGHKGQYSLALGTAYTDYQTQVNVKIALPVKQPKPSNITYGVG
HMC46        SSLNFGNGYGVSVGAAIGGHKGQYSLALGTAYTDYQTQVNVKIALPVKQPKPSNITYGVG
HMC56        SSLNFGNGYGVSVGAAIGGHKGQYSLALGTAYTDYQTQVNVKIALPVKQPKPSNITYGVG
             *******. *:*.**********:***********************:**********:*

33921        FVYNFQ*
CIP542       FVYNFQ*
DMC64        FVYNFQ*
DMC111       FVYNFQ*
35000HP      FVYNFQ*
82-0369      FVYNFQ*
6644         FVYNFQ*
HMC46        FVYNFQ*
HMC56        FVYNFQ*
             *******
```

**Note:** The NcaA sequence of NZS1, NZS2, NZS3, NZS4, and NZV1 were identical to that of 35000HP.

# 16) PAL (HD1772)

```
5000HP       MKKIAKVLMIAAPAFVLTACSSSSGKTDANANMNGDAMAVNQFGGMTTEDLQTRYNTVYF
33921        MKKIAKVLMIAAPAFVLTACSSSSGKTDANANMNGDAMAVNQFGGMTSEDLQTRYNTVYF
CIP542       MKKIAKVLMIAAPAFVLTACSSSSGKTDANANMNGDAMAVNQFGGMTSEDLQTRYNTVYF
DMC64        MKKIAKVLMIAAPAFVLTACSSSSGKTDANANMNGDAMAVNQFGGMTSEDLQTRYNTVYF
DMC111       MKKIAKVLMIAAPAFVLTACSSSSGKTDANANMNGDAMAVNQFGGMTSEDLQTRYNTVYF
             ***********************************************:************

35000HP      NFDSYAVEGEYRQLLDAHAAYLTSSNSKVTVTGHADERGTPEYNIALGQRRADAVKNYLA
33921        NFDSYAVEGEYRQLLDAHAAYLTSSNSKVTVTGHADERGTPEYNIALGQRRADAVKNYLA
CIP542       NFDSYAVEGEYRQLLDAHAAYLTSSNSKVTVTGHADERGTPEYNIALGQRRADAVKNYLA
DMC64        NFDSYAVEGEYRQLLDAHAAYLTSSNSKVTVTGHADERGTPEYNIALGQRRADAVKNYLA
DMC111       NFDSYAVEGEYRQLLDAHAAYLTSSNSKVTVTGHADERGTPEYNIALGQRRADAVKNYLA
             ************************************************************

35000HP      TKGVSQVSTVSYGEEKPSVLGHTEADYAKNRRAVLEY*
33921        TKGVSQVSTVSYGEEKPSVLGHTEADYAKNRRAVLEY*
CIP542       TKGVSQVSTVSYGEEKPSVLGHTEADYAKNRRAVLEY*
DMC64        TKGVSQVSTVSYGEEKPSVLGHTEADYAKNRRAVLEY*
DMC111       TKGVSQVSTVSYGEEKPSVLGHTEADYAKNRRAVLEY*
             **************************************
```

**Note:** The PAL sequence of NZS1, NZS2, NZS3, NZS4, NZV1, 82-029362, 6644, HD183, HMC46, and HMC56 were identical to that of 35000HP.

# 17) RelA (HD1185)

```
33921          MVAIRRSHELDPSTFELASWSASLQMSPITFEELQIAWRYIHEKLDTDSYHLMWDGVEMV
CIP542         MVAIRRSHELDPSTFELASWSASLQMSPITFEELQIAWRYIHEKLDTDSYHLMWDGVEMV
DMC64          MVAIRRSHELDPSTFELASWSASLQMSPITFEELQIAWRYIHEKLDTDSYHLMWDGVEMV
DMC111         MVAIRRSHELDPSTFELASWSASLQMSPITFEELQIAWRYIHEKLDTDSYHLMWDGVEMV
NZV1           MVAIRRSHELDPSTFELASWSASLQMSPITFEELQIAWRYIHEKLDTDSYHLMWDGVEMV
35000HP        MVAIRRSHELDPSTFELASWSASLQMSPITFEELQIAWRYIHEKLDTDSYHLMWDGVEMV
NZS1           MVAIRRSHELDPSTFELASWSASLQMSPITFEELQIAWRYIHEKLDTDSYHLMWDGVEMV
NZS2           MVAIRRSHELDPSTFELASWSASLQMSPITFEELQIAWRYIHEKLDTDSYHLMWDGVEMV
NZS3           MVAIRRSHELDPSTFELASWSASLQMSPITFEELQIAWRYIHEKLDTDSYHLMWDGVEMV
NZS4           MVAIRRSHELDPSTFELASWSASLQMSPITFEELQIAWRYIHEKLDTDSYHLMWDGVEMV
82-029362      MVAIRRSHELDPSTFELASWSASLQMSPITFEELQIAWRYIHEKLDTDSYHLMWDGVEMV
6644           MVAIRRSHELDPSTFELASWSASLQMSPITFEELQIAWRYIHEKLDTDSYHLMWDGVEMV
HMC46          MVAIRRSHELDPSTFELASWSASLQMSPITFEELQIAWRYIHEKLDTDSYHLMWDGVEMV
HMC56          MVAIRRSHELDPSTFELASWSASLQMSPITFEELQIAWRYIHEKLDTDSYHLMWDGVEMV
               ************************************************************

33921          ELLHGLNMDDDSLVAALLFPLVKNNIVDLAQVKEEFSNQVKNLVKGVIEMDNIRQLNANS
CIP542         ELLHGLNMDDDSLVAALLFPLVKNNIVDLAQVKEEFSNQVKNLVKGVIEMDNIRQLNANS
DMC64          ELLHGLNMDDDSLVAALLFPLVKNNIVDLAQVKEEFSNQVKNLVKGVIEMDNIRQLNANS
DMC111         ELLHGLNMDDDSLVAALLFPLVKNNIVDLAQVKEEFSNQVKNLVKGVIEMDNIRQLNANS
NZV1           ELLHGLNMDDDSLVAALLFPLVKNNIVDLAQVKEEFSNQVKNLVKGVIEMDNIRQLNANS
35000HP        ELLHGLNMDDDSLVAALLFPLVKNNIVDLAQVKEEFSNQVKNLVKGVIEMDNIRQLNASS
NZS1           ELLHGLNMDDDSLVAALLFPLVKNNIVDLAQVKEEFSNQVKNLVKGVIEMDNIRQLNANS
NZS2           ELLHGLNMDDDSLVAALLFPLVKNNIVDLAQVKEEFSNQVKNLVKGVIEMDNIRQLNANS
NZS3           ELLHGLNMDDDSLVAALLFPLVKNNIVDLAQVKEEFSNQVKNLVKGVIEMDNIRQLNANS
NZS4           ELLHGLNMDDDSLVAALLFPLVKNNIVDLAQVKEEFSNQVKNLVKGVIEMDNIRQLNANS
82-029362      ELLHGLNMDDDSLVAALLFPLVKNNIVDLAQVKEEFSNQVKNLVKGVIEMDNIRQLNANS
6644           ELLHGLNMDDDSLVAALLFPLVKNNIVDLAQVKEEFSNQVKNLVKGVIEMDNIRQLNANS
HMC46          ELLHGLNMDDDSLVAALLFPLVKNNIVDLAQVKEEFSNQVKNLVKGVIEMDNIRQLNANS
HMC56          ELLHGLNMDDDSLVAALLFPLVKNNIVDLAQVKEEFSNQVKNLVKGVIEMDNIRQLNANS
               **********************************************************.*

33921          RSDFQIDNIRRMLLAMVDDFRCVVIKLAERIAYLREIHRYTEEDLVLAAKECSHIYAPLA
CIP542         RSDFQIDNIRRMLLAMVDDFRCVVIKLAERIAYLREIHRYTEEDLVLAAKECSHIYAPLA
DMC64          RSDFQIDNIRRMLLAMVDDFRCVVIKLAERIAYLREIHRYTEEDLVLAAKECSHIYAPLA
DMC111         RSDFQIDNIRRMLLAMVDDFRCVVIKLAERIAYLREIHRYTEEDLVLAAKECSHIYAPLA
NZV1           RSDFQIDNIRRMLLAMVDDFRCVVIKLAERIAYLREIHRYTEEDLVLAAKECSHIYAPLA
35000HP        RSDFQIDNIRRMLLAMVDDFRCVVIKLAERIAYLREIHRYTEEDLVLAAKECSHIYAPLA
NZS1           RSDFQIDNIRRMLLAMVDDFRCVVIKLAERIAYLREIHRYTEEDLVLAAKECSHIYAPLA
```

```
NZS2          RSDFQIDNIRRMLLAMVDDFRCVVIKLAERIAYLREIHRYTEEDLVLAAKECSHIYAPLA
NZS3          RSDFQIDNIRRMLLAMVDDFRCVVIKLAERIAYLREIHRYTEEDLVLAAKECSHIYAPLA
NZS4          RSDFQIDNIRRMLLAMVDDFRCVVIKLAERIAYLREIHRYTEEDLVLAAKECSHIYAPLA
82-029362     RSDFQIDNIRRMLLAMVDDFRCVVIKLAERIAYLREIHRYTEEDLVLAAKECSHIYAPLA
6644          RSDFQIDNIRRMLLAMVDDFRCVVIKLAERIAYLREIHRYTEEDLVLAAKECSHIYAPLA
HMC46         RSDFQIDNIRRMLLAMVDDFRCVVIKLAERIAYLREIHRYTEEDLVLAAKECSHIYAPLA
HMC56         RSDFQIDNIRRMLLAMVDDFRCVVIKLAERIAYLREIHRYTEEDLVLAAKECSHIYAPLA
              ************************************************************

33921         NRLGIGQLKWELEDYCFRVLHPQSYRRIATQLGERRLERENYIANFVSQLTASLTEEVDT
CIP542        NRLGIGQLKWELEDYCFRVLHPQSYRRIATQLGERRLERENYIANFVSQLTASLTEEVDT
DMC64         NRLGIGQLKWELEDYCFRVLHPQSYRRIATQLGERRLERENYIANFVSQLTASLTEEVDT
DMC111        NRLGIGQLKWELEDYCFRVLHPQSYRRIATQLGERRLERENYIANFVSQLTASLTEEVDT
NZV1          NRLGIGQLKWELEDYCFRVLHPQSYRRIATQLGERRLERENYIANFVSQLTASLTEEVDT
35000HP       NRLGIGQLKWELEDYCFRVLHPQSYRRIATQLGERRLERENYIANFVSQLTASLTEEVDT
NZS1          NRLGIGQLKWELEDYCFRVLHPQSYRRIATQLGERRLERENYIANFVSQLTASLTEEVDT
NZS2          NRLGIGQLKWELEDYCFRVLHPQSYRRIATQLGERRLERENYIANFVSQLTASLTEEVDT
NZS3          NRLGIGQLKWELEDYCFRVLHPQSYRRIATQLGERRLERENYIANFVSQLTASLTEEVDT
NZS4          NRLGIGQLKWELEDYCFRVLHPQSYRRIATQLGERRLERENYIANFVSQLTASLTEEVDT
82-029362     NRLGIGQLKWELEDYCFRVLHPQSYRRIATQLGERRLERENYIANFVSQLTASLTEEVDT
6644          NRLGIGQLKWELEDYCFRVLHPQSYRRIATQLGERRLERENYIANFVSQLTASLTEEVDT
HMC46         NRLGIGQLKWELEDYCFRVLHPQSYRRIATQLGERRLERENYIANFVSQLTASLTEEVDT
HMC56         NRLGIGQLKWELEDYCFRVLHPQSYRRIATQLGERRLERENYIANFVSQLTASLTEEVDT
              ************************************************************

33921         LEVYGRPKHIYSIWKKMQKKNVQFEQLFDIRAVRVIVPNVQDCYSVLGIIHTQYKHLPEH
CIP542        LEVYGRPKHIYSIWKKMQKKNVQFEQLFDIRAVRVIVPNVQDCYSVLGIIHTQYKHLPEH
DMC64         LEVYGRPKHIYSIWKKMQKKNVQFEQLFDIRAVRVIVPNVQDCYSVLGIIHTQYKHLPEH
DMC111        LEVYGRPKHIYSIWKKMQKKNVQFEQLFDIRAVRVIVPNVQDCYSVLGIIHTQYKHLPEH
NZV1          LEVYGRPKHIYSIWKKMQKKNVQFEQLFDIRAVRVIVPNVQDCYSVLGIIHTQYKHLPEH
35000HP       LEVYGRPKHIYSIWKKMQKKNVQFEQLFDIRAVRVIVPNVQDCYSVLGIIHTQYKHLPEH
NZS1          LEVYGRPKHIYSIWKKMQKKNVQFEQLFDIRAVRVIVPNVQDCYSVLGIIHTQYKHLPEH
NZS2          LEVYGRPKHIYSIWKKMQKKNVQFEQLFDIRAVRVIVPNVQDCYSVLGIIHTQYKHLPEH
NZS3          LEVYGRPKHIYSIWKKMQKKNVQFEQLFDIRAVRVIVPNVQDCYSVLGIIHTQYKHLPEH
NZS4          LEVYGRPKHIYSIWKKMQKKNVQFEQLFDIRAVRVIVPNVQDCYSVLGIIHTQYKHLPEH
82-029362     LEVYGRPKHIYSIWKKMQKKNVQFEQLFDIRAVRVIVPNVQDCYSVLGIIHTQYKHLPEH
6644          LEVYGRPKHIYSIWKKMQKKNVQFEQLFDIRAVRVIVPNVQDCYSVLGIIHTQYKHLPEH
HMC46         LEVYGRPKHIYSIWKKMQKKNVQFEQLFDIRAVRVIVPNVQDCYSVLGIIHTQYKHLPEH
HMC56         LEVYGRPKHIYSIWKKMQKKNVQFEQLFDIRAVRVIVPNVQDCYSVLGIIHTQYKHLPEH
              ************************************************************

33921         FDDYIAHPKPNGYQSIHTVVLGEGEQPIEVQIRTRKMHDEAELGIAAHWKYKEGQTVSRS
CIP542        FDDYIAHPKPNGYQSIHTVVLGEGEQPIEVQIRTRKMHDEAELGIAAHWKYKEGQTVSRS
DMC64         FDDYIAHPKPNGYQSIHTVVLGEGEQPIEVQIRTRKMHDEAELGIAAHWKYKEGQTVSRS
DMC111        FDDYIAHPKPNGYQSIHTVVLGEGEQPIEVQIRTRKMHDEAELGIAAHWKYKEGQTVSRS
NZV1          FDDYIAHPKPNGYQSIHTVVLGEGEQPIEVQIRTRKMHDEAELGIAAHWKYKEGPTVSRS
35000HP       FDDYIAHPKPNGYQSIHTVVLGEGEQPIEVQIRTRKMHDEAELGIAAHWKYKEGPTVSRS
NZS1          FDDYIAHPKPNGYQSIHTVVLGEGEQPIEVQIRTRKMHDEAELGIAAHWKYKEGPTVSRS
NZS2          FDDYIAHPKPNGYQSIHTVVLGEGEQPIEVQIRTRKMHDEAELGIAAHWKYKEGPTVSRS
NZS3          FDDYIAHPKPNGYQSIHTVVLGEGEQPIEVQIRTRKMHDEAELGIAAHWKYKEGPTVSRS
NZS4          FDDYIAHPKPNGYQSIHTVVLGEGEQPIEVQIRTRKMHDEAELGIAAHWKYKEGPTVSRS
82-029362     FDDYIAHPKPNGYQSIHTVVLGEGEQPIEVQIRTRKMHDEAELGIAAHWKYKEGPTVSRS
6644          FDDYIAHPKPNGYQSIHTVVLGEGEQPIEVQIRTRKMHDEAELGIAAHWKYKEGPTVSRS
HMC46         FDDYIAHPKPNGYQSIHTVVLGEGEQPIEVQIRTRKMHDEAELGIAAHWKYKEGPTVSRS
HMC56         FDDYIAHPKPNGYQSIHTVVLGEGEQPIEVQIRTRKMHDEAELGIAAHWKYKEGPTVSRS
              ****************************************************** *****

33921         GYEEKIIWLRKLLAWQNDLAASSDMMAEMRSQIFDDRVYVFTPRGEVIDLPKNSTPLDFA
CIP542        GYEEKIIWLRKLLAWQNDLAASSDMMAEMRSQIFDDRVYVFTPRGEVIDLPKNSTPLDFA
DMC64         GYEEKIIWLRKLLAWQNDLAASSDMMAEMRSQIFDDRVYVFTPRGEVIDLPKNSTPLDFA
DMC111        GYEEKIIWLRKLLAWQNDLAASSDMMAEMRSQIFDDRVYVFTPRGEVIDLPKNSTPLDFA
NZV1          GYEEKIIWLRKLLAWQNDLAVSSDMMADMRSQIFDDRVYVFTPRGEVIDLPKNSTPLDFA
35000HP       GYEEKIIWLRKLLAWQNDLAVSSDMMADMRSQIFDDRVYVFTPRGEVIDLPKNSTPLDFA
NZS1          GYEEKIIWLRKLLAWQNDLAVSSDMMADMRSQIFDDRVYVFTPRGEVIDLPKNSTPLDFA
NZS2          GYEEKIIWLRKLLAWQNDLAVSSDMMADMRSQIFDDRVYVFTPRGEVIDLPKNSTPLDFA
NZS3          GYEEKIIWLRKLLAWQNDLAVSSDMMADMRSQIFDDRVYVFTPRGEVIDLPKNSTPLDFA
NZS4          GYEEKIIWLRKLLAWQNDLAVSSDMMADMRSQIFDDRVYVFTPRGEVIDLPKNSTPLDFA
82-029362     GYEEKIIWLRKLLAWQNDLAVSSDMMADMRSQIFDDRVYVFTPRGEVIDLPKNSTPLDFA
6644          GYEEKIIWLRKLLAWQNDLAVSSDMMADMRSQIFDDRVYVFTPRGEVIDLPKNSTPLDFA
HMC46         GYEEKIIWLRKLLAWQNDLAVSSDMMADMRSQIFDDRVYVFTPRGEVIDLPKNSTPLDFA
HMC56         GYEEKIIWLRKLLAWQNDLAVSSDMMADMRSQIFDDRVYVFTPRGEVIDLPKNSTPLDFA
              ********************.******:********************************
```

```
33921          YAVHSEIGHRCIGAKIADRIVPFTYVLQMGDQVEIITQKTPNPSRDWLSPQAGFVNTSKA
CIP542         YAVHSEIGHRCIGAKIADRIVPFTYVLQMGDQVEIITQKTPNPSRDWLSPQAGFVNTSKA
DMC64          YAVHSEIGHRCIGAKIADRIVPFTYVLQMGDQVEIITQKTPNPSRDWLSPQAGFVNTSKA
DMC111         YAVHSEIGHRCIGAKIADRIVPFTYVLQMGDQVEIITQKTPNPSRDWLSPQAGFVNTSKA
NZV1           YAVHSEIGHRCIGAKIADRIVPFTYVLQMGDQVEIITQKTPNPSRDWLSPQAGFVNTSKA
35000HP        YAVHSEIGHRCIGAKIADRIVPFTYVLQMGDQVEIITQKTPNPSRDWLSPQAGFVNTSKA
NZS1           YAVHSEIGHRCIGAKIADRIVPFTYVLQMGDQVEIITQKTPNPSRDWLSPQAGFVNTSKA
NZS2           YAVHSEIGHRCIGAKIADRIVPFTYVLQMGDQVEIITQKTPNPSRDWLSPQAGFVNTSKA
NZS3           YAVHSEIGHRCIGAKIADRIVPFTYVLQMGDQVEIITQKTPNPSRDWLSPQAGFVNTSKA
NZS4           YAVHSEIGHRCIGAKIADRIVPFTYVLQMGDQVEIITQKTPNPSRDWLSPQAGFVNTSKA
82-029362      YAVHSEIGHRCIGAKIADRIVPFTYVLQMGDQVEIITQKTPNPSRDWLSPQAGFVNTSKA
6644           YAVHSEIGHRCIGAKIADRIVPFTYVLQMGDQVEIITQKTPNPSRDWLSPQAGFVNTSKA
HMC46          YAVHSEIGHRCIGAKIADRIVPFTYVLQMGDQVEIITQKTPNPSRDWLSPQAGFVNTSKA
HMC56          YAVHSEIGHRCIGAKIADRIVPFTYVLQMGDQVEIITQKTPNPSRDWLSPQAGFVNTSKA
               ************************************************************

33921          RAKIIAWFKKLDREKNIPIGKEALEAEISRLGLSHKQIEQYALPRYNLKQFDDLYAAIGG
CIP542         RAKIIAWFKKLDREKNIPIGKEALEAEISRLGLSHKQIEQYALPRYNLKQFDDLYAAIGG
DMC64          RAKIIAWFKKLDREKNIPIGKEALEAEISRLGLSHKQIEQYALPRYNLKQFDDLYAAIGG
DMC111         RAKIIAWFKKLDREKNIPIGKEALEAEISRLGLSHKQIEQYALPRYNLKQFDDLYAAIGG
NZV1           RAKIIAWFKKLDREKNIPLGKEALEAEISRLGLSHKQIEQYALPRYNLKQFDDLYAAIGG
35000HP        RAKIIAWFKKLDREKNIPLGKEALEAEISRLGLSHKQIEQYALPRYNLKQFDDLYAAIGG
NZS1           RAKIIAWFKKLDREKNIPLGKEALEAEISRLGLSHKQIEQYALPRYNLKQFDDLYAAIGG
NZS2           RAKIIAWFKKLDREKNIPLGKEALEAEISRLGLSHKQIEQYALPRYNLKQFDDLYAAIGG
NZS3           RAKIIAWFKKLDREKNIPLGKEALEAEISRLGLSHKQIEQYALPRYNLKQFDDLYAAIGG
NZS4           RAKIIAWFKKLDREKNIPLGKEALEAEISRLGLSHKQIEQYALPRYNLKQFDDLYAAIGG
82-029362      RAKIIAWFKKLDREKNIPLGKEALEAEISRLGLSHKQIEQYALPRYNLKQFDDLYAAIGG
6644           RAKIIAWFKKLDREKNIPLGKEALEAEISRLGLSHKQIEQYALPRYNLKQFDDLYAAIGG
HMC46          RAKIIAWFKKLDREKNIPLGKEALEAEISRLGLSHKQIEQYALPRYNLKQFDDLYAAIGG
HMC56          RAKIIAWFKKLDREKNIPLGKEALEAEISRLGLSHKQIEQYALPRYNLKQFDDLYAAIGG
               ******************:*****************************************

33921          GDIRLNQLSHYLQSKLMKPTAEQEDEAVLKHVNKNAYNAQQQKGKNGQIIIDSVGNLMHN
CIP542         GDIRLNQLSHYLQSKLMKPTAEQEDEAVLKHVNKNAYNAQQQKGKNGQIIIDSVGNLMHN
DMC64          GDIRLNQLSHYLQSKLMKPTAEQEDEAVLKHVNKNAYNAQQQKGKNGQIIIDGVGNLMHN
DMC111         GDIRLNQLSHYLQSKLMKPTAEQEDEAVLKHVNKNAYNAQQQKGKNGQIIIDGVGNLMHN
NZV1           GDIRLNQLSHYLQSKLIKPTAEQEDEAVLKHVNKNAYNAQQQKGKNGQIIIDGVGNLMHN
35000HP        GDIRLNQLSHYLQSKLIKPTAEQEDEAVLKHVNKNAYNAQQQKGKNGQIIIDGVGNLMHN
NZS1           GDIRLNQLSHYLQSKLIKPTAEQEDEAVLKHVNKNAYNAQQQKGKNGQIIIDGVGNLMHN
NZS2           GDIRLNQLSHYLQSKLIKPTAEQEDEAVLKHVNKNAYNAQQQKGKNGQIIIDGVGNLMHN
NZS3           GDIRLNQLSHYLQSKLIKPTAEQEDEAVLKHVNKNAYNAQQQKGKNGQIIIDGVGNLMHN
NZS4           GDIRLNQLSHYLQSKLIKPTAEQEDEAVLKHVNKNAYNAQQQKGKNGQIIIDGVGNLMHN
82-029362      GDIRLNQLSHYLQSKLIKPTAEQEDEAVLKHVNKNAYNAQQQKGKNGQIIIDGVGNLMHN
6644           GDIRLNQLSHYLQSKLIKPTAEQEDEAVLKHVNKNAYNAQQQKGKNGQIIIDGVGNLMHN
HMC46          GDIRLNQLSHYLQSKLIKPTAEQEDEAVLKHVNKNAYNAQQQKGKNGQIIIDGVGNLMHN
HMC56          GDIRLNQLSHYLQSKLIKPTAEQEDEAVLKHVNKNAYNAQQQKGKNGQIIIDGVGNLMHN
               ****************:***********************************.*******

33921          LACCCQPIPGDDIVGYITQGRGISIHCANCEQLFELRSANPERIVSAQWDSHFKAGFSLR
CIP542         LACCCQPIPGDDIVGYITQGRGISIHCANCEQLFELRSANPERIVSAQWDSHFKAGFSLR
DMC64          LACCCQPIPGDDIVGYITQGRGISIHCANCEQLFELRSANPERIVSAQWDSHFKAGFSLR
DMC111         LACCCQPIPGDDIVGYITQGRGISIHCANCEQLFELRSANPERIVSAQWDSHFKAGFSLR
NZV1           LARCCQPIPGDDIVGYITQGRGISIHCANCEQLFELRSANPERIVSAQWDSHFKAGFSLR
35000HP        LARCCQPIPGDDIVGYITQGRGISIHCANCEQLFELRSANPERIVSAQWDSHFKAGFSLR
NZS1           LARCCQPIPGDDIVGYITQGRGISIHCANCEQLFELRSANPERIVSAQWDSHFKAGFSLR
NZS2           LARCCQPIPGDDIVGYITQGRGISIHCANCEQLFELRSANPERIVSAQWDSHFKAGFSLR
NZS3           LARCCQPIPGDDIVGYITQGRGISIHCANCEQLFELRSANPERIVSAQWDSHFKAGFSLR
NZS4           LARCCQPIPGDDIVGYITQGRGISIHCANCEQLFELRSANPERIVSAQWDSHFKAGFSLR
82-029362      LARCCQPIPGDDIVGYITQGRGISIHCANCEQLFELRSANPERIVSAQWDSHFKAGFSLR
6644           LARCCQPIPGDDIVGYITQGRGISIHCANCEQLFELRSANPERIVSAQWDSHFKAGFSLR
HMC46          LARCCQPIPGDDIVGYITQGRGISIHCANCEQLFELRSANPERIVSAQWDSHFKAGFSLR
HMC56          LARCCQPIPGDDIVGYITQGRGISIHCANCEQLFELRSANPERIVSAQWDSHFKAGFSLR
               ** *********************************************************

33921          IRIIANECNGLLRDVSAVMANEKVNVLTVASRQDIKRGLTVMDIELEMSNVEM-------
CIP542         IRIIANECNGLLRDVSAVMANEKVNVLTVASRQDIKRGLTVMDIELEMSNVEM-------
DMC64          IRIIANECNGLLRDVSAVMANEKVNVLTVASRQDIKRGLTVMDIELEMSNVEM-------
DMC111         IRIIANECNGLLRDVSAVMANEKVNVLTVASRQDIKRGLTVMDIELEMSNVEM-------
NZV1           IRIIANERNGLLRDVSAVMANEKVNVLTVASRQDIKRGLTVMDIELEMSNVEMLAKILTR
35000HP        IRIIANERNGLLRDVSAVMANEKVNVLTVASRQDIKRGLTVMDIELEMSNVEML------
NZS1           IRIIANERNGLLRDVSAVMANEKVNVLTVASRQDIKRGLTVMDIELEMSNVEML------
NZS2           IRIIANERNGLLRDVSAVMANEKVNVLTVASRQDIKRGLTVMDIELEMSNVEML------
NZS3           IRIIANERNGLLRDVSAVMANEKVNVLTVASRQDIKRGLTVMDIELEMSNVEML------
```

```
NZS4          IRIIANERNGLLRDVSAVMANEKVNVLTVASRQDIKRGLTVMDIELEMSNVEML------
82-029362     IRIIANERNGLLRDVSAVMANEKVNVLTVASRQDIKRGLTVMDIELEMSNVEML------
6644          IRIIANERNGLLRDVSAVMANEKVNVLTVASRQDIKRGLTVMDIELEMSNVEML------
HMC46         IRIIANERNGLLRDVSAVMANEKVNVLTVASRQDIKRGLTVMDIELEMSNVEML------
HMC56         IRIIANERNGLLRDVSAVMANEKVNVLTVASRQDIKRGLTVMDIELEMSNVEML------
              ******* *********************************************

33921         -------------LAKILTRISQLESVIEAKRLAN*
CIP542        -------------LAKILTRISQLESVIEAKRLAN*
DMC64         -------------LAKILTRISQLESVIEAKRLAN*
DMC111        -------------LAKILTRISQLESVIEAKRLAN*
NZV1          VMDIELEMSNVEMLAKILTRISQLESVIEAKRLAN*
35000HP       --------------AKILTRISQLESVIEAKRLAN*
NZS1          --------------AKILTRISQLESVIEAKRLAN*
NZS2          --------------AKILTRISQLESVIEAKRLAN*
NZS3          --------------AKILTRISQLESVIEAKRLAN*
NZS4          --------------AKILTRISQLESVIEAKRLAN*
82-029362     --------------AKILTRISQLESVIEAKRLAN*
6644          --------------AKILTRISQLESVIEAKRLAN*
HMC46         --------------AKILTRISQLESVIEAKRLAN*
HMC56         --------------AKILTRISQLESVIEAKRLAN*
                            **********************
```

# 18) SapA (HD1230)

```
35000HP       MKILALSILKFSPFFAVFCWISTAYSAPRVPKELSADSLIYCTSISGLSFNPQKADVGTN
82-029362     MKILALSILKFSPFFAVFCWISTAYSAPRVPKELSADSLIYCTSISGLSFNPQKADVGTN
6644          MKILALSILKFSPFFAVFCWISTAYSAPRVPKELSADSLIYCTSISGLSFNPQKADVGTN
HMC46         MKILALSILKFSPFFAVFCWISTAYSAPRVPKELSADSLIYCTSISGLSFNPQKADVGTN
HMC56         MKILALSILKFSPFFAVFCWISTAYSAPRVPKELSADSLIYCTSISGLSFNPQKADVGTN
33921         MKILALSILKFSPFFAVFCWISTAYAAPRVPKELSADSLIYCTSISGLSFNPQKADVGTN
CIP542        MKILALSILKFSPFFAVFCWISTAYAAPRVPKELSADSLIYCTSISGLSFNPQKADVGTN
DMC64         MKILALSILKFSPFFAVFCWISTAYAAPRVPKELSADSLIYCTSISGLSFNPQKADVGTN
DMC111        MKILALSILKFSPFFAVFCWISTAYAAPRVPKELSADSLIYCTSISGLSFNPQKADVGTN
              *************************:**********************************

35000HP       MNVVTEQIYDKLFEIDRHTHRVIPSLAETFSVSDDGKEITLNLRRQVAFHKTPWFTPTRL
82-029362     MNVVTEQIYDKLFEIDRHTHRVIPSLAETFSVSDDGKEITLNLRRQVAFHKTPWFTPTRL
6644          MNVVTEQIYDKLFEIDRHTHRVIPSLAETFSVSDDGKEITLNLRRQVAFHKTPWFTPTRL
HMC46         MNVVTEQIYDKLFEIDRHTHRVIPSLAETFSVSDDGKEITLNLRRQVAFHKTPWFTPTRL
HMC56         MNVVTEQIYDKLFEIDRHTHRVIPSLAETFSVSDDGKEITLNLRRQVAFHKTPWFTPTRL
33921         MNVVTEQIYDKLFEIDRHTHRVIPSLAETFSVSDDGKEITLNLRRQVAFHKTPWFTPTRL
CIP542        MNVVTEQIYDKLFEIDRHTHRVIPSLAETFSVSDDGKEITLNLRRQVAFHKTPWFTPTRL
DMC64         MNVVTEQIYDKLFEIDRHTHRVIPSLAETFSVSDDGKEITLNLRRQVAFHKTPWFTPTRL
DMC111        MNVVTEQIYDKLFEIDRHTHRVIPSLAETFSVSDDGKEITLNLRRQVAFHKTPWFTPTRL
              ************************************************************

35000HP       FNAEDVVFSLNRMIGNVEELPALDFNEDSKEQFQQNQRYAYHFKANLAHYPYFESVALKK
82-029362     FNAEDVVFSLNHMIGNVEELPALDFNEDSKEQFQQNQRYAYHFKANLAHYPYFESVALKK
6644          FNAEDVVFSLNHMIGNVEELPALDFNEDSKEQFQQNQRYAYHFKANLAHYPYFESVALKK
HMC46         FNAEDVVFSLNHMIGNVEELPALDFNEDSKEQFQQNQRYAYHFKANLAHYPYFESVALKK
HMC56         FNAEDVVFSLNHMIGNVEELPALDFNEDSKEQFQQNQRYAYHFKANLAHYPYFESVALKK
33921         FNAEDVVFSLNRMIGNVEELPALDFNEDSKEQFQQNQRYAYHFKANLAHYPYFESVALKK
CIP542        FNAEDVVFSLNRMIGNVEELPALDFNEDSKEQFQQNQRYAYHFKANLAHYPYFESVALKK
DMC64         FNAEDVVFSLNRMIGNVEELPALDFNEDSKEQFQQNQRYAYHFKANLAHYPYFESVALKK
DMC111        FNAEDVVFSLNRMIGNVEELPALDFNEDSKEQFQQNQRYAYHFKANLAHYPYFESVALKK
              ***********:************************************************

35000HP       KIAKISAPNEYTVKIHLVAPDNSVLAHLASQYAVILSKEYALLLNADENLAQLDLLPVGT
82-029362     KIAKISAPNEYTVKIHLVAPDNSVLAHLASQYAVILSKEYALLLNADENLAQLDLLPVGT
6644          KIAKISAPNEYTVKIHLVAPDNSVLAHLASQYAVILSKEYALLLNADENLAQLDLLPVGT
HMC46         KIAKISAPNEYTVKIHLVAPDNSVLAHLASQYAVILSKEYALLLNADENLAQLDLLPVGT
HMC56         KIAKISAPNEYTVKIHLVAPDNSVLAHLASQYAVILSKEYALLLNADENLAQLDLLPVGT
33921         KIVKISAPNEYTVKIHLVAPDNSVLAHLASQYAVILSKEYALLLNADENLAQLDLLPVGT
CIP542        KIVKISAPNEYTVKIHLVAPDNSVLAHLASQYAVILSKEYALLLNADENLAQLDLLPVGT
DMC64         KIVKISAPNEYTVKIHLVAPDNSVLAHLASQYAVILSKEYALLLNADENLAQLDLLPVGT
DMC111        KIVKISAPNEYTVKIHLVAPDNSVLAHLASQYAVILSKEYALLLNADENLAQLDLLPVGT
              **.*********************************************************

35000HP       GVYQLSDYIQNEYVRLKPNPVYWGEKAKINNVVVDFSSNSTGRMAKYLNQECDIVAQPEP
82-029362     GVYQLSDYIQNEYVRLKPNPVYWGEKAKINNVVVDFSSNSTGRMAKYLNQECDIVAQPEP
```

```
6644           GVYQLSDYIQNEYVRLKPNPVYWGEKAKINNVVVDFSSNSTGRMAKYLNQECDIVAQPEP
HMC46          GVYQLSDYIQNEYVRLKPNPVYWGEKAKINNVVVDFSSNSTGRMAKYLNQECDIVAQPEP
HMC56          GVYQLSDYIQNEYVRLKPNPVYWGEKAKINNVVVDFSSNSTGRMAKYLNQECDIVAQPEP
33921          GVYQLSDYIQNEYVRLKPNPVYWGEKAKINNVVVDFSSNSTGRMAKYLNQECDIVAQPEP
CIP542         GVYQLSDYIQNEYVRLKPNPVYWGEKAKINNVVVDFSSNSTGRMAKYLNQECDIVAQPEP
DMC64          GVYQLSDYIQNEYVRLKPNPVYWGEKAKINNVVVDFSSNSTGRMAKYLNQECDIVAQPEP
DMC111         GVYQLSDYIQNEYVRLKPNPVYWGEKAKINNVVVDFSSNSTGRMAKYLNQECDIVAQPEP
               ************************************************************

35000HP        SQRRVISSYEIVESPGANLAFLAFNMQKEKMQDIAFRRQIAQAINRERLVKALFYGSAEV
82-029362      SQRRVISSYEIVESPGANLAFLAFNMQKEKMQDIAFRRQIAQAINRERLVKALFYGSAEV
6644           SQRRVISSYEIVESPGANLAFLAFNMQKEKMQDIAFRRQIAQAINRERLVKALFYGSAEV
HMC46          SQRRVISSYEIVESPGANLAFLAFNMQKEKMQDIAFRRQIAQAINRERLVKALFYGSAEV
HMC56          SQRRVISSYEIVESPGANLAFLAFNMQKEKMQDIAFRRQIAQAINRERLVKALFYGSAEV
33921          SQRRVISSYEIVESPGANLAFLAFNMQKEKMQDIAFRRQIAQAINRERLVKALFYGSAEV
CIP542         SQRRVISSYEIVESPGANLAFLAFNMQKEKMQDIAFRRQIAQAINRERLVKALFYGSAEV
DMC64          SQRRVISSYEIVESPGANLAFLAFNMQKEKMQDIAFRRQIAQAINRERLVKALFYGSAEV
DMC111         SQRRVISSYEIVESPGANLAFLAFNMQKEKMQDIAFRRQIAQAINRERLVKALFYGSAEV
               ************************************************************

35000HP        ADNVLPSALFAQKNPAAYPYKAPQPRAKNAKLDRLIFWVLDESRVYNLHPLKMAEMIRND
82-029362      ADNVLPSALFAQKNPAAYPYKAPQPRAKNAKLDRLIFWVLDESRVYNLHPLKMAEMIRND
6644           ADNVLPSALFAQKNPAAYPYKAPQPRAKNAKLDRLIFWVLDESRVYNLHPLKMAEMIRND
HMC46          ADNVLPSALFAQKNPAAYPYKAPQPRAKNAKLDRLIFWVLDESRVYNLHPLKMAEMIRND
HMC56          ADNVLPSALFAQKNPAAYPYKAPQPRAKNAKLDRLIFWVLDESRVYNLHPLKMAEMIRND
33921          ADNVLPSALFAQKNPAAYPYKAPQPRAKNAKLDRLIFWVLDESRVYNLHPLKMAEMIRND
CIP542         ADNVLPSALFAQKNPAAYPYKAPQPRAKNAKLDRLIFWVLDESRVYNLHPLKMAEMIRND
DMC64          ADNVLPSALFAQKNPAAYPYKAPQPRAKNAKLDRLIFWVLDESRVYNLHPLKMAEMIRND
DMC111         ADNVLPSALFAQKNPAAYPYKAPQPRAKNAKLDRLIFWVLDESRVYNLHPLKMAEMIRND
               ************************************************************

35000HP        LKKINIDVIIRPVSRAKVVQLAAAGKADYDLILTGWLANNLDPNAFLSPILSCRTQNKVT
82-029362      LKKINIDVIIRPVSRAKVVQLAAAGKADYDLILTGWLANNLDPNAFLSPILSCRTQNKVT
6644           LKKINIDVIIRPVSRAKVVQLAAAGKADYDLILTGWLANNLDPNAFLSPILSCRTQNKVT
HMC46          LKKINIDVIIRPVSRAKVVQLAAAGKADYDLILTGWLANNLDPNAFLSPILSCRTQNKVT
HMC56          LKKINIDVIIRPVSRAKVVQLAAAGKADYDLILTGWLANNLDPNAFLSPILSCRTQNKVT
33921          LKKINIDAIIRPVSRAKVVQLAAAGKADYDLILTGWLANNLDPNAFLSPILSCRTQNKVT
CIP542         LKKINIDAIIRPVSRAKVVQLAAAGKADYDLILTGWLANNLDPNAFLSPILSCRTQNKVT
DMC64          LKKINIDAIIRPVSRAKVVQLAAAGKADYDLILTGWLANNLDPNAFLSPILSCRTQNKVT
DMC111         LKKINIDAIIRPVSRAKVVQLAAAGKADYDLILTGWLANNLDPNAFLSPILSCRTQNKVT
               *******.****************************************************

35000HP        NLANWCHQQFDEWLEIAKANQVPYVRNMIYKQTQALLEEQLPILPLLHAQRSLFVNQKIK
82-029362      NLANWCHQQFDEWLEIAKANQVPYVRNMIYKQTQALLEEQLPILPLLHAQRSLFVNQKIK
6644           NLANWCHQQFDEWLEIAKANQVPYVRNMIYKQTQALLEEQLPILPLLHAQRSLFVNQKIK
HMC46          NLANWCHQQFDEWLEIAKANQVPYVRNMIYKQTQALLEEQLPILPLLHAQRSLFVNQKIK
HMC56          NLANWCHQQFDEWLEIAKANQVPYVRNMIYKQTQALLEEQLPILPLLHAQRSLFVNQKIK
33921          NLANWCHQQFDEWLEIAKANQVPYVRNMIDKQTQALLEEQLPILPLLHAQRSLFVNQKIK
CIP542         NLANWCHQQFDEWLEIAKANQVPYVRNMIDKQTQALLEEQLPILPLLHAQRSLFVNQKIK
DMC64          NLANWCHQQFDEWLEIAKANQVPYVRNMIDKQTQALLEEQLPILPLLHAQRSLFVNQKIK
DMC111         NLANWCHQQFDEWLEIAKANQVPYVRNMIDKQTQALLEEQLPILPLLHAQRSLFVNQKIK
               ***************************** ******************************

35000HP        NAHIEPFGQVRLSELTLHQE*
82-029362      NAHIEPFGQVRLSELTLHQE*
6644           NAHIEPFGQVRLSELTLHQE*
HMC46          NAHIEPFGQVRLSELTLHQE*
HMC56          NAHIEPFGQVRLSELTLHQE*
33921          NAHIEPFGQVRLSELTLHQE*
CIP542         NAHIEPFGQVRLSELTLHQE*
DMC64          NAHIEPFGQVRLSELTLHQE*
DMC111         NAHIEPFGQVRLSELTLHQE*
               *********************
```

**Note:** The SapA sequence of NZS1, NZS2, NZS3, NZS4, and NZV1 were identical to that of 35000HP.

# 19) SapB (HD1231)

```
35000HP      MLFAFIRRLFLSLITLIILTLIGYNILLRDPLNHFMDLYGIQAYFSYVMGLLHGDFGISY
33921        MLFAFIRRLFLSLITLIILTLIGYNILLRDPLNHFMDLYGIQAYFSYVMGLLHGDFGISY
CIP542       MLFAFIRRLFLSLITLIILTLIGYNILLRDPLNHFMDLYGIQAYFSYVMGLLHGDFGISY
```

```
DMC64        MLFAFIRRLFLSLITLIILTLIGYNILLRDPLNHFMDLYGIQAYFSYVMGLLHGDFGISY
DMC111       MLFAFIRRLFLSLITLIILTLIGYNILLRDPLNHFMDLYGIQAYFSYVMGLLHGDFGISY
             ************************************************************

35000HP      SNGDPIANQILNVFPATISLCFAALFVSVIIGIPLGFVAASFRYNVVGKLLAIVSAFSLA
33921        SNGDPIANQILNVFPATISLCFAALFVSVIIGIPLGFVAASFRDNVVGKLLAIVSAFSLA
CIP542       SNGDPIANQILNVFPATISLCFAALFVSVIIGIPLGFVAASFRDNVVGKLLAIVSAFSLA
DMC64        SNGDPIANQILNVFPATISLCFAALFVSVIIGIPLGFVAASFRDNVVGKLLAIVSAFSLA
DMC111       SNGDPIANQILNVFPATISLCFAALFVSVIIGIPLGFVAASFRDNVVGKLLAIVSAFSLA
             ******************************************* ****************

35000HP      IPVFWLAIMALYYAASNDWQIAAVGELHPIYEISLVTGFRLLDIFLADSPYKLKMMQSVL
33921        IPVFWLAIMALYYAASNDWQIAAVGELHPIYEISLVTGFRLLDIFLADSPYKLKMMQSVL
CIP542       IPVFWLAIMALYYAASNDWQIAAVGELHPIYEISLVTGFRLLDIFLADSPYKLKMMQSVL
DMC64        IPVFWLAIMALYYAASNDWQIAAVGELHPIYEISLVTGFRLLDIFLADSPYKLKMMQSVL
DMC111       IPVFWLAIMALYYAASNDWQIAAVGELHPIYEISLVTGFRLLDIFLADSPYKLKMMQSVL
             ************************************************************

35000HP      HHLALPTLILALPATLEVIRFTRQRAEYVMKQNYIKVARTRGWSPYKIWLKHILRNTLPA
33921        HHLALPTLILALPATLEVIRFTRQRAEYVMKQNYIKVARTRGWSPYKIWLKHILRNTLPA
CIP542       HHLALPTLILALPATLEVIRFTRQRAEYVMKQNYIKVARTRGWSPYKIWLKHILRNTLPA
DMC64        HHLALPTLILALPATLEVIRFTRQRAEYVMKQNYIKVARTRGWSPYKIWLKHILRNTLPA
DMC111       HHLALPTLILALPATLEVIRFTRQRAEYVMKQNYIKVARTRGWSPYKIWLKHILRNTLPA
             ************************************************************

35000HP      LIPMIARNLTLVFAFAMLVENIFSWGGIGLWLINALAIQDYNAISAGVVAIGLFVLGVDI
33921        LIPMIARNLTLVFAFAMLVENIFSWGGIGLWLINALAIQDYNAISAGVVAIDLFVLGVDI
CIP542       LIPMIARNLTLVFAFAMLVENIFSWGGIGLWLINALAIQDYNAISAGVVAIDLFVLGVDI
DMC64        LIPMIARNLTLVFAFAMLVENIFSWGGIGLWLINALAIQDYNAISAGVVAIDLFVLGVDI
DMC111       LIPMIARNLTLVFAFAMLVENIFSWGGIGLWLINALAIQDYNAISAGVVAIDLFVLGVDI
             *************************************************** ********

35000HP      LVRLVTTLLDPSQKKDWYVK*
33921        LVRLVTTLLDPSQKKDWYVK*
CIP542       LVRLVTTLLDPSQKKDWYVK*
DMC64        LVRLVTTLLDPSQKKDWYVK*
DMC111       LVRLVTTLLDPSQKKDWYVK*
             *********************
```

**Note:** The SapB sequence of NZS1, NZS2, NZS3, NZS4, NZV1, 82-029362, 6644, HD183, HMC46, and HMC56 were identical to that of 35000HP.

# 20) SapC (HD1232)

```
35000HP        MLNREEPEQFRQSDYAKQFWLELRQDKVALASLYFFLILLFLTFAGELIAPYQVNTQFVG
NZS1           MLNREEPEQFRQSDYAKQFWLELRQDKVALASLYFFLILLFLTFAGELIAPYQVNTQFVG
NZS2           MLNREEPEQFRQSDYAKQFWLELRQDKVALASLYFFLILLFLTFAGELIAPYQVNTQFVG
NZS3           MLNREEPEQFRQSDYAKQFWLELRQDKVALASLYFFLILLFLTFAGELIAPYQVNTQFVG
NZS4           MLNREEPEQFRQSDYAKQFWLELRQDKVALASLYFFLILLFLTFAGELIAPYQVNTQFVG
NZV1           MLNREEPEQFRQSDYAKQFWLELRQDKVALASLYFFLILLFLTFAGELIAPYQVNTQFVG
82-029362      MLNREEPEQFRQSDYAKQFWLELRQDKVALASLYFFLILLFLTFAGELIAPYQVNTQFVG
6644           MLNREEPEQFRQSDYAKQFWLELRQDKVALASLYFFLILLFLTFAGELIAPYQVNTQFVG
HD183          MLNREEPEQFRQSDYAKQFWLELRQDKVALASLYFFLILLFLTFAGELIAPYQVNTQFVG
HMC46          MLNREEPEQFRQSDYAKQFWLELRQDKVALASLYFFLILLFLTFAGELIAPYQVNTQFVG
HMC56          MLNREEPEQFRQSDYAKQFWLELRQDKVALASLYFFLILLFLTFAGELIAPYQVNTQFVG
33921          MLNREEPEQFRQSDYAKQFWLELRQDKVALASLYFFLILLFLTFAGELIAPYQVNTQFVG
CIP542         MLNREEPEQFRQSDYAKQFWLELRQDKVALASLYFFLILLFLTFAGELIAPYQVNTQFVG
DMC64          MLNREEPEQFRQSDYAKQFWLELRQDKVALASLYFFLILLFLTFAGELIAPYQVNTQFVG
DMC111         MLNREEPEQFRQSDYAKQFWLELRQDKVALASLYFFLILLFLTFAGELIAPYQVNTQFVG
               ************************************************************

35000HP        FELLPPSWDDYGQISHFFGTDDLGRDIFSRILAGFYYTVGAALLISFAIAIIGGVIGVLA
NZS1           FELLPPSWDDYGQISHFFGTDDLGRDIFSRILAGFYYTVGAALLISFAIAIIGGVIGVLA
NZS2           FELLPPSWDDYGQISHFFGTDDLGRDIFSRILAGFYYTVGAALLISFAIAIIGGVIGVLA
NZS3           FELLPPSWDDYGQISHFFGTDDLGRDIFSRILAGFYYTVGAALLISFAIAIIGGVIGVLA
NZS4           FELLPPSWDDYGQISHFFGTDDLGRDIFSRILAGFYYTVGAALLISFAIAIIGGVIGVLA
NZV1           FELLPPSWDDYGQISHFFGTDDLGRDIFSRILAGFYYTVGAALLISFAIAIIGGVIGVLA
82-029362      FELLPPSWDDYGQISHFFGTDDLGRDIFSRILAGFYYTVGAALLISFAIAIIGGVIGVLA
6644           FELLPPSWDDYGQISHFFGTDDLGRDIFSRILAGFYYTVGAALLISFAIAIIGGVIGVLA
HD183          FELLPPSWDDYGQISHFFGTDDLGRDIFSRILAGFYYTVGAALLISFAIAIIGGVIGVLA
HMC46          FELLPPSWDDYGQISHFFGTDDLGRDIFSRILAGFYYTVGAALLISFAIAIIGGVIGVLA
HMC56          FELLPPSWDDYGQISHFFGTDDLGRDIFSRILAGFYYTVGAALLISFAIAIIGGVIGVLA
```

```
33921          FELLPPSWGDYGQISYFFGTDDLGRDIFSRILAGFYYTVGAALLISFAIAIIGGVIGVLA
CIP542         FELLPPSWGDYGQISYFFGTDDLGRDIFSRILAGFYYTVGAALLISFAIAIIGGVIGVLA
DMC64          FELLPPSWGDYGQISYFFGTDDLGRDIFSRILAGFYYTVGAALLISFAIAIIGGVIGVLA
DMC111         FELLPPSWGDYGQISYFFGTDDLGRDIFSRILAGFYYTVGAALLISFAIAIIGGVIGVLA
               ******** ******:*********************************************

35000HP        GTSRKAISFLGHLFDTFLFIPTLIIAIIIATLMEASLINAMLAIFLAMLPHFIHKIYQAT
NZS1           GTSRKAISFLGHLFDTFLFIPTLIIAIIIATLMEASLINAMLAIFLAMLPHFIHKIYQAT
NZS2           GTSRKAISFLGHLFDTFLFIPTLIIAIIIATLMEASLINAMLAIFLAMLPHFIHKIYQAT
NZS3           GTSRKAISFLGHLFDTFLFIPTLIIAIIIATLMEASLINAMLAIFLAMLPHFIHKIYQAT
NZS4           GTSRKAISFLGHLFDTFLFIPTLIIAIIIATLMEASLINAMLAIFLAMLPHFIHKIYQAT
NZV1           GTSRKAISFLGHLFDTFLFIPTLIIAIIIATLMEASLINAMLAIFLAMLPHFIHKIYQAT
82-029362      GTSRKAISFLGHLFDTFLFIPTLIIAIIIATLMEASLINAMLAIFLAMLPHFIHKIYQAT
6644           GTSRKAISFLGHLFDTFLFIPTLIIAIIIATLMEASLINAMLAIFLAMLPHFIHKIYQAT
HD183          GTSRKAISFLGHLFDTFLFIPTLIIAIIIATLMEASLINAMLAIFLAMLPHFIHKIYQAT
HMC46          GTSRKAISFLGHLFDTFLFIPTLIIAIIIATLMEASLINAMLAIFLAMLPHFIHKIYQAT
HMC56          GTSRKAISFLGHLFDTFLFIPTLIIAIIIATLMEASLINAMLAIFLAMLPHFIHKIYQAT
33921          GTSRKAISFLGHLFDTFLFIPTLIIAIIIATLMEASLINAMLAIFLAMLPHFIHKIYQAT
CIP542         GTSRKAISFLGHLFDTFLFIPTLIIAIIIATLMEASLINAMLAIFLAMLPHFIHKIYQAT
DMC64          GTSRKAISFLGHLFDTFLFIPTLIIAIIIATLMEASLINAMLAIFLAMLPHFIHKIYQAT
DMC111         GTSRKAISFLGHLFDTFLFIPTLIIAIIIATLMEASLINAMLAIFLAMLPHFIHKIYQAT
               ************************************************************

35000HP        EQQLKREYVITLRLDGISRWQLIKEVVLPNLTALAVKEMTHICIIAVLDINALSFIGLGA
NZS1           EQQLKREYVITLRLDGISRWQLIKEVVLPNLTALAVKEMTHICIIAVLDINALSFIGLGA
NZS2           EQQLKREYVITLRLDGISRWQLIKEVVLPNLTALAVKEMTHICIIAVLDINALSFIGLGA
NZS3           EQQLKREYVITLRLDGISRWQLIKEVVLPNLTALAVKEMTHICIIAVLDINALSFIGLGA
NZS4           EQQLKREYVITLRLDGISRWQLIKEVVLPNLTALAVKEMTHICIIAVLDINALSFIGLGA
NZV1           EQQLKREYVITLRLDGISRWQLIKEVVLPNLTALAVKEMTHICIIAVLDINALSFIGLGA
82-029362      EQQLKREYVITLRLDGISRWQLIKEVVLPNLTALAVKEMTHICIIAVLDINALSFIGLGA
6644           EQQLKREYVITLRLDGISRWQLIKEVVLPNLTALAVKEMTHICIIAVLDINALSFIGLGA
HD183          EQQLKREYVITLRLDGISRWQLIKEVVLPNLTALAVKEMTHICIIAVLDINALSFIGLGA
HMC46          EQQLKREYVITLRLDGISRWQLIKEVVLPNLTALAVKEMTHICIIAVLDINALSFIGLGA
HMC56          EQQLKREYVITLRLDGISRWQLIKEVVLPNLTALAVKEMTHICIIAVLDINALSFIGLGA
33921          EQQLKREYVITLRLDGISRWQLIKEVVLPNLTALAVKEMTHICIIAVLDINALSFIGLGA
CIP542         EQQLKREYVITLRLDGISRWQLIKEVVLPNLTALAVKEMTHICIIAVLDINALSFIGLGA
DMC64          EQQLKREYVITLRLDGISRWQLIKEVVLPNLTALAVKEMTHICIIAVLDINALSFIGLGA
DMC111         EQQLKREYVITLRLDGISRWQLIKEVVLPNLTALAVKEMTHICIIAVLDINALSFIGLGA
               ************************************************************

35000HP        QSPMPEWGVMIKDSIELIYIAPWTVILPGIITILVILIISMLGNSISRVLEKHRY*
NZS1           QSPMPEWGVMIKDSIELIYIAPWTVILPGIITILVILIISMLGNSISRVLEKHRY*
NZS2           QSPMPEWGVMIKDSIELIYIAPWTVILPGIITILVILIISMLGNSISRVLEKHRY*
NZS3           QSPMPEWGVMIKDSIELIYIAPWTVILPGIITILVILIISMLGNSISRVLEKHRY*
NZS4           QSPMPEWGVMIKDSIELIYIAPWTVILPGIITILVILIISMLGNSISRVLEKHRY*
NZV1           QSPMPEWGVMIKDSIELIYIAPWTVILPGIITILVILIISMLGNSISRVLEKHRY*
82-029362      QSPMPEWGVMIKDSIELIYIAPWTVILPGIITILVILIISMLGNSISRVLEKHRY*
6644           QSPMPEWGVMIKDSIELIYIAPWTVILPGIITILVILIISMLGNSISRVLEKHRY*
HD183          QSPMPEWGVMIKDSIELIYIAPWTVILPGIITILVILIISMLGNSISRVLEKHRY*
HMC46          QSPMPEWGVMIKDSIELIYIAPWTVILPGIITILVILIISMLGNSISRVLEKHRY*
HMC56          QSPMPEWGVMIKDSIELIYIAPWTVILPGIITILVILIISMLGNSISRVLEKHRY*
33921          QSPMPEWGVMIKDSIELIYIAPWTVILPGIITILVILIISMLGNSISRVLEKHRY*
CIP542         QSPMPEWGVMIKDSIELIYIAPWTVILPGIITILVILIISMLGNSISRVLEKHRY*
DMC64          QSPMPEWGVMIKDSIELIYIAPWTVILPGIITILVILIISMLGNSISRVLEKHRY*
DMC111         QSPMPEWGVMIKDSIELIYIAPWTVILPGIITILVILIISMLGNSISRVLEKHRY*
               ********************************************************
```

# 21) SpoT (HD1924)

```
35000HP      LHLFEPLHSIIQTYLPTDKIEWIQRAFVVARDAHEGQTRSSGEPYITHPVAVATIIAEMK
33921        LHLFEPLHSIIQTYLPTDKIEWIQRAFVVARDAHEGQTRSSGEPYITHPVAVATIIAEMK
CIP542       LHLFEPLHSIIQTYLPTDKIEWIQRAFVVARDAHEGQTRSSGEPYITHPVAVATIIAEMK
DMC64        LHLFEPLHSIIQTYLPTDKIEWIQRAFVVARDAHEGQTRSSGEPYITHPVAVATIIAEMK
DMC111       LHLFEPLHSIIQTYLPTDKIEWIQRAFVVARDAHEGQTRSSGEPYITHPVAVATIIAEMK
             ************************************************************

35000HP      LDHEAIMAALLHDVIEDTPYTEEELAAEFGSNVAKIVQGVSKLDKLKFRTRQEAQVENFR
33921        LDHEAIMAALLHDVIEDTPYTEEELAAEFGSNVAKIVQGVSKLDKLKFRTRQEAQVENFR
CIP542       LDHEAIMAALLHDVIEDTPYTEEELAAEFGSNVAKIVQGVSKLDKLKFRTRQEAQVENFR
DMC64        LDHEAIMAALLHDVIEDTPYTEEELAAEFGSNVAKIVQGVSKLDKLKFRTRQEAQVENFR
DMC111       LDHEAIMAALLHDVIEDTPYTEEELAAEFGSNVAKIVQGVSKLDKLKFRTRQEAQVENFR
```

```
            ************************************************************

35000HP     KMILAMTKDIRVVLIKLADRTHNMRTLGSLRPDKRRRIAKETLEIYSPIAHRLGIEHLKN
33921       KMILAMTKDIRVVLIKLADRTHNMRTLGSLRPDKRRRIAKETLEIYSPLAHRLGIEHLKN
CIP542      KMILAMTKDIRVVLIKLADRTHNMRTLGSLRPDKRRRIAKETLEIYSPLAHRLGIEHLKN
DMC64       KMILAMTKDIRVVLIKLADRTHNMRTLGSLRPDKRRRIAKETLEIYSPLAHRLGIEHLKN
DMC111      KMILAMTKDIRVVLIKLADRTHNMRTLGSLRPDKRRRIAKETLEIYSPLAHRLGIEHLKN
            ************************************************:***********

35000HP     ELENLCFQAMHPYRYRILRLAIDMARGTRQDLLSTISHEIQMRLDEMGIKGRVYGREKHL
33921       ELENLCFQAMHPYRYRILRLAIDMARGTRQDLLSTISHEIQMRLDEMGIKGRVYGREKHL
CIP542      ELENLCFQAMHPYRYRILRLAIDMARGTRQDLLSTISHEIQMRLDEMGIKGRVYGREKHL
DMC64       ELENLCFQAMHPYRYRILRLAIDMARGTRQDLLSTISHEIQMRLDEMGIKGRVYGREKHL
DMC111      ELENLCFQAMHPYRYRILRLAIDMARGTRQDLLSTISHEIQMRLDEMGIKGRVYGREKHL
            ************************************************************

35000HP     YALYEKMRQRDQHFHSILDIYAFRIVVNSIDNCYRVLGQMHALYKPRPYQIRDYIAVPKS
33921       YALYEKMRQRDQHFHSILDIYAFRIVVNSIDNCYRVLGQMHALYKPRPYQIRDYIAVPKS
CIP542      YALYEKMRQRDQHFHSILDIYAFRIVVNSIDNCYRVLGQMHALYKPRPYQIRDYIAVPKS
DMC64       YALYEKMRQRDQHFHSILDIYAFRIVVNSIDNCYRVLGQMHALYKPRPYQIRDYIAVPKS
DMC111      YALYEKMRQRDQHFHSILDIYAFRIVVNSIDNCYRVLGQMHALYKPRPYQIRDYIAVPKS
            ************************************************************

35000HP     NGYQSLHTSMIGHKGMPIEVQIRTEDMDLMAELGVAAHWRYSEDQTQTTSVQQKAQQWLR
33921       NGYQSLHTSMIGHKGMPIEVQIRTEDMDLMAELGVAAHWRYSEDQTQTTSVQQKAQQWLR
CIP542      NGYQSLHTSMIGHKGMPIEVQIRTEDMDLMAELGVAAHWRYSEDQTQTTSVQQKAQQWLR
DMC64       NGYQSLHTSMIGHKGMPIEVQIRTEDMDLMAELGVAAHWRYSEDQTQTTSVQQKAQQWLR
DMC111      NGYQSLHTSMIGHKGMPIEVQIRTEDMDLMAELGVAAHWRYSEDQTQTTSVQQKAQQWLR
            ************************************************************

35000HP     SIVELQQSAGNSDEFIENVKSDLFSDDIYVFTPKGRIVELPAKATAIDFAYAVHSDIGDC
33921       SIVELQQSAGNSDEFIENVKSDLFSDDIYVFTPKGRIVELPAKATAIDFAYAVHSDIGDC
CIP542      SIVELQQSAGNSDEFIENVKSDLFSDDIYVFTPKGRIVELPAKATAIDFAYAVHSDIGDC
DMC64       SIVELQQSAGNSDEFIENVKSDLFSDDIYVFTPKGRIVELPAKATAIDFAYAVHSDIGDC
DMC111      SIVELQQSAGNSDEFIENVKSDLFSDDIYVFTPKGRIVELPAKATAIDFAYAVHSDIGDC
            ************************************************************

35000HP     CIGATVDRKPYPISQPLESGQTVEIITKAGKHPKRAWLNFVVTAKARSKIKAALKQFELI
33921       CIGATVDRKPYPISQPLESGQTVEIITKAGKHPKRAWLNFVVTAKARSKIKAALKQFELI
CIP542      CIGATVDRKPYPISQPLESGQTVEIITKAGKHPKRAWLNFVVTAKARSKIKAALKQFELI
DMC64       CIGATVDRKPYPISQPLESGQTVEIITKAGKHPKRAWLNFVVTAKARSKIKAALKQFELI
DMC111      CIGATVDRKPYPISQPLESGQTVEIITKAGKHPKRAWLNFVVTAKARSKIKAALKQFELI
            ************************************************************

35000HP     AEKPEETEKVEQLDIELEIKDQIGVLANITNTIASMNSNIGMIESRPNDKGNYQVKIRIS
33921       AEKPEETEKVEQLDIELEIKDQTGVLANITNTIASMNSNIGMIESRPNDKGNYQVKMRIS
CIP542      AEKPEETEKVEQLDIELEIKDQTGVLANITNTIASMNSNIGMIESRPNDKGNYQVKMRIS
DMC64       AEKPEETEKVEQLDIELEIKDQTGVLANITNTIASMNSNIGMIESRPNDKGNYQVKMRIS
DMC111      AEKPEETEKVEQLDIELEIKDQTGVLANITNTIASMNSNIGMIESRPNDKGNYQVKMRIS
            ********************** *********************************:***

35000HP     VTDKTHLYVVIQKLIRVRGVIRVAKVSA*
33921       VTDKTHLYVVIQKLIRVRGVIRVAKVSA*
CIP542      VTDKTHLYVVIQKLIRVRGVIRVAKVSA*
DMC64       VTDKTHLYVVIQKLIRVRGVIRVAKVSA*
DMC111      VTDKTHLYVVIQKLIRVRGVIRVAKVSA*
            *****************************
```

**Note:** The SpoT sequence of NZS1, NZS2, NZS3, NZS4, NZV1, 82-029362, 6644, HD183, HMC46, and HMC56 were identical to that of 35000HP.

# 22) TadA (HD1304)

```
35000HP       MLTKDQQVFFRNALLSNLNVDTLDEIENERSKLVTELTQSLYRVANTNNIYITPYDATDM
82-029362     MLTKDQQVFFRNALLSNLNVDTLDEIENERSKLVTELTQSLYRVANTNNIYITPYDATDM
6644          MLTKDQQVFFRNALLSNLNVDTLDEIENERSKLVTELTQSLYRVANTNNIYITPYDATDM
HD183         MLTKDQQVFFRNALLSNLNVDTLDEIENERSKLVTELTQSLYRVANTNNIYITPYDATDM
HMC46         MLTKDQQVFFRNALLSNLNVDTLDEIENERSKLVTELTQSLYRVANTNNIYITPYDATDM
HMC56         MLTKDQQVFFRNALLSNLNVDTLDEIENERSKLVTELTQSLYRVANTNNIYITPYDATDM
33921         MLTKDQQVFFRNALLSNLNVDTLDEIENERSKLVTELTQSLYRVANTNNIYITPYDATDM
CIP542        MLTKDQQVFFRNALLSNLNVDTLDEIENERSKLVTELTQSLYRVANTNNIYITPYDATDM
DMC64         MLTKDQQVFFRNALLSNLNVDTLDEIENERSKLVTELTQSLYRVANTNNIYITPYDATDM
```

```
DMC111          MLTKDQQVFFRNALLSNLNVDTLDEIENERSKLVTELTQSLYRVANTNNIYITPYDATDM
                ************************************************************

35000HP         AEIVADEIGGYGPIRELMEDDTVNDILVNGPDNIWIERAGVLEKTNKTFINNEQLTDIAK
82-029362       AEIVADEIGGYGPIRELMEDDTVNDILVNGPDNIWIERAGVLEKTNKTFINNEQLTDIAK
6644            AEIVADEIGGYGPIRELMEDDTVNDILVNGPDNIWIERAGVLEKTNKTFINNEQLTDIAK
HD183           AEIVADEIGGYGPIRELMEDDTVNDILVNGPDNIWIERAGVLEKTNKTFINNEQLTDIAK
HMC46           AEIVADEIGGYGPIRELMEDDTVNDILVNGPDNIWIERAGVLEKTNKTFINNEQLTDIAK
HMC56           AEIVADEIGGYGPIRELMEDDTVNDILVNGPDNIWIERAGVLEKTNKTFINNEQLTDIAK
33921           AEIVADEIGGYGPIRELMEDDTVNDILVNGPDNIWVERAGVLEKTNKTFINNEQLTDIAK
CIP542          AEIVADEIGGYGPIRELMEDDTVNDILVNGPDNIWVERAGVLEKTNKTFINNEQLTDIAK
DMC64           AEIVADEIGGYGPIRELMEDDTVNDILVNGPDNIWVERAGVLEKTNKTFINNEQLTDIAK
DMC111          AEIVADEIGGYGPIRELMEDDTVNDILVNGPDNIWVERAGVLEKTNKTFINNEQLTDIAK
                ***********************************:************************

35000HP         RLVARVGRRIDEGMPLVDSRLPDGSRLNVVIQPIALDGTSISIRKFSKSKKSLQELVNFG
82-029362       RLVARVGRRIDEGMPLVDSRLPDGSRLNVVIQPIALDGTSISIRKFSKSKKSLQELVNFG
6644            RLVARVGRRIDEGMPLVDSRLPDGSRLNVVIQPIALDGTSISIRKFSKSKKSLQELVNFG
HD183           RLVARVGRRIDEGMPLVDSRLPDGSRLNVVIQPIALDGTSISIRKFSKSKKSLQELVNFG
HMC46           RLVARVGRRIDEGMPLVDSRLPDGSRLNVVIQPIALDGTSISIRKFSKSKKSLQELVNFG
HMC56           RLVARVGRRIDEGMPLVDSRLPDGSRLNVVIQPIALDGTSISIRKFSKSKKSLQELVNFG
33921           RLVARVGRRIDESMPLVDSRLPDGSRLNVVIQPIALDGTSISIRKFSKSKKSLQELVNFG
CIP542          RLVARVGRRIDESMPLVDSRLPDGSRLNVVIQPIALDGTSISIRKFSKSKKSLQELVNFG
DMC64           RLVARVGRRIDESMPLVDSRLPDGSRLNVVIQPIALDGTSISIRKFSKSKKSLQELVNFG
DMC111          RLVARVGRRIDESMPLVDSRLPDGSRLNVVIQPIALDGTSISIRKFSKSKKSLQELVNFG
                ************.***********************************************

35000HP         SMTLDMANFLIIAARSRVNIIVSGGTGSGKTTLLNALSSYISPTERVLTLEDTAELRLEQ
82-029362       SMTLDMANFLIIAARSRVNIIVSGGTGSGKTTLLNALSSYISPTERVLTLEDTAELRLEQ
6644            SMTLDMANFLIIAARSRVNIIVSGGTGSGKTTLLNALSSYISPTERVLTLEDTAELRLEQ
HD183           SMTLDMANFLIIAARSRVNIIVSGGTGSGKTTLLNALSSYISPTERVLTLEDTAELRLEQ
HMC46           SMTLDMANFLIIAARSRVNIIVSGGTGSGKTTLLNALSSYISPTERVLTLEDTAELRLEQ
HMC56           SMTLDMANFLIIAARSRVNIIVSGGTGSGKTTLLNALSSYISPTERVLTLEDTAELRLEQ
33921           SMTLDMANFLIIAARSRVNIIVSGGTGSGKTTLLNALSSYISPTERVLTLEDTAELRLEQ
CIP542          SMTLDMANFLIIAARSRVNIIVSGGTGSGKTTLLNALSSYISPTERVLTLEDTAELRLEQ
DMC64           SMTLDMANFLIIAARSRVNIIVSGGTGSGKTTLLNALSSYISPTERVLTLEDTAELRLEQ
DMC111          SMTLDMANFLIIAARSRVNIIVSGGTGSGKTTLLNALSSYISPTERVLTLEDTAELRLEQ
                ************************************************************

35000HP         PHVVRLETRLAGVERTGEITMQDLVINALRMRPERIIVGECRGAEAFQMLQAMNTGHDGS
82-029362       PHVVRLETRLAGVERTGEITMQDLVINALRMRPERIIVGECRGAEAFQMLQAMNTGHDGS
6644            PHVVRLETRLAGVERTGEITMQDLVINALRMRPERIIVGECRGAEAFQMLQAMNTGHDGS
HD183           PHVVRLETRLAGVERTGEITMQDLVINALRMRPERIIVGECRGAEAFQMLQAMNTGHDGS
HMC46           PHVVRLETRLAGVERTGEITMQDLVINALRMRPERIIVGECRGAEAFQMLQAMNTGHDGS
HMC56           PHVVRLETRLAGVERTGEITMQDLVINALRMRPERIIVGECRGAEAFQMLQAMNTGHDGS
33921           PHVVRLETRLAGVERTGEITMQDLVINALRMRPERIIVGECRGAEAFQMLQAMNTGHDGS
CIP542          PHVVRLETRLAGVERTGEITMQDLVINALRMRPERIIVGECRGAEAFQMLQAMNTGHDGS
DMC64           PHVVRLETRLAGVERTGEITMQDLVINALRMRPERIIVGECRGAEAFQMLQAMNTGHDGS
DMC111          PHVVRLETRLAGVERTGEITMQDLVINALRMRPERIIVGECRGAEAFQMLQAMNTGHDGS
                ************************************************************

35000HP         MSTLHANTPRDATARLESMVMMSNASLPLEAIRRNIASAVNIIIQASRLNDGSRKVMNIT
82-029362       MSTLHANTPRDATARLESMVMMSNASLPLEAIRRNIASAVNIIIQASRLNDGSRKVMNIT
6644            MSTLHANTPRDATARLESMVMMSNASLPLEAIRRNIASAVNIIIQASRLNDGSRKVMNIT
HD183           MSTLHANTPRDATARLESMVMMSNASLPLEAIRRNIASAVNIIIQASRLNDGSRKVMNIT
HMC46           MSTLHANTPRDATARLESMVMMSNASLPLEAIRRNIASAVNIIIQASRLNDGSRKVMNIT
HMC56           MSTLHANTPRDATARLESMVMMSNASLPLEAIRRNIASAVNIIIQASRLNDGSRKVMNIT
33921           MSTLHANTPRDATARLESMVMMSNASLPLEAIRRNIASAVNIIIQASRLNDGSRKVMNIT
CIP542          MSTLHANTPRDATARLESMVMMSNASLPLEAIRRNIASAVNIIIQASRLNDGSRKVMNIT
DMC64           MSTLHANTPRDATARLESMVMMSNASLPLEAIRRNIASAVNIIIQASRLNDGSRKVMNIT
DMC111          MSTLHANTPRDATARLESMVMMSNASLPLEAIRRNIASAVNIIIQASRLNDGSRKVMNIT
                ************************************************************

35000HP         EVMGMENGQIVLQDIFSFEASQHRDENNKIIGKFVNHGLLTRSAVYQNAQVFNLTGELQN
82-029362       EVMGMENGQIVLQDIFSFEASQHRDENNKIIGKFVNHGLLTRSAVYQNAQVFNLTGELQN
6644            EVMGMENGQIVLQDIFSFEASQHRDENNKIIGKFVNHGLLTRSAVYQNAQVFNLTGELQN
HD183           EVMGMENGQIVLQDIFSFEASQHRDENNKIIGKFVNHGLLTRSAVYQNAQVFNLTGELQN
HMC46           EVMGMENGQIVLQDIFSFEASQHRDENNKIIGKFVNHGLLTRSAVYQNAQVFNLTGELQN
HMC56           EVMGMENGQIVLQDIFSFEASQHRDENNKIIGKFVNHGLLTRSAVYQNAQVFNLTGELQN
33921           EVMGMENGQIVLQDIFSFEPSQHRDENNKIIGKFVNHGLLTRSAVYQNAQVFNLTGELQN
CIP542          EVMGMENGQIVLQDIFSFEPSQHRDENNKIIGKFVNHGLLTRSAVYQNAQVFNLTGELQN
DMC64           EVMGMENGQIVLQDIFSFEPSQHRDENNKIIGKFVNHGLLTRSAVYQNAQVFNLTGELQN
DMC111          EVMGMENGQIVLQDIFSFEPSQHRDENNKIIGKFVNHGLLTRSAVYQNAQVFNLTGELQN
```

```
                ******************* ****************************************

35000HP         IFREHAQ*
82-029362       IFREHAQ*
6644            IFREHAQ*
HD183           IFREHAQ*
HMC46           IFREHAQ*
HMC56           IFREHAQ*
33921           IFREHAQ*
CIP542          IFREHAQ*
DMC64           IFREHAQ*
DMC111          IFREHAQ*
                ********
```

**Note:** The TadA sequence of NZS1, NZS2, NZS3, NZS4, and NZV1 were identical to that of 35000HP.

# 23) WecA (HD1844)

```
35000HP       MRRLLKKFKSRYLYAILHICYIILSFRNNFLMWLTFIAVFIVSFASLILMRPVAEKIGLI
33921         MRRLLKKFESRYLYAILHICYIILSFRNNFLMWLTFIAVFIVSFASLILMRPVAEKIGLI
CIP542        MRRLLKKFESRYLYAILHICYIILSFRNNFLMWLTFIAVFIVSFASLILMRPVAEKIGLI
DMC64         MRRLLKKFESRYLYAILHICYIILSFRNNFLMWLTFIAVFIVSFASLILMRPVAEKIGLI
DMC111        MRRLLKKFESRYLYAILHICYIILSFRNNFLMWLTFIAVFIVSFASLILMRPVAEKIGLI
              ********:***************************************************

35000HP       DKPNYRKRHQGLIPLIGGIALFLGNLTFYFIEWQDMRLPWLYLTAVTVLLVIGLLDDRFD
33921         DKSNYRKRHQGLIPLIGGIALFLGNLTFYFIEWQDMRLPWLYLTAVTVLLVIGLLDDRFD
CIP542        DKSNYRKRHQGLIPLIGGIALFLGNLTFYFIEWQDMRLPWLYLTAVTVLLVIGLLDDRFD
DMC64         DKSNYRKRHQGLIPLIGGIALFLGNLTFYFIEWQDMRLPWLYLTAVTVLLVIGLLDDRFD
DMC111        DKSNYRKRHQGLIPLIGGIALFLGNLTFYFIEWQDMRLPWLYLTAVTVLLVIGLLDDRFD
              ** *********************************************************

35000HP       VSPFLRIGLQAGLAGLMIYHGLSLESLGQVIAPFSIKLGILGTVFTILITIGVINAFNMV
33921         VSPFLRIGLQAGLAGLMIYHGLSLESLGQVIAPFSIKLGILGTVFTILITIGVINAFNMV
CIP542        VSPFLRIGLQAGLAGLMIYHGLSLESLGQVIAPFSIKLGILGTVFTILITIGVINAFNMV
DMC64         VSPFLRIGLQAGLAGLMIYHGLSLESLGQVIAPFSIKLGILGTVFTILITIGVINAFNMV
DMC111        VSPFLRIGLQAGLAGLMIYHGLSLESLGQVIAPFSIKLGILGTVFTILITIGVINAFNMV
              ************************************************************

35000HP       DGIDGLLAGLSSASFAGIGVLMWLDEQYSLAYWCFALIVVLIPYAMFNLSLLGAKWKVFM
33921         DGIDGLLAGLSSASFAGIGVLMWLDEQYSLAYWCFALIVVLIPYAMFNLSLLGPKWKVFM
CIP542        DGIDGLLAGLSSASFAGIGVLMWLDEQYSLAYWCFALIVVLIPYAMFNLSLLGPKWKVFM
DMC64         DGIDGLLAGLSSASFAGIGVLMWLDEQYSLAYWCFALIVVLIPYAMFNLSLLGPKWKVFM
DMC111        DGIDGLLAGLSSASFAGIGVLMWLDEQYSLAYWCFALIVVLIPYAMFNLSLLGPKWKVFM
              ***************************************************** ******

35000HP       GDSGSTLIGFTIIWILLLSTQGQGHAISPITGLWLIAVPLIDMVAVVLRRLKKGKSPFRP
33921         GDSGSTLIGFTIIWILLLSTQGQGHAISPITGLWLIAVPLIDMVAVVLRRLKKGKSPFRP
CIP542        GDSGSTLIGFTIIWILLLSTQGQGHAISPITGLWLIAVPLIDMVAVVLRRLKKGKSPFRP
DMC64         GDSGSTLIGFTIIWILLLSTQGQGHAISPITGLWLIAVPLIDMVAVVLRRLKKGKSPFRP
DMC111        GDSGSTLIGFTIIWILLLSTQGQGHAISPITGLWLIAVPLIDMVAVVLRRLKKGKSPFRP
              ************************************************************

35000HP       DRLHLHHLMMRAGLTSRQALAVITLGATICSMIGVFGEYYYWNQWAMTAGFIALFFIYAY
33921         DCLHLHHLMMRAGLTSRQALAVITLGATICSMIGVLGEYYYWNQWAMTAGFIALFFIYAY
CIP542        DCLHLHHLMMRAGLTSRQALAVITLGATICSMIGVLGEYYYWNQWAMTAGFIALFFIYAY
DMC64         DCLHLHHLMMRAGLTSRQALAVITLGATICSMIGVLGEYYYWNQWAMTAGFIALFFIYAY
DMC111        DCLHLHHLMMRAGLTSRQALAVITLGATICSMIGVLGEYYYWNQWAMTAGFIALFFIYAY
              * *********************************:************************

35000HP       SITHAWRMTRFIRRMKRREKRKQHA*
33921         SITHAWRMTRFVRRMKRREKRKQHA*
CIP542        SITHAWRMTRFVRRMKRREKRKQHA*
DMC64         SITHAWRMTRFVRRMKRREKRKQHA*
DMC111        SITHAWRMTRFVRRMKRREKRKQHA*
              ***********:**************
```

**Note:** The WecA sequence of NZS1, NZS2, NZS3, NZS4, NZV1, 82-029362, 6644, HD183, HMC46, and HMC56 were identical to that of 35000HP.
